# Inferring functional modules of protein families with probabilistic topic models

**DOI:** 10.1186/1471-2105-12-141

**Published:** 2011-05-09

**Authors:** Sebastian GA Konietzny, Laura Dietz, Alice C McHardy

**Affiliations:** 1 Max Planck Research Group for Computational Genomics and Epidemiology, Max Planck Institute for Informatics, University Campus E1 4, 66123 Saarbrücken, Germany; 2 Department for Algorithmic Bioinformatics, Heinrich Heine University Düsseldorf, 40225 Düsseldorf, Germany; 3 Department of Databases and Information Systems, Max Planck Institute for Informatics, University Campus E1 4, 66123 Saarbrücken, Germany

## Abstract

**Background:**

Genome and metagenome studies have identified thousands of protein families whose functions are poorly understood and for which techniques for functional characterization provide only partial information. For such proteins, the genome context can give further information about their functional context.

**Results:**

We describe a Bayesian method, based on a probabilistic topic model, which directly identifies functional modules of protein families. The method explores the co-occurrence patterns of protein families across a collection of sequence samples to infer a probabilistic model of arbitrarily-sized functional modules.

**Conclusions:**

We show that our method identifies protein modules - some of which correspond to well-known biological processes - that are tightly interconnected with known functional interactions and are different from the interactions identified by pairwise co-occurrence. The modules are not specific to any given organism and may combine different realizations of a protein complex or pathway within different taxa.

## Background

Cells are complex dynamic systems capable of performing a variety of biochemical processes, many of which are of medical or industrial relevance, such as antibiotic biosynthesis or pathways for plant biomass degradation [1]. Despite the large number of sequenced genomes and metagenomes that are becoming available, our knowledge of the biological processes encoded therein is still limited and process-level genome annotation is far from complete [2-4]. Thus, the lack of high quality functional annotation or knowledge of the functional context for the majority of genes in any given genome/metagenome represents one of the biggest obstacles to obtaining quantitative insights into the relevant biological systems [5,6].

The functional units of signal transduction pathways, metabolic or gene regulatory networks are the products of individual genes, and the analysis of biological processes starts with their identification and characterization. A class of methods known as genome context methods are commonly used to infer the functional relationships between genes. One such method is pairwise co-occurrence (or phylogenetic) profiling [7]. This technique is based on the 'guilt by association' principle [8], which states that genes whose products are functionally coupled are likely to co-evolve and show similar evolutionary histories, resulting in conserved co-occurrence patterns across genomes [9]. The phylogenetic profile of a gene defines the organisms in which orthologs can be found, usually encoded as a binary or a real-valued vector with a length corresponding to the number of genomes considered [7,10,11]. A functional linkage between a pair of genes is predicted if their phylogenetic profiles show pairwise similarity. Commonly used similarity or distance measures are the Hamming distance, Pearson's correlation coefficient, the mutual information and the Jaccard coefficient (for a summary, see [11]). Furthermore, functional coupling is frequently seen in genes that are in spatial proximity to each other in the genome [12]. This can be due to their organization in operons, which allows the joint expression and regulation of functionally related genes. Therefore, conserved gene neighborhoods are a strong predictor for functional coupling [12]. Other genome context methods search for gene fusion events [13], similar expression patterns [14] or shared transcription factor binding sites [15]. In particular, gene fusion events, in which two genes with linked functions have been fused into one gene during evolution, provide substantial evidence for functional linkage. An obvious strategy to improve functional linkage prediction is to combine these methods [16]. This approach is realized in the STRING database [17].

### Functional module detection

A functional module is defined as a set of proteins that jointly participate in a biological process [18,19]. As such, it is likely to be rich in proteins that are functionally coupled in a pairwise manner. If not all proteins, at least some subsets of a module's proteins are likely to be tightly coupled in their function. Accordingly, the proteins involved may map to densely connected subgraphs of protein-protein interaction networks.

A three step approach for detecting functional modules is common practice: First, genome context methods are used to identify pairwise interactions between proteins. Subsequently, the predicted interactions are combined into a functional linkage graph, in which the nodes represent the proteins, and the weighted edges represent the combined evidence for a functional relationship [16,20,21]. Finally, graph-based clustering techniques are used to identify communities of proteins that are likely to be functionally related [22,23]. Many definitions for communities in graphs exist [24]; however, the detection of functional modules essentially corresponds to identification of highly connected subgraphs [25]. Graph-based clustering and problems related to graph partitioning are often NP-hard, but can be tackled by approximate methods with good (though not optimal) results [24].

Watanabe *et al*. [26] used the Bond Energy Algorithm to find clusters of functionally coupled proteins, based on pairwise co-occurrence patterns, without constructing a graph. Their method uses pairwise distances between gene occurrence profiles, measured with the Hamming distance, to identify (disjoint) groups and is able to detect first-order transitive relationships between proteins. However, biological modules need not be disjoint, in general, and a potential limitation of this approach is the greedy nature of the algorithm, which makes the results sensitive to the order of the input data [26].

Besides the aforementioned unsupervised methods, supervised methods such as support vector machines have been applied to identify the proteins of metabolic and signal transduction pathways [27-29]. Note that the selection of genomes may be a critical factor for context analyses, because of phylogenetically conserved signals in the annotation data and a taxonomic bias in the determined genome sequences [30]. Jothi *et al*. studied the influence of genome selection on the results of co-occurrence profiling and suggested to use phylogenetically diverse, non-redundant sets of genomes [10].

Using genome context information in combination with state-of-the-art machine learning approaches is one of the most promising avenues to make progress in functional inference and has not been greatly explored at present [31]. Here, we demonstrate the utility of this approach for functional context inference. In particular, we use a Bayesian method known as Latent Dirichlet Allocation (LDA), which is based on a probabilistic topic model [32]. Topic models are used in text mining applications to reveal statistical relationships between words in collections of text documents, because it was observed that strong relationships usually correlate well with semantic agreement of words. The LDA model has previously been applied to identify protein relationships from MEDLINE abstracts of scientific articles [33,34] and to identify genes with similar behavior in multiple chemo-genomic experiments with *Saccharomyces cerevisiae *[35]. In contrast to this, our method processes large collections of genome annotations to detect functional modules of biological processes with heterogeneous sizes, which allows both small and large processes to be captured. Furthermore, a Bayesian model like LDA promises a robust performance with respect to common noise present in genome annotations, which greatly vary in quality, and may in part be incomplete or incorporate false functional assignments [36]. We applied our technique to a large collection of microbial genome annotations and compared the results with a state-of-the-art pairwise co-occurrence method. Our method identified a largely distinct set of predictions, many of which are supported by known functional interactions from STRING. The set of inferred modules partially maps to known KEGG pathways and the modules indicate a functional context for many protein families of currently unknown function. Our results thus represent a novel source of functional context assignments for protein families.

## Results and discussion

### A Bayesian method for functional module inference

Our method uses Latent Dirichlet Allocation (Methods) for inferring functional modules of biological processes as follows: A set of genome annotations serves as the document corpus, with individual genome annotations representing the documents. We define a fixed-sized vocabulary of words based on the gene annotations, such that words correspond to functional descriptors for gene products, as for instance orthologous groups (OGs) of genes [37], FIGfams [38], Pfam terms [39], EC numbers or other commonly used functional identifiers. Genes that are annotated with a certain functional descriptor represent single instances of the respective word. Note that our method treats genome annotations as a 'bag of functional descriptors', meaning that the order of genes in the genome sequence is not considered.

**Figure 1:**
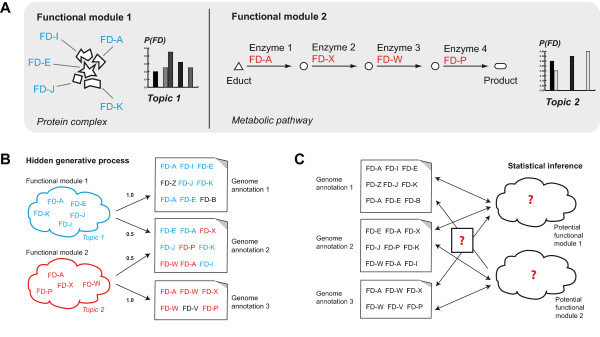
**The LDA model assumes a hidden generative process that can be inversed for statistical inference**. In our approach, topics are assumed to represent the unknown biological modules that have shaped the contents of genomes. As a simplifying example, the influence of two modules on the contents of three genome annotations is considered. ***Panel A: ***Functional descriptors (*FD *terms) are associated with proteins in the modules, and each module is represented by a probability distribution over *FD *terms. ***Panel B: ***The hidden generative process: Genome annotations are assumed to be generated from weighted mixtures of the probability distributions. The two clouds show the *FD *term set with the highest probabilities for each module. Note that the second genome annotation is equally shaped by both modules, whereas the other two annotations are solely shaped by one module. ***Panel C***: The input data as seen by our method. No *a priori *knowledge about the underlying modules is necessary. The potential functional modules are latent variables of the model that will be inferred from the collection. The identified modules are not necessarily specific to any given microbe, but potentially combine different realizations of a complex or pathway from different organisms.

The collection of functional descriptors, grouped into genome documents, serves as input to LDA, and Gibbs Sampling is used for model inference (Methods). So-called topics represent the latent variables of the LDA model and their values are inferred from the collection of genome annotations (Figure 1). Each inferred LDA topic defines a probability distribution ('topic distribution') over the chosen vocabulary. Functional descriptors with high probabilities show similar co-occurrence patterns within the collection of annotations. According to the 'guilt by association' principle, the inferred topics are likely to represent functional modules - sets of protein domains or orthologous groups of genes that are functionally linked to each other.

We used the *k *obtained topic distributions to define potential functional modules (PF-modules): Each PF-module is defined by a single topic and comprises the set of functional descriptors selected from the topic distribution by applying a threshold value *C*. For our experiments, we used *C *= 0.01. This choice of *C *was guided by visual inspection of the topic distributions, in accordance with [35]. In this study, identifiers for orthologous groups (OGs) of genes, i.e. COG and NOG terms from the eggNOG database [37], were used as input vocabulary for LDA. As such, the inferred potential functional modules correspond to groups of conserved gene families.

### Functional module inference from prokaryotic genomes

We applied our method to 575 prokaryotic genome annotations from the STRING database, and tested it for different settings of *k *(*k *= 100 - 500), with three independent runs for each setting. We achieved the best results for *k *= 200 and *k *= 400 (see discussion below), and discuss results for *k *= 200 in more detail. To obtain a reliable estimate of model stability for *k *= 200, we performed six additional runs and averaged numerical results over the PF-module sets of all nine runs.

The largest PF-module consists of 61 (standard deviation (s.d.) 6.34) OGs; the average module size is 19.4 (s.d. 0.26). Based on the module sizes, we would expect approximately 3,880 OGs to be associated with the modules. However, the inferred modules overlap in OG content, as only 1,554.3 (s.d. 38.2) distinct OGs on average are forming the set of modules of a run. In total, 868 OGs were consistently associated with the modules for all nine runs. These are likely to represent a core of conserved gene families with strong co-occurrence patterns in the data.

In accordance to the similar module size distributions over the nine runs, we also observed little variation over runs based on other evaluation criteria, discussed below. In the following, we therefore discuss the results from a randomly chosen, exemplary run with *k *set to 200. In this run, 198 non-empty PF-modules were identified (Additional file 1, Tables S1-198); for two topic distributions, no OGs exceeded the probability threshold *C*. Of these 198 modules, 70 particularly stable PF-modules could be tracked over all nine runs (Additional file 1, Tables S1-70). The average size of the 70 stable modules is 15 OGs, and of the 1,532 OGs associated with all 198 modules of the exemplary run, 43.9% are part of these stable modules. Note that the tracking of topic identities over runs follows a greedy heuristic strategy and may underestimate the true number of stable PF-modules (Methods).

We analyzed the functional consistency of the 198 modules in terms of their enrichment in COG functional categories [40]. On average, the most frequent functional category present in a module (with a minimum module size of seven OGs) is associated with 35% of the contained OGs (41.1% for the stable modules). This implies that modules are heterogeneous, but often include a significant portion of OGs from the same functional category. Interestingly, one of the most abundant categories is general function prediction only (histograms in Additional file 2), which contains OGs with insufficiently characterized functions. Thus, placement in a functional module might further indicate the functionalities for these gene families.

We could map 15 of the stable modules (Table 1) and 49 of all modules of the exemplary run (Additional file 3, Table S1) to KEGG pathways, based on six or more matched KO terms in the respective pathway. Overall, the modules contained many interactions annotated in the KEGG database. However, this only explained a part of the PF-modules, so we evaluated the functional coherence of the identified groups by means of additional quantitative measures.

**Table 1:** Profile of KEGG pathways with at least six matches to one of the stable modules

KEGG name	Modules
ABC transporters	5

Flagellar assembly	1

Porphyrin and chlorophyll metabolism	1

Oxidative phosphorylation	1

Bacterial secretion system	2

Two-component system	2

Phenylalanine, tyrosine and tryptophan biosynthesis	1

Phenylalanine metabolism	1

Starch and sucrose metabolism	1

The modules were identified as being stable across nine independent runs of our method with *k *= 200.

### Functional coherence of modules

For the analyzed genomes, the STRING database provides evidence for pairwise functional couplings of OGs related to different types of functional modules, such as metabolic and signal transduction pathways, as well as protein complexes. The data include predictions from several genome context methods, as well as functional relationships from public protein-protein interaction and metabolic pathway databases. For our evaluation, we exclusively selected pairs of OGs that were sufficiently supported by evidence other than co-occurrence profiling (Methods). Overall, the reference dataset with this restriction comprised 60,880 distinct OG pairs. To evaluate the functional coherence of a module, we considered all possible pairs of OGs within the module, and determined the percentage of these found in the reference set. Module derived OG pairs that are part of the reference are referred to as *verified pairwise functional couplings *(verified-PWF-couplings). A high proportion of verified pairs then indicate the functional coherence of a module.

**Figure 2:**
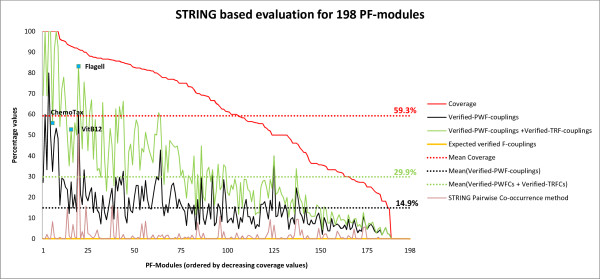
**Overview of the STRING-based evaluation of 198 modules**. We evaluated 198 PF-modules from a randomly chosen run with *k *= 200. Coverage values for the modules serve to assess their functional coherence. A coverage value of 100% means that the complete OG set of a module is interconnected within the reference functional network and forms a single cluster therein. If the coverage is 50%, then the same holds for half of the OGs of a module. The plot shows coverage values for all 198 modules of the exemplary run (modules 'ChemoTax', 'Flagell' and 'VitB12' are discussed in detail in the Results section). '*Verified-PWF-couplings*': The percentage of *verified pairwise functional couplings *with respect to all tested OG pairs of a module. '*Verified-PWF-couplings + Verified-TRF-couplings*': The percentage of a module's OG pairs that are either verified pairwise couplings or *verified first-order transitive functional couplings*. '*Expected verified F-couplings*': The expected percentage of verified pairwise couplings to be found by chance for the OG set of a module. For an average-sized module, we expect to obtain less than one (*E*[*h*] = 0.19) verified pairwise functional coupling by chance. The dashed lines indicate mean values, and the averaged mean coverage over all nine runs is 57.9% (1.3% s.d.). Finally, we determined the fraction of OG pairs within a module which are verified and have also been predicted by the pairwise co-occurrence method used by STRING.

The average percentage of verified pairwise functional couplings for all 198 modules of the exemplary run is 14.9% (Figure 2), and the individual results for the modules are highly significant according to an estimate based on the hypergeometric distribution (Methods). Given the size of the input vocabulary (10,431 OG terms), the probability of an arbitrary OG pair matching a pair from the reference set by chance is *P_hit _*= 0.0011. For an average-sized functional module, which consists of 19 OGs and 171 OG pairs, one would expect to observe less than one match by chance (*E*[*h*] = 0.19). Thus, even small numbers of verified couplings are highly significant.

**Figure 3:**
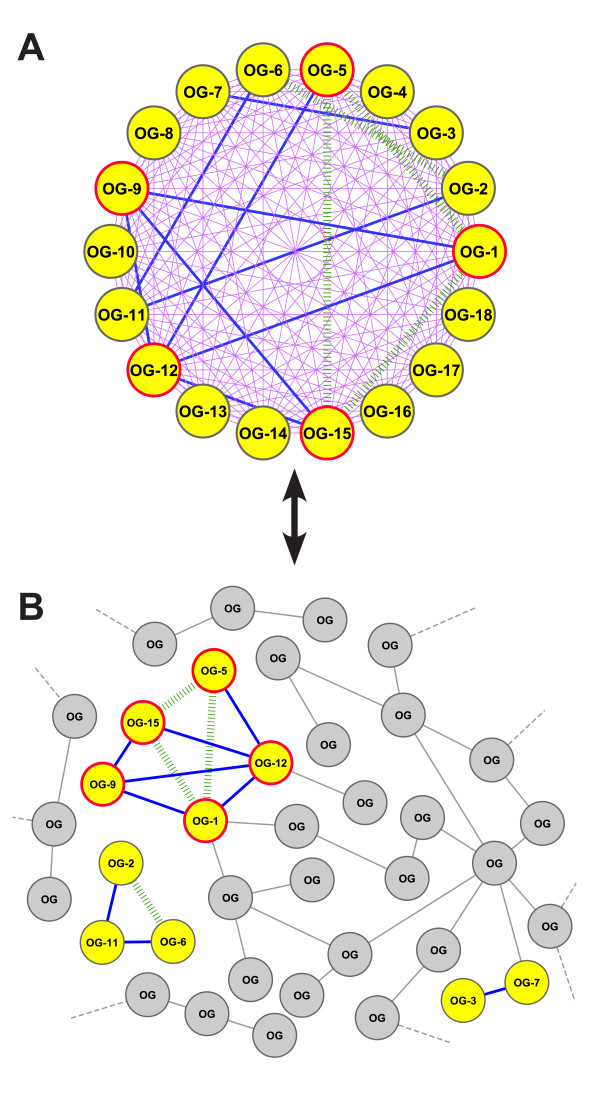
**Evaluation scheme for the inferred potential functional modules**. ***Panel A: ***A group of 18 OGs, representing an average-sized module. To assess the functional coherence of the module, all possible OG pairs of the module (153 pairs, illustrated as *purple *lines) are matched against a high confidence reference set of OG pairs from STRING. ***Panel B: ***The functional network spanned by the reference OG pairs. Pairwise interactions in the network that are matched by OG pairs of the module are marked as *blue *edges. In contrast, the majority of linkages in the reference network are marked in *gray*, indicating that they are not matched by any pair from the module. In each case where three module OGs are exclusively connected by two blue edges, we presume evidence for a transitive relationship (*green *edges), even though the third edge that completes the triangular relationship is not contained in the reference set. Thus, given the 153 tested pairs, the module yields nine verified pairwise interactions, plus four additional (first-order) transitive interactions. Note that in this case, the module covers three connected components in the network. The five OGs marked with *red *boundaries are part of the largest connected subcomponent, resulting in a coverage value of 5/18 = 27.8%.

The proteins of a functional module may not directly interact, but be transitively linked to each other instead. Therefore, we also searched for indirect relationships. The OG pairs in the reference set correspond to edges in a functional network defined by high confidence interactions in STRING. We matched the OG pairs of a module against this network. If a pair exists in this reference network, it may either be an isolated edge or an edge connected to other matched edges. Ideally, all OGs of a module are functionally related and form a single connected component within the network. This would mean that they are either directly or indirectly functionally linked to each other. We denote the fraction of a module's OGs that are part of the largest connected component in the reference set as the module's *coverage *(Methods; Figure 3). Thus, if a module has coverage of 75%, this means that three-fourths of the module's OGs are either directly or indirectly linked to each other.

In total, we found 132 modules with an average size of 20.8 OGs and coverage of more than 50%. We also evaluated the first-order transitive relationships (*verified-TRF-couplings*), for which the total fraction of verified pairs rose from 14.9% to 29.9% (Figure 2).

We subsequently investigated how the validated pairs of the modules are dispersed within the reference network, i.e. whether they form isolated edges or highly-connected components. To this end, for each module, we determined the number of its connected components within the reference network. More than half of the modules map to only one such component; the average is less than two (1.67) components per module. Thus, the majority of modules represent large clusters within the reference network, with only a few isolated edges.

### Modules with matches to KEGG pathways

**Figure 4:**
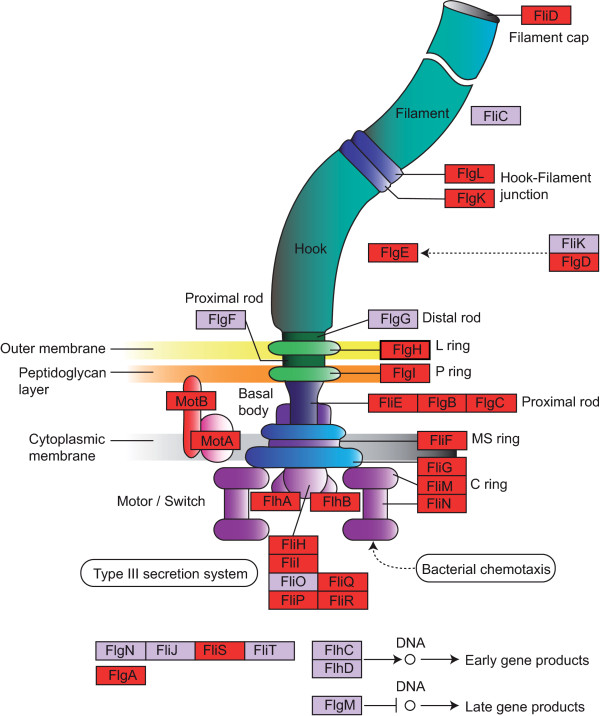
**Mapping of the 'Flagell' module to the KEGG map 'flagellar assembly'**. OGs of the 'Flagell' module are mapped to the KEGG map. Matched items are highlighted in *red*. This image is adapted from the original KEGG map.

We found three interesting modules (named 'ChemoTax', 'Flagell' and 'VitB12') among the stable modules in all nine LDA runs. These have a COG category functional enrichment of more than 50%. The first two modules are related to chemotaxis and the flagellar apparatus. 'ChemoTax' consists of 16 OGs and is very rich in signal transduction genes (OGs in the category T, 50%; Additional file 1, Table S1). Strikingly, this module achieves a coverage of 100%. Of the 67 verified OG pairs (39 verified pairwise couplings + 28 verified transitive couplings), only 10 were also detected by co-occurrence profiling. The larger 'Flagell' module consists of 35 OGs and is rich in cell motility genes (category N, 86%; Additional file 1, Table S2). This module has a coverage value of 91%. In the 'Flagell' module, we identified 355 verified pairwise and 140 verified transitive functional couplings, whereas pairwise co-occurrence profiling identified 301 of these 495 couplings. Interestingly, 'ChemoTax' and 'Flagell' share two gene families (*COG0835 *and *COG2201*), which are both assigned to the functional categories for signal transduction (category T) and cell motility (category N). 'ChemoTax' and 'Flagell' thus may capture different aspects of a biological network related to chemotaxis and cell motility. While 'ChemoTax' does not correspond to a KEGG pathway and mainly consists of the OGs responsible for signal transduction, 'Flagell' comprises structural components of the flagellar apparatus and contains most of the elements of the respective KEGG map (Figure 4). Indeed, the relationship between the flagellar apparatus and chemotaxis is well known [41,42].

Furthermore, we identified a module comprising 32 gene families (Additional file 1, Table S3) that largely maps to the KEGG pathway 'Porphyrin and chlorophyll metabolism' (24 matches, KEGG map in Additional file 4). The module almost completely covers the process of synthesis from precorrin-2 to the vitamin B12 coenzyme, and we therefore refer to this module as 'VitB12'. The module has a coverage of 93.8%, while pairwise co-occurrence only predicted 29.8% of the 245 verified pairwise or transitive functional couplings. It should be noted that 'VitB12' clearly represents a meaningful module, although its 118 OG pairs with direct matches in the reference set cover only 23.8% of the tested pairs. Most supported pairs for this module originate from the 'database' and 'neighborhood' channels of STRING.

Another interesting stable module of 66 OGs that comprises ribosome-related gene families was found for *k *= 400 in all nine runs (Additional file 5, Table S1; the module was not found in all runs for *k *= 200; however, a corresponding module found in the exemplary run with *k *= 200 is presented in Additional file 1, Table S123). The module maps to the KEGG reference pathways 'Ribosome' (29 OGs, KEGG map in Additional file 6) and 'Aminoacyl-tRNA biosynthesis' (13 OGs). The functional enrichment for category translation and ribosomal structure (J) is 73%, and four gene families represent translation factors. Thus, our method is capable of identifying modules with a larger functional context than a single KEGG pathway. Interestingly, the module has a coverage value of 97% and none of the interactions were found by pairwise co-occurrence. Note that this module is not the only module related to the ribosome KEGG pathway. Several other modules map to pathways that are involved in protein biosynthesis. The number of verified pairs for all presented modules was highly significant according to our significance estimate (*P_mult_*_*_hit_*≤0.001).

### Functional coherence of the modules in dependence of LDA parameter k

We evaluated the impact of *k*, the number of topics to be inferred, in a series of experiments with *k *set to 100, 200, 300, 400 and 500 (Table 2). Each experiment comprised three independent runs. The mean module size increased notably from *k *= 100 (13.82) to *k *= 200 (19.47), whereas it remained similar for *k *= 200, 300, 400 and 500. Interestingly, the functional coverage values only varied slightly for different settings of *k*, although the number of modules and their sizes steadily increased. This suggests that most of the identified modules for different settings of *k *were supported by evidence from STRING. The number of stable modules is larger for *k *= 400 and *k *= 500 than for runs with smaller values of *k*, and these modules also contain more distinct OGs. Thus, for larger values of *k*, a larger part of the STRING reference interaction network is identified. We additionally matched the stable modules from each experiment to the KEGG database to identify pathways with six or more hits to any one of the modules. For *k *= 200 and *k *= 400, this resulted in the most diverse profiles with matches to 20 different KEGG pathways. We decided to use a setting of *k *= 200 for a further detailed analysis, which included six additional runs, because this setting showed good results with respect to identified KEGG pathways and the largest support by known functional interactions; in terms of the fraction of identified stable modules with a coverage of at least 50% (Table 2). Further details of this comparison are discussed in a Supplementary note in Additional file 7.

**Table 2:** Comparison of results for varying numbers of inferred topics

*k*	Mean # OGs associated with the modules		Mean module size		Mean coverage for *k *modules		Stable modules with ≥5 OGs and coverage ≥50%
	**Average**	**s.d**.	**Average**	**s.d**.	**Average**	**s.d**.	

100	635	28.6	13.82	0.27	64%	1%	33 (33%)

200	1560	24.9	19.47	0.11	58%	1%	66 (33%)

300	2009	31.1	21.76	0.9	56.7%	0.6%	68 (22.7%)

400	2223	33.9	22.9	0.42	53%	1%	102 (25.5%)

500	2378	7	22.34	0.19	49%	0%	97 (19.4%)

We performed 5 experiments to test different settings of *k*. The reported numbers for each experiment are averaged mean values of three runs. We used a greedy approach to track module identities across the runs for each setting of *k*, and refer to the modules identified in all three runs as the stable modules. The last column gives the number of stable modules with sufficient evidence for functional coherence based on the STRING analysis (in parentheses, we denote the fraction of stable modules satisfying the conditions, with respect to *k*).

### Comparison with pairwise co-occurrence profiling

**Figure 5:**
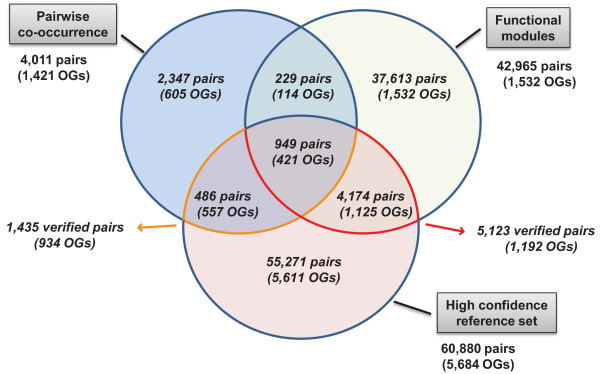
**Pairwise predictions deduced from 198 modules compared with predictions of the co-occurrence baseline method**. The Venn diagram visualizes the overlaps between the different OG pair sets. The partitions are defined over OG pairs. For each section of the Venn diagram, the number of distinct OG terms defined as parts of the pairs is noted. Note that the OG sets of the single sections are not necessarily disjoint. A large total of 5,123 pairs have been validated for the modules. Additionally, 7,603 first-order transitive relationships could be validated based on the reference set (not included in the Venn diagram). Based on our estimate *E*[*h*] for the expected number *h *of random matches for a single module, we would expect less than 50 matches to the reference set for the 42,695 tested pairs of the modules by chance.

We compared our method with the state-of-the-art pairwise co-occurrence profiling method used in STRING. Pairwise co-occurrence identified a small fraction of the verified pairwise or first-order transitive couplings of the modules (Figure 2, Figure 5). The Venn diagram in Figure 5 shows the overlap with the reference set for both methods. Overall, both methods detected a small subset of the 60,880 reference pairs, resulting in recall rates of 8.4% for the modules and 2.4% for pairwise co-occurrence. These rates suggest that, in general, co-occurrence patterns contribute different evidence for functional linkage than other available sources of information about functional linkages. The PF-modules cover a largely distinct set of interactions, which includes 66.1% of the validated predictions of the pairwise method. The 4,174 validated functional couplings exclusive to the PF-modules exceed the overall number of linkages predicted by pairwise profiling. We mapped these 4,174 pairs to KEGG and found matches for 34.6% of them. The most abundant KEGG pathways were 'Ribosome', 'Two-component system' and 'Oxidative phosphorylation'.

For a complete evaluation, the precision, i.e. the fraction of correct assignments of all predicted linkages should be determined. However, this is complicated by the fact that non-existing interactions (according to the reference set) may reflect incomplete knowledge rather than the absence of interaction. Furthermore, solely for the sake of comparison with pairwise co-occurrence, all possible pairs were enumerated as functional linkage candidates within each module, which is not an assumption warranted by our method and results in a large number of pairwise interactions being tested. With these restrictions being applied, precision values for both methods are 35.8% (pairwise co-occurrence) and 11.9% (functional module inference). This value may serve as an estimate of the lower bound of the actual precision for functional module inference. As pairs of gene families in a module may also interact indirectly with each other, they may not directly match a pair of the reference set. When taking these indirect interactions in the reference set into consideration (verified-TRF-couplings), we found evidence for 7,603 OG pairs that represented further functional relationships implicitly verified by STRING. Combined with the 5,123 directly verified pairs, this corresponds to 29.6% of the tested 42,965 pairs and may serve as a second estimator of precision for the presented functional module inference.

The capacity to identify indirect relationships highlights an important advantage of directly inferring groups of functionally related OGs. Of the identified transitively linked pairs, 828 could be mapped to KEGG pathways. Interestingly, the fraction of verified-TRF-couplings also serves as an indicator for meaningful relationships revealed by the modules that are not directly identified by any of the pairwise operating genome context methods used in STRING. As such OG pairs are not explicitly part of the reference set, none of the prediction methods in STRING provided sufficient evidence for their direct coupling. However, as demonstrated, a transitive relationship exists in the reference.

**Figure 6:**
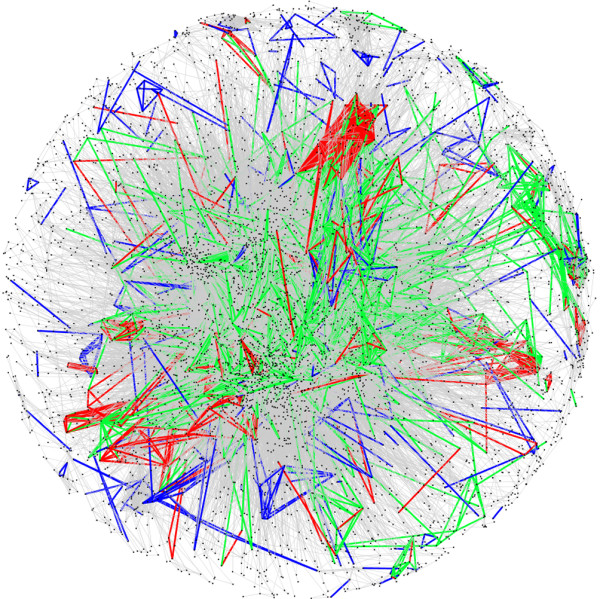
**Visualization of the functional network spanned by the OG pairs of the reference set**. The figure shows the pairwise functional interactions defined by the reference set as edges between OGs in a network graph. The subset of verified pairwise predictions from the modules is shown in *green*, whereas the subset of verified predictions by pairwise co-occurrence profiling is shown in *blue*. Functional interactions that are predicted by both methods are colored in *red*, and those not detected by any of the methods are shown in *gray*.

The modules cover parts of the reference network that tend to be tightly interconnected. Figure 6 visualizes the densely connected core of the functional network defined by the reference set, showing the embedded prediction sets of both methods (a picture of the complete network is provided in Additional file 8). The modules that we have discussed are embedded in this network, showing that our method is capable of identifying meaningful groups of OGs in a dense interaction network.

## Conclusions

We proposed and evaluated a new probabilistic method for directly identifying functional modules of gene or protein families using co-occurrence patterns in a collection of annotated sequence samples. In our analysis, we used orthologous groups of genes (OGs), which are considered to be a reliable estimator for isofunctional groups of genes [38,40]. However, one could also use terms such as FIGfams, which incorporate careful manual curation by experts [38], KO terms, Pfam domains, TIGRfam terms or EC numbers [39,43].

The presented method is capable of simultaneously processing a large number of genome or metagenome annotations. We tested our methodology on a comprehensive set of microbial genomes; but certainly a targeted selection of a suitable collection of genome annotations for input will be a major key to the detection of further interesting PF-modules in the future.

Our method returns a soft clustering of functional annotation terms, in which a term can be assigned to multiple modules. This is well suited for the problem of assigning gene families to biological processes, given the multiplicity of roles and functionalities associated with some gene families, which may depend, for instance, on the genomic context [3]. Furthermore, processes may appear in multiple, slightly different variants across genomes. Such process variations arise, for instance, through alternative branches in metabolic pathways, or through reactions that can be realized by structurally different proteins [44,45]. The topic model that we use accounts for this phenomenon adequately. LDA topics are globally defined for the analyzed collection of genomes and therefore generalize over slightly different variants of a process, instead of splitting them into multiple modules. Thus, a functional module may combine multiple isofunctional but non-orthologous gene families, which fulfill similar roles in different organisms.

LDA is an unsupervised method, which requires no *a priori *knowledge about the structures it identifies. This gives us the opportunity to detect previously unknown functional modules. However, this also means that the nature of the biological entities captured by these modules is uncertain. For instance, metabolic and signal transduction pathways, protein complexes or mixtures of these might be the underlying biological signals.

We evaluated the biological significance of the identified modules using functional interactions from STRING, which integrate different sources of biological information about functional modules. As a result, we found that PF-modules cover diverse biological signals, such as protein complexes ('Ribosome' and 'Flagell'), signal transduction components ('ChemoTax') and metabolic pathways ('VitB12'). A convenient property of the method is that the input data are represented as 'bags of gene families' and thus no knowledge of the neighboring genes is required. Therefore, it can also be applied to highly fragmented metagenomes with many short fragments, for which there is currently a shortage of analysis techniques [5,46,47].

In our study, we observed that the identified potential functional modules are significantly enriched with high confidence functional interactions and capture well-known biological processes, such as chemotaxis. Moreover, the majority of the modules' high confidence interactions were not detected by a state-of-the-art pairwise co-occurrence method.

Notably, a great number of newly implied OG interactions, derived from the PF-modules, could not be verified as pairwise interactions, but received reasonable support as first-order transitive relationships from STRING instead. And many of these interactions could also be mapped to KEGG pathways. In summary, this shows that an approach of direct inference of functional modules reveals further information about biological processes. We believe that the direct inference of groups of genes is well suited for the discovery of functional context, as biological processes incorporate many indirect functional couplings between the encoded proteins. For instance, proteins may serve as network hubs that link two or more processes. In this case, proteins from the processes involved will be directly coupled to the hub and only indirectly to the proteins of the other processes. Finally, in many cases, the proteins of a particular process vary between different organisms [44,45]. These scenarios result in proteins that are only transitively linked to each other via other proteins of a process.

The inference procedure of the LDA model is based on a Markov Chain Monte Carlo method. With a limited number of iterations, such methods are non-deterministic, i.e. a series of LDA runs will not produce exactly the same sets of PF-modules. At the chosen stop point of iterations, we observed that the size distribution as well as the degree of functional coherence over the module sets varied only slightly between runs, indicating that there is sufficient convergence of the Markov chain. Our heuristic search strategy allowed us to identify 70 stable modules across the nine runs we performed. However, many of the other PF-modules also occur in more than one run. We investigated the relationship between stability and functional coherence. The average coverage over all 198 PF-modules was 59.3%, while the 128 less stable PF-modules showed an average coverage of 61.1%. Thus, also the less stable modules contribute significantly to the overall estimate of functional coherence.

An interesting future direction for research will be the use of the genome-specific topic-weights to investigate how the gene family content of a functional module varies in individual genomes (Supplementary note in Additional file 7, heatmap in Additional file 9). We found that the OGs of the modules are associated with almost all the COG functional categories and that OGs with no specific functional assignment (category R) are frequent. Therefore, we could use modules to refine gene annotations in the following way: Assuming that a given module shows evidence for being related to a specific biological process and that the module is assigned a high weight for a genome of interest. Then, poorly characterized genes of this genome, whose gene families are associated with the module, could be tentatively associated with this biological process. Methods for assessing the function of poorly characterized genes from putative interaction partners are currently being investigated [31]. Another interesting research direction will be to analyze the evolution of functional groups, based on the presence or absence of (parts of) a module across taxa.

Prediction methods for functional relationships that rely on conserved genomic context are prone to false positive predictions if pseudo-genes are involved [48,49]. Pseudo-genes are functionally disabled copies of genes, which typically occur in at least 1-5% of gene-like sequences in prokaryotic genomes [50]. We found several PF-modules that integrate different transposable elements. In these cases, pseudo-genes might have had an impact on the inferred modules. LDA, like other co-occurrence techniques, could principally be misguided in cases where multiple copies of an OG reside in the same genome by chance without being retained by selection for a certain functionality, e.g. due to repeats of a genomic sequence.

We compared results for different choices of the number of topics to be inferred, and suggest choosing a setting for *k *between 200 and 400 for the analyzed set of genomes. Finding an optimal choice for the number of topics corresponds to the problem of model selection in latent class cluster analysis [51]. Blei *et al*. [52] proposed tackling this problem by modeling a hierarchy of topics and embedding LDA into a Hierarchical Dirichlet Process [52,53]. However, the additional level of complexity in these models is likely to cause the inference process to be more time-consuming, and convergence of the process has to be carefully monitored.

In summary, we found that our method allows identification of well-known biological processes, as well as the discovery of new modules supported by high confidence functional interactions. It furthermore places many gene families of currently poorly characterized function within a functional context. The presented technique could thus help to enhance our knowledge of the biological processes governing microbial life and reveal new functional connections for many microbial genes.

## Methods

### Technical aspects of the LDA model

LDA assumes the existence of a fixed number *k *of underlying 'topics' that define the essential semantics of the whole text corpus. Each topic *t_i _*(with *i*∈{1,...,*k*}) defines a probability distribution ('topic distribution') over the vocabulary *V *of words: *P*(*w*|*t_i_*) for *w*∈*V*. Words with high probabilities under a topic distribution are statistically linked to each other, i.e. they have similar co-occurrence patterns with respect to the document collection. This way, each topic defines a specific grouping of words that are thought to be semantically related. However, the topics represent latent variables of the LDA model that need to be inferred from the input data.

LDA uses *k *multinomial distributions with Dirichlet priors to model the topics. These distributions are globally defined for all documents of the collection, but the model assigns weights to the individual topics for each document. For each document *d_j _*(with *j*∈{1,... *D*}), a set of probabilities *P*(*t_i_*|*d_j_*) exists for *i*∈{1,... *k*}, which represent the weights. The underlying assumption is that the documents are the result of a hidden generative process, in which the observed word frequencies have been generated from a document-specific, weighted mixture of the topic distributions. This relationship between observed word frequencies and the topic distributions is reflected in *P*(*w*|*d_j_*), which is the probability of observing a certain word *w*∈*V *as part of document *d_j_*:

The word content of a single document *d_j _*mainly corresponds to a set of words contributed by the subset of topics with the highest probabilities *P*(*t_i_*|*d*). Note that words may have high probabilities in multiple topic distributions. Such ambiguous words are thus related to more than one topic. However, depending on the document in which an instance of a word appears, one may assess the word's correct topic affiliation based on the document-specific weighting of topics. To this end, LDA also offers a probabilistic framework that enables the user to estimate the probabilities for assigning a word instance to certain topics, depending on the document in which it occurs.

For inference of the latent model parameters, Markov Chain Monte Carlo (MCMC) techniques [54], such as Gibbs sampling, can be applied [55]. At the end of the inference process, one obtains the topic distributions and document-specific probability weights for the topics.

#### Monitoring stability of the inferred model

MCMC sampling techniques efficiently estimate the posterior distribution over model parameters [54]. However, the actual time needed for convergence cannot be estimated precisely, and the efficiency of the sampling depends on the complexity of the model and the analyzed data. To assess the convergence of the inference process, a commonly used approach is to compare the results from a number of runs. We used the symmetrized version of the Kullback-Leibler divergence (KL divergence):

defined for two distributions, *p *and *q*, to assess the stability of the inferred topic distributions across the final results from different runs [56]. Using KL divergence, we performed an all-against-all similarity comparison between the topics of two different runs. Then, topics that showed the smallest distances from each other were mapped between runs with a greedy best first search. Note that this easy to implement algorithm identifies a local optimum which does not necessarily represent a globally optimal solution in terms of the minimal KL divergences between the topics in all formed pairs. A best first search strategy may disregard suboptimal choices of pairs that would allow an improved overall mapping, due to improved mappings of other topics. If a mapping was circularly closed over *N *different runs (i.e. topic *i *of run 1 mapped to topic *j *of run 2, topic *j *mapped to topic *k *of run 3, and so on, until topic *l *of run *N *mapped back to topic *i *of run 1), we say the topic behaved consistently across these runs. We refer to topics that we could track consistently across all performed runs as stable topics.

### Input data and preprocessing

Genome annotations for 575 prokaryotic genome sequences were downloaded from the STRING database (version 8.2) [17]. The number of distinct OGs (COG/NOG terms) in this dataset is 47,993. We removed all terms appearing in less than 10 genomes, which reduced the vocabulary to 10,431 distinct OGs. This filtering step facilitated evaluation with respect to the computational requirements. In subsequent experiments, we found that removing this restriction does not significantly change the results (data not shown).

#### Parameter settings for LDA runs and availability of the LDA implementation

Our method uses the LDA implementation available at http://gibbslda.sourceforge.net/, which relies on Gibbs sampling [55]. The LDA model defines two hyperparameters, *α *and *β*, which specify the underlying Dirichlet prior distributions. LDA was run with 2,500 iterations and the following parameter settings: *k *= 200 (the number of topics), *α *= 0.5, *β *= 0.01. The experiments were repeated nine times to ensure stability of the results.

### Evaluation

#### Construction of a STRING-based reference set of high confidence interactions

The STRING database provides information about pairwise functional couplings of OGs for the analyzed set of genomes. Supporting evidence for each OG pair can stem from seven sources (channels): 'neighborhood', 'fusion', 'co-occurence', 'coexpression', 'experimental', 'database' and 'textmining'. For each evidence channel, a score quantifies its reliability. These reliability estimates were derived by benchmarking the predictive performance of the individual channels against a reference set of protein associations from the KEGG database [16]. STRING also provides a 'combined score' as an integrated measure of support from all evidence channels [17].

The STRING data (version 8.2) define 6,007,943 distinct pairs of OGs as being functionally coupled, based on a combined evidence score of 0.15 or more. Combined scores in the range of [0.4-0.7] represent a medium level of confidence, whereas the range of [0.7-1.0] denotes a high level of confidence [17]. We followed the procedure described in [17] to combine information from the different channels without co-occurrence information into a combined score, using a script provided by the database maintainers http://bitbucket.org/mkuhn/stringtools/src/tip/prior_correction/discard_channels_cogs.py. Thus, the modified combined scores represent evidence for pairwise functional coupling independent of co-occurrence. This procedure resulted in 2,472,604 remaining pairs, because discarding information from one channel decreases the overall combined scores, and, as a result, some fell below the threshold of 0.15. We used the modified combined scores to define a high confidence reference set of known pairwise functional couplings. Our reference comprises all OG pairs from STRING for which *(i) *both OGs are present in the input vocabulary *V *and *(ii) *the modified combined score is at least 0.7. The resulting reference set consisted of 60,880 unique OG pairs (Additional file 10).

#### Assessing the functional coherence of the potential functional modules

For each PF-module, we determined the set *S_PFM _*of all possible unique OG pairs. For a PF-module of size *l*, the number of pairs is *m*=|*S_PFM_*| = (*l*·(*l*-1))/2. We then tried to identify as many of these pairs as possible within the reference set of pairs and refer to matches as *verified pairwise functional couplings *(verified-PWF-couplings). Furthermore, for each OG pair of a PF-module without a direct match to the reference set, we searched for a third OG from the respective module with a verified-PWF-coupling to both OGs of the original pair. OG pairs that were validated by this approach are referred to as *verified first-order transitive functional couplings *(verified-TRF-coupling). Additionally, we computed the transitive closure for each module with respect to the functional network spanned by the OG pairs of the reference set. Therefore, we determined the set of verified pairwise functional couplings for the OG set of the respective module, and used this set of pairs as edges between OG nodes to construct an undirected graph. Finally, we determined the connected sub-components of the graph, and used the percentage of the module's OGs that were part of the largest connected component as an estimate for the module's functional coherence. We refer to this value as the module's *coverage*.

#### Significance estimation

The data from STRING allow us to verify pairwise OG interactions. We assessed the statistical significance of finding *h *OG pairs with matches to the reference set of interactions among the pairs of a PF-module. We used the hypergeometric distribution to estimate the probability of observing this result by chance:

Let *D *be the set of all possible unique pairs of OGs for the input vocabulary *V*. Given a vocabulary of size |*V*|, the number of pairs in *D *is |*D*| = (|*V*|·(|*V*|-1))/2. Further, let *U *be the reference set of OG pairs. |*U*| denotes the size of the set. Due to the construction rules of the reference set, *U *⊆ *D *holds and therefore a random pair in *D *will also be part of *U *with probability . If we count the observation of a match to the reference set as a success, matching a random OG pair against the reference can be regarded as a Bernoulli experiment with the success probability *P_hit_*.

Now, since *S_PFM _*represents the set of unique OG pairs of a PF-module, let *m *= |*S_PFM_*| be the size of this set. We used the hypergeometric distribution to assess the probability of achieving *h *verified-PWF-couplings for *S_PFM _*by chance. The subset *U *of *D*, comprising the OG pairs defined in the reference, represents the set of possible successes if we randomly draw from the population *D*. Thus, the probability *P_hyper _*(*X *= *h*) of observing *h *successes by chance, given that we draw *m *times from the population *D *without replacement is:

This gives the expected number of random matches for a set *S_PFM _*as .

Finally, the cumulative distribution function *P*(*X*≤*x) *of the hypergeometric distribution was used to estimate the probability of observing *h *or more hits among *m *randomly formed query pairs. This is *P_mult_hit_*(*h*) = *P*(*X*≥*h*) = 1-*P*(X≤*h*-1).

#### Comparison with a state-of-the-art co-occurrence method

The pairwise co-occurrence profiling method of STRING evaluates the mutual information between OG profiles and also accounts for biases in the data caused by phylogenetic relationships between genomes [16]. We determined the predictions of the pairwise profiling method for our comparison as follows: Starting with all OG pairs provided by STRING, we first removed all pairs containing OGs which were not part of the vocabulary *V *in our analysis. Then, we determined all OG pairs with a score of at least 0.4 from the 'co-occurrence' channel. This score threshold corresponds to the lower bound of the medium confidence interval for STRING scores.

#### Mapping OG identifiers to KEGG pathway maps

KEGG pathway maps are defined for orthologous groups of genes. However, these maps are based on KEGG-specific identifiers for such groups, known as KO (KEGG Orthology) terms [57]. To map OG identifiers from the eggNOG database to KO identifiers of the KEGG maps, a mapping table from KEGG was used. Note that these mappings are not defined for NOG terms.

## List of abbreviations

KL divergence: Kullback-Leibler divergence; KO: KEGG Orthology; LDA: Latent Dirichlet Allocation; MCMC: Markov Chain Monte Carlo; OG: Orthologous group (of genes); PF-module: Potential functional module; Verified-PWF-coupling (VPWFC): Verified pairwise functional coupling; Verified-TRF-coupling (VTRFC): Verified (first-order) transitive functional coupling;

## Authors' contributions

SGAK designed the evaluation setup and performed the experiments; LD provided technical advice on Latent Dirichlet Allocation; SGAK and ACM wrote the manuscript; ACM devised the project and gave conceptual advice. All authors read and approved the final manuscript.

## Supplementary Material

Additional file 1 **A list of 198 potential functional modules**. The Supplementary Tables **S1-198 **show 198 potential functional modules that were identified in a randomly chosen, exemplary run of the presented method (*k *= 200). Tables **S1-70 **represent the subset of particularly stable modules that could be tracked consistently across nine independent runs of the method. Click here for file

Additional file 2 **Comparison of histograms over COG functional categories**. Comparison of two histograms over COG functional categories for (***A***) 70 stable modules and (***B***) modules that could not be tracked across all nine runs. Click here for file

Additional file 3 **Profile of KEGG pathways with at least six matches to one of the 198 modules**. Supplementary Table **S1**: List of KEGG pathways with at least six matches of their KO terms to one of the 198 potential functional modules inferred in the exemplary run with *k *= 200. Click here for file

Additional file 4 **Visualized matches to the KEGG pathway 'Porphyrin and chlorophyll metabolism'**. KO terms that are matched by the OGs of the respective potential functional module are highlighted in *pink*. Click here for file

Additional file 5 **'Ribosome'-related functional module**. Supplementary Table **S1**: OGs of the 'Ribosome'-related functional module that was identified in nine runs with *k *= 400. Click here for file

Additional file 6 **Visualized matches to the KEGG pathway 'Ribosome'**. KO terms that are matched by the OGs of the respective potential functional module are highlighted in *pink*. Click here for file

Additional file 7 **Supplementary note**. This document includes additional details of the comparison of results for different settings of *k*, and a discussion on the distribution of the probability weights of the modules across the analyzed genomes. Click here for file

Additional file 8 **Visualization of the functional network spanned by the OG pairs of the reference set**. Pairwise functional interactions are defined by the reference set as edges between OGs in a network graph. The subset of verified pairwise predictions from the modules is shown in *green*, whereas the subset of verified predictions by pairwise co-occurrence profiling is shown in *blue*. Functional interactions that are predicted by both methods are colored *red*, and those not detected by any of the methods are shown in *gray*. Click here for file

Additional file 9 **Visualization of the distribution of probability weights of the modules across the analyzed genomes**. The unclustered heatmap indicates the strengths of probability weights of the 198 modules across the genomes. Rows represent the genomes, whereas columns represent the weights of the modules. The brighter the color of a cell, the larger is the probability weight for the respective PF-module. We re-scaled the values of each row, using minimum and maximum values, to fit values to the interval [0,1]. A discussion of this heatmap is part of the Supplementary note in Additional file 7. Click here for file

Additional file 10 **Reference set of high confidence pairwise OG interactions**. List of high confidence interactions with evidence support values from the individual STRING channels, and modified combined scores. Click here for file
