# Supplementary material for: Inferring functional modules of protein families with probabilistic topic models
Source: BMC Bioinformatics. 2011 May 9;12:141. doi: 10.1186/1471-2105-12-141 (PMC3098182; doi:10.1186/1471-2105-12-141)
Supplement: Additional file 1 — A list of 198 potential functional modules. The Supplementary Tables S1-198 show 198 potential functional modules that were identified in a randomly chosen, exemplary run of the presented method (k = 200). Tables S1-70 represent the subset of particularly stable modules that could be tracked consistently across nine independent runs of the method. [file 1471-2105-12-141-S1.PDF]

List of 198 inferred functional modules

Sebastian Gil Anthony Konietzny

April 28, 2011

This document contains a complete listing of the 198 non-empty potential functional modules (PF-modules) that were inferred with our approach, in an exemplary run with setting k=200. Each module is displayed as a table that lists the OG terms (COG and NOG identifers) satisfying the threshold condition for the respective topic distribution.

COG or NOG terms that are marked with an asterisk following the rank constitute the subset of OGs determining the coverage value of a module.

Tables S1-S70 represent the set of PF-modules that could be tracked across nine independent runs of our method. The remaining tables are taken from an arbitrary run with setting k=200.

We are interested in topic distributions which differ considerably from a non-informative uniform distribution over the vocabulary of OG terms. Therefore, we applied a heuristic ranking method for the topics. For each topic distribution, we computed the Kullback-Leibler divergence with respect to a uniform distribution. The larger the divergence, the higher we estimate the information content of the topics. This way, the functional modules represented by the remaining tables have been ranked according to their estimated information content.

Rank	Probability	OG	Description
1*	0.406	COG0840 [N,T]	Methyl-accepting chemotaxis protein
2*	0.093	COG0784 [T]	FOG: CheY-like receiver
3*	0.055	COG0835 [N,T]	Chemotaxis signal transduction protein
4*	0.053	COG0643 [N,T]	Chemotaxis protein histidine kinase and related kinases
5*	0.032	COG2201 [N,T]	Chemotaxis response regulator containing a CheY-like receiver domain and a methylesterase domain
6*	0.029	COG1352 [N,T]	Methylase of chemotaxis methyl-accepting proteins
7*	0.027	COG0517 [R]	FOG: CBS domain
8*	0.019	COG3706 [T]	Response regulator containing a CheY-like receiver domain and a GGDEF domain
9*	0.019	COG1871 [N,T]	Chemotaxis protein; stimulates methylation of MCP proteins
10*	0.019	COG0491 [R]	Zn-dependent hydrolases, including glyoxylases
11*	0.018	COG1309 [K]	Transcriptional regulator
12*	0.017	COG0399 [M]	Predicted pyridoxal phosphate-dependent enzyme apparently involved in regulation of cell wall biogenesis
13*	0.013	COG0110 [R]	Acetyltransferase (isoleucine patch superfamily)
14*	0.013	COG0367 [E]	Asparagine synthase (glutamine-hydrolyzing)
15*	0.012	COG0451 [M,G]	Nucleoside-diphosphate-sugar epimerases
16*	0.011	COG2378 [K]	Predicted transcriptional regulator

Table S 1: Module 201.

Rank	Probability	OG	Description
1*	0.063	COG1344 [N]	Flagellin and related hook-associated proteins
2*	0.034	COG1360 [N]	Flagellar motor protein
3*	0.030	COG1886 [N,U]	Flagellar motor switch/type III secretory pathway protein
4*	0.029	COG1291 [N]	Flagellar motor component
5*	0.027	COG1157 [N,U]	Flagellar biosynthesis/type III secretory pathway ATPase
6*	0.026	COG4786 [N]	Flagellar basal body rod protein
7*	0.023	COG1749 [N]	Flagellar hook protein FlgE
8*	0.023	COG1345 [N]	Flagellar capping protein
9*	0.023	COG1377 [N,U]	Flagellar biosynthesis pathway, component FlhB
10*	0.023	COG1256 [N]	Flagellar hook-associated protein
11*	0.023	COG0835 [N,T]	Chemotaxis signal transduction protein
12*	0.023	COG1536 [N]	Flagellar motor switch protein
13	0.023	COG0741 [M]	Soluble lytic murein transglycosylase and related regulatory proteins (some contain LysM/invasin domains)
14*	0.022	COG1298 [N,U]	Flagellar biosynthesis pathway, component FlhA
15*	0.022	COG1766 [N,U]	Flagellar biosynthesis/type III secretory pathway lipoprotein
16*	0.022	COG1815 [N]	Flagellar basal body protein
17*	0.022	COG1338 [N,U]	Flagellar biosynthesis pathway, component FliP
18*	0.021	COG1558 [N]	Flagellar basal body rod protein
19*	0.020	COG1684 [N,U]	Flagellar biosynthesis pathway, component FliR
20*	0.020	COG1677 [N,U]	Flagellar hook-basal body protein
21*	0.020	COG1843 [N]	Flagellar hook capping protein
22*	0.020	COG1868 [N]	Flagellar motor switch protein
23*	0.020	COG1516 [N,U,O]	Flagellin-specific chaperone FliS
24*	0.019	COG1317 [N,U]	Flagellar biosynthesis/type III secretory pathway protein
25*	0.019	COG1987 [N,U]	Flagellar biosynthesis pathway, component FliQ
26*	0.018	COG1191 [K]	DNA-directed RNA polymerase specialized sigma subunit
27*	0.017	COG2063 [N]	Flagellar basal body L-ring protein
28*	0.016	COG1706 [N]	Flagellar basal-body P-ring protein
29	0.016	COG0330 [O]	Membrane protease subunits, stomatin/prohibitin homologs
30*	0.014	COG1508 [K]	DNA-directed RNA polymerase specialized sigma subunit, sigma54 homolog
31*	0.013	COG1419 [N]	Flagellar GTP-binding protein
32*	0.013	COG1580 [N]	Flagellar basal body-associated protein
33	0.012	COG1192 [D]	ATPases involved in chromosome partitioning
34*	0.012	COG2201 [N,T]	Chemotaxis response regulator containing a CheY-like receiver domain and a methylesterase domain
35*	0.011	COG1261 [N,O]	Flagellar basal body P-ring biosynthesis protein

Table S 2: Module 202.

Rank	Probability	OG	Description
1*	0.079	COG0500 [Q,R]	SAM-dependent methyltransferases
2*	0.038	COG2243 [H]	Precorrin-2 methylase
3*	0.035	COG1492 [H]	Cobyric acid synthase
4*	0.033	COG2082 [H]	Precorrin isomerase
5*	0.033	COG1270 [H]	Cobalamin biosynthesis protein CobD/CbiB
6*	0.032	COG2241 [H]	Precorrin-6B methylase 1
7*	0.032	COG1010 [H]	Precorrin-3B methylase
8*	0.032	COG2242 [H]	Precorrin-6B methylase 2
9*	0.032	COG2875 [H]	Precorrin-4 methylase
10*	0.031	COG1797 [H]	Cobyrinic acid a,c-diamide synthase
11*	0.028	COG0368 [H]	Cobalamin-5-phosphate synthase
12*	0.027	COG0007 [H]	Uroporphyrinogen-III methylase
13*	0.026	COG2073 [H]	Cobalamin biosynthesis protein CbiG
14*	0.026	COG0079 [E]	Histidinol-phosphate/aromatic aminotransferase and cobyric acid decarboxylase
15*	0.026	COG0115 [E,H]	Branched-chain amino acid aminotransferase/4-amino-4-deoxychorismate lyase
16*	0.025	COG2087 [H]	Adenosyl cobinamide kinase/adenosyl cobinamide phosphate guanylyltransferase
17*	0.024	COG1903 [H]	Cobalamin biosynthesis protein CbiD
18*	0.024	COG2038 [H]	NaMN:DMB phosphoribosyltransferase
19*	0.023	COG2099 [H]	Precorrin-6x reductase
20*	0.023	COG0388 [R]	Predicted amidohydrolase
21*	0.021	COG1249 [C]	Pyruvate/2-oxoglutarate dehydrogenase complex, dihydrolipoamide dehydrogenase (E3) component, and related enzymes
22*	0.019	COG0406 [G]	Fructose-2,6-bisphosphatase
23*	0.018	COG2109 [H]	ATP:corrinoid adenosyltransferase
24*	0.017	COG0456 [R]	Acetyltransferases
25*	0.015	COG0001 [H]	Glutamate-1-semialdehyde aminotransferase
26*	0.014	COG1429 [H]	Cobalamin biosynthesis protein CobN and related Mg-chelatases
27	0.013	COG0226 [P]	ABC-type phosphate transport system, periplasmic component
28	0.013	COG0382 [H]	4-hydroxybenzoate polyprenyltransferase and related prenyltransferases
29*	0.012	COG1587 [H]	Uroporphyrinogen-III synthase
30*	0.012	COG0824 [R]	Predicted thioesterase
31*	0.012	COG0491 [R]	Zn-dependent hydrolases, including glyoxylases
32*	0.011	COG0458 [E,F]	Carbamoylphosphate synthase large subunit (split gene in MJ)

Table S 3: Module 204.

Rank	Probability	OG	Description
1*	0.037	COG1249 [C]	Pyruvate/2-oxoglutarate dehydrogenase complex, dihydrolipoamide dehydrogenase (E3) component, and related enzymes
2*	0.032	COG1894 [C]	NADH:ubiquinone oxidoreductase, NADH-binding (51 kD) subunit
3*	0.031	COG0843 [C]	Heme/copper-type cytochrome/quinol oxidases, subunit 1
4*	0.030	COG1034 [C]	NADH dehydrogenase/NADH:ubiquinone oxidoreductase 75 kD subunit (chain G)
5*	0.028	COG1622 [C]	Heme/copper-type cytochrome/quinol oxidases, subunit 2
6*	0.028	COG1905 [C]	NADH:ubiquinone oxidoreductase 24 kD subunit
7*	0.027	COG0377 [C]	NADH:ubiquinone oxidoreductase 20 kD subunit and related Fe-S oxidoreductases
8*	0.027	COG0838 [C]	NADH:ubiquinone oxidoreductase subunit 3 (chain A)
9*	0.027	COG1008 [C]	NADH:ubiquinone oxidoreductase subunit 4 (chain M)
10*	0.026	COG1009 [C,P]	NADH:ubiquinone oxidoreductase subunit 5 (chain L)/Multisubunit Na+/H+ antiporter, MnhA subunit
11*	0.026	COG1007 [C]	NADH:ubiquinone oxidoreductase subunit 2 (chain N)
12*	0.026	COG1143 [C]	Formate hydrogenlyase subunit 6/NADH:ubiquinone oxidoreductase 23 kD subunit (chain I)
13*	0.026	COG0852 [C]	NADH:ubiquinone oxidoreductase 27 kD subunit
14*	0.026	COG1845 [C]	Heme/copper-type cytochrome/quinol oxidase, subunit 3
15*	0.025	COG0713 [C]	NADH:ubiquinone oxidoreductase subunit 11 or 4L (chain K)
16*	0.025	COG1005 [C]	NADH:ubiquinone oxidoreductase subunit 1 (chain H)
17*	0.025	COG0649 [C]	NADH:ubiquinone oxidoreductase 49 kD subunit 7
18*	0.024	COG1999 [R]	Uncharacterized protein SCO1/SenC/PrrC, involved in biogenesis of respiratory and photosynthetic systems
19*	0.022	COG0839 [C]	NADH:ubiquinone oxidoreductase subunit 6 (chain J)
20*	0.022	COG0320 [H]	Lipoate synthase
21*	0.022	COG0281 [C]	Malic enzyme
22*	0.020	COG0109 [O]	Polyprenyltransferase (cytochrome oxidase assembly factor)
23*	0.019	COG0508 [C]	Pyruvate/2-oxoglutarate dehydrogenase complex, dihydrolipoamide acyltransferase (E2) component, and related enzymes
24*	0.018	COG0590 [F,J]	Cytosine/adenosine deaminases
25*	0.017	COG1612 [O]	Uncharacterized protein required for cytochrome oxidase assembly
26	0.017	COG1565 [S]	Uncharacterized conserved protein
27	0.017	COG1183 [I]	Phosphatidylserine synthase

28*	0.015	COG0624 [E]	Acetylornithine deacetylase/Succinyl-diaminopimelate desuccinylase and related deacylases
29*	0.015	COG0039 [C]	Malate/lactate dehydrogenases
30*	0.015	COG1146 [C]	Ferredoxin
31*	0.014	COG0479 [C]	Succinate dehydrogenase/fumarate reductase, Fe-S protein subunit
32	0.014	COG0009 [J]	Putative translation factor (SUA5)
33*	0.014	COG1048 [C]	Aconitase A
34	0.014	COG1253 [R]	Hemolysins and related proteins containing CBS domains
35	0.013	COG1074 [L]	ATP-dependent exoDNAse (exonuclease V) beta subunit (contains helicase and exonuclease domains)
36	0.013	COG0466 [O]	ATP-dependent Lon protease, bacterial type
37*	0.012	COG0492 [O]	Thioredoxin reductase
38	0.010	COG0568 [K]	DNA-directed RNA polymerase, sigma subunit (sigma70/sigma32)

Table S 4: Module 203.

Rank	Probability	OG	Description
1*	0.331	COG1662 [L]	Transposase and inactivated derivatives, IS1 family
2*	0.156	COG3677 [L]	Transposase and inactivated derivatives
3	0.151	COG2963 [L]	Transposase and inactivated derivatives
4	0.147	COG2801 [L]	Transposase and inactivated derivatives
5	0.042	COG3385 [L]	FOG: Transposase and inactivated derivatives
6	0.016	COG4886 [S]	Leucine-rich repeat (LRR) protein
7	0.013	NOG25595 [L]	Transposase
8	0.011	NOG25351 [S]	Annotation not available

Table S 5: Module 4.

Rank	Probability	OG	Description
1*	0.379	COG0675 [L]	Transposase and inactivated derivatives
2	0.141	COG3385 [L]	FOG: Transposase and inactivated derivatives
3	0.044	COG1192 [D]	ATPases involved in chromosome partitioning
4*	0.039	COG2452 [L]	Predicted site-specific integrase-resolvase
5	0.033	COG3415 [L]	Transposase and inactivated derivatives
6*	0.017	COG1943 [L]	Transposase and inactivated derivatives
7	0.014	COG2897 [P]	Rhodanese-related sulfurtransferase
8	0.014	COG0433 [R]	Predicted ATPase
9	0.012	COG1225 [O]	Peroxiredoxin
10	0.011	COG0464 [O]	ATPases of the AAA+ class
11	0.011	COG1488 [H]	Nicotinic acid phosphoribosyltransferase

Table S 6: Module 6.

Rank	Probability	OG	Description
1*	0.074	COG1028 [I,Q,R]	Dehydrogenases with different specificities (related to short-chain alcohol dehydrogenases)
2*	0.064	COG2202 [T]	FOG: PAS/PAC domain
3*	0.043	COG0784 [T]	FOG: CheY-like receiver
4*	0.039	COG0477 [G,E,P,R]	Permeases of the major facilitator superfamily
5*	0.030	COG2710 [C]	Nitrogenase molybdenum-iron protein, alpha and beta chains
6*	0.029	COG0583 [K]	Transcriptional regulator
7*	0.026	COG5001 [T]	Predicted signal transduction protein containing a membrane domain, an EAL and a GGDEF domain
8*	0.022	COG1309 [K]	Transcriptional regulator
9*	0.019	COG0318 [I,Q]	Acyl-CoA synthetases (AMP-forming)/AMP-acid ligases II
10	0.015	COG1305 [E]	Transglutaminase-like enzymes, putative cysteine proteases
11	0.013	COG1233 [Q]	Phytoene dehydrogenase and related proteins
12*	0.012	COG0535 [R]	Predicted Fe-S oxidoreductases
13*	0.010	COG0604 [C,R]	NADPH:quinone reductase and related Zn-dependent oxidoreductases
14*	0.010	COG0697 [G,E,R]	Permeases of the drug/metabolite transporter (DMT) superfamily
15	0.010	COG1850 [G]	Ribulose 1,5-bisphosphate carboxylase, large subunit

Table S 7: Module 8.

Rank	Probability	OG	Description
1	0.076	COG0582 [L]	Integrase
2	0.048	COG4644 [L]	Transposase and inactivated derivatives, TnpA family
3	0.023	COG0550 [L]	Topoisomerase IA
4	0.021	COG0741 [M]	Soluble lytic murein transglycosylase and related regulatory proteins (some contain LysM/invasin domains)
5*	0.020	NOG10760 [S]	Annotation not available
6	0.018	COG2003 [L]	DNA repair proteins
7	0.017	NOG04077 [S]	Helicase
8	0.016	COG1651 [O]	Protein-disulfide isomerase
9*	0.016	NOG04080 [S]	Annotation not available
10*	0.016	NOG25647 [S]	Type IV secretory protein VirB4
11*	0.016	NOG04079 [S]	Annotation not available
12	0.014	COG1192 [D]	ATPases involved in chromosome partitioning
13*	0.014	NOG04076 [S]	Annotation not available
14*	0.014	NOG11305 [S]	Annotation not available
15*	0.014	NOG11941 [S]	Annotation not available
16*	0.014	NOG11996 [S]	Annotation not available
17	0.014	COG0210 [L]	Superfamily I DNA and RNA helicases
18	0.014	COG0625 [O]	Glutathione S-transferase
19	0.013	NOG05921 [S]	Annotation not available
20*	0.013	NOG07322 [S]	Annotation not available
21*	0.013	NOG12842 [S]	Annotation not available
22	0.012	COG0389 [L]	Nucleotidyltransferase/DNA polymerase involved in DNA repair
23	0.012	NOG07320 [S]	Annotation not available
24*	0.012	NOG13741 [S]	Annotation not available
25	0.011	NOG06516 [S]	Annotation not available
26*	0.011	NOG43706 [S]	Annotation not available
27	0.011	COG0789 [K]	Predicted transcriptional regulators
28	0.011	NOG07319 [S]	Annotation not available
29	0.011	COG0270 [L]	Site-specific DNA methylase
30	0.011	COG0629 [L]	Single-stranded DNA-binding protein
31*	0.011	NOG71004 [S]	Annotation not available
32*	0.011	NOG48157 [S]	Annotation not available
33*	0.010	NOG40693 [S]	Annotation not available

Table S 8: Module 10.

Rank	Probability	OG	Description
1*	0.189	COG2199 [T]	FOG: GGDEF domain
2*	0.128	COG2200 [T]	FOG: EAL domain
3	0.059	COG0582 [L]	Integrase
4*	0.059	COG0438 [M]	Glycosyltransferase
5*	0.055	COG0500 [Q,R]	SAM-dependent methyltransferases
6*	0.053	COG0745 [T,K]	Response regulators consisting of a CheY-like receiver domain and a winged-helix DNA-binding domain
7*	0.049	COG0494 [L,R]	NTP pyrophosphohydrolases including oxidative damage repair enzymes
8*	0.038	COG2207 [K]	AraC-type DNA-binding domain-containing proteins
9*	0.038	COG0834 [E,T]	ABC-type amino acid transport/signal transduction systems, periplasmic component/domain
10*	0.034	COG2202 [T]	FOG: PAS/PAC domain
11	0.024	COG0668 [M]	Small-conductance mechanosensitive channel
12	0.020	COG1396 [K]	Predicted transcriptional regulators
13*	0.018	COG0697 [G,E,R]	Permeases of the drug/metabolite transporter (DMT) superfamily
14	0.016	COG0563 [F]	Adenylate kinase and related kinases
15	0.015	COG1192 [D]	ATPases involved in chromosome partitioning
16*	0.013	COG2770 [T]	FOG: HAMP domain
17	0.010	COG0070 [E]	Glutamate synthase domain 3

Table S 9: Module 11.

Rank	Probability	OG	Description
1*	0.647	COG2801 [L]	Transposase and inactivated derivatives
2*	0.248	COG2963 [L]	Transposase and inactivated derivatives
3	0.017	COG0477 [G,E,P,R]	Permeases of the major facilitator superfamily

Table S 10: Module 13.

Rank	Probability	OG	Description
1*	0.264	COG0834 [E,T]	ABC-type amino acid transport/signal transduction systems, periplasmic component/domain
2*	0.191	COG0765 [E]	ABC-type amino acid transport system, permease component
3*	0.121	COG1126 [E]	ABC-type polar amino acid transport system, ATPase component
4*	0.039	COG0624 [E]	Acetylornithine deacetylase/Succinyl-diaminopimelate desuccinylase and related deacylases
5*	0.036	COG0436 [E]	Aspartate/tyrosine/aromatic aminotransferase
6	0.033	COG1108 [P]	ABC-type Mn2+/Zn2+ transport systems, permease components
7*	0.027	COG0110 [R]	Acetyltransferase (isoleucine patch superfamily)
8	0.026	COG1121 [P]	ABC-type Mn/Zn transport systems, ATPase component
9*	0.022	COG0318 [I,Q]	Acyl-CoA synthetases (AMP-forming)/AMP-acid ligases II
10	0.022	COG0803 [P]	ABC-type metal ion transport system, periplasmic component/surface adhesin
11	0.020	COG0053 [P]	Predicted Co/Zn/Cd cation transporters
12*	0.020	COG0008 [J]	Glutamyl- and glutaminyl-tRNA synthetases
13*	0.019	COG0251 [J]	Putative translation initiation inhibitor, yjgF family
14*	0.015	COG0456 [R]	Acetyltransferases
15*	0.011	COG0078 [E]	Ornithine carbamoyltransferase
16*	0.011	COG0548 [E]	Acetylglutamate kinase

Table S 11: Module 16.

Rank	Probability	OG	Description
1*	0.029	COG1595 [K]	DNA-directed RNA polymerase specialized sigma subunit, sigma24 homolog
2*	0.029	COG1309 [K]	Transcriptional regulator
3*	0.023	COG1020 [Q]	Non-ribosomal peptide synthetase modules and related proteins
4*	0.022	COG0318 [I,Q]	Acyl-CoA synthetases (AMP-forming)/AMP-acid ligases II
5*	0.020	COG2072 [P]	Predicted flavoprotein involved in K+ transport
6*	0.019	COG0515 [R,T,K,L]	Serine/threonine protein kinase
7*	0.018	COG0627 [R]	Predicted esterase
8*	0.017	COG0491 [R]	Zn-dependent hydrolases, including glyoxylases
9*	0.017	COG0642 [T]	Signal transduction histidine kinase
10*	0.016	COG0526 [O,C]	Thiol-disulfide isomerase and thioredoxins
11*	0.013	COG2141 [C]	Coenzyme F420-dependent N5,N10-methylene tetrahydromethanopterin reductase and related flavin-dependent oxidoreductases
12*	0.011	COG1396 [K]	Predicted transcriptional regulators
13*	0.011	COG0607 [P]	Rhodanese-related sulfurtransferase
14*	0.011	COG2217 [P]	Cation transport ATPase
15*	0.011	COG0657 [I]	Esterase/lipase
16*	0.010	COG1716 [T]	FOG: FHA domain
17*	0.010	COG4799 [I]	Acetyl-CoA carboxylase, carboxyltransferase component (subunits alpha and beta)

Table S 12: Module 17.

Rank	Probability	OG	Description
1*	0.057	COG1309 [K]	Transcriptional regulator
2*	0.043	COG0515 [R,T,K,L]	Serine/threonine protein kinase
3*	0.040	COG1595 [K]	DNA-directed RNA polymerase specialized sigma subunit, sigma24 homolog
4*	0.034	COG1131 [V]	ABC-type multidrug transport system, ATPase component
5*	0.029	COG2197 [T,K]	Response regulator containing a CheY-like receiver domain and an HTH DNA-binding domain
6*	0.028	COG2141 [C]	Coenzyme F420-dependent N5,N10-methylene tetrahydromethanopterin reductase and related flavin-dependent oxidoreductases
7*	0.024	COG2208 [T,K]	Serine phosphatase RsbU, regulator of sigma subunit
8*	0.022	COG2124 [Q]	Cytochrome P450
9*	0.022	COG0642 [T]	Signal transduction histidine kinase
10*	0.020	COG1132 [V]	ABC-type multidrug transport system, ATPase and permease components
11*	0.019	COG0667 [C]	Predicted oxidoreductases (related to aryl-alcohol dehydrogenases)
12*	0.017	COG0702 [M,G]	Predicted nucleoside-diphosphate-sugar epimerases
13*	0.016	COG0654 [H,C]	2-polyprenyl-6-methoxyphenol hydroxylase and related FAD-dependent oxidoreductases
14*	0.014	COG4585 [T]	Signal transduction histidine kinase
15*	0.014	COG2378 [K]	Predicted transcriptional regulator
16*	0.013	COG1960 [I]	Acyl-CoA dehydrogenases
17*	0.012	COG0789 [K]	Predicted transcriptional regulators
18	0.012	COG3629 [T]	DNA-binding transcriptional activator of the SARP family
19	0.012	COG2018 [R]	Uncharacterized distant relative of homeotic protein bithoraxoid
20	0.011	COG1670 [J]	Acetyltransferases, including N-acetylases of ribosomal proteins
21*	0.010	COG1191 [K]	DNA-directed RNA polymerase specialized sigma subunit
22*	0.010	COG0842 [V]	ABC-type multidrug transport system, permease component

Table S 13: Module 20.

Rank	Probability	OG	Description
1*	0.040	COG0028 [E,H]	Thiamine pyrophosphate-requiring enzymes [acetolactate synthase, pyruvate dehydrogenase (cytochrome), glyoxylate carboligase, phosphonopyruvate decarboxylase]
2*	0.037	COG0526 [O,C]	Thiol-disulfide isomerase and thioredoxins
3*	0.031	COG0119 [E]	Isopropylmalate/homocitrate/citramalate synthases
4*	0.022	COG0079 [E]	Histidinol-phosphate/aromatic aminotransferase and cobyric acid decarboxylase
5*	0.021	COG0066 [E]	3-isopropylmalate dehydratase small subunit
6*	0.019	COG0069 [E]	Glutamate synthase domain 2
7*	0.019	COG0547 [E]	Anthranilate phosphoribosyltransferase
8*	0.018	COG0159 [E]	Tryptophan synthase alpha chain
9*	0.018	COG0620 [E]	Methionine synthase II (cobalamin-independent)
10*	0.018	COG0460 [E]	Homoserine dehydrogenase
11*	0.018	COG0019 [E]	Diaminopimelate decarboxylase
12*	0.018	COG0067 [E]	Glutamate synthase domain 1
13*	0.018	COG0135 [E]	Phosphoribosylanthranilate isomerase
14*	0.017	COG0059 [E,H]	Ketol-acid reductoisomerase
15*	0.017	COG0512 [E,H]	Anthranilate/para-aminobenzoate synthases component II
16*	0.017	COG0147 [E,H]	Anthranilate/para-aminobenzoate synthases component I
17*	0.017	COG0070 [E]	Glutamate synthase domain 3
18*	0.017	COG0106 [E]	Phosphoribosylformimino-5-aminoimidazole carboxamide ribonucleotide (ProFAR) isomerase
19*	0.017	COG0548 [E]	Acetylglutamate kinase
20*	0.017	COG0139 [E]	Phosphoribosyl-AMP cyclohydrolase
21*	0.016	COG0107 [E]	Imidazoleglycerol-phosphate synthase
22*	0.016	COG0002 [E]	Acetylglutamate semialdehyde dehydrogenase
23*	0.016	COG0165 [E]	Argininosuccinate lyase
24*	0.016	COG0065 [E]	3-isopropylmalate dehydratase large subunit
25*	0.016	COG0118 [E]	Glutamine amidotransferase
26*	0.016	COG0137 [E]	Argininosuccinate synthase
27*	0.016	COG0082 [E]	Chorismate synthase
28*	0.016	COG0134 [E]	Indole-3-glycerol phosphate synthase
29*	0.015	COG0131 [E]	Imidazoleglycerol-phosphate dehydratase

30*	0.015	COG0287 [E]	Prephenate dehydrogenase
31*	0.015	COG4992 [E]	Ornithine/acetylornithine aminotransferase
32*	0.015	COG0040 [E]	ATP phosphoribosyltransferase
33*	0.015	COG0077 [E]	Prephenate dehydratase
34	0.014	COG0004 [P]	Ammonia permease
35*	0.014	COG0436 [E]	Aspartate/tyrosine/aromatic aminotransferase
36*	0.014	COG0129 [E,G]	Dihydroxyacid dehydratase/phosphogluconate dehydratase
37*	0.014	COG0458 [E,F]	Carbamoylphosphate synthase large subunit (split gene in MJ)
38	0.014	COG0573 [P]	ABC-type phosphate transport system, permease component
39*	0.013	COG0141 [E]	Histidinol dehydrogenase
40*	0.013	COG0140 [E]	Phosphoribosyl-ATP pyrophosphohydrolase
41*	0.013	COG0440 [E]	Acetolactate synthase, small (regulatory) subunit
42*	0.013	COG0527 [E]	Aspartokinases
43	0.012	COG1117 [P]	ABC-type phosphate transport system, ATPase component
44*	0.012	COG0057 [G]	Glyceraldehyde-3-phosphate dehydrogenase/erythrose-4-phosphate dehydrogenase
45*	0.011	COG0133 [E]	Tryptophan synthase beta chain
46*	0.011	COG0425 [O]	Predicted redox protein, regulator of disulfide bond formation
47	0.011	COG0239 [D]	Integral membrane protein possibly involved in chromosome condensation
48*	0.011	COG1605 [E]	Chorismate mutase
49*	0.010	COG1364 [E]	N-acetylglutamate synthase (N-acetylornithine aminotransferase)
50	0.010	COG0602 [O]	Organic radical activating enzymes

Table S 14: Module 21.

Rank	Probability	OG	Description
1*	0.066	COG0477 [G,E,P,R]	Permeases of the major facilitator superfamily
2*	0.027	COG1609 [K]	Transcriptional regulators
3*	0.018	COG0673 [R]	Predicted dehydrogenases and related proteins
4	0.013	COG1253 [R]	Hemolysins and related proteins containing CBS domains
5*	0.012	COG0438 [M]	Glycosyltransferase
6*	0.012	COG1349 [K,G]	Transcriptional regulators of sugar metabolism
7	0.012	COG0262 [H]	Dihydrofolate reductase
8	0.011	COG1113 [E]	Gamma-aminobutyrate permease and related permeases

Table S 15: Module 23.

Rank	Probability	OG	Description
1*	0.132	COG5651 [N]	PPE-repeat proteins
2*	0.045	COG0500 [Q,R]	SAM-dependent methyltransferases
3*	0.035	COG0596 [R]	Predicted hydrolases or acyltransferases (alpha/beta hydrolase superfamily)
4*	0.031	COG0477 [G,E,P,R]	Permeases of the major facilitator superfamily
5	0.025	COG1848 [R]	Predicted nucleic acid-binding protein, contains PIN domain
6*	0.019	COG3321 [Q]	Polyketide synthase modules and related proteins
7*	0.018	COG0277 [C]	FAD/FMN-containing dehydrogenases
8	0.018	COG1487 [R]	Predicted nucleic acid-binding protein, contains PIN domain
9*	0.016	COG0451 [M,G]	Nucleoside-diphosphate-sugar epimerases
10*	0.015	COG2114 [T]	Adenylate cyclase, family 3 (some proteins contain HAMP domain)
11*	0.014	COG3315 [Q]	O-Methyltransferase involved in polyketide biosynthesis
12*	0.013	COG0657 [I]	Esterase/lipase
13*	0.011	COG2230 [M]	Cyclopropane fatty acid synthase and related methyltransferases
14*	0.011	COG3328 [L]	Transposase and inactivated derivatives
15*	0.010	COG0664 [T]	cAMP-binding proteins - catabolite gene activator and regulatory subunit of cAMP-dependent protein kinases
16	0.010	COG2337 [T]	Growth inhibitor

Table S 16: Module 24.

Rank	Probability	OG	Description
1*	0.054	COG0642 [T]	Signal transduction histidine kinase
2*	0.044	COG0784 [T]	FOG: CheY-like receiver
3*	0.043	COG2207 [K]	AraC-type DNA-binding domain-containing proteins
4*	0.029	COG0500 [Q,R]	SAM-dependent methyltransferases
5*	0.026	COG0834 [E,T]	ABC-type amino acid transport/signal transduction systems, periplasmic component/domain
6*	0.017	COG0583 [K]	Transcriptional regulator
7*	0.015	COG0789 [K]	Predicted transcriptional regulators
8*	0.013	COG2204 [T]	Response regulator containing CheY-like receiver, AAA-type ATPase, and DNA-binding domains
9*	0.011	COG0388 [R]	Predicted amidohydrolase
10*	0.010	COG2197 [T,K]	Response regulator containing a CheY-like receiver domain and an HTH DNA-binding domain

Table S 17: Module 28.

Rank	Probability	OG	Description
1	0.500	COG3436 [L]	Transposase and inactivated derivatives
2	0.087	COG3344 [L]	Retron-type reverse transcriptase
3	0.037	COG3385 [L]	FOG: Transposase and inactivated derivatives
4	0.031	COG1192 [D]	ATPases involved in chromosome partitioning
5	0.030	NOG26112 [L]	Transposase
6	0.020	COG2963 [L]	Transposase and inactivated derivatives
7	0.019	NOG04436 [L]	Transposase
8	0.018	NOG44700 [L]	Transposase
9	0.017	NOG40905 [L]	Transposase
10	0.015	NOG25595 [L]	Transposase
11	0.013	COG1112 [L]	Superfamily I DNA and RNA helicases and helicase subunits
12	0.013	COG0389 [L]	Nucleotidyltransferase/DNA polymerase involved in DNA repair
13	0.011	NOG45227 [L]	Transposase

Table S 18: Module 29.

Rank	Probability	OG	Description
1	0.646	COG3547 [L]	Transposase and inactivated derivatives
2	0.037	COG0265 [O]	Trypsin-like serine proteases, typically periplasmic, contain C-terminal PDZ domain
3	0.032	COG1475 [K]	Predicted transcriptional regulators
4	0.026	COG1136 [V]	ABC-type antimicrobial peptide transport system, ATPase component
5	0.025	COG1846 [K]	Transcriptional regulators
6	0.014	COG2337 [T]	Growth inhibitor
7	0.013	COG1249 [C]	Pyruvate/2-oxoglutarate dehydrogenase complex, dihydrolipoamide dehydrogenase (E3) component, and related enzymes
8	0.012	COG0663 [R]	Carbonic anhydrases/acetyltransferases, isoleucine patch superfamily
9	0.012	COG0406 [G]	Fructose-2,6-bisphosphatase

Table S 19: Module 30.

Rank	Probability	OG	Description
1*	0.099	COG1172 [G]	Ribose/xylose/arabinose/galactoside ABC-type transport systems, permease components
2*	0.098	COG1129 [G]	ABC-type sugar transport system, ATPase component
3*	0.098	COG1879 [G]	ABC-type sugar transport system, periplasmic component
4*	0.060	COG1609 [K]	Transcriptional regulators
5*	0.050	COG0524 [G]	Sugar kinases, ribokinase family
6*	0.027	COG1082 [G]	Sugar phosphate isomerases/epimerases
7*	0.027	COG1070 [G]	Sugar (pentulose and hexulose) kinases
8*	0.025	COG2186 [K]	Transcriptional regulators
9*	0.024	COG0673 [R]	Predicted dehydrogenases and related proteins
10*	0.022	COG1737 [K]	Transcriptional regulators
11*	0.019	COG1349 [K,G]	Transcriptional regulators of sugar metabolism
12*	0.018	COG2390 [K]	Transcriptional regulator, contains sigma factor-related N-terminal domain
13*	0.017	COG0765 [E]	ABC-type amino acid transport system, permease component
14*	0.015	COG1063 [E,R]	Threonine dehydrogenase and related Zn-dependent dehydrogenases
15*	0.015	COG1126 [E]	ABC-type polar amino acid transport system, ATPase component
16*	0.015	COG2376 [G]	Dihydroxyacetone kinase
17	0.015	COG0600 [P]	ABC-type nitrate/sulfonate/bicarbonate transport system, permease component
18	0.013	COG1177 [E]	ABC-type spermidine/putrescine transport system, permease component II
19*	0.013	COG4213 [G]	ABC-type xylose transport system, periplasmic component
20*	0.012	COG3839 [G]	ABC-type sugar transport systems, ATPase components
21*	0.012	COG4948 [M,R]	L-alanine-DL-glutamate epimerase and related enzymes of enolase superfamily
22*	0.011	COG4214 [G]	ABC-type xylose transport system, permease component
23*	0.011	COG0246 [G]	Mannitol-1-phosphate/altronate dehydrogenases
24	0.011	COG0402 [F,R]	Cytosine deaminase and related metal-dependent hydrolases
25*	0.010	COG0800 [G]	2-keto-3-deoxy-6-phosphogluconate aldolase

Table S 20: Module 38.

Rank	Probability	OG	Description
1	0.077	COG3291 [R]	FOG: PKD repeat
2*	0.047	COG2202 [T]	FOG: PAS/PAC domain
3*	0.043	COG0457 [R]	FOG: TPR repeat
4*	0.034	COG0500 [Q,R]	SAM-dependent methyltransferases
5*	0.032	COG3920 [T]	Signal transduction histidine kinase
6	0.019	COG5421 [L]	Transposase
7	0.017	COG4742 [K]	Predicted transcriptional regulator
8	0.016	COG0655 [R]	Multimeric flavodoxin WrbA
9	0.015	COG3391 [S]	Uncharacterized conserved protein
10	0.014	COG1520 [S]	FOG: WD40-like repeat
11	0.013	COG1413 [C]	FOG: HEAT repeat
12	0.012	COG5012 [R]	Predicted cobalamin binding protein
13	0.011	COG1122 [P]	ABC-type cobalt transport system, ATPase component
14*	0.011	COG1846 [K]	Transcriptional regulators
15	0.010	COG0145 [E,Q]	N-methylhydantoinase A/acetone carboxylase, beta subunit

Table S 21: Module 41.

Rank	Probability	OG	Description
1	0.013	COG0840 [N,T]	Methyl-accepting chemotaxis protein

Table S 22: Module 45.

Rank	Probability	OG	Description
1*	0.029	COG0845 [M]	Membrane-fusion protein
2*	0.024	COG2199 [T]	FOG: GGDEF domain
3*	0.020	COG0477 [G,E,P,R]	Permeases of the major facilitator superfamily
4*	0.019	COG0697 [G,E,R]	Permeases of the drug/metabolite transporter (DMT) superfamily
5*	0.018	COG0841 [V]	Cation/multidrug efflux pump
6*	0.017	COG0642 [T]	Signal transduction histidine kinase
7*	0.015	COG0513 [L,K,J]	Superfamily II DNA and RNA helicases
8*	0.013	COG1670 [J]	Acetyltransferases, including N-acetylases of ribosomal proteins
9	0.012	COG1757 [C]	Na+/H+ antiporter
10*	0.011	COG1846 [K]	Transcriptional regulators

Table S 23: Module 49.

Rank	Probability	OG	Description
1*	0.171	COG0747 [E]	ABC-type dipeptide transport system, periplasmic component
2*	0.160	COG0601 [E,P]	ABC-type dipeptide/oligopeptide/nickel transport systems, permease components
3*	0.148	COG1173 [E,P]	ABC-type dipeptide/oligopeptide/nickel transport systems, permease components
4*	0.117	COG4608 [E]	ABC-type oligopeptide transport system, ATPase component
5*	0.113	COG0444 [E,P]	ABC-type dipeptide/oligopeptide/nickel transport system, ATPase component
6*	0.035	COG0454 [K,R]	Histone acetyltransferase HPA2 and related acetyltransferases
7	0.033	COG1132 [V]	ABC-type multidrug transport system, ATPase and permease components
8	0.028	COG1473 [R]	Metal-dependent amidase/aminoacylase/carboxypeptidase
9*	0.026	COG1136 [V]	ABC-type antimicrobial peptide transport system, ATPase component
10*	0.022	COG0577 [V]	ABC-type antimicrobial peptide transport system, permease component
11*	0.018	COG0518 [F]	GMP synthase - Glutamine amidotransferase domain

Table S 24: Module 53.

Rank	Probability	OG	Description
1*	0.027	COG0625 [O]	Glutathione S-transferase
2	0.024	COG3637 [M]	Opacity protein and related surface antigens
3	0.021	COG1376 [S]	Uncharacterized protein conserved in bacteria
4*	0.020	COG0596 [R]	Predicted hydrolases or acyltransferases (alpha/beta hydrolase superfamily)
5*	0.018	COG0664 [T]	cAMP-binding proteins - catabolite gene activator and regulatory subunit of cAMP-dependent protein kinases
6*	0.017	COG2114 [T]	Adenylate cyclase, family 3 (some proteins contain HAMP domain)
7	0.017	COG0845 [M]	Membrane-fusion protein
8	0.016	COG0841 [V]	Cation/multidrug efflux pump
9	0.015	COG2207 [K]	AraC-type DNA-binding domain-containing proteins
10	0.015	COG1846 [K]	Transcriptional regulators
11	0.015	COG4249 [R]	Uncharacterized protein containing caspase domain
12*	0.011	COG1680 [V]	Beta-lactamase class C and other penicillin binding proteins

Table S 25: Module 62.

Rank	Probability	OG	Description
1*	0.271	COG0642 [T]	Signal transduction histidine kinase
2*	0.264	COG2202 [T]	FOG: PAS/PAC domain
3*	0.190	COG0784 [T]	FOG: CheY-like receiver
4*	0.053	COG2203 [T]	FOG: GAF domain
5*	0.019	COG1136 [V]	ABC-type antimicrobial peptide transport system, ATPase component
6*	0.012	COG0451 [M,G]	Nucleoside-diphosphate-sugar epimerases

Table S 26: Module 63.

Rank	Probability	OG	Description
1	0.023	COG1538 [M,U]	Outer membrane protein
2*	0.021	COG0702 [M,G]	Predicted nucleoside-diphosphate-sugar epimerases
3*	0.021	COG1032 [C]	Fe-S oxidoreductase
4*	0.020	COG0451 [M,G]	Nucleoside-diphosphate-sugar epimerases
5	0.019	COG0003 [P]	Oxyanion-translocating ATPase
6*	0.019	COG0446 [R]	Uncharacterized NAD(FAD)-dependent dehydrogenases
7*	0.019	COG1028 [I,Q,R]	Dehydrogenases with different specificities (related to short-chain alcohol dehydrogenases)
8	0.015	COG0366 [G]	Glycosidases
9*	0.015	COG0543 [H,C]	2-polyprenylphenol hydroxylase and related flavodoxin oxidoreductases
10	0.013	COG1357 [S]	Uncharacterized low-complexity proteins
11*	0.012	COG0074 [C]	Succinyl-CoA synthetase, alpha subunit
12	0.011	COG0546 [R]	Predicted phosphatases

Table S 27: Module 65.

Rank	Probability	OG	Description
1*	0.068	COG3501 [S]	Uncharacterized protein conserved in bacteria
2*	0.041	COG2197 [T,K]	Response regulator containing a CheY-like receiver domain and an HTH DNA-binding domain
3*	0.039	COG0438 [M]	Glycosyltransferase
4*	0.034	COG0542 [O]	ATPases with chaperone activity, ATP-binding subunit
5*	0.029	COG3209 [M]	Rhs family protein
6*	0.028	COG3515 [S]	Uncharacterized protein conserved in bacteria
7*	0.027	COG3523 [S]	Uncharacterized protein conserved in bacteria
8*	0.026	COG3519 [S]	Uncharacterized protein conserved in bacteria
9*	0.024	COG3517 [S]	Uncharacterized protein conserved in bacteria
10*	0.023	COG4104 [S]	Uncharacterized conserved protein
11*	0.023	COG3522 [S]	Uncharacterized protein conserved in bacteria
12*	0.022	COG3516 [S]	Uncharacterized protein conserved in bacteria
13*	0.022	COG3455 [S]	Uncharacterized protein conserved in bacteria
14*	0.022	COG3157 [S]	Hemolysin-coregulated protein (uncharacterized)
15*	0.021	COG3520 [S]	Uncharacterized protein conserved in bacteria
16*	0.019	COG3518 [S]	Uncharacterized protein conserved in bacteria
17*	0.018	COG0318 [I,Q]	Acyl-CoA synthetases (AMP-forming)/AMP-acid ligases II
18*	0.016	COG2771 [K]	DNA-binding HTH domain-containing proteins
19*	0.015	COG0596 [R]	Predicted hydrolases or acyltransferases (alpha/beta hydrolase superfamily)
20	0.014	COG1450 [N,U]	Type II secretory pathway, component PulD
21*	0.014	COG3521 [S]	Uncharacterized protein conserved in bacteria
22*	0.014	COG0332 [I]	3-oxoacyl-[acyl-carrier-protein] synthase III
23*	0.013	COG1024 [I]	Enoyl-CoA hydratase/carnithine racemase
24	0.013	COG0586 [S]	Uncharacterized membrane-associated protein
25	0.013	COG1858 [P]	Cytochrome c peroxidase
26*	0.013	COG0446 [R]	Uncharacterized NAD(FAD)-dependent dehydrogenases
27*	0.013	COG1360 [N]	Flagellar motor protein
28*	0.012	COG3456 [T]	Uncharacterized conserved protein, contains FHA domain
29	0.010	COG1502 [I]	Phosphatidylserine/phosphatidylglycerophosphate/cardiolipin synthases and related enzymes

Table S 28: Module 68.

Rank	Probability	OG	Description
1	0.017	COG0778 [C]	Nitroreductase
2	0.015	COG0566 [J]	rRNA methylases
3	0.015	COG0793 [M]	Periplasmic protease
4	0.015	COG1373 [R]	Predicted ATPase (AAA+ superfamily)
5	0.011	COG1596 [M]	Periplasmic protein involved in polysaccharide export
6	0.011	NOG44579 [S]	Annotation not available
7	0.011	COG0577 [V]	ABC-type antimicrobial peptide transport system, permease component

Table S 29: Module 70.

Rank	Probability	OG	Description
1*	0.193	COG0438 [M]	Glycosyltransferase
2*	0.123	COG0463 [M]	Glycosyltransferases involved in cell wall biogenesis
3*	0.058	COG0451 [M,G]	Nucleoside-diphosphate-sugar epimerases
4*	0.044	COG1216 [R]	Predicted glycosyltransferases
5*	0.033	COG0673 [R]	Predicted dehydrogenases and related proteins
6*	0.031	COG0110 [R]	Acetyltransferase (isoleucine patch superfamily)
7*	0.029	COG2244 [R]	Membrane protein involved in the export of O-antigen and teichoic acid
8*	0.028	COG0399 [M]	Predicted pyridoxal phosphate-dependent enzyme apparently involved in regulation of cell wall biogenesis
9*	0.024	COG0726 [G]	Predicted xylanase/chitin deacetylase
10*	0.024	COG2148 [M]	Sugar transferases involved in lipopolysaccharide synthesis
11*	0.024	COG0489 [D]	ATPases involved in chromosome partitioning
12*	0.023	COG1898 [M]	dTDP-4-dehydrorhamnose 3,5-epimerase and related enzymes
13*	0.018	COG0367 [E]	Asparagine synthase (glutamine-hydrolyzing)
14*	0.017	COG0472 [M]	UDP-N-acetylmuramyl pentapeptide phosphotransferase/UDP-N-acetylglucosamine-1-phosphate transferase
15*	0.016	COG0028 [E,H]	Thiamine pyrophosphate-requiring enzymes [acetolactate synthase, pyruvate dehydrogenase (cytochrome), glyoxylate carboligase, phosphonopyruvate decarboxylase]
16*	0.015	COG0693 [R]	Putative intracellular protease/amidase
17*	0.015	COG0247 [C]	Fe-S oxidoreductase
18*	0.014	COG1091 [M]	dTDP-4-dehydrorhamnose reductase
19	0.014	COG0226 [P]	ABC-type phosphate transport system, periplasmic component
20	0.012	COG0535 [R]	Predicted Fe-S oxidoreductases
21	0.012	COG1408 [R]	Predicted phosphohydrolases
22*	0.012	COG1209 [M]	dTDP-glucose pyrophosphorylase
23*	0.011	COG1082 [G]	Sugar phosphate isomerases/epimerases
24*	0.011	COG1045 [E]	Serine acetyltransferase
25*	0.010	COG0071 [O]	Molecular chaperone (small heat shock protein)
26	0.010	COG0628 [R]	Predicted permease
27*	0.010	COG1208 [M,J]	Nucleoside-diphosphate-sugar pyrophosphorylase involved in lipopolysaccharide biosynthesis/translation initiation factor 2B, gamma/epsilon subunits (eIF-2Bgamma/eIF-2Bepsilon)

Table S 30: Module 74.

Rank	Probability	OG	Description
1	0.018	COG1846 [K]	Transcriptional regulators
2	0.015	COG1674 [D]	DNA segregation ATPase FtsK/SpoIIIE and related proteins
3	0.014	COG2508 [T,Q]	Regulator of polyketide synthase expression
4	0.011	COG0508 [C]	Pyruvate/2-oxoglutarate dehydrogenase complex, dihydrolipoamide acyltransferase (E2) component, and related enzymes
5	0.011	COG1316 [K]	Transcriptional regulator

Table S 31: Module 76.

Rank	Probability	OG	Description
1*	0.025	COG0845 [M]	Membrane-fusion protein
2*	0.024	COG0642 [T]	Signal transduction histidine kinase
3*	0.024	COG0500 [Q,R]	SAM-dependent methyltransferases
4*	0.021	COG0596 [R]	Predicted hydrolases or acyltransferases (alpha/beta hydrolase superfamily)
5*	0.021	COG0583 [K]	Transcriptional regulator
6*	0.018	COG1538 [M,U]	Outer membrane protein
7*	0.016	COG1752 [R]	Predicted esterase of the alpha-beta hydrolase superfamily
8*	0.015	COG1028 [I,Q,R]	Dehydrogenases with different specificities (related to short-chain alcohol dehydrogenases)
9	0.015	COG0790 [R]	FOG: TPR repeat, SEL1 subfamily
10*	0.014	COG5001 [T]	Predicted signal transduction protein containing a membrane domain, an EAL and a GGDEF domain
11*	0.011	COG0834 [E,T]	ABC-type amino acid transport/signal transduction systems, periplasmic component/domain
12	0.011	COG1373 [R]	Predicted ATPase (AAA+ superfamily)
13*	0.011	COG0624 [E]	Acetylornithine deacetylase/Succinyl-diaminopimelate desuccinylase and related deacylases
14*	0.010	COG0666 [R]	FOG: Ankyrin repeat

Table S 32: Module 81.

Rank	Probability	OG	Description
1*	0.018	COG0642 [T]	Signal transduction histidine kinase
2*	0.014	COG1396 [K]	Predicted transcriptional regulators
3*	0.014	COG3920 [T]	Signal transduction histidine kinase
4*	0.011	COG2204 [T]	Response regulator containing CheY-like receiver, AAA-type ATPase, and DNA-binding domains

Table S 33: Module 82.

Rank	Probability	OG	Description
1*	0.030	COG2206 [T]	HD-GYP domain
2	0.017	COG4974 [L]	Site-specific recombinase XerD
3	0.016	COG1905 [C]	NADH:ubiquinone oxidoreductase 24 kD subunit
4*	0.016	COG2199 [T]	FOG: GGDEF domain
5*	0.016	COG2197 [T,K]	Response regulator containing a CheY-like receiver domain and an HTH DNA-binding domain
6	0.015	COG0735 [P]	Fe2+/Zn2+ uptake regulation proteins
7	0.015	COG1307 [S]	Uncharacterized protein conserved in bacteria
8	0.014	COG2876 [E]	3-deoxy-D-arabino-heptulosonate 7-phosphate (DAHP) synthase
9	0.013	COG0265 [O]	Trypsin-like serine proteases, typically periplasmic, contain C-terminal PDZ domain
10	0.012	COG2805 [N,U]	Tfp pilus assembly protein, pilus retraction ATPase PilT
11	0.011	COG0569 [P]	K+ transport systems, NAD-binding component
12	0.011	COG0621 [J]	2-methylthioadenine synthetase

Table S 34: Module 83.

Rank	Probability	OG	Description
1	0.014	COG0654 [H,C]	2-polyprenyl-6-methoxyphenol hydroxylase and related FAD-dependent oxidoreductases
2	0.013	COG0316 [S]	Uncharacterized conserved protein
3	0.010	COG0847 [L]	DNA polymerase III, epsilon subunit and related 3-5 exonucleases

Table S 35: Module 85.

Rank	Probability	OG	Description
1*	0.039	COG1145 [C]	Ferredoxin
2	0.020	COG0517 [R]	FOG: CBS domain
3	0.013	COG0655 [R]	Multimeric flavodoxin WrbA
4*	0.013	COG1035 [C]	Coenzyme F420-reducing hydrogenase, beta subunit
5	0.011	COG2710 [C]	Nitrogenase molybdenum-iron protein, alpha and beta chains
6*	0.011	COG1146 [C]	Ferredoxin

Table S 36: Module 87.

Rank	Probability	OG	Description
1*	0.197	COG1629 [P]	Outer membrane receptor proteins, mostly Fe transport
2*	0.060	COG1028 [I,Q,R]	Dehydrogenases with different specificities (related to short-chain alcohol dehydrogenases)
3*	0.033	COG0784 [T]	FOG: CheY-like receiver
4*	0.024	COG0625 [O]	Glutathione S-transferase
5	0.019	COG1228 [Q]	Imidazolonepropionase and related amidohydrolases
6*	0.019	COG0346 [E]	Lactoylglutathione lyase and related lyases
7	0.016	COG1680 [V]	Beta-lactamase class C and other penicillin binding proteins
8*	0.015	COG2234 [R]	Predicted aminopeptidases
9*	0.014	COG1506 [E]	Dipeptidyl aminopeptidases/acylaminoacyl-peptidases
10*	0.014	COG1595 [K]	DNA-directed RNA polymerase specialized sigma subunit, sigma24 homolog
11*	0.014	COG1538 [M,U]	Outer membrane protein
12	0.013	NOG10077 [-]	Tryptophan halogenase
13*	0.012	COG0308 [E]	Aminopeptidase N
14*	0.012	COG1609 [K]	Transcriptional regulators
15	0.011	COG1230 [P]	Co/Zn/Cd efflux system component
16*	0.010	COG3279 [K,T]	Response regulator of the LytR/AlgR family

Table S 37: Module 102.

Rank	Probability	OG	Description
1	0.028	COG1708 [R]	Predicted nucleotidyltransferases
2	0.026	COG1522 [K]	Transcriptional regulators
3	0.021	COG2250 [S]	Uncharacterized conserved protein related to C-terminal domain of eukaryotic chaperone, SACSIN
4	0.019	COG0467 [T]	RecA-superfamily ATPases implicated in signal transduction
5	0.016	COG0446 [R]	Uncharacterized NAD(FAD)-dependent dehydrogenases
6*	0.016	COG0517 [R]	FOG: CBS domain
7	0.016	COG1848 [R]	Predicted nucleic acid-binding protein, contains PIN domain
8	0.014	COG0535 [R]	Predicted Fe-S oxidoreductases
9	0.013	COG1672 [R]	Predicted ATPase (AAA+ superfamily)
10	0.013	COG0697 [G,E,R]	Permeases of the drug/metabolite transporter (DMT) superfamily
11	0.012	COG0433 [R]	Predicted ATPase
12	0.011	COG1373 [R]	Predicted ATPase (AAA+ superfamily)
13	0.011	COG0144 [J]	tRNA and rRNA cytosine-C5-methylases
14*	0.010	COG1011 [R]	Predicted hydrolase (HAD superfamily)

Table S 38: Module 103.

Rank	Probability	OG	Description
1*	0.324	COG1484 [L]	DNA replication protein
2*	0.280	COG4584 [L]	Transposase and inactivated derivatives
3	0.079	COG0582 [L]	Integrase
4	0.040	COG3344 [L]	Retron-type reverse transcriptase
5	0.037	COG1028 [I,Q,R]	Dehydrogenases with different specificities (related to short-chain alcohol dehydrogenases)
6	0.021	COG1475 [K]	Predicted transcriptional regulators
7*	0.019	COG4644 [L]	Transposase and inactivated derivatives, TnpA family
8	0.018	COG1846 [K]	Transcriptional regulators
9	0.012	NOG10792 [L]	Transposase
10	0.011	COG0439 [I]	Biotin carboxylase

Table S 39: Module 105.

Rank	Probability	OG	Description
1*	0.032	COG0561 [R]	Predicted hydrolases of the HAD superfamily
2	0.024	COG1132 [V]	ABC-type multidrug transport system, ATPase and permease components
3	0.023	COG5421 [L]	Transposase
4	0.020	COG1136 [V]	ABC-type antimicrobial peptide transport system, ATPase component
5	0.015	COG0531 [E]	Amino acid transporters
6	0.015	COG3839 [G]	ABC-type sugar transport systems, ATPase components
7*	0.014	COG0596 [R]	Predicted hydrolases or acyltransferases (alpha/beta hydrolase superfamily)
8	0.014	NOG46681 [S]	Lipoprotein
9	0.013	COG0595 [R]	Predicted hydrolase of the metallo-beta-lactamase superfamily
10*	0.013	COG0056 [C]	F0F1-type ATP synthase, alpha subunit
11	0.013	COG0618 [R]	Exopolyphosphatase-related proteins
12*	0.012	COG0055 [C]	F0F1-type ATP synthase, beta subunit
13	0.012	NOG12793 [S]	Calcium ion binding protein
14*	0.011	COG0508 [C]	Pyruvate/2-oxoglutarate dehydrogenase complex, dihydrolipoamide acyltransferase (E2) component, and related enzymes
15	0.011	COG1122 [P]	ABC-type cobalt transport system, ATPase component
16	0.010	COG0474 [P]	Cation transport ATPase
17	0.010	COG1307 [S]	Uncharacterized protein conserved in bacteria

Table S 40: Module 107.

Rank	Probability	OG	Description
1*	0.024	COG3121 [N,U]	P pilus assembly protein, chaperone PapD
2	0.021	COG3328 [L]	Transposase and inactivated derivatives
3*	0.020	COG3188 [N,U]	P pilus assembly protein, porin PapC
4*	0.019	COG3210 [U]	Large exoproteins involved in heme utilization or adhesion
5	0.013	COG0451 [M,G]	Nucleoside-diphosphate-sugar epimerases
6	0.013	COG1175 [G]	ABC-type sugar transport systems, permease components
7*	0.012	COG3501 [S]	Uncharacterized protein conserved in bacteria
8*	0.012	COG3157 [S]	Hemolysin-coregulated protein (uncharacterized)
9*	0.011	COG2831 [U]	Hemolysin activation/secretion protein
10*	0.011	COG3515 [S]	Uncharacterized protein conserved in bacteria
11	0.011	COG0395 [G]	ABC-type sugar transport system, permease component

Table S 41: Module 112.

Rank	Probability	OG	Description
1*	0.054	COG0477 [G,E,P,R]	Permeases of the major facilitator superfamily
2*	0.025	COG1309 [K]	Transcriptional regulator
3*	0.018	COG1028 [I,Q,R]	Dehydrogenases with different specificities (related to short-chain alcohol dehydrogenases)
4*	0.015	COG0642 [T]	Signal transduction histidine kinase
5*	0.011	COG0789 [K]	Predicted transcriptional regulators
6*	0.010	COG0318 [I,Q]	Acyl-CoA synthetases (AMP-forming)/AMP-acid ligases II

Table S 42: Module 117.

Rank	Probability	OG	Description
1	0.132	NOG12793 [S]	Calcium ion binding protein
2	0.090	NOG75023 [K]	Regulator protein
3	0.076	NOG73426 [K]	Regulator protein
4	0.047	NOG71304 [-]	Methyltransferase
5	0.040	NOG85258 [K]	Regulator protein
6	0.031	NOG122382 [L]	Transposase
7	0.026	NOG75518 [R]	Protease family
8	0.024	NOG120882 [K]	Regulator protein
9	0.023	NOG87366 [R]	Acetyltransferase
10	0.015	NOG26848 [T]	Anti sigma protein
11	0.015	NOG86872 [R]	Hydrolase
12	0.015	NOG138440 [C]	Ferredoxin protein
13	0.015	NOG85333 [S]	O-Antigen protein
14	0.015	NOG87019 [R]	Hydrolase
15	0.014	NOG27013 [K]	RNA polymerase
16	0.013	NOG76823 [T]	Regulator protein
17	0.013	NOG87647 [H]	Biosynthesis protein
18	0.013	NOG84808 [K]	Transcriptional regulator protein
19	0.013	NOG86494 [V]	HNH endonuclease
20	0.012	COG2159 [R]	Predicted metal-dependent hydrolase of the TIM-barrel fold
21	0.011	COG0204 [I]	1-acyl-sn-glycerol-3-phosphate acyltransferase
22	0.011	NOG148142 [-]	Acyl carrier protein
23	0.011	NOG44334 [S]	Annotation not available

Table S 43: Module 123.

Rank	Probability	OG	Description
1*	0.036	COG1609 [K]	Transcriptional regulators
2*	0.035	COG0583 [K]	Transcriptional regulator
3*	0.029	COG0834 [E,T]	ABC-type amino acid transport/signal transduction systems, periplasmic component/domain
4*	0.026	COG1028 [I,Q,R]	Dehydrogenases with different specificities (related to short-chain alcohol dehydrogenases)
5*	0.020	COG2197 [T,K]	Response regulator containing a CheY-like receiver domain and an HTH DNA-binding domain
6*	0.014	COG0840 [N,T]	Methyl-accepting chemotaxis protein
7*	0.013	COG1940 [K,G]	Transcriptional regulator/sugar kinase
8*	0.013	COG2200 [T]	FOG: EAL domain
9*	0.012	COG1024 [I]	Enoyl-CoA hydratase/carnithine racemase
10	0.012	COG1396 [K]	Predicted transcriptional regulators
11*	0.011	COG1349 [K,G]	Transcriptional regulators of sugar metabolism
12*	0.011	COG1670 [J]	Acetyltransferases, including N-acetylases of ribosomal proteins
13*	0.011	COG0765 [E]	ABC-type amino acid transport system, permease component
14	0.010	COG3842 [E]	ABC-type spermidine/putrescine transport systems, ATPase components
15*	0.010	COG1879 [G]	ABC-type sugar transport system, periplasmic component
16*	0.010	COG1566 [V]	Multidrug resistance efflux pump

Table S 44: Module 135.

Rank	Probability	OG	Description
1	0.065	COG4636 [S]	Uncharacterized protein conserved in cyanobacteria
2*	0.044	COG0515 [R,T,K,L]	Serine/threonine protein kinase
3*	0.036	COG0784 [T]	FOG: CheY-like receiver
4*	0.036	COG2203 [T]	FOG: GAF domain
5*	0.024	COG0457 [R]	FOG: TPR repeat
6*	0.024	COG0642 [T]	Signal transduction histidine kinase
7*	0.023	COG1357 [S]	Uncharacterized low-complexity proteins
8*	0.019	COG2319 [R]	FOG: WD40 repeat
9*	0.013	COG1716 [T]	FOG: FHA domain
10*	0.013	COG0664 [T]	cAMP-binding proteins - catabolite gene activator and regulatory subunit of cAMP-dependent protein kinases
11	0.012	COG5464 [S]	Uncharacterized conserved protein
12*	0.011	COG5635 [T]	Predicted NTPase (NACHT family)
13*	0.011	COG1413 [C]	FOG: HEAT repeat
14	0.011	COG3293 [L]	Transposase and inactivated derivatives
15*	0.011	COG2214 [O]	DnaJ-class molecular chaperone
16*	0.011	COG4995 [S]	Uncharacterized protein conserved in bacteria
17*	0.010	COG0715 [P]	ABC-type nitrate/sulfonate/bicarbonate transport systems, periplasmic components

Table S 45: Module 136.

Rank	Probability	OG	Description
1*	0.064	COG0477 [G,E,P,R]	Permeases of the major facilitator superfamily
2	0.030	COG0317 [T,K]	Guanosine polyphosphate pyrophosphohydrolases/synthetases
3	0.017	COG3202 [C]	ATP/ADP translocase
4*	0.015	COG0500 [Q,R]	SAM-dependent methyltransferases
5*	0.013	COG0457 [R]	FOG: TPR repeat
6*	0.011	COG1132 [V]	ABC-type multidrug transport system, ATPase and permease components
7	0.011	COG1373 [R]	Predicted ATPase (AAA+ superfamily)
8*	0.010	COG0591 [E,R]	Na+/proline symporter
9	0.010	COG5464 [S]	Uncharacterized conserved protein

Table S 46: Module 140.

Rank	Probability	OG	Description
1*	0.136	COG1028 [I,Q,R]	Dehydrogenases with different specificities (related to short-chain alcohol dehydrogenases)
2*	0.058	COG1012 [C]	NAD-dependent aldehyde dehydrogenases
3*	0.057	COG1309 [K]	Transcriptional regulator
4*	0.055	COG4638 [P,R]	Phenylpropionate dioxygenase and related ring-hydroxylating dioxygenases, large terminal subunit
5*	0.049	COG0596 [R]	Predicted hydrolases or acyltransferases (alpha/beta hydrolase superfamily)
6*	0.034	COG2072 [P]	Predicted flavoprotein involved in K+ transport
7*	0.033	COG0346 [E]	Lactoylglutathione lyase and related lyases
8*	0.025	COG2207 [K]	AraC-type DNA-binding domain-containing proteins
9*	0.024	COG2124 [Q]	Cytochrome P450
10*	0.023	COG2303 [E]	Choline dehydrogenase and related flavoproteins
11*	0.021	COG0657 [I]	Esterase/lipase
12*	0.017	COG2141 [C]	Coenzyme F420-dependent N5,N10-methylene tetrahydromethanopterin reductase and related flavin-dependent oxidoreductases
13*	0.016	COG5517 [Q]	Small subunit of phenylpropionate dioxygenase
14*	0.014	COG0654 [H,C]	2-polyprenyl-6-methoxyphenol hydroxylase and related FAD-dependent oxidoreductases
15*	0.013	COG2159 [R]	Predicted metal-dependent hydrolase of the TIM-barrel fold
16*	0.013	COG0446 [R]	Uncharacterized NAD(FAD)-dependent dehydrogenases
17*	0.012	COG1053 [C]	Succinate dehydrogenase/fumarate reductase, flavoprotein subunit
18*	0.011	COG3653 [Q]	N-acyl-D-aspartate/D-glutamate deacylase
19*	0.011	COG1335 [Q]	Amidases related to nicotinamidase
20*	0.010	COG3119 [P]	Arylsulfatase A and related enzymes

Table S 47: Module 141.

Rank	Probability	OG	Description
1	0.235	COG3181 [S]	Uncharacterized protein conserved in bacteria
2*	0.069	COG0583 [K]	Transcriptional regulator
3*	0.048	COG1028 [I,Q,R]	Dehydrogenases with different specificities (related to short-chain alcohol dehydrogenases)
4*	0.046	COG1804 [C]	Predicted acyl-CoA transferases/carnitine dehydratase
5*	0.034	COG1414 [K]	Transcriptional regulator
6*	0.017	COG1802 [K]	Transcriptional regulators
7*	0.017	COG0834 [E,T]	ABC-type amino acid transport/signal transduction systems, periplasmic component/domain
8*	0.016	COG0747 [E]	ABC-type dipeptide transport system, periplasmic component
9*	0.016	COG0251 [J]	Putative translation initiation inhibitor, yjgF family
10*	0.016	COG0765 [E]	ABC-type amino acid transport system, permease component
11	0.015	COG2079 [R]	Uncharacterized protein involved in propionate catabolism
12*	0.015	COG1126 [E]	ABC-type polar amino acid transport system, ATPase component
13*	0.014	COG1335 [Q]	Amidases related to nicotinamidase
14*	0.012	COG1173 [E,P]	ABC-type dipeptide/oligopeptide/nickel transport systems, permease components
15*	0.012	COG0028 [E,H]	Thiamine pyrophosphate-requiring enzymes [acetolactate synthase, pyruvate dehydrogenase (cytochrome), glyoxylate carboligase, phosphonopyruvate decarboxylase]
16*	0.012	COG1024 [I]	Enoyl-CoA hydratase/carnithine racemase
17*	0.012	COG0601 [E,P]	ABC-type dipeptide/oligopeptide/nickel transport systems, permease components
18*	0.011	COG1042 [C]	Acyl-CoA synthetase (NDP forming)

Table S 48: Module 145.

Rank	Probability	OG	Description
1*	0.045	COG1396 [K]	Predicted transcriptional regulators
2	0.017	COG3942 [R]	Surface antigen
3*	0.016	COG0477 [G,E,P,R]	Permeases of the major facilitator superfamily
4*	0.013	COG0463 [M]	Glycosyltransferases involved in cell wall biogenesis
5	0.013	COG1705 [N,U]	Muramidase (flagellum-specific)
6	0.012	COG0577 [V]	ABC-type antimicrobial peptide transport system, permease component
7	0.011	COG0698 [G]	Ribose 5-phosphate isomerase RpiB
8*	0.010	COG1309 [K]	Transcriptional regulator

Table S 49: Module 146.

Rank	Probability	OG	Description
1*	0.093	COG0500 [Q,R]	SAM-dependent methyltransferases
2	0.066	COG0270 [L]	Site-specific DNA methylase
3*	0.062	COG1012 [C]	NAD-dependent aldehyde dehydrogenases
4*	0.050	COG0438 [M]	Glycosyltransferase
5*	0.039	COG0110 [R]	Acetyltransferase (isoleucine patch superfamily)
6*	0.037	COG0513 [L,K,J]	Superfamily II DNA and RNA helicases
7	0.035	COG0317 [T,K]	Guanosine polyphosphate pyrophosphohydrolases/synthetases
8*	0.034	COG0517 [R]	FOG: CBS domain
9*	0.034	COG0596 [R]	Predicted hydrolases or acyltransferases (alpha/beta hydrolase superfamily)
10*	0.033	COG0318 [I,Q]	Acyl-CoA synthetases (AMP-forming)/AMP-acid ligases II
11*	0.027	COG0436 [E]	Aspartate/tyrosine/aromatic aminotransferase
12*	0.024	COG0457 [R]	FOG: TPR repeat
13	0.024	COG3727 [L]	DNA G:T-mismatch repair endonuclease
14*	0.023	COG0451 [M,G]	Nucleoside-diphosphate-sugar epimerases
15	0.021	COG2812 [L]	DNA polymerase III, gamma/tau subunits
16*	0.020	COG0242 [J]	N-formylmethionyl-tRNA deformylase
17*	0.019	COG1088 [M]	dTDP-D-glucose 4,6-dehydratase
18*	0.018	COG0465 [O]	ATP-dependent Zn proteases
19*	0.017	COG0662 [G]	Mannose-6-phosphate isomerase
20	0.016	COG0462 [F,E]	Phosphoribosylpyrophosphate synthetase
21*	0.016	COG0365 [I]	Acyl-coenzyme A synthetases/AMP-(fatty) acid ligases
22*	0.015	COG1053 [C]	Succinate dehydrogenase/fumarate reductase, flavoprotein subunit
23*	0.012	COG1131 [V]	ABC-type multidrug transport system, ATPase component
24	0.010	COG0547 [E]	Anthranilate phosphoribosyltransferase
25	0.010	NOG25517 [R]	Endonuclease

Table S 50: Module 151.

Rank	Probability	OG	Description
1	0.024	COG1595 [K]	DNA-directed RNA polymerase specialized sigma subunit, sigma24 homolog
2	0.023	COG1191 [K]	DNA-directed RNA polymerase specialized sigma subunit
3	0.021	COG0840 [N,T]	Methyl-accepting chemotaxis protein
4*	0.020	COG0726 [G]	Predicted xylanase/chitin deacetylase
5	0.019	COG3829 [K,T]	Transcriptional regulator containing PAS, AAA-type ATPase, and DNA-binding domains
6	0.016	COG1686 [M]	D-alanyl-D-alanine carboxypeptidase
7*	0.016	COG0739 [M]	Membrane proteins related to metalloendopeptidases
8	0.011	COG3773 [M]	Cell wall hydrolyses involved in spore germination
9*	0.011	COG1404 [O]	Subtilisin-like serine proteases
10	0.011	COG2323 [S]	Predicted membrane protein
11	0.010	COG2002 [K]	Regulators of stationary/sporulation gene expression
12	0.010	NOG04273 [-]	Germination protein

Table S 51: Module 161.

Rank	Probability	OG	Description
1	0.022	NOG12793 [S]	Calcium ion binding protein
2	0.021	COG0457 [R]	FOG: TPR repeat

Table S 52: Module 163.

Rank	Probability	OG	Description
1*	0.101	COG1638 [G]	TRAP-type C4-dicarboxylate transport system, periplasmic component
2*	0.094	COG1593 [G]	TRAP-type C4-dicarboxylate transport system, large permease component
3	0.049	COG0697 [G,E,R]	Permeases of the drug/metabolite transporter (DMT) superfamily
4*	0.047	COG3090 [G]	TRAP-type C4-dicarboxylate transport system, small permease component
5*	0.040	COG1802 [K]	Transcriptional regulators
6	0.035	COG3333 [S]	Uncharacterized protein conserved in bacteria
7	0.025	COG4663 [Q]	TRAP-type mannitol/chloroaromatic compound transport system, periplasmic component
8	0.024	COG2358 [R]	TRAP-type uncharacterized transport system, periplasmic component
9	0.024	COG4664 [Q]	TRAP-type mannitol/chloroaromatic compound transport system, large permease component
10*	0.024	COG0129 [E,G]	Dihydroxyacid dehydratase/phosphogluconate dehydratase
11*	0.023	COG2084 [I]	3-hydroxyisobutyrate dehydrogenase and related beta-hydroxyacid dehydrogenases
12*	0.021	COG0624 [E]	Acetylornithine deacetylase/Succinyl-diaminopimelate desuccinylase and related deacylases
13*	0.021	COG0277 [C]	FAD/FMN-containing dehydrogenases
14	0.020	COG4666 [R]	TRAP-type uncharacterized transport system, fused permease components
15	0.018	COG0679 [R]	Predicted permeases
16	0.015	COG1280 [E]	Putative threonine efflux protein
17*	0.014	COG0075 [E]	Serine-pyruvate aminotransferase/archaeal aspartate aminotransferase
18	0.014	COG4665 [Q]	TRAP-type mannitol/chloroaromatic compound transport system, small permease component
19	0.011	COG0526 [O,C]	Thiol-disulfide isomerase and thioredoxins
20*	0.011	COG2197 [T,K]	Response regulator containing a CheY-like receiver domain and an HTH DNA-binding domain
21*	0.010	COG0179 [Q]	2-keto-4-pentenoate hydratase/2-oxohepta-3-ene-1,7-dioic acid hydratase (catechol pathway)

Table S 53: Module 165.

Rank	Probability	OG	Description
1*	0.043	COG0477 [G,E,P,R]	Permeases of the major facilitator superfamily
2	0.021	COG3942 [R]	Surface antigen
3*	0.011	COG0451 [M,G]	Nucleoside-diphosphate-sugar epimerases
4	0.010	NOG150116 [S]	Annotation not available

Table S 54: Module 170.

Rank	Probability	OG	Description
1*	0.100	COG2204 [T]	Response regulator containing CheY-like receiver, AAA-type ATPase, and DNA-binding domains
2*	0.040	COG0642 [T]	Signal transduction histidine kinase
3	0.025	COG1032 [C]	Fe-S oxidoreductase
4*	0.015	COG0535 [R]	Predicted Fe-S oxidoreductases
5	0.014	COG0741 [M]	Soluble lytic murein transglycosylase and related regulatory proteins (some contain LysM/invasin domains)
6*	0.013	COG1538 [M,U]	Outer membrane protein
7	0.012	COG0437 [C]	Fe-S-cluster-containing hydrogenase components 1
8*	0.012	COG3604 [K,T]	Transcriptional regulator containing GAF, AAA-type ATPase, and DNA binding domains
9	0.010	COG2804 [N,U]	Type II secretory pathway, ATPase PulE/Tfp pilus assembly pathway, ATPase PilB

Table S 55: Module 171.

Rank	Probability	OG	Description
1	0.073	COG0438 [M]	Glycosyltransferase
2*	0.044	COG1518 [L]	Uncharacterized protein predicted to be involved in DNA repair
3	0.038	COG1366 [T]	Anti-anti-sigma regulatory factor (antagonist of anti-sigma factor)
4*	0.038	COG1203 [R]	Predicted helicases
5	0.033	COG0863 [L]	DNA modification methylase
6	0.029	COG0745 [T,K]	Response regulators consisting of a CheY-like receiver domain and a winged-helix DNA-binding domain
7*	0.023	COG1468 [L]	RecB family exonuclease
8*	0.020	COG1343 [L]	Uncharacterized protein predicted to be involved in DNA repair
9	0.020	COG0119 [E]	Isopropylmalate/homocitrate/citramalate synthases
10	0.020	COG1669 [R]	Predicted nucleotidyltransferases
11	0.016	COG2172 [T]	Anti-sigma regulatory factor (Ser/Thr protein kinase)
12	0.014	COG1002 [V]	Type II restriction enzyme, methylase subunits
13	0.013	COG4636 [S]	Uncharacterized protein conserved in cyanobacteria
14*	0.013	COG3649 [L]	Uncharacterized protein predicted to be involved in DNA repair
15	0.012	COG0205 [G]	6-phosphofructokinase
16	0.012	COG0474 [P]	Cation transport ATPase
17	0.012	COG2361 [S]	Uncharacterized conserved protein
18*	0.012	NOG10084 [L]	Crispr-Associated protein
19	0.011	COG0367 [E]	Asparagine synthase (glutamine-hydrolyzing)
20	0.010	COG2189 [L]	Adenine specific DNA methylase Mod

Table S 56: Module 172.

Rank	Probability	OG	Description
1*	0.092	COG0515 [R,T,K,L]	Serine/threonine protein kinase
2*	0.082	COG0577 [V]	ABC-type antimicrobial peptide transport system, permease component
3*	0.059	COG3119 [P]	Arylsulfatase A and related enzymes
4*	0.045	COG0673 [R]	Predicted dehydrogenases and related proteins
5*	0.040	COG0457 [R]	FOG: TPR repeat
6*	0.028	COG1595 [K]	DNA-directed RNA polymerase specialized sigma subunit, sigma24 homolog
7	0.022	COG1695 [K]	Predicted transcriptional regulators
8*	0.021	COG0500 [Q,R]	SAM-dependent methyltransferases
9*	0.020	COG1131 [V]	ABC-type multidrug transport system, ATPase component
10*	0.017	COG2234 [R]	Predicted aminopeptidases
11*	0.017	COG0845 [M]	Membrane-fusion protein
12*	0.017	COG0823 [U]	Periplasmic component of the Tol biopolymer transport system
13*	0.016	COG3391 [S]	Uncharacterized conserved protein
14*	0.015	COG5616 [S]	Predicted integral membrane protein
15	0.014	NOG72404 [R]	Glycosyl hydrolase
16*	0.013	COG1228 [Q]	Imidazolonepropionase and related amidohydrolases
17	0.013	NOG87301 [-]	Aspic/Unbv protein
18*	0.013	COG4948 [M,R]	L-alanine-DL-glutamate epimerase and related enzymes of enolase superfamily
19*	0.012	COG0531 [E]	Amino acid transporters
20*	0.012	COG1680 [V]	Beta-lactamase class C and other penicillin binding proteins
21*	0.011	COG0265 [O]	Trypsin-like serine proteases, typically periplasmic, contain C-terminal PDZ domain
22*	0.011	COG1506 [E]	Dipeptidyl aminopeptidases/acylaminoacyl-peptidases
23*	0.011	COG0667 [C]	Predicted oxidoreductases (related to aryl-alcohol dehydrogenases)
24*	0.010	COG1082 [G]	Sugar phosphate isomerases/epimerases
25	0.010	NOG43592 [K]	RNA polymerase
26*	0.010	COG0657 [I]	Esterase/lipase

Table S 57: Module 174.

Rank	Probability	OG	Description
1*	0.029	COG0583 [K]	Transcriptional regulator
2	0.016	NOG04436 [L]	Transposase
3*	0.014	COG0531 [E]	Amino acid transporters
4	0.013	COG1404 [O]	Subtilisin-like serine proteases
5*	0.013	COG0745 [T,K]	Response regulators consisting of a CheY-like receiver domain and a winged-helix DNA-binding domain
6*	0.013	COG0845 [M]	Membrane-fusion protein
7	0.012	COG0810 [M]	Periplasmic protein TonB, links inner and outer membranes
8	0.012	COG5295 [U,W]	Autotransporter adhesin
9	0.011	COG3209 [M]	Rhs family protein
10	0.011	COG1506 [E]	Dipeptidyl aminopeptidases/acylaminoacyl-peptidases
11	0.011	COG3039 [L]	Transposase and inactivated derivatives, IS5 family

Table S 58: Module 178.

Rank	Probability	OG	Description
1*	0.067	COG1846 [K]	Transcriptional regulators
2	0.024	COG1887 [M]	Putative glycosyl/glycerophosphate transferases involved in teichoic acid biosynthesis TagF/TagB/EpsJ/RodC
3*	0.022	COG1309 [K]	Transcriptional regulator
4	0.019	COG0577 [V]	ABC-type antimicrobial peptide transport system, permease component
5*	0.019	COG1396 [K]	Predicted transcriptional regulators
6	0.019	COG1266 [R]	Predicted metal-dependent membrane protease
7*	0.019	COG2207 [K]	AraC-type DNA-binding domain-containing proteins
8	0.017	COG2111 [P]	Multisubunit Na+/H+ antiporter, MnhB subunit
9*	0.016	COG0789 [K]	Predicted transcriptional regulators
10	0.015	COG1174 [E]	ABC-type proline/glycine betaine transport systems, permease component
11	0.015	COG1670 [J]	Acetyltransferases, including N-acetylases of ribosomal proteins
12	0.014	COG1063 [E,R]	Threonine dehydrogenase and related Zn-dependent dehydrogenases
13	0.013	COG3279 [K,T]	Response regulator of the LytR/AlgR family
14	0.012	COG0739 [M]	Membrane proteins related to metalloendopeptidases
15	0.012	COG1388 [M]	FOG: LysM repeat
16	0.010	COG0095 [H]	Lipoate-protein ligase A
17	0.010	COG1937 [S]	Uncharacterized protein conserved in bacteria
18	0.010	COG0735 [P]	Fe2+/Zn2+ uptake regulation proteins

Table S 59: Module 180.

Rank	Probability	OG	Description
1*	0.059	COG1609 [K]	Transcriptional regulators
2*	0.055	COG1653 [G]	ABC-type sugar transport system, periplasmic component
3*	0.044	COG0395 [G]	ABC-type sugar transport system, permease component
4*	0.040	COG1175 [G]	ABC-type sugar transport systems, permease components
5*	0.039	COG0366 [G]	Glycosidases
6*	0.039	COG0673 [R]	Predicted dehydrogenases and related proteins
7	0.026	COG1366 [T]	Anti-anti-sigma regulatory factor (antagonist of anti-sigma factor)
8*	0.026	COG2730 [G]	Endoglucanase
9*	0.024	COG1940 [K,G]	Transcriptional regulator/sugar kinase
10*	0.023	COG1082 [G]	Sugar phosphate isomerases/epimerases
11*	0.020	COG2723 [G]	Beta-glucosidase/6-phospho-beta-glucosidase/beta-galactosidase
12*	0.018	COG1070 [G]	Sugar (pentulose and hexulose) kinases
13*	0.018	COG1486 [G]	Alpha-galactosidases/6-phospho-beta-glucosidases, family 4 of glycosyl hydrolases
14	0.015	COG1132 [V]	ABC-type multidrug transport system, ATPase and permease components
15	0.015	COG1305 [E]	Transglutaminase-like enzymes, putative cysteine proteases
16*	0.015	COG1472 [G]	Beta-glucosidase-related glycosidases
17*	0.013	COG3507 [G]	Beta-xylosidase
18*	0.012	COG1349 [K,G]	Transcriptional regulators of sugar metabolism
19*	0.012	COG1874 [G]	Beta-galactosidase
20*	0.012	COG3693 [G]	Beta-1,4-xylanase

Table S 60: Module 182.

Rank	Probability	OG	Description
1*	0.037	COG0526 [O,C]	Thiol-disulfide isomerase and thioredoxins
2	0.021	COG2353 [S]	Uncharacterized conserved protein
3*	0.021	COG0500 [Q,R]	SAM-dependent methyltransferases
4	0.012	COG1451 [R]	Predicted metal-dependent hydrolase
5	0.012	COG1610 [S]	Uncharacterized conserved protein
6	0.012	COG3419 [N,U]	Tfp pilus assembly protein, tip-associated adhesin PilY1
7	0.011	COG0665 [E]	Glycine/D-amino acid oxidases (deaminating)
8	0.011	COG2826 [L]	Transposase and inactivated derivatives, IS30 family
9	0.010	COG1807 [M]	4-amino-4-deoxy-L-arabinose transferase and related glycosyltransferases of PMT family
10	0.010	COG2805 [N,U]	Tfp pilus assembly protein, pilus retraction ATPase PilT
11	0.010	COG4969 [N,U]	Tfp pilus assembly protein, major pilin PilA

Table S 61: Module 183.

Rank	Probability	OG	Description
1*	0.181	COG0732 [V]	Restriction endonuclease S subunits
2*	0.161	COG0286 [V]	Type I restriction-modification system methyltransferase subunit
3*	0.118	COG0610 [V]	Type I site-specific restriction-modification system, R (restriction) subunit and related helicases
4	0.057	COG0582 [L]	Integrase
5	0.055	COG0210 [L]	Superfamily I DNA and RNA helicases
6	0.052	COG0553 [K,L]	Superfamily II DNA/RNA helicases, SNF2 family
7*	0.035	COG3177 [S]	Uncharacterized conserved protein
8*	0.033	COG4096 [V]	Type I site-specific restriction-modification system, R (restriction) subunit and related helicases
9*	0.033	COG1451 [R]	Predicted metal-dependent hydrolase
10	0.029	COG2865 [K]	Predicted transcriptional regulator containing an HTH domain and an uncharacterized domain shared with the mammalian protein Schlafen
11	0.020	COG0513 [L,K,J]	Superfamily II DNA and RNA helicases
12	0.015	COG2189 [L]	Adenine specific DNA methylase Mod
13	0.013	COG0581 [P]	ABC-type phosphate transport system, permease component
14	0.012	COG1295 [S]	Predicted membrane protein
15	0.012	COG3943 [R]	Virulence protein
16	0.010	COG0104 [F]	Adenylosuccinate synthase
17	0.010	COG2856 [E]	Predicted Zn peptidase

Table S 62: Module 184.

Rank	Probability	OG	Description
1*	0.028	COG2199 [T]	FOG: GGDEF domain
2	0.024	COG0531 [E]	Amino acid transporters
3	0.020	COG3188 [N,U]	P pilus assembly protein, porin PapC
4*	0.019	COG2200 [T]	FOG: EAL domain
5	0.017	COG0582 [L]	Integrase
6	0.016	COG1063 [E,R]	Threonine dehydrogenase and related Zn-dependent dehydrogenases
7	0.012	COG4948 [M,R]	L-alanine-DL-glutamate epimerase and related enzymes of enolase superfamily

Table S 63: Module 185.

Rank	Probability	OG	Description
1*	0.084	COG0457 [R]	FOG: TPR repeat
2*	0.037	COG1192 [D]	ATPases involved in chromosome partitioning
3	0.025	COG0739 [M]	Membrane proteins related to metalloendopeptidases
4	0.017	COG0795 [R]	Predicted permeases
5	0.013	COG0330 [O]	Membrane protease subunits, stomatin/prohibitin homologs
6	0.013	COG1022 [I]	Long-chain acyl-CoA synthetases (AMP-forming)
7*	0.012	COG0714 [R]	MoxR-like ATPases
8	0.012	COG4166 [E]	ABC-type oligopeptide transport system, periplasmic component
9	0.012	COG4591 [M]	ABC-type transport system, involved in lipoprotein release, permease component
10	0.012	COG1664 [M]	Integral membrane protein CcmA involved in cell shape determination
11*	0.011	COG2304 [R]	Uncharacterized protein containing a von Willebrand factor type A (vWA) domain
12*	0.010	COG0326 [O]	Molecular chaperone, HSP90 family

Table S 64: Module 186.

Rank	Probability	OG	Description
1*	0.161	COG0683 [E]	ABC-type branched-chain amino acid transport systems, periplasmic component
2*	0.114	COG0410 [E]	ABC-type branched-chain amino acid transport systems, ATPase component
3*	0.112	COG0559 [E]	Branched-chain amino acid ABC-type transport system, permease components
4*	0.110	COG4177 [E]	ABC-type branched-chain amino acid transport system, permease component
5*	0.099	COG0411 [E]	ABC-type branched-chain amino acid transport systems, ATPase component
6*	0.031	COG1116 [P]	ABC-type nitrate/sulfonate/bicarbonate transport system, ATPase component
7*	0.031	COG0600 [P]	ABC-type nitrate/sulfonate/bicarbonate transport system, permease component
8*	0.024	COG3842 [E]	ABC-type spermidine/putrescine transport systems, ATPase components
9	0.022	COG0642 [T]	Signal transduction histidine kinase
10*	0.017	COG0154 [J]	Asp-tRNAAsn/Glu-tRNAGln amidotransferase A subunit and related amidases
11	0.015	COG0446 [R]	Uncharacterized NAD(FAD)-dependent dehydrogenases
12*	0.013	COG0365 [I]	Acyl-coenzyme A synthetases/AMP-(fatty) acid ligases
13*	0.013	COG1177 [E]	ABC-type spermidine/putrescine transport system, permease component II
14*	0.012	COG0687 [E]	Spermidine/putrescine-binding periplasmic protein

Table S 65: Module 187.

Rank	Probability	OG	Description
1	0.018	COG0814 [E]	Amino acid permeases
2	0.013	COG3203 [M]	Outer membrane protein (porin)
3	0.013	COG0607 [P]	Rhodanese-related sulfurtransferase
4	0.012	COG1187 [J]	16S rRNA uridine-516 pseudouridylate synthase and related pseudouridylate synthases
5	0.012	COG0716 [C]	Flavodoxins
6	0.010	COG3005 [C]	Nitrate/TMAO reductases, membrane-bound tetraheme cytochrome c subunit

Table S 66: Module 188.

Rank	Probability	OG	Description
1*	0.024	COG0845 [M]	Membrane-fusion protein
2*	0.018	COG2197 [T,K]	Response regulator containing a CheY-like receiver domain and an HTH DNA-binding domain
3*	0.012	COG2931 [Q]	RTX toxins and related Ca2+-binding proteins
4	0.012	COG1413 [C]	FOG: HEAT repeat
5	0.011	COG0633 [C]	Ferredoxin
6	0.010	COG1032 [C]	Fe-S oxidoreductase
7*	0.010	COG2274 [V]	ABC-type bacteriocin/lantibiotic exporters, contain an N-terminal double-glycine peptidase domain

Table S 67: Module 190.

Rank	Probability	OG	Description
1	0.012	COG1357 [S]	Uncharacterized low-complexity proteins

Table S 68: Module 192.

Rank	Probability	OG	Description
1	0.059	COG1487 [R]	Predicted nucleic acid-binding protein, contains PIN domain
2	0.046	COG3093 [R]	Plasmid maintenance system antidote protein
3	0.043	COG0845 [M]	Membrane-fusion protein
4	0.041	COG1132 [V]	ABC-type multidrug transport system, ATPase and permease components
5*	0.035	COG3550 [R]	Uncharacterized protein related to capsule biosynthesis enzymes
6	0.033	COG3177 [S]	Uncharacterized conserved protein
7	0.032	COG3668 [R]	Plasmid stabilization system protein
8	0.027	COG3549 [R]	Plasmid maintenance system killer protein
9*	0.024	COG2944 [K]	Predicted transcriptional regulator
10	0.021	COG4974 [L]	Site-specific recombinase XerD
11*	0.020	COG2026 [J,D]	Cytotoxic translational repressor of toxin-antitoxin stability system
12*	0.020	COG2161 [D]	Antitoxin of toxin-antitoxin stability system
13	0.019	COG3636 [K]	Predicted transcriptional regulator
14	0.019	COG3657 [S]	Uncharacterized protein conserved in bacteria
15	0.018	COG1598 [S]	Uncharacterized conserved protein
16	0.018	COG2929 [S]	Uncharacterized protein conserved in bacteria
17*	0.016	COG2337 [T]	Growth inhibitor
18	0.016	COG4679 [S]	Phage-related protein
19	0.014	COG5606 [S]	Uncharacterized conserved small protein
20*	0.014	COG3077 [L]	DNA-damage-inducible protein J
21	0.013	COG0304 [I,Q]	3-oxoacyl-(acyl-carrier-protein) synthase
22	0.013	COG0702 [M,G]	Predicted nucleoside-diphosphate-sugar epimerases
23	0.012	COG3744 [S]	Uncharacterized protein conserved in bacteria
24*	0.011	COG4115 [S]	Uncharacterized protein conserved in bacteria
25*	0.010	COG3041 [S]	Uncharacterized protein conserved in bacteria

Table S 69: Module 195.

Rank	Probability	OG	Description
1	0.073	COG0583 [K]	Transcriptional regulator
2	0.048	COG1475 [K]	Predicted transcriptional regulators
3	0.030	COG2217 [P]	Cation transport ATPase
4	0.029	COG1961 [L]	Site-specific recombinases, DNA invertase Pin homologs
5*	0.028	COG3505 [U]	Type IV secretory pathway, VirD4 components
6	0.027	COG0582 [L]	Integrase
7	0.025	COG1192 [D]	ATPases involved in chromosome partitioning
8*	0.025	COG3846 [U]	Type IV secretory pathway, TrbL components
9*	0.024	COG5489 [S]	Uncharacterized conserved protein
10*	0.024	COG3504 [U]	Type IV secretory pathway, VirB9 components
11*	0.023	COG2948 [U]	Type IV secretory pathway, VirB10 components
12*	0.022	COG3451 [U]	Type IV secretory pathway, VirB4 components
13	0.020	COG2132 [Q]	Putative multicopper oxidases
14*	0.019	COG3838 [U]	Type IV secretory pathway, VirB2 components (pilins)
15*	0.018	COG3843 [U]	Type IV secretory pathway, VirD2 components (relaxase)
16*	0.018	COG5314 [U]	Conjugal transfer/entry exclusion protein
17	0.017	COG0741 [M]	Soluble lytic murein transglycosylase and related regulatory proteins (some contain LysM/invasin domains)
18*	0.017	COG3701 [U]	Type IV secretory pathway, TrbF components
19	0.017	COG4962 [U]	Flp pilus assembly protein, ATPase CpaF
20	0.017	COG0845 [M]	Membrane-fusion protein
21*	0.016	COG4959 [O,U]	Type IV secretory pathway, protease TraF
22*	0.014	COG5268 [N,U]	Type IV secretory pathway, TrbD component
23*	0.013	COG4227 [L]	Antirestriction protein
24*	0.012	COG5639 [S]	Uncharacterized conserved small protein
25	0.012	NOG83182 [R]	Methylase
26*	0.012	COG5534 [L]	Plasmid replication initiator protein
27	0.011	COG0330 [O]	Membrane protease subunits, stomatin/prohibitin homologs
28	0.011	NOG10283 [S]	Annotation not available
29*	0.011	NOG14076 [S]	Annotation not available
30	0.010	COG4625 [S]	Uncharacterized protein with a C-terminal OMP (outer membrane protein) domain

Table S 70: Module 198.

Rank	Probability	OG	Description
1*	0.525	COG3335 [L]	Transposase and inactivated derivatives
2	0.168	COG3293 [L]	Transposase and inactivated derivatives
3*	0.041	COG3415 [L]	Transposase and inactivated derivatives
4	0.013	COG0286 [V]	Type I restriction-modification system methyltransferase subunit
5	0.012	COG0491 [R]	Zn-dependent hydrolases, including glyoxylases
6	0.010	COG0591 [E,R]	Na+/proline symporter

Table S 71: Module 69 – with rank 4 according to estimated information content.

Rank	Probability	OG	Description
1	0.494	COG3328 [L]	Transposase and inactivated derivatives
2	0.154	COG2826 [L]	Transposase and inactivated derivatives, IS30 family
3	0.035	NOG122382 [L]	Transposase
4	0.029	COG1961 [L]	Site-specific recombinases, DNA invertase Pin homologs
5*	0.027	COG1028 [I,Q,R]	Dehydrogenases with different specificities (related to short-chain alcohol dehydrogenases)
6	0.018	NOG87598 [L]	Transposase
7	0.017	COG1914 [P]	Mn2+ and Fe2+ transporters of the NRAMP family
8	0.011	COG0261 [J]	Ribosomal protein L21
9*	0.010	COG0039 [C]	Malate/lactate dehydrogenases
10*	0.010	COG0282 [C]	Acetate kinase

Table S 72: Module 200 – with rank 7 according to estimated information content.

Rank	Probability	OG	Description
1*	0.310	COG3293 [L]	Transposase and inactivated derivatives
2*	0.212	COG3039 [L]	Transposase and inactivated derivatives, IS5 family
3*	0.122	COG2801 [L]	Transposase and inactivated derivatives
4	0.079	COG5659 [L]	FOG: Transposase
5	0.054	NOG137074 [L]	Transposase
6	0.047	COG2826 [L]	Transposase and inactivated derivatives, IS30 family

Table S 73: Module 126 – with rank 8 according to estimated information content.

Rank	Probability	OG	Description
1*	0.203	COG0614 [P]	ABC-type Fe3+-hydroxamate transport system, periplasmic component
2*	0.155	COG0609 [P]	ABC-type Fe3+-siderophore transport system, permease component
3*	0.137	COG1120 [P,H]	ABC-type cobalamin/Fe3+-siderophores transport systems, ATPase components
4	0.078	COG0500 [Q,R]	SAM-dependent methyltransferases
5	0.048	COG0454 [K,R]	Histone acetyltransferase HPA2 and related acetyltransferases
6	0.038	COG1136 [V]	ABC-type antimicrobial peptide transport system, ATPase component
7*	0.034	COG1121 [P]	ABC-type Mn/Zn transport systems, ATPase component
8*	0.033	COG0803 [P]	ABC-type metal ion transport system, periplasmic component/surface adhesin
9*	0.032	COG1108 [P]	ABC-type Mn2+/Zn2+ transport systems, permease components
10	0.030	COG1840 [P]	ABC-type Fe3+ transport system, periplasmic component
11	0.016	COG1451 [R]	Predicted metal-dependent hydrolase
12	0.015	COG0352 [H]	Thiamine monophosphate synthase
13	0.011	COG1073 [R]	Hydrolases of the alpha/beta superfamily
14	0.011	COG0452 [H]	Phosphopantothenoylcysteine synthetase/decarboxylase

Table S 74: Module 131 – with rank 14 according to estimated information content.

Rank	Probability	OG	Description
1	0.436	COG1943 [L]	Transposase and inactivated derivatives
2	0.038	COG0726 [G]	Predicted xylanase/chitin deacetylase
3	0.034	COG1249 [C]	Pyruvate/2-oxoglutarate dehydrogenase complex, dihydrolipoamide dehydrogenase (E3) component, and related enzymes
4	0.032	COG0697 [G,E,R]	Permeases of the drug/metabolite transporter (DMT) superfamily
5*	0.026	COG0671 [I]	Membrane-associated phospholipid phosphatase
6	0.023	COG0419 [L]	ATPase involved in DNA repair
7	0.023	COG0436 [E]	Aspartate/tyrosine/aromatic aminotransferase
8	0.022	COG1902 [C]	NADH:flavin oxidoreductases, Old Yellow Enzyme family
9	0.016	COG0006 [E]	Xaa-Pro aminopeptidase
10	0.016	COG0577 [V]	ABC-type antimicrobial peptide transport system, permease component
11	0.016	COG1266 [R]	Predicted metal-dependent membrane protease
12	0.015	COG0668 [M]	Small-conductance mechanosensitive channel
13*	0.014	COG0558 [I]	Phosphatidylglycerophosphate synthase

Table S 75: Module 43 – with rank 15 according to estimated information content.

Rank	Probability	OG	Description
1*	0.275	COG1020 [Q]	Non-ribosomal peptide synthetase modules and related proteins
2*	0.219	COG3321 [Q]	Polyketide synthase modules and related proteins
3*	0.033	COG3319 [Q]	Thioesterase domains of type I polyketide synthases or non-ribosomal peptide synthetases
4*	0.030	COG0318 [I,Q]	Acyl-CoA synthetases (AMP-forming)/AMP-acid ligases II
5*	0.027	COG3208 [Q]	Predicted thioesterase involved in non-ribosomal peptide biosynthesis
6*	0.021	COG0454 [K,R]	Histone acetyltransferase HPA2 and related acetyltransferases
7	0.019	COG0443 [O]	Molecular chaperone
8	0.013	COG1733 [K]	Predicted transcriptional regulators
9*	0.012	COG0500 [Q,R]	SAM-dependent methyltransferases
10	0.011	COG2801 [L]	Transposase and inactivated derivatives
11*	0.010	COG0160 [E]	4-aminobutyrate aminotransferase and related aminotransferases
12*	0.010	COG1357 [S]	Uncharacterized low-complexity proteins
13*	0.010	COG0367 [E]	Asparagine synthase (glutamine-hydrolyzing)

Table S 76: Module 120 – with rank 18 according to estimated information content.

Rank	Probability	OG	Description
1	0.334	COG5433 [L]	Transposase
2*	0.123	COG1028 [I,Q,R]	Dehydrogenases with different specificities (related to short-chain alcohol dehydrogenases)
3*	0.091	COG0477 [G,E,P,R]	Permeases of the major facilitator superfamily
4*	0.062	COG0500 [Q,R]	SAM-dependent methyltransferases
5*	0.032	COG0526 [O,C]	Thiol-disulfide isomerase and thioredoxins
6	0.031	COG0635 [H]	Coproporphyrinogen III oxidase and related Fe-S oxidoreductases
7*	0.025	COG0577 [V]	ABC-type antimicrobial peptide transport system, permease component
8*	0.024	COG1012 [C]	NAD-dependent aldehyde dehydrogenases
9*	0.017	COG1230 [P]	Co/Zn/Cd efflux system component
10*	0.016	COG1131 [V]	ABC-type multidrug transport system, ATPase component
11*	0.012	COG0596 [R]	Predicted hydrolases or acyltransferases (alpha/beta hydrolase superfamily)
12	0.012	COG1835 [I]	Predicted acyltransferases
13*	0.010	COG0621 [J]	2-methylthioadenine synthetase

Table S 77: Module 92 – with rank 19 according to estimated information content.

Rank	Probability	OG	Description
1*	0.121	COG1175 [G]	ABC-type sugar transport systems, permease components
2*	0.117	COG1653 [G]	ABC-type sugar transport system, periplasmic component
3*	0.113	COG0395 [G]	ABC-type sugar transport system, permease component
4*	0.066	COG3839 [G]	ABC-type sugar transport systems, ATPase components
5*	0.062	COG0747 [E]	ABC-type dipeptide transport system, periplasmic component
6*	0.059	COG1173 [E,P]	ABC-type dipeptide/oligopeptide/nickel transport systems, permease components
7*	0.057	COG0601 [E,P]	ABC-type dipeptide/oligopeptide/nickel transport systems, permease components
8*	0.047	COG0673 [R]	Predicted dehydrogenases and related proteins
9*	0.026	COG0444 [E,P]	ABC-type dipeptide/oligopeptide/nickel transport system, ATPase component
10	0.022	COG0640 [K]	Predicted transcriptional regulators
11	0.021	COG0454 [K,R]	Histone acetyltransferase HPA2 and related acetyltransferases
12*	0.020	COG4608 [E]	ABC-type oligopeptide transport system, ATPase component
13*	0.019	COG0329 [E,M]	Dihydrodipicolinate synthase/N-acetylneuraminate lyase
14*	0.018	COG1011 [R]	Predicted hydrolase (HAD superfamily)
15*	0.013	COG0637 [R]	Predicted phosphatase/phosphohexomutase
16	0.013	COG1744 [R]	Uncharacterized ABC-type transport system, periplasmic component/surface lipoprotein
17	0.013	COG3845 [R]	ABC-type uncharacterized transport systems, ATPase components
18	0.012	COG1079 [R]	Uncharacterized ABC-type transport system, permease component
19	0.011	COG4603 [R]	ABC-type uncharacterized transport system, permease component
20*	0.011	COG1940 [K,G]	Transcriptional regulator/sugar kinase

Table S 78: Module 194 – with rank 20 according to estimated information content.

Rank	Probability	OG	Description
1*	0.178	COG1960 [I]	Acyl-CoA dehydrogenases
2*	0.131	COG1024 [I]	Enoyl-CoA hydratase/carnithine racemase
3*	0.100	COG0318 [I,Q]	Acyl-CoA synthetases (AMP-forming)/AMP-acid ligases II
4*	0.080	COG0183 [I]	Acetyl-CoA acetyltransferase
5*	0.050	COG1804 [C]	Predicted acyl-CoA transferases/carnitine dehydratase
6*	0.037	COG2030 [I]	Acyl dehydratase
7*	0.034	COG0491 [R]	Zn-dependent hydrolases, including glyoxylases
8*	0.029	COG2050 [Q]	Uncharacterized protein, possibly involved in aromatic compounds catabolism
9*	0.022	COG0604 [C,R]	NADPH:quinone reductase and related Zn-dependent oxidoreductases
10*	0.019	COG0154 [J]	Asp-tRNAAsn/Glu-tRNAGln amidotransferase A subunit and related amidases
11*	0.019	COG1250 [I]	3-hydroxyacyl-CoA dehydrogenase
12*	0.016	COG1902 [C]	NADH:flavin oxidoreductases, Old Yellow Enzyme family
13*	0.015	COG0365 [I]	Acyl-coenzyme A synthetases/AMP-(fatty) acid ligases
14*	0.014	COG0028 [E,H]	Thiamine pyrophosphate-requiring enzymes [acetolactate synthase, pyruvate dehydrogenase (cytochrome), glyoxylate carboligase, phosphonopyruvate decarboxylase]
15*	0.013	COG0824 [R]	Predicted thioesterase
16*	0.012	COG0179 [Q]	2-keto-4-pentenoate hydratase/2-oxohepta-3-ene-1,7-dioic acid hydratase (catechol pathway)
17*	0.011	COG2301 [G]	Citrate lyase beta subunit

Table S 79: Module 144 – with rank 21 according to estimated information content.

Rank	Probability	OG	Description
1*	0.291	COG0457 [R]	FOG: TPR repeat
2*	0.128	COG0500 [Q,R]	SAM-dependent methyltransferases
3*	0.061	COG0463 [M]	Glycosyltransferases involved in cell wall biogenesis
4*	0.036	COG1404 [O]	Subtilisin-like serine proteases
5*	0.034	COG0265 [O]	Trypsin-like serine proteases, typically periplasmic, contain C-terminal PDZ domain
6*	0.031	COG1807 [M]	4-amino-4-deoxy-L-arabinose transferase and related glycosyltransferases of PMT family
7*	0.026	COG0436 [E]	Aspartate/tyrosine/aromatic aminotransferase
8*	0.024	COG0236 [I,Q]	Acyl carrier protein
9	0.023	COG2165 [N,U]	Type II secretory pathway, pseudopilin PulG
10*	0.021	COG1132 [V]	ABC-type multidrug transport system, ATPase and permease components
11*	0.020	COG0526 [O,C]	Thiol-disulfide isomerase and thioredoxins
12*	0.020	COG0612 [R]	Predicted Zn-dependent peptidases
13*	0.019	COG0705 [R]	Uncharacterized membrane protein (homolog of Drosophila rhomboid)
14	0.018	COG2132 [Q]	Putative multicopper oxidases
15*	0.014	COG1181 [M]	D-alanine-D-alanine ligase and related ATP-grasp enzymes
16*	0.014	COG0666 [R]	FOG: Ankyrin repeat
17	0.014	COG1235 [R]	Metal-dependent hydrolases of the beta-lactamase superfamily I
18	0.011	COG0628 [R]	Predicted permease
19	0.011	COG1525 [L]	Micrococcal nuclease (thermonuclease) homologs
20*	0.011	COG0760 [O]	Parvulin-like peptidyl-prolyl isomerase

Table S 80: Module 176 – with rank 22 according to estimated information content.

Rank	Probability	OG	Description
1	0.389	COG3464 [L]	Transposase and inactivated derivatives
2	0.054	COG0438 [M]	Glycosyltransferase
3	0.051	COG1396 [K]	Predicted transcriptional regulators
4	0.050	COG0494 [L,R]	NTP pyrophosphohydrolases including oxidative damage repair enzymes
5*	0.042	COG1012 [C]	NAD-dependent aldehyde dehydrogenases
6*	0.020	COG0431 [R]	Predicted flavoprotein
7*	0.019	COG0057 [G]	Glyceraldehyde-3-phosphate dehydrogenase/erythrose-4-phosphate dehydrogenase
8	0.018	COG0134 [E]	Indole-3-glycerol phosphate synthase
9	0.018	COG0768 [M]	Cell division protein FtsI/penicillin-binding protein 2
10	0.016	COG0199 [J]	Ribosomal protein S14
11	0.013	COG0231 [J]	Translation elongation factor P (EF-P)/translation initiation factor 5A (eIF-5A)
12*	0.013	COG0329 [E,M]	Dihydrodipicolinate synthase/N-acetylneuraminate lyase
13*	0.013	COG2151 [R]	Predicted metal-sulfur cluster biosynthetic enzyme
14	0.011	COG1214 [O]	Inactive homolog of metal-dependent proteases, putative molecular chaperone
15*	0.011	COG1454 [C]	Alcohol dehydrogenase, class IV
16	0.010	COG0590 [F,J]	Cytosine/adenosine deaminases

Table S 81: Module 119 – with rank 24 according to estimated information content.

Rank	Probability	OG	Description
1*	0.226	COG0477 [G,E,P,R]	Permeases of the major facilitator superfamily
2*	0.120	COG0438 [M]	Glycosyltransferase
3*	0.084	COG0697 [G,E,R]	Permeases of the drug/metabolite transporter (DMT) superfamily
4	0.044	COG0730 [R]	Predicted permeases
5	0.029	COG0350 [L]	Methylated DNA-protein cysteine methyltransferase
6	0.024	COG0239 [D]	Integral membrane protein possibly involved in chromosome condensation
7*	0.023	COG0665 [E]	Glycine/D-amino acid oxidases (deaminating)
8*	0.021	COG0252 [E,J]	L-asparaginase/archaeal Glu-tRNAGln amidotransferase subunit D
9*	0.020	COG0385 [R]	Predicted Na+-dependent transporter
10*	0.019	COG0179 [Q]	2-keto-4-pentenoate hydratase/2-oxohepta-3-ene-1,7-dioic acid hydratase (catechol pathway)
11*	0.019	COG0517 [R]	FOG: CBS domain
12*	0.018	COG0119 [E]	Isopropylmalate/homocitrate/citramalate synthases
13*	0.017	COG0334 [E]	Glutamate dehydrogenase/leucine dehydrogenase
14*	0.017	COG1011 [R]	Predicted hydrolase (HAD superfamily)
15*	0.016	COG0526 [O,C]	Thiol-disulfide isomerase and thioredoxins
16*	0.015	COG1304 [C]	L-lactate dehydrogenase (FMN-dependent) and related alpha-hydroxy acid dehydrogenases
17	0.015	COG0061 [G]	Predicted sugar kinase
18	0.014	COG2855 [S]	Predicted membrane protein
19*	0.013	COG0489 [D]	ATPases involved in chromosome partitioning
20	0.012	COG2032 [P]	Cu/Zn superoxide dismutase
21*	0.012	COG0842 [V]	ABC-type multidrug transport system, permease component
22	0.012	COG4988 [C,O]	ABC-type transport system involved in cytochrome bd biosynthesis, ATPase and permease components
23	0.011	COG0668 [M]	Small-conductance mechanosensitive channel

Table S 82: Module 101 – with rank 25 according to estimated information content.

Rank	Probability	OG	Description
1*	0.330	COG3666 [L]	Transposase and inactivated derivatives
2*	0.076	COG0745 [T,K]	Response regulators consisting of a CheY-like receiver domain and a winged-helix DNA-binding domain
3	0.054	COG1961 [L]	Site-specific recombinases, DNA invertase Pin homologs
4*	0.034	COG0577 [V]	ABC-type antimicrobial peptide transport system, permease component
5*	0.029	COG1131 [V]	ABC-type multidrug transport system, ATPase component
6*	0.027	COG2217 [P]	Cation transport ATPase
7*	0.024	COG0842 [V]	ABC-type multidrug transport system, permease component
8*	0.024	COG0640 [K]	Predicted transcriptional regulators
9*	0.021	COG1902 [C]	NADH:flavin oxidoreductases, Old Yellow Enzyme family
10*	0.021	COG0318 [I,Q]	Acyl-CoA synthetases (AMP-forming)/AMP-acid ligases II
11*	0.019	COG0500 [Q,R]	SAM-dependent methyltransferases
12*	0.018	COG0210 [L]	Superfamily I DNA and RNA helicases
13	0.014	COG0542 [O]	ATPases with chaperone activity, ATP-binding subunit
14	0.014	COG0546 [R]	Predicted phosphatases
15*	0.014	COG1012 [C]	NAD-dependent aldehyde dehydrogenases
16*	0.012	COG0334 [E]	Glutamate dehydrogenase/leucine dehydrogenase
17*	0.012	COG0248 [F,P]	Exopolyphosphatase

Table S 83: Module 72 – with rank 26 according to estimated information content.

Rank	Probability	OG	Description
1*	0.108	COG0477 [G,E,P,R]	Permeases of the major facilitator superfamily
2*	0.094	COG1309 [K]	Transcriptional regulator
3*	0.090	COG0583 [K]	Transcriptional regulator
4*	0.064	COG0596 [R]	Predicted hydrolases or acyltransferases (alpha/beta hydrolase superfamily)
5*	0.056	COG0454 [K,R]	Histone acetyltransferase HPA2 and related acetyltransferases
6*	0.054	COG1028 [I,Q,R]	Dehydrogenases with different specificities (related to short-chain alcohol dehydrogenases)
7*	0.046	COG1396 [K]	Predicted transcriptional regulators
8*	0.046	COG2207 [K]	AraC-type DNA-binding domain-containing proteins
9*	0.038	COG0346 [E]	Lactoylglutathione lyase and related lyases
10	0.032	COG1733 [K]	Predicted transcriptional regulators
11*	0.028	COG1846 [K]	Transcriptional regulators
12*	0.026	COG1670 [J]	Acetyltransferases, including N-acetylases of ribosomal proteins
13*	0.020	COG0640 [K]	Predicted transcriptional regulators
14*	0.019	COG0667 [C]	Predicted oxidoreductases (related to aryl-alcohol dehydrogenases)
15*	0.017	COG1335 [Q]	Amidases related to nicotinamidase
16*	0.016	COG1063 [E,R]	Threonine dehydrogenase and related Zn-dependent dehydrogenases
17*	0.015	COG1167 [K,E]	Transcriptional regulators containing a DNA-binding HTH domain and an aminotransferase domain (MocR family) and their eukaryotic orthologs
18*	0.015	COG0463 [M]	Glycosyltransferases involved in cell wall biogenesis
19*	0.013	COG0730 [R]	Predicted permeases
20*	0.012	COG2814 [G]	Arabinose efflux permease
21*	0.011	COG0693 [R]	Putative intracellular protease/amidase
22*	0.011	COG0531 [E]	Amino acid transporters

Table S 84: Module 89 – with rank 27 according to estimated information content.

Rank	Probability	OG	Description
1*	0.162	COG0463 [M]	Glycosyltransferases involved in cell wall biogenesis
2*	0.096	COG0438 [M]	Glycosyltransferase
3*	0.052	COG2217 [P]	Cation transport ATPase
4*	0.051	COG1091 [M]	dTDP-4-dehydrorhamnose reductase
5*	0.047	COG1209 [M]	dTDP-glucose pyrophosphorylase
6*	0.046	COG1898 [M]	dTDP-4-dehydrorhamnose 3,5-epimerase and related enzymes
7	0.044	COG0494 [L,R]	NTP pyrophosphohydrolases including oxidative damage repair enzymes
8*	0.037	COG1088 [M]	dTDP-D-glucose 4,6-dehydratase
9	0.035	COG1192 [D]	ATPases involved in chromosome partitioning
10*	0.032	COG0586 [S]	Uncharacterized membrane-associated protein
11*	0.032	COG1682 [G,M]	ABC-type polysaccharide/polyol phosphate export systems, permease component
12*	0.031	COG1216 [R]	Predicted glycosyltransferases
13*	0.031	COG0489 [D]	ATPases involved in chromosome partitioning
14*	0.029	COG1134 [G,M]	ABC-type polysaccharide/polyol phosphate transport system, ATPase component
15	0.026	COG0708 [L]	Exonuclease III
16*	0.022	COG0248 [F,P]	Exopolyphosphatase
17*	0.020	COG0436 [E]	Aspartate/tyrosine/aromatic aminotransferase
18*	0.018	COG0469 [G]	Pyruvate kinase
19*	0.015	COG0640 [K]	Predicted transcriptional regulators
20	0.011	COG3049 [M]	Penicillin V acylase and related amidases
21*	0.010	COG1087 [M]	UDP-glucose 4-epimerase

Table S 85: Module 108 – with rank 28 according to estimated information content.

Rank	Probability	OG	Description
1*	0.151	COG0582 [L]	Integrase
2	0.125	COG3316 [L]	Transposase and inactivated derivatives
3*	0.093	COG1961 [L]	Site-specific recombinases, DNA invertase Pin homologs
4*	0.072	COG1028 [I,Q,R]	Dehydrogenases with different specificities (related to short-chain alcohol dehydrogenases)
5*	0.041	COG0640 [K]	Predicted transcriptional regulators
6*	0.041	COG2217 [P]	Cation transport ATPase
7*	0.039	COG1309 [K]	Transcriptional regulator
8*	0.038	COG0500 [Q,R]	SAM-dependent methyltransferases
9*	0.024	COG0454 [K,R]	Histone acetyltransferase HPA2 and related acetyltransferases
10	0.020	COG1473 [R]	Metal-dependent amidase/aminoacylase/carboxypeptidase
11*	0.019	COG0589 [T]	Universal stress protein UspA and related nucleotide-binding proteins
12	0.016	COG1475 [K]	Predicted transcriptional regulators
13	0.015	COG0507 [L]	ATP-dependent exoDNAse (exonuclease V), alpha subunit - helicase superfamily I member
14*	0.014	COG2030 [I]	Acyl dehydratase
15*	0.012	COG0110 [R]	Acetyltransferase (isoleucine patch superfamily)
16*	0.012	COG0206 [D]	Cell division GTPase
17*	0.011	COG1409 [R]	Predicted phosphohydrolases
18*	0.011	COG1210 [M]	UDP-glucose pyrophosphorylase

Table S 86: Module 59 – with rank 30 according to estimated information content.

Rank	Probability	OG	Description
1*	0.112	COG1309 [K]	Transcriptional regulator
2*	0.088	COG0640 [K]	Predicted transcriptional regulators
3*	0.066	COG0842 [V]	ABC-type multidrug transport system, permease component
4*	0.062	COG0500 [Q,R]	SAM-dependent methyltransferases
5*	0.049	COG0463 [M]	Glycosyltransferases involved in cell wall biogenesis
6*	0.047	COG1396 [K]	Predicted transcriptional regulators
7	0.037	COG1073 [R]	Hydrolases of the alpha/beta superfamily
8*	0.036	COG1131 [V]	ABC-type multidrug transport system, ATPase component
9*	0.034	COG1011 [R]	Predicted hydrolase (HAD superfamily)
10	0.032	COG0628 [R]	Predicted permease
11*	0.030	COG1454 [C]	Alcohol dehydrogenase, class IV
12*	0.028	COG1063 [E,R]	Threonine dehydrogenase and related Zn-dependent dehydrogenases
13*	0.027	COG1853 [R]	Conserved protein/domain typically associated with flavoprotein oxygenases, DIM6/NTAB family
14*	0.025	COG0607 [P]	Rhodanese-related sulfurtransferase
15*	0.023	COG0451 [M,G]	Nucleoside-diphosphate-sugar epimerases
16*	0.022	COG1902 [C]	NADH:flavin oxidoreductases, Old Yellow Enzyme family
17*	0.021	COG0726 [G]	Predicted xylanase/chitin deacetylase
18*	0.019	COG0520 [E]	Selenocysteine lyase
19*	0.016	COG1917 [S]	Uncharacterized conserved protein, contains double-stranded beta-helix domain
20*	0.016	COG0394 [T]	Protein-tyrosine-phosphatase
21*	0.013	COG0078 [E]	Ornithine carbamoyltransferase
22	0.013	COG0590 [F,J]	Cytosine/adenosine deaminases
23	0.012	COG0004 [P]	Ammonia permease
24*	0.011	COG2873 [E]	O-acetylhomoserine sulfhydrylase
25*	0.010	COG0489 [D]	ATPases involved in chromosome partitioning

Table S 87: Module 94 – with rank 31 according to estimated information content.

Rank	Probability	OG	Description
1*	0.287	COG0477 [G,E,P,R]	Permeases of the major facilitator superfamily
2	0.030	COG2205 [T]	Osmosensitive K+ channel histidine kinase
3	0.028	COG1271 [C]	Cytochrome bd-type quinol oxidase, subunit 1
4*	0.028	COG0693 [R]	Putative intracellular protease/amidase
5	0.027	COG2060 [P]	K+-transporting ATPase, A chain
6	0.026	COG2156 [P]	K+-transporting ATPase, c chain
7	0.026	COG2216 [P]	High-affinity K+ transport system, ATPase chain B
8	0.025	COG0388 [R]	Predicted amidohydrolase
9	0.025	COG0577 [V]	ABC-type antimicrobial peptide transport system, permease component
10	0.022	COG1488 [H]	Nicotinic acid phosphoribosyltransferase
11*	0.021	COG1502 [I]	Phosphatidylserine/phosphatidylglycerophosphate/cardiolipin synthases and related enzymes
12*	0.021	COG0586 [S]	Uncharacterized membrane-associated protein
13	0.019	COG0546 [R]	Predicted phosphatases
14	0.019	COG0039 [C]	Malate/lactate dehydrogenases
15	0.018	COG1294 [C]	Cytochrome bd-type quinol oxidase, subunit 2
16	0.018	COG0598 [P]	Mg2+ and Co2+ transporters
17*	0.017	COG2076 [P]	Membrane transporters of cations and cationic drugs
18	0.016	COG1918 [P]	Fe2+ transport system protein A
19*	0.016	COG0169 [E]	Shikimate 5-dehydrogenase
20*	0.015	COG0306 [P]	Phosphate/sulphate permeases
21	0.015	COG1253 [R]	Hemolysins and related proteins containing CBS domains
22*	0.014	COG0607 [P]	Rhodanese-related sulfurtransferase
23*	0.014	COG0671 [I]	Membrane-associated phospholipid phosphatase
24	0.013	COG0370 [P]	Fe2+ transport system protein B
25	0.012	COG0138 [F]	AICAR transformylase/IMP cyclohydrolase PurH (only IMP cyclohydrolase domain in Aful)
26	0.012	COG0073 [R]	EMAP domain
27	0.010	COG0162 [J]	Tyrosyl-tRNA synthetase
28	0.010	COG0574 [G]	Phosphoenolpyruvate synthase/pyruvate phosphate dikinase

Table S 88: Module 127 – with rank 32 according to estimated information content.

Rank	Probability	OG	Description
1*	0.104	COG0500 [Q,R]	SAM-dependent methyltransferases
2	0.070	COG0601 [E,P]	ABC-type dipeptide/oligopeptide/nickel transport systems, permease components
3*	0.059	COG0697 [G,E,R]	Permeases of the drug/metabolite transporter (DMT) superfamily
4	0.059	COG1173 [E,P]	ABC-type dipeptide/oligopeptide/nickel transport systems, permease components
5	0.051	COG0747 [E]	ABC-type dipeptide transport system, periplasmic component
6	0.041	COG0251 [J]	Putative translation initiation inhibitor, yjgF family
7	0.038	COG4608 [E]	ABC-type oligopeptide transport system, ATPase component
8*	0.036	COG0664 [T]	cAMP-binding proteins - catabolite gene activator and regulatory subunit of cAMP-dependent protein kinases
9	0.035	COG0444 [E,P]	ABC-type dipeptide/oligopeptide/nickel transport system, ATPase component
10*	0.031	COG0329 [E,M]	Dihydrodipicolinate synthase/N-acetylneuraminate lyase
11	0.029	COG0475 [P]	Kef-type K+ transport systems, membrane components
12*	0.029	COG0580 [G]	Glycerol uptake facilitator and related permeases (Major Intrinsic Protein Family)
13	0.029	COG1108 [P]	ABC-type Mn2+/Zn2+ transport systems, permease components
14	0.023	COG0226 [P]	ABC-type phosphate transport system, periplasmic component
15	0.023	COG2116 [P]	Formate/nitrite family of transporters
16*	0.022	COG1940 [K,G]	Transcriptional regulator/sugar kinase
17*	0.019	COG0402 [F,R]	Cytosine deaminase and related metal-dependent hydrolases
18	0.019	COG1121 [P]	ABC-type Mn/Zn transport systems, ATPase component
19	0.017	COG0628 [R]	Predicted permease
20*	0.016	COG0516 [F]	IMP dehydrogenase/GMP reductase
21*	0.016	COG0624 [E]	Acetylornithine deacetylase/Succinyl-diaminopimelate desuccinylase and related deacylases
22	0.016	COG2244 [R]	Membrane protein involved in the export of O-antigen and teichoic acid
23	0.015	COG0573 [P]	ABC-type phosphate transport system, permease component
24*	0.013	COG1053 [C]	Succinate dehydrogenase/fumarate reductase, flavoprotein subunit
25*	0.011	COG1309 [K]	Transcriptional regulator
26*	0.010	COG0549 [E]	Carbamate kinase

Table S 89: Module 177 – with rank 33 according to estimated information content.

Rank	Probability	OG	Description
1*	0.095	COG1263 [G]	Phosphotransferase system IIC components, glucose/maltose/N-acetylglucosamine-specific
2*	0.094	COG1264 [G]	Phosphotransferase system IIB components
3*	0.069	COG1762 [G,T]	Phosphotransferase system mannitol/fructose-specific IIA domain (Ntr-type)
4*	0.044	COG2190 [G]	Phosphotransferase system IIA components
5*	0.044	COG3711 [K]	Transcriptional antiterminator
6*	0.037	COG1299 [G]	Phosphotransferase system, fructose-specific IIC component
7*	0.031	COG1737 [K]	Transcriptional regulators
8*	0.030	COG0561 [R]	Predicted hydrolases of the HAD superfamily
9*	0.028	COG1940 [K,G]	Transcriptional regulator/sugar kinase
10*	0.027	COG2723 [G]	Beta-glucosidase/6-phospho-beta-glucosidase/beta-galactosidase
11*	0.025	COG1445 [G]	Phosphotransferase system fructose-specific component IIB
12*	0.025	COG1349 [K,G]	Transcriptional regulators of sugar metabolism
13*	0.024	COG2188 [K]	Transcriptional regulators
14*	0.022	COG1447 [G]	Phosphotransferase system cellobiose-specific component IIA
15*	0.019	COG1105 [G]	Fructose-1-phosphate kinase and related fructose-6-phosphate kinase (PfkB)
16	0.017	COG1131 [V]	ABC-type multidrug transport system, ATPase component
17*	0.016	COG1455 [G]	Phosphotransferase system cellobiose-specific component IIC
18	0.016	COG1902 [C]	NADH:flavin oxidoreductases, Old Yellow Enzyme family
19*	0.015	COG3414 [G]	Phosphotransferase system, galactitol-specific IIB component
20*	0.015	COG2376 [G]	Dihydroxyacetone kinase
21*	0.014	COG0191 [G]	Fructose/tagatose bisphosphate aldolase
22*	0.013	COG0366 [G]	Glycosidases
23	0.013	COG2116 [P]	Formate/nitrite family of transporters
24*	0.013	COG0524 [G]	Sugar kinases, ribokinase family
25	0.012	COG0580 [G]	Glycerol uptake facilitator and related permeases (Major Intrinsic Protein Family)
26*	0.011	COG0698 [G]	Ribose 5-phosphate isomerase RpiB
27	0.011	COG2235 [E]	Arginine deiminase
28	0.010	COG0053 [P]	Predicted Co/Zn/Cd cation transporters
29*	0.010	COG1621 [G]	Beta-fructosidases (levanase/invertase)

Table S 90: Module 19 – with rank 34 according to estimated information content.

Rank	Probability	OG	Description
1*	0.118	COG1529 [C]	Aerobic-type carbon monoxide dehydrogenase, large subunit CoxL/CutL homologs
2*	0.104	COG2080 [C]	Aerobic-type carbon monoxide dehydrogenase, small subunit CoxS/CutS homologs
3*	0.060	COG0624 [E]	Acetylornithine deacetylase/Succinyl-diaminopimelate desuccinylase and related deacylases
4*	0.058	COG1319 [C]	Aerobic-type carbon monoxide dehydrogenase, middle subunit CoxM/CutM homologs
5*	0.053	COG0500 [Q,R]	SAM-dependent methyltransferases
6*	0.051	COG1975 [O]	Xanthine and CO dehydrogenases maturation factor, XdhC/CoxF family
7*	0.037	COG0329 [E,M]	Dihydrodipicolinate synthase/N-acetylneuraminate lyase
8*	0.033	COG0388 [R]	Predicted amidohydrolase
9*	0.030	COG2068 [R]	Uncharacterized MobA-related protein
10*	0.027	COG0251 [J]	Putative translation initiation inhibitor, yjgF family
11*	0.026	COG1146 [C]	Ferredoxin
12*	0.025	COG1215 [M]	Glycosyltransferases, probably involved in cell wall biogenesis
13*	0.024	COG2897 [P]	Rhodanese-related sulfurtransferase
14*	0.024	COG0473 [C,E]	Isocitrate/isopropylmalate dehydrogenase
15*	0.023	COG1171 [E]	Threonine dehydratase
16*	0.017	COG0044 [F]	Dihydroorotase and related cyclic amidohydrolases
17*	0.015	COG0517 [R]	FOG: CBS domain
18*	0.014	COG0350 [L]	Methylated DNA-protein cysteine methyltransferase
19*	0.013	COG1788 [I]	Acyl CoA:acetate/3-ketoacid CoA transferase, alpha subunit
20*	0.011	COG1804 [C]	Predicted acyl-CoA transferases/carnitine dehydratase
21	0.010	COG0614 [P]	ABC-type Fe3+-hydroxamate transport system, periplasmic component
22	0.010	COG1937 [S]	Uncharacterized protein conserved in bacteria

Table S 91: Module 143 – with rank 35 according to estimated information content.

Rank	Probability	OG	Description
1*	0.266	COG0583 [K]	Transcriptional regulator
2*	0.074	COG2207 [K]	AraC-type DNA-binding domain-containing proteins
3*	0.032	COG1280 [E]	Putative threonine efflux protein
4*	0.028	COG0697 [G,E,R]	Permeases of the drug/metabolite transporter (DMT) superfamily
5*	0.025	COG1902 [C]	NADH:flavin oxidoreductases, Old Yellow Enzyme family
6*	0.021	COG2199 [T]	FOG: GGDEF domain
7*	0.019	COG0251 [J]	Putative translation initiation inhibitor, yjgF family
8*	0.017	COG1566 [V]	Multidrug resistance efflux pump
9*	0.016	COG1028 [I,Q,R]	Dehydrogenases with different specificities (related to short-chain alcohol dehydrogenases)
10*	0.016	COG0625 [O]	Glutathione S-transferase
11*	0.015	COG2771 [K]	DNA-binding HTH domain-containing proteins
12*	0.014	COG1522 [K]	Transcriptional regulators
13*	0.013	COG0454 [K,R]	Histone acetyltransferase HPA2 and related acetyltransferases
14	0.013	COG1840 [P]	ABC-type Fe3+ transport system, periplasmic component
15	0.011	COG0523 [R]	Putative GTPases (G3E family)

Table S 92: Module 150 – with rank 38 according to estimated information content.

Rank	Probability	OG	Description
1*	0.062	COG0477 [G,E,P,R]	Permeases of the major facilitator superfamily
2*	0.056	COG1132 [V]	ABC-type multidrug transport system, ATPase and permease components
3*	0.048	COG0831 [E]	Urea amidohydrolase (urease) gamma subunit
4*	0.046	COG0378 [O,K]	Ni2+-binding GTPase involved in regulation of expression and maturation of urease and hydrogenase
5*	0.046	COG0804 [E]	Urea amidohydrolase (urease) alpha subunit
6*	0.045	COG0832 [E]	Urea amidohydrolase (urease) beta subunit
7*	0.045	COG0830 [O]	Urease accessory protein UreF
8*	0.040	COG0829 [O]	Urease accessory protein UreH
9*	0.037	COG2217 [P]	Cation transport ATPase
10*	0.033	COG2371 [O]	Urease accessory protein UreE
11*	0.033	COG0842 [V]	ABC-type multidrug transport system, permease component
12*	0.030	COG0492 [O]	Thioredoxin reductase
13*	0.029	COG4674 [R]	Uncharacterized ABC-type transport system, ATPase component
14*	0.029	COG0665 [E]	Glycine/D-amino acid oxidases (deaminating)
15*	0.027	COG0436 [E]	Aspartate/tyrosine/aromatic aminotransferase
16	0.023	COG1253 [R]	Hemolysins and related proteins containing CBS domains
17*	0.020	COG0346 [E]	Lactoylglutathione lyase and related lyases
18	0.020	COG0042 [J]	tRNA-dihydrouridine synthase
19*	0.018	COG0547 [E]	Anthranilate phosphoribosyltransferase
20	0.018	COG0523 [R]	Putative GTPases (G3E family)
21	0.017	COG0582 [L]	Integrase
22*	0.015	COG0010 [E]	Arginase/agmatinase/formimionoglutamate hydrolase, arginase family
23*	0.015	COG1045 [E]	Serine acetyltransferase
24*	0.015	COG0329 [E,M]	Dihydrodipicolinate synthase/N-acetylneuraminate lyase
25*	0.013	COG0788 [F]	Formyltetrahydrofolate hydrolase
26*	0.013	COG0057 [G]	Glyceraldehyde-3-phosphate dehydrogenase/erythrose-4-phosphate dehydrogenase
27*	0.012	COG0212 [H]	5-formyltetrahydrofolate cyclo-ligase
28*	0.011	COG0439 [I]	Biotin carboxylase
29*	0.011	COG0520 [E]	Selenocysteine lyase
30*	0.010	COG0115 [E,H]	Branched-chain amino acid aminotransferase/4-amino-4-deoxychorismate lyase
31*	0.010	COG0443 [O]	Molecular chaperone

Table S 93: Module 39 – with rank 39 according to estimated information content.

Rank	Probability	OG	Description
1	0.080	COG3639 [P]	ABC-type phosphate/phosphonate transport system, permease component
2*	0.079	COG0596 [R]	Predicted hydrolases or acyltransferases (alpha/beta hydrolase superfamily)
3*	0.059	COG1028 [I,Q,R]	Dehydrogenases with different specificities (related to short-chain alcohol dehydrogenases)
4	0.056	COG3221 [P]	ABC-type phosphate/phosphonate transport system, periplasmic component
5*	0.053	COG0454 [K,R]	Histone acetyltransferase HPA2 and related acetyltransferases
6	0.051	COG3638 [P]	ABC-type phosphate/phosphonate transport system, ATPase component
7*	0.049	COG1309 [K]	Transcriptional regulator
8*	0.038	COG0778 [C]	Nitroreductase
9	0.026	COG3454 [P]	Metal-dependent hydrolase involved in phosphonate metabolism
10*	0.025	COG0697 [G,E,R]	Permeases of the drug/metabolite transporter (DMT) superfamily
11*	0.025	COG0277 [C]	FAD/FMN-containing dehydrogenases
12*	0.023	COG1230 [P]	Co/Zn/Cd efflux system component
13	0.023	COG4107 [P]	ABC-type phosphonate transport system, ATPase component
14	0.022	COG3626 [P]	Uncharacterized enzyme of phosphonate metabolism
15	0.022	COG3627 [P]	Uncharacterized enzyme of phosphonate metabolism
16	0.022	COG4778 [P]	ABC-type phosphonate transport system, ATPase component
17	0.021	COG3709 [P]	Uncharacterized component of phosphonate metabolism
18	0.020	COG3625 [P]	Uncharacterized enzyme of phosphonate metabolism
19	0.018	COG3624 [P]	Uncharacterized enzyme of phosphonate metabolism
20*	0.017	COG0584 [C]	Glycerophosphoryl diester phosphodiesterase
21*	0.017	COG0446 [R]	Uncharacterized NAD(FAD)-dependent dehydrogenases
22	0.016	COG1235 [R]	Metal-dependent hydrolases of the beta-lactamase superfamily I
23	0.014	COG1942 [R]	Uncharacterized protein, 4-oxalocrotonate tautomerase homolog
24*	0.012	COG0450 [O]	Peroxiredoxin
25	0.011	COG1544 [J]	Ribosome-associated protein Y (PSrp-1)
26*	0.010	COG1680 [V]	Beta-lactamase class C and other penicillin binding proteins

Table S 94: Module 33 – with rank 40 according to estimated information content.

Rank	Probability	OG	Description
1*	0.089	COG0589 [T]	Universal stress protein UspA and related nucleotide-binding proteins
2*	0.065	COG0842 [V]	ABC-type multidrug transport system, permease component
3*	0.044	COG1132 [V]	ABC-type multidrug transport system, ATPase and permease components
4*	0.040	COG0346 [E]	Lactoylglutathione lyase and related lyases
5*	0.039	COG0318 [I,Q]	Acyl-CoA synthetases (AMP-forming)/AMP-acid ligases II
6*	0.039	COG0446 [R]	Uncharacterized NAD(FAD)-dependent dehydrogenases
7*	0.039	COG0500 [Q,R]	SAM-dependent methyltransferases
8*	0.036	COG0119 [E]	Isopropylmalate/homocitrate/citramalate synthases
9*	0.034	COG0517 [R]	FOG: CBS domain
10*	0.033	COG1063 [E,R]	Threonine dehydrogenase and related Zn-dependent dehydrogenases
11*	0.027	COG0394 [T]	Protein-tyrosine-phosphatase
12*	0.027	COG0071 [O]	Molecular chaperone (small heat shock protein)
13*	0.025	COG0693 [R]	Putative intracellular protease/amidase
14*	0.025	COG1131 [V]	ABC-type multidrug transport system, ATPase component
15	0.025	COG0389 [L]	Nucleotidyltransferase/DNA polymerase involved in DNA repair
16*	0.023	COG1252 [C]	NADH dehydrogenase, FAD-containing subunit
17*	0.023	COG0574 [G]	Phosphoenolpyruvate synthase/pyruvate phosphate dikinase
18*	0.020	COG0511 [I]	Biotin carboxyl carrier protein
19*	0.019	COG0179 [Q]	2-keto-4-pentenoate hydratase/2-oxohepta-3-ene-1,7-dioic acid hydratase (catechol pathway)
20*	0.019	COG0578 [C]	Glycerol-3-phosphate dehydrogenase
21*	0.017	COG0655 [R]	Multimeric flavodoxin WrbA
22*	0.016	COG1249 [C]	Pyruvate/2-oxoglutarate dehydrogenase complex, dihydrolipoamide dehydrogenase (E3) component, and related enzymes
23*	0.016	COG2226 [H]	Methylase involved in ubiquinone/menaquinone biosynthesis
24*	0.015	COG0439 [I]	Biotin carboxylase
25	0.014	COG0433 [R]	Predicted ATPase
26*	0.013	COG0142 [H]	Geranylgeranyl pyrophosphate synthase
27*	0.013	COG0404 [E]	Glycine cleavage system T protein (aminomethyltransferase)
28*	0.012	COG0174 [E]	Glutamine synthetase
29	0.012	COG2220 [R]	Predicted Zn-dependent hydrolases of the beta-lactamase fold
30	0.011	COG0590 [F,J]	Cytosine/adenosine deaminases

Table S 95: Module 173 – with rank 41 according to estimated information content.

Rank	Probability	OG	Description
1*	0.073	COG0517 [R]	FOG: CBS domain
2*	0.070	COG0500 [Q,R]	SAM-dependent methyltransferases
3*	0.054	COG0491 [R]	Zn-dependent hydrolases, including glyoxylases
4*	0.039	COG0640 [K]	Predicted transcriptional regulators
5*	0.036	COG1109 [G]	Phosphomannomutase
6*	0.033	COG0574 [G]	Phosphoenolpyruvate synthase/pyruvate phosphate dikinase
7*	0.030	COG0456 [R]	Acetyltransferases
8*	0.025	COG0778 [C]	Nitroreductase
9*	0.025	COG0451 [M,G]	Nucleoside-diphosphate-sugar epimerases
10*	0.024	COG0111 [H,E]	Phosphoglycerate dehydrogenase and related dehydrogenases
11*	0.023	COG0524 [G]	Sugar kinases, ribokinase family
12*	0.023	COG0518 [F]	GMP synthase - Glutamine amidotransferase domain
13*	0.022	COG0061 [G]	Predicted sugar kinase
14	0.022	COG1985 [H]	Pyrimidine reductase, riboflavin biosynthesis
15*	0.022	COG0136 [E]	Aspartate-semialdehyde dehydrogenase
16*	0.021	COG0174 [E]	Glutamine synthetase
17*	0.020	COG0152 [F]	Phosphoribosylaminoimidazolesuccinocarboxamide (SAICAR) synthase
18*	0.020	COG0008 [J]	Glutamyl- and glutaminyl-tRNA synthetases
19	0.019	COG0167 [F]	Dihydroorotate dehydrogenase
20*	0.019	COG0294 [H]	Dihydropteroate synthase and related enzymes
21*	0.019	COG0452 [H]	Phosphopantothenoylcysteine synthetase/decarboxylase
22*	0.018	COG0006 [E]	Xaa-Pro aminopeptidase
23*	0.018	COG2217 [P]	Cation transport ATPase
24*	0.018	COG1648 [H]	Siroheme synthase (precorrin-2 oxidase/ferrochelatase domain)
25*	0.017	COG0047 [F]	Phosphoribosylformylglycinamidine (FGAM) synthase, glutamine amidotransferase domain
26*	0.017	COG0113 [H]	Delta-aminolevulinic acid dehydratase
27	0.017	COG0053 [P]	Predicted Co/Zn/Cd cation transporters
28*	0.017	COG0422 [H]	Thiamine biosynthesis protein ThiC
29*	0.016	COG0519 [F]	GMP synthase, PP-ATPase domain/subunit
30*	0.014	COG0041 [F]	Phosphoribosylcarboxyaminoimidazole (NCAIR) mutase
31	0.013	COG0157 [H]	Nicotinate-nucleotide pyrophosphorylase
32*	0.013	COG0476 [H]	Dinucleotide-utilizing enzymes involved in molybdopterin and thiamine biosynthesis family 2
33*	0.012	COG0001 [H]	Glutamate-1-semialdehyde aminotransferase
34*	0.011	COG0034 [F]	Glutamine phosphoribosylpyrophosphate amidotransferase
35	0.011	COG0379 [H]	Quinolinate synthase
36*	0.010	COG1587 [H]	Uroporphyrinogen-III synthase

Table S 96: Module 84 – with rank 42 according to estimated information content.

Rank	Probability	OG	Description
1*	0.168	COG1028 [I,Q,R]	Dehydrogenases with different specificities (related to short-chain alcohol dehydrogenases)
2*	0.069	COG2141 [C]	Coenzyme F420-dependent N5,N10-methylene tetrahydromethanopterin reductase and related flavin-dependent oxidoreductases
3*	0.045	COG1802 [K]	Transcriptional regulators
4*	0.032	COG0715 [P]	ABC-type nitrate/sulfonate/bicarbonate transport systems, periplasmic components
5*	0.030	COG0154 [J]	Asp-tRNAAsn/Glu-tRNAGln amidotransferase A subunit and related amidases
6*	0.030	COG0600 [P]	ABC-type nitrate/sulfonate/bicarbonate transport system, permease component
7*	0.028	COG0702 [M,G]	Predicted nucleoside-diphosphate-sugar epimerases
8*	0.027	COG0604 [C,R]	NADPH:quinone reductase and related Zn-dependent oxidoreductases
9*	0.027	COG1116 [P]	ABC-type nitrate/sulfonate/bicarbonate transport system, ATPase component
10*	0.021	COG0667 [C]	Predicted oxidoreductases (related to aryl-alcohol dehydrogenases)
11*	0.021	COG0491 [R]	Zn-dependent hydrolases, including glyoxylases
12	0.020	COG1846 [K]	Transcriptional regulators
13*	0.020	COG3386 [G]	Gluconolactonase
14*	0.020	COG1853 [R]	Conserved protein/domain typically associated with flavoprotein oxygenases, DIM6/NTAB family
15*	0.018	COG0438 [M]	Glycosyltransferase
16	0.017	COG1123 [R]	ATPase components of various ABC-type transport systems, contain duplicated ATPase
17*	0.017	COG1835 [I]	Predicted acyltransferases
18*	0.015	COG1064 [R]	Zn-dependent alcohol dehydrogenases
19*	0.015	COG0111 [H,E]	Phosphoglycerate dehydrogenase and related dehydrogenases
20*	0.013	COG4221 [R]	Short-chain alcohol dehydrogenase of unknown specificity
21*	0.012	COG0657 [I]	Esterase/lipase
22*	0.010	COG0300 [R]	Short-chain dehydrogenases of various substrate specificities
23*	0.010	COG1062 [C]	Zn-dependent alcohol dehydrogenases, class III
24*	0.010	COG0262 [H]	Dihydrofolate reductase
25	0.010	COG1764 [O]	Predicted redox protein, regulator of disulfide bond formation

Table S 97: Module 129 – with rank 43 according to estimated information content.

Rank	Probability	OG	Description
1*	0.072	COG0508 [C]	Pyruvate/2-oxoglutarate dehydrogenase complex, dihydrolipoamide acyltransferase (E2) component, and related enzymes
2	0.050	COG0582 [L]	Integrase
3*	0.045	COG0022 [C]	Pyruvate/2-oxoglutarate dehydrogenase complex, dehydrogenase (E1) component, eukaryotic type, beta subunit
4*	0.045	COG1071 [C]	Pyruvate/2-oxoglutarate dehydrogenase complex, dehydrogenase (E1) component, eukaryotic type, alpha subunit
5*	0.044	COG0697 [G,E,R]	Permeases of the drug/metabolite transporter (DMT) superfamily
6*	0.030	COG0436 [E]	Aspartate/tyrosine/aromatic aminotransferase
7*	0.030	COG0500 [Q,R]	SAM-dependent methyltransferases
8*	0.028	COG0110 [R]	Acetyltransferase (isoleucine patch superfamily)
9*	0.027	COG0318 [I,Q]	Acyl-CoA synthetases (AMP-forming)/AMP-acid ligases II
10	0.026	COG0807 [H]	GTP cyclohydrolase II
11	0.025	COG1611 [R]	Predicted Rossmann fold nucleotide-binding protein
12	0.022	COG1694 [R]	Predicted pyrophosphatase
13	0.021	COG0108 [H]	3,4-dihydroxy-2-butanone 4-phosphate synthase
14*	0.020	COG0176 [G]	Transaldolase
15	0.020	COG1271 [C]	Cytochrome bd-type quinol oxidase, subunit 1
16*	0.020	COG1249 [C]	Pyruvate/2-oxoglutarate dehydrogenase complex, dihydrolipoamide dehydrogenase (E3) component, and related enzymes
17*	0.020	COG0511 [I]	Biotin carboxyl carrier protein
18*	0.019	COG0334 [E]	Glutamate dehydrogenase/leucine dehydrogenase
19	0.019	COG0307 [H]	Riboflavin synthase alpha chain
20*	0.019	COG0450 [O]	Peroxiredoxin
21	0.018	COG0352 [H]	Thiamine monophosphate synthase
22	0.018	COG0117 [H]	Pyrimidine deaminase
23*	0.018	COG0329 [E,M]	Dihydrodipicolinate synthase/N-acetylneuraminate lyase
24	0.017	COG1985 [H]	Pyrimidine reductase, riboflavin biosynthesis
25*	0.017	COG0509 [E]	Glycine cleavage system H protein (lipoate-binding)
26	0.017	COG1502 [I]	Phosphatidylserine/phosphatidylglycerophosphate/cardiolipin synthases and related enzymes
27*	0.016	COG0502 [H]	Biotin synthase and related enzymes
28	0.016	COG0471 [P]	Di- and tricarboxylate transporters

29	0.016	COG1294 [C]	Cytochrome bd-type quinol oxidase, subunit 2
30	0.016	COG0054 [H]	Riboflavin synthase beta-chain
31*	0.015	COG1607 [I]	Acyl-CoA hydrolase
32*	0.013	COG0527 [E]	Aspartokinases
33*	0.013	COG0764 [I]	3-hydroxymyristoyl/3-hydroxydecanoyl-(acyl carrier protein) dehydratases
34*	0.011	COG0095 [H]	Lipoate-protein ligase A
35*	0.010	COG0350 [L]	Methylated DNA-protein cysteine methyltransferase

Table S 98: Module 35 – with rank 47 according to estimated information content.

Rank	Probability	OG	Description
1	0.050	COG4603 [R]	ABC-type uncharacterized transport system, permease component
2	0.047	COG3845 [R]	ABC-type uncharacterized transport systems, ATPase components
3	0.046	COG0526 [O,C]	Thiol-disulfide isomerase and thioredoxins
4	0.044	COG1744 [R]	Uncharacterized ABC-type transport system, periplasmic component/surface lipoprotein
5*	0.040	COG0444 [E,P]	ABC-type dipeptide/oligopeptide/nickel transport system, ATPase component
6	0.038	COG1079 [R]	Uncharacterized ABC-type transport system, permease component
7*	0.038	COG1173 [E,P]	ABC-type dipeptide/oligopeptide/nickel transport systems, permease components
8*	0.035	COG0601 [E,P]	ABC-type dipeptide/oligopeptide/nickel transport systems, permease components
9	0.033	COG3842 [E]	ABC-type spermidine/putrescine transport systems, ATPase components
10	0.030	COG1131 [V]	ABC-type multidrug transport system, ATPase component
11	0.029	COG1122 [P]	ABC-type cobalt transport system, ATPase component
12	0.024	COG0569 [P]	K+ transport systems, NAD-binding component
13	0.024	COG0573 [P]	ABC-type phosphate transport system, permease component
14*	0.023	COG0035 [F]	Uracil phosphoribosyltransferase
15	0.023	COG0581 [P]	ABC-type phosphate transport system, permease component
16	0.023	COG1177 [E]	ABC-type spermidine/putrescine transport system, permease component II
17	0.023	COG0171 [H]	NAD synthase
18*	0.023	COG0563 [F]	Adenylate kinase and related kinases
19	0.022	COG1176 [E]	ABC-type spermidine/putrescine transport system, permease component I
20	0.022	COG0584 [C]	Glycerophosphoryl diester phosphodiesterase
21	0.021	COG0168 [P]	Trk-type K+ transport systems, membrane components
22	0.020	COG0226 [P]	ABC-type phosphate transport system, periplasmic component
23	0.019	COG0704 [P]	Phosphate uptake regulator
24	0.019	COG0554 [C]	Glycerol kinase
25*	0.018	COG0006 [E]	Xaa-Pro aminopeptidase
26	0.018	COG0492 [O]	Thioredoxin reductase
27*	0.017	COG0295 [F]	Cytidine deaminase
28	0.017	COG0619 [P]	ABC-type cobalt transport system, permease component CbiQ and related transporters

29	0.016	COG1117 [P]	ABC-type phosphate transport system, ATPase component
30*	0.016	COG4608 [E]	ABC-type oligopeptide transport system, ATPase component
31*	0.015	COG0503 [F]	Adenine/guanine phosphoribosyltransferases and related PRPP-binding proteins
32*	0.015	COG1210 [M]	UDP-glucose pyrophosphorylase
33	0.015	COG0687 [E]	Spermidine/putrescine-binding periplasmic protein
34*	0.011	COG1404 [O]	Subtilisin-like serine proteases

Table S 99: Module 106 – with rank 48 according to estimated information content.

Rank	Probability	OG	Description
1*	0.120	COG0454 [K,R]	Histone acetyltransferase HPA2 and related acetyltransferases
2*	0.082	COG1670 [J]	Acetyltransferases, including N-acetylases of ribosomal proteins
3*	0.039	COG1835 [I]	Predicted acyltransferases
4*	0.038	COG2217 [P]	Cation transport ATPase
5*	0.037	COG1680 [V]	Beta-lactamase class C and other penicillin binding proteins
6*	0.030	COG1249 [C]	Pyruvate/2-oxoglutarate dehydrogenase complex, dihydrolipoamide dehydrogenase (E3) component, and related enzymes
7*	0.027	COG0262 [H]	Dihydrofolate reductase
8*	0.027	COG0488 [R]	ATPase components of ABC transporters with duplicated ATPase domains
9*	0.023	COG0332 [I]	3-oxoacyl-[acyl-carrier-protein] synthase III
10	0.022	COG1733 [K]	Predicted transcriptional regulators
11*	0.021	COG1132 [V]	ABC-type multidrug transport system, ATPase and permease components
12*	0.021	COG1357 [S]	Uncharacterized low-complexity proteins
13	0.018	COG1359 [S]	Uncharacterized conserved protein
14	0.017	COG0389 [L]	Nucleotidyltransferase/DNA polymerase involved in DNA repair
15*	0.017	COG0251 [J]	Putative translation initiation inhibitor, yjgF family
16*	0.016	COG0346 [E]	Lactoylglutathione lyase and related lyases
17*	0.016	COG0076 [E]	Glutamate decarboxylase and related PLP-dependent proteins
18*	0.016	COG0281 [C]	Malic enzyme
19*	0.015	COG1073 [R]	Hydrolases of the alpha/beta superfamily
20	0.015	COG2957 [E]	Peptidylarginine deiminase and related enzymes
21	0.014	COG0350 [L]	Methylated DNA-protein cysteine methyltransferase
22*	0.014	COG0366 [G]	Glycosidases
23	0.014	COG0598 [P]	Mg2+ and Co2+ transporters
24	0.014	COG1228 [Q]	Imidazolonepropionase and related amidohydrolases
25*	0.013	COG1597 [I,R]	Sphingosine kinase and enzymes related to eukaryotic diacylglycerol kinase
26*	0.013	COG0384 [R]	Predicted epimerase, PhzC/PhzF homolog
27	0.012	COG1253 [R]	Hemolysins and related proteins containing CBS domains
28*	0.011	COG0439 [I]	Biotin carboxylase
29	0.010	COG0475 [P]	Kef-type K+ transport systems, membrane components

Table S 100: Module 61 – with rank 49 according to estimated information content.

Rank	Probability	OG	Description
1	0.128	COG0745 [T,K]	Response regulators consisting of a CheY-like receiver domain and a winged-helix DNA-binding domain
2	0.110	COG0642 [T]	Signal transduction histidine kinase
3*	0.032	COG0680 [C]	Ni,Fe-hydrogenase maturation factor
4*	0.031	COG0374 [C]	Ni,Fe-hydrogenase I large subunit
5*	0.031	COG1740 [C]	Ni,Fe-hydrogenase I small subunit
6	0.030	COG0526 [O,C]	Thiol-disulfide isomerase and thioredoxins
7*	0.028	COG0378 [O,K]	Ni2+-binding GTPase involved in regulation of expression and maturation of urease and hydrogenase
8*	0.027	COG0068 [O]	Hydrogenase maturation factor
9*	0.026	COG0309 [O]	Hydrogenase maturation factor
10*	0.026	COG0409 [O]	Hydrogenase maturation factor
11*	0.025	COG0375 [R]	Zn finger protein HypA/HybF (possibly regulating hydrogenase expression)
12*	0.024	COG0298 [O]	Hydrogenase maturation factor
13	0.023	COG2217 [P]	Cation transport ATPase
14*	0.021	COG1145 [C]	Ferredoxin
15	0.019	COG0119 [E]	Isopropylmalate/homocitrate/citramalate synthases
16	0.018	COG2896 [H]	Molybdenum cofactor biosynthesis enzyme
17	0.017	COG0789 [K]	Predicted transcriptional regulators
18	0.015	COG1055 [P]	Na+/H+ antiporter NhaD and related arsenite permeases
19*	0.013	COG1969 [C]	Ni,Fe-hydrogenase I cytochrome b subunit
20	0.012	COG2081 [R]	Predicted flavoproteins
21*	0.012	COG1034 [C]	NADH dehydrogenase/NADH:ubiquinone oxidoreductase 75 kD subunit (chain G)
22	0.011	COG0465 [O]	ATP-dependent Zn proteases
23	0.011	COG0057 [G]	Glyceraldehyde-3-phosphate dehydrogenase/erythrose-4-phosphate dehydrogenase
24	0.011	COG0598 [P]	Mg2+ and Co2+ transporters
25*	0.010	COG0493 [E,R]	NADPH-dependent glutamate synthase beta chain and related oxidoreductases

Table S 101: Module 179 – with rank 50 according to estimated information content.

Rank	Probability	OG	Description
1	0.161	COG0582 [L]	Integrase
2	0.048	COG0745 [T,K]	Response regulators consisting of a CheY-like receiver domain and a winged-helix DNA-binding domain
3*	0.042	COG4626 [R]	Phage terminase-like protein, large subunit
4*	0.040	COG4695 [S]	Phage-related protein
5	0.033	COG3617 [K]	Prophage antirepressor
6	0.028	COG0740 [O,U]	Protease subunit of ATP-dependent Clp proteases
7*	0.028	COG1403 [V]	Restriction endonuclease
8	0.028	COG1396 [K]	Predicted transcriptional regulators
9	0.027	COG1961 [L]	Site-specific recombinases, DNA invertase Pin homologs
10	0.024	COG1475 [K]	Predicted transcriptional regulators
11	0.024	COG5283 [S]	Phage-related tail protein
12	0.023	COG0553 [K,L]	Superfamily II DNA/RNA helicases, SNF2 family
13	0.022	COG0642 [T]	Signal transduction histidine kinase
14	0.021	COG1783 [R]	Phage terminase large subunit
15	0.020	COG0629 [L]	Single-stranded DNA-binding protein
16	0.018	COG3378 [R]	Predicted ATPase
17	0.017	COG3561 [K]	Phage anti-repressor protein
18	0.017	COG3646 [S]	Uncharacterized phage-encoded protein
19	0.015	COG3645 [S]	Uncharacterized phage-encoded protein
20*	0.014	COG3740 [R]	Phage head maturation protease
21	0.014	COG0863 [L]	DNA modification methylase
22	0.013	COG3728 [L]	Phage terminase, small subunit
23	0.012	COG4570 [L]	Holliday junction resolvase
24*	0.012	COG4653 [R]	Predicted phage phi-C31 gp36 major capsid-like protein
25*	0.012	COG3747 [L]	Phage terminase, small subunit
26*	0.011	NOG75880 [S]	Phage protein
27	0.011	COG3723 [L]	Recombinational DNA repair protein (RecE pathway)
28	0.010	COG3299 [S]	Uncharacterized homolog of phage Mu protein gp47
29	0.010	NOG11122 [L]	DNA polymerase

Table S 102: Module 154 – with rank 51 according to estimated information content.

Rank	Probability	OG	Description
1*	0.098	COG1028 [I,Q,R]	Dehydrogenases with different specificities (related to short-chain alcohol dehydrogenases)
2*	0.051	COG0586 [S]	Uncharacterized membrane-associated protein
3*	0.044	COG0671 [I]	Membrane-associated phospholipid phosphatase
4*	0.036	COG0737 [F]	5-nucleotidase/2,3-cyclic phosphodiesterase and related esterases
5*	0.034	COG0280 [C]	Phosphotransacetylase
6*	0.031	COG0564 [J]	Pseudouridylate synthases, 23S RNA-specific
7*	0.031	COG0513 [L,K,J]	Superfamily II DNA and RNA helicases
8	0.030	COG1479 [S]	Uncharacterized conserved protein
9*	0.029	COG1502 [I]	Phosphatidylserine/phosphatidylglycerophosphate/cardiolipin synthases and related enzymes
10*	0.028	COG0329 [E,M]	Dihydrodipicolinate synthase/N-acetylneuraminate lyase
11*	0.026	COG0019 [E]	Diaminopimelate decarboxylase
12*	0.025	COG1475 [K]	Predicted transcriptional regulators
13*	0.024	COG0550 [L]	Topoisomerase IA
14*	0.023	COG1253 [R]	Hemolysins and related proteins containing CBS domains
15*	0.021	COG0187 [L]	Type IIA topoisomerase (DNA gyrase/topo II, topoisomerase IV), B subunit
16*	0.021	COG0758 [L,U]	Predicted Rossmann fold nucleotide-binding protein involved in DNA uptake
17*	0.021	COG0334 [E]	Glutamate dehydrogenase/leucine dehydrogenase
18*	0.020	COG0282 [C]	Acetate kinase
19*	0.020	COG0503 [F]	Adenine/guanine phosphoribosyltransferases and related PRPP-binding proteins
20	0.019	COG0681 [U]	Signal peptidase I
21*	0.018	COG0208 [F]	Ribonucleotide reductase, beta subunit
22*	0.018	COG1985 [H]	Pyrimidine reductase, riboflavin biosynthesis
23*	0.017	COG0472 [M]	UDP-N-acetylmuramyl pentapeptide phosphotransferase/UDP-N-acetylglucosamine-1-phosphate transferase
24*	0.016	COG1012 [C]	NAD-dependent aldehyde dehydrogenases
25*	0.015	COG0258 [L]	5-3 exonuclease (including N-terminal domain of PolI)
26	0.015	COG0358 [L]	DNA primase (bacterial type)
27*	0.014	COG0006 [E]	Xaa-Pro aminopeptidase
28	0.014	COG1741 [R]	Pirin-related protein
29*	0.013	COG1187 [J]	16S rRNA uridine-516 pseudouridylate synthase and related pseudouridylate synthases
30*	0.011	COG0015 [F]	Adenylosuccinate lyase
31*	0.011	COG2898 [S]	Uncharacterized conserved protein
32	0.010	COG1275 [P]	Tellurite resistance protein and related permeases

Table S 103: Module 111 – with rank 54 according to estimated information content.

Rank	Probability	OG	Description
1*	0.059	COG1131 [V]	ABC-type multidrug transport system, ATPase component
2*	0.050	COG0524 [G]	Sugar kinases, ribokinase family
3*	0.049	COG0842 [V]	ABC-type multidrug transport system, permease component
4	0.049	COG1112 [L]	Superfamily I DNA and RNA helicases and helicase subunits
5*	0.045	COG0436 [E]	Aspartate/tyrosine/aromatic aminotransferase
6*	0.041	COG0463 [M]	Glycosyltransferases involved in cell wall biogenesis
7	0.041	COG0714 [R]	MoxR-like ATPases
8*	0.035	COG0656 [R]	Aldo/keto reductases, related to diketogulonate reductase
9*	0.031	COG0520 [E]	Selenocysteine lyase
10*	0.030	COG1109 [G]	Phosphomannomutase
11*	0.029	COG0366 [G]	Glycosidases
12*	0.028	COG1335 [Q]	Amidases related to nicotinamidase
13*	0.025	COG0404 [E]	Glycine cleavage system T protein (aminomethyltransferase)
14*	0.024	COG0073 [R]	EMAP domain
15*	0.024	COG0209 [F]	Ribonucleotide reductase, alpha subunit
16*	0.023	COG0584 [C]	Glycerophosphoryl diester phosphodiesterase
17*	0.019	COG0403 [E]	Glycine cleavage system protein P (pyridoxal-binding), N-terminal domain
18*	0.019	COG1003 [E]	Glycine cleavage system protein P (pyridoxal-binding), C-terminal domain
19*	0.018	COG0509 [E]	Glycine cleavage system H protein (lipoate-binding)
20*	0.016	COG0018 [J]	Arginyl-tRNA synthetase
21*	0.016	COG0519 [F]	GMP synthase, PP-ATPase domain/subunit
22*	0.015	COG0685 [E]	5,10-methylenetetrahydrofolate reductase
23*	0.014	COG0057 [G]	Glyceraldehyde-3-phosphate dehydrogenase/erythrose-4-phosphate dehydrogenase
24*	0.013	COG0078 [E]	Ornithine carbamoyltransferase
25*	0.013	COG0554 [C]	Glycerol kinase
26*	0.013	COG1209 [M]	dTDP-glucose pyrophosphorylase
27*	0.011	COG0013 [J]	Alanyl-tRNA synthetase
28*	0.010	COG0110 [R]	Acetyltransferase (isoleucine patch superfamily)
29*	0.010	COG0029 [H]	Aspartate oxidase
30*	0.010	COG0538 [C]	Isocitrate dehydrogenases

Table S 104: Module 166 – with rank 55 according to estimated information content.

Rank	Probability	OG	Description
1*	0.152	COG2207 [K]	AraC-type DNA-binding domain-containing proteins
2*	0.051	COG2771 [K]	DNA-binding HTH domain-containing proteins
3*	0.039	COG1012 [C]	NAD-dependent aldehyde dehydrogenases
4*	0.032	COG0697 [G,E,R]	Permeases of the drug/metabolite transporter (DMT) superfamily
5*	0.027	COG0673 [R]	Predicted dehydrogenases and related proteins
6	0.025	COG0861 [P]	Membrane protein TerC, possibly involved in tellurium resistance
7*	0.023	COG0366 [G]	Glycosidases
8*	0.023	COG0451 [M,G]	Nucleoside-diphosphate-sugar epimerases
9	0.022	COG1073 [R]	Hydrolases of the alpha/beta superfamily
10	0.021	COG0493 [E,R]	NADPH-dependent glutamate synthase beta chain and related oxidoreductases
11*	0.019	COG0789 [K]	Predicted transcriptional regulators
12*	0.019	COG3104 [E]	Dipeptide/tripeptide permease
13*	0.018	COG2188 [K]	Transcriptional regulators
14	0.016	COG2076 [P]	Membrane transporters of cations and cationic drugs
15	0.016	COG1760 [E]	L-serine deaminase
16*	0.015	COG1486 [G]	Alpha-galactosidases/6-phospho-beta-glucosidases, family 4 of glycosyl hydrolases
17	0.014	COG0791 [M]	Cell wall-associated hydrolases (invasion-associated proteins)
18	0.014	COG0471 [P]	Di- and tricarboxylate transporters
19*	0.014	COG1414 [K]	Transcriptional regulator
20*	0.013	COG1301 [C]	Na+/H+-dicarboxylate symporters
21*	0.013	COG2244 [R]	Membrane protein involved in the export of O-antigen and teichoic acid
22	0.013	COG0687 [E]	Spermidine/putrescine-binding periplasmic protein
23*	0.012	COG0371 [C]	Glycerol dehydrogenase and related enzymes
24*	0.012	COG1221 [K,T]	Transcriptional regulators containing an AAA-type ATPase domain and a DNA-binding domain
25	0.012	COG0444 [E,P]	ABC-type dipeptide/oligopeptide/nickel transport system, ATPase component
26*	0.012	COG0071 [O]	Molecular chaperone (small heat shock protein)
27*	0.010	COG0280 [C]	Phosphotransacetylase

Table S 105: Module 48 – with rank 57 according to estimated information content.

Rank	Probability	OG	Description
1*	0.075	COG0463 [M]	Glycosyltransferases involved in cell wall biogenesis
2*	0.070	COG0451 [M,G]	Nucleoside-diphosphate-sugar epimerases
3*	0.050	COG0399 [M]	Predicted pyridoxal phosphate-dependent enzyme apparently involved in regulation of cell wall biogenesis
4*	0.041	COG2870 [M]	ADP-heptose synthase, bifunctional sugar kinase/adenylyltransferase
5*	0.040	COG0859 [M]	ADP-heptose:LPS heptosyltransferase
6*	0.038	COG0526 [O,C]	Thiol-disulfide isomerase and thioredoxins
7*	0.037	COG0241 [E]	Histidinol phosphatase and related phosphatases
8*	0.033	COG0279 [G]	Phosphoheptose isomerase
9*	0.031	COG2089 [M]	Sialic acid synthase
10*	0.031	COG0673 [R]	Predicted dehydrogenases and related proteins
11	0.027	COG0659 [P]	Sulfate permease and related transporters (MFS superfamily)
12*	0.024	COG1083 [M]	CMP-N-acetylneuraminic acid synthetase
13*	0.023	COG0436 [E]	Aspartate/tyrosine/aromatic aminotransferase
14*	0.023	COG0381 [M]	UDP-N-acetylglucosamine 2-epimerase
15	0.022	COG0475 [P]	Kef-type K+ transport systems, membrane components
16*	0.020	COG1004 [M]	Predicted UDP-glucose 6-dehydrogenase
17*	0.017	COG0586 [S]	Uncharacterized membrane-associated protein
18*	0.016	COG1208 [M,J]	Nucleoside-diphosphate-sugar pyrophosphorylase involved in lipopolysaccharide biosynthesis/translation initiation factor 2B, gamma/epsilon subunits (eIF-2Bgamma/eIF-2Bepsilon)
19*	0.016	COG0720 [H]	6-pyruvoyl-tetrahydropterin synthase
20*	0.015	COG0225 [O]	Peptide methionine sulfoxide reductase
21*	0.014	COG3959 [G]	Transketolase, N-terminal subunit
22*	0.014	COG0127 [F]	Xanthosine triphosphate pyrophosphatase
23*	0.014	COG0492 [O]	Thioredoxin reductase
24*	0.014	COG0210 [L]	Superfamily I DNA and RNA helicases
25*	0.013	COG0815 [M]	Apolipoprotein N-acyltransferase
26*	0.012	COG0611 [H]	Thiamine monophosphate kinase
27*	0.012	COG0450 [O]	Peroxiredoxin
28	0.011	COG0861 [P]	Membrane protein TerC, possibly involved in tellurium resistance
29*	0.011	COG0209 [F]	Ribonucleotide reductase, alpha subunit
30	0.011	COG0547 [E]	Anthranilate phosphoribosyltransferase
31*	0.010	COG0483 [G]	Archaeal fructose-1,6-bisphosphatase and related enzymes of inositol monophosphatase family

Table S 106: Module 191 – with rank 58 according to estimated information content.

Rank	Probability	OG	Description
1*	0.086	COG0438 [M]	Glycosyltransferase
2*	0.068	COG0500 [Q,R]	SAM-dependent methyltransferases
3*	0.047	COG0463 [M]	Glycosyltransferases involved in cell wall biogenesis
4*	0.045	COG0494 [L,R]	NTP pyrophosphohydrolases including oxidative damage repair enzymes
5*	0.030	COG0714 [R]	MoxR-like ATPases
6*	0.025	COG1009 [C,P]	NADH:ubiquinone oxidoreductase subunit 5 (chain L)/Multisubunit Na+/H+ antiporter, MnhA subunit
7*	0.023	COG0501 [O]	Zn-dependent protease with chaperone function
8*	0.022	COG0377 [C]	NADH:ubiquinone oxidoreductase 20 kD subunit and related Fe-S oxidoreductases
9	0.020	COG2220 [R]	Predicted Zn-dependent hydrolases of the beta-lactamase fold
10*	0.020	COG0674 [C]	Pyruvate:ferredoxin oxidoreductase and related 2-oxoacid:ferredoxin oxidoreductases, alpha subunit
11*	0.020	COG0649 [C]	NADH:ubiquinone oxidoreductase 49 kD subunit 7
12*	0.020	COG1005 [C]	NADH:ubiquinone oxidoreductase subunit 1 (chain H)
13*	0.020	COG0517 [R]	FOG: CBS domain
14*	0.020	COG1013 [C]	Pyruvate:ferredoxin oxidoreductase and related 2-oxoacid:ferredoxin oxidoreductases, beta subunit
15*	0.019	COG0852 [C]	NADH:ubiquinone oxidoreductase 27 kD subunit
16*	0.018	COG1143 [C]	Formate hydrogenlyase subunit 6/NADH:ubiquinone oxidoreductase 23 kD subunit (chain I)
17*	0.017	COG1208 [M,J]	Nucleoside-diphosphate-sugar pyrophosphorylase involved in lipopolysaccharide biosynthesis/translation initiation factor 2B, gamma/epsilon subunits (eIF-2Bgamma/eIF-2Bepsilon)
18*	0.017	COG0031 [E]	Cysteine synthase
19*	0.017	COG0005 [F]	Purine nucleoside phosphorylase
20*	0.017	COG0626 [E]	Cystathionine beta-lyases/cystathionine gamma-synthases
21*	0.016	COG1007 [C]	NADH:ubiquinone oxidoreductase subunit 2 (chain N)
22*	0.016	COG0838 [C]	NADH:ubiquinone oxidoreductase subunit 3 (chain A)
23	0.016	COG1573 [L]	Uracil-DNA glycosylase
24*	0.016	COG0665 [E]	Glycine/D-amino acid oxidases (deaminating)
25*	0.015	COG0713 [C]	NADH:ubiquinone oxidoreductase subunit 11 or 4L (chain K)
26*	0.015	COG0839 [C]	NADH:ubiquinone oxidoreductase subunit 6 (chain J)
27*	0.014	COG0281 [C]	Malic enzyme
28*	0.014	COG1994 [R]	Zn-dependent proteases

29*	0.013	COG0524 [G]	Sugar kinases, ribokinase family
30*	0.012	COG0190 [H]	5,10-methylene-tetrahydrofolate dehydrogenase/Methenyl tetrahydrofolate cyclohydrolase
31*	0.012	COG0480 [J]	Translation elongation factors (GTPases)
32*	0.012	COG0537 [F,G,R]	Diadenosine tetraphosphate (Ap4A) hydrolase and other HIT family hydrolases
33*	0.012	COG1014 [C]	Pyruvate:ferredoxin oxidoreductase and related 2-oxoacid:ferredoxin oxidoreductases, gamma subunit
34*	0.011	COG0476 [H]	Dinucleotide-utilizing enzymes involved in molybdopterin and thiamine biosynthesis family 2
35*	0.011	COG1008 [C]	NADH:ubiquinone oxidoreductase subunit 4 (chain M)
36*	0.011	COG0071 [O]	Molecular chaperone (small heat shock protein)
37*	0.010	COG0607 [P]	Rhodanese-related sulfurtransferase
38*	0.010	COG1977 [H]	Molybdopterin converting factor, small subunit
39*	0.010	COG0330 [O]	Membrane protease subunits, stomatin/prohibitin homologs
40	0.010	COG2154 [H]	Pterin-4a-carbinolamine dehydratase

Table S 107: Module 196 – with rank 59 according to estimated information content.

Rank	Probability	OG	Description
1	0.130	COG3209 [M]	Rhs family protein
2*	0.064	COG0477 [G,E,P,R]	Permeases of the major facilitator superfamily
3	0.062	COG2310 [T]	Uncharacterized proteins involved in stress response, homologs of TerZ and putative cAMP-binding protein CABP1
4*	0.042	COG1396 [K]	Predicted transcriptional regulators
5*	0.030	COG0454 [K,R]	Histone acetyltransferase HPA2 and related acetyltransferases
6	0.028	COG1136 [V]	ABC-type antimicrobial peptide transport system, ATPase component
7	0.027	COG0251 [J]	Putative translation initiation inhibitor, yjgF family
8*	0.025	COG4886 [S]	Leucine-rich repeat (LRR) protein
9	0.023	COG1961 [L]	Site-specific recombinases, DNA invertase Pin homologs
10*	0.022	COG0697 [G,E,R]	Permeases of the drug/metabolite transporter (DMT) superfamily
11	0.021	COG0715 [P]	ABC-type nitrate/sulfonate/bicarbonate transport systems, periplasmic components
12*	0.019	COG0500 [Q,R]	SAM-dependent methyltransferases
13*	0.017	COG0388 [R]	Predicted amidohydrolase
14*	0.016	COG0288 [P]	Carbonic anhydrase
15	0.015	COG1178 [P]	ABC-type Fe3+ transport system, permease component
16*	0.015	COG0515 [R,T,K,L]	Serine/threonine protein kinase
17*	0.015	COG1974 [K,T]	SOS-response transcriptional repressors (RecA-mediated autopeptidases)
18	0.013	COG0714 [R]	MoxR-like ATPases
19	0.013	COG3793 [P]	Tellurite resistance protein
20	0.013	NOG06421 [S]	Annotation not available
21	0.012	COG0191 [G]	Fructose/tagatose bisphosphate aldolase
22	0.012	COG0574 [G]	Phosphoenolpyruvate synthase/pyruvate phosphate dikinase
23	0.012	COG4245 [R]	Uncharacterized protein encoded in toxicity protection region of plasmid R478, contains von Willebrand factor (vWF) domain
24	0.012	COG1052 [C,H,R]	Lactate dehydrogenase and related dehydrogenases
25	0.011	NOG06420 [S]	Annotation not available
26	0.011	COG1479 [S]	Uncharacterized conserved protein
27	0.011	COG4110 [R]	Uncharacterized protein involved in stress response
28	0.010	COG5281 [S]	Phage-related minor tail protein

Table S 108: Module 93 – with rank 60 according to estimated information content.

Rank	Probability	OG	Description
1*	0.114	COG1629 [P]	Outer membrane receptor proteins, mostly Fe transport
2*	0.058	COG1538 [M,U]	Outer membrane protein
3*	0.054	COG1595 [K]	DNA-directed RNA polymerase specialized sigma subunit, sigma24 homolog
4*	0.042	COG0745 [T,K]	Response regulators consisting of a CheY-like receiver domain and a winged-helix DNA-binding domain
5	0.037	COG0715 [P]	ABC-type nitrate/sulfonate/bicarbonate transport systems, periplasmic components
6*	0.032	COG3712 [P,T]	Fe2+-dicitrate sensor, membrane component
7*	0.030	COG0845 [M]	Membrane-fusion protein
8	0.028	COG3210 [U]	Large exoproteins involved in heme utilization or adhesion
9*	0.022	COG0841 [V]	Cation/multidrug efflux pump
10	0.020	COG0454 [K,R]	Histone acetyltransferase HPA2 and related acetyltransferases
11*	0.019	COG3182 [S]	Uncharacterized iron-regulated membrane protein
12	0.017	COG0600 [P]	ABC-type nitrate/sulfonate/bicarbonate transport system, permease component
13*	0.017	COG0848 [U]	Biopolymer transport protein
14*	0.016	COG4773 [P]	Outer membrane receptor for ferric coprogen and ferric-rhodotorulic acid
15	0.016	COG1116 [P]	ABC-type nitrate/sulfonate/bicarbonate transport system, ATPase component
16	0.016	COG1167 [K,E]	Transcriptional regulators containing a DNA-binding HTH domain and an aminotransferase domain (MocR family) and their eukaryotic orthologs
17	0.015	COG4625 [S]	Uncharacterized protein with a C-terminal OMP (outer membrane protein) domain
18	0.013	COG1464 [P]	ABC-type metal ion transport system, periplasmic component/surface antigen
19	0.013	COG0861 [P]	Membrane protein TerC, possibly involved in tellurium resistance
20*	0.011	COG1309 [K]	Transcriptional regulator
21	0.011	COG1984 [E]	Allophanate hydrolase subunit 2
22	0.010	COG3659 [M]	Carbohydrate-selective porin
23	0.010	COG1235 [R]	Metal-dependent hydrolases of the beta-lactamase superfamily I

Table S 109: Module 80 – with rank 62 according to estimated information content.

Rank	Probability	OG	Description
1*	0.051	COG0768 [M]	Cell division protein FtsI/penicillin-binding protein 2
2*	0.036	COG0772 [D]	Bacterial cell division membrane protein
3*	0.032	COG0847 [L]	DNA polymerase III, epsilon subunit and related 3-5 exonucleases
4	0.030	COG0042 [J]	tRNA-dihydrouridine synthase
5*	0.030	COG0204 [I]	1-acyl-sn-glycerol-3-phosphate acyltransferase
6*	0.029	COG0740 [O,U]	Protease subunit of ATP-dependent Clp proteases
7*	0.027	COG1674 [D]	DNA segregation ATPase FtsK/SpoIIIE and related proteins
8*	0.027	COG1219 [O]	ATP-dependent protease Clp, ATPase subunit
9*	0.025	COG0756 [F]	dUTPase
10*	0.025	COG1185 [J]	Polyribonucleotide nucleotidyltransferase (polynucleotide phosphorylase)
11*	0.024	COG0771 [M]	UDP-N-acetylmuramoylalanine-D-glutamate ligase
12	0.024	COG1862 [U]	Preprotein translocase subunit YajC
13*	0.023	COG0258 [L]	5-3 exonuclease (including N-terminal domain of PolI)
14*	0.023	COG0812 [M]	UDP-N-acetylmuramate dehydrogenase
15*	0.023	COG1074 [L]	ATP-dependent exoDNAse (exonuclease V) beta subunit (contains helicase and exonuclease domains)
16*	0.022	COG0294 [H]	Dihydropteroate synthase and related enzymes
17	0.022	COG1158 [K]	Transcription termination factor
18*	0.021	COG0331 [I]	(acyl-carrier-protein) S-malonyltransferase
19*	0.021	COG0773 [M]	UDP-N-acetylmuramate-alanine ligase
20	0.021	COG0289 [E]	Dihydrodipicolinate reductase
21	0.021	COG0136 [E]	Aspartate-semialdehyde dehydrogenase
22*	0.021	COG1181 [M]	D-alanine-D-alanine ligase and related ATP-grasp enzymes
23*	0.020	COG0608 [L]	Single-stranded DNA-specific exonuclease
24	0.020	COG0781 [K]	Transcription termination factor
25*	0.019	COG0304 [I,Q]	3-oxoacyl-(acyl-carrier-protein) synthase
26*	0.019	COG0472 [M]	UDP-N-acetylmuramyl pentapeptide phosphotransferase/UDP-N-acetylglucosamine-1-phosphate transferase
27*	0.019	COG1792 [M]	Cell shape-determining protein
28*	0.019	COG0265 [O]	Trypsin-like serine proteases, typically periplasmic, contain C-terminal PDZ domain

29*	0.019	COG0332 [I]	3-oxoacyl-[acyl-carrier-protein] synthase III
30	0.018	COG0407 [H]	Uroporphyrinogen-III decarboxylase
31	0.017	COG1104 [E]	Cysteine sulfinate desulfinase/cysteine desulfurase and related enzymes
32*	0.017	COG1077 [D]	Actin-like ATPase involved in cell morphogenesis
33	0.015	COG1301 [C]	Na+/H+-dicarboxylate symporters
34	0.015	COG0527 [E]	Aspartokinases
35*	0.013	COG0563 [F]	Adenylate kinase and related kinases
36	0.012	COG0694 [O]	Thioredoxin-like proteins and domains
37*	0.012	COG0517 [R]	FOG: CBS domain
38	0.012	COG2094 [L]	3-methyladenine DNA glycosylase
39*	0.011	COG0564 [J]	Pseudouridylate synthases, 23S RNA-specific
40*	0.010	COG0262 [H]	Dihydrofolate reductase

Table S 110: Module 125 – with rank 63 according to estimated information content.

Rank	Probability	OG	Description
1*	0.045	COG0741 [M]	Soluble lytic murein transglycosylase and related regulatory proteins (some contain LysM/invasin domains)
2*	0.042	COG0739 [M]	Membrane proteins related to metalloendopeptidases
3*	0.039	COG0776 [L]	Bacterial nucleoid DNA-binding protein
4*	0.036	COG0760 [O]	Parvulin-like peptidyl-prolyl isomerase
5	0.033	COG0494 [L,R]	NTP pyrophosphohydrolases including oxidative damage repair enzymes
6	0.026	COG0607 [P]	Rhodanese-related sulfurtransferase
7*	0.026	COG0265 [O]	Trypsin-like serine proteases, typically periplasmic, contain C-terminal PDZ domain
8	0.019	COG1959 [K]	Predicted transcriptional regulator
9	0.019	COG3288 [C]	NAD/NADP transhydrogenase alpha subunit
10	0.018	COG0780 [R]	Enzyme related to GTP cyclohydrolase I
11*	0.018	COG1452 [M]	Organic solvent tolerance protein OstA
12	0.018	COG1559 [R]	Predicted periplasmic solute-binding protein
13	0.018	COG0794 [M]	Predicted sugar phosphate isomerase involved in capsule formation
14	0.017	COG2853 [M]	Surface lipoprotein
15	0.017	COG1663 [M]	Tetraacyldisaccharide-1-P 4-kinase
16	0.017	COG1282 [C]	NAD/NADP transhydrogenase beta subunit
17	0.017	COG1729 [S]	Uncharacterized protein conserved in bacteria
18	0.016	COG2001 [S]	Uncharacterized protein conserved in bacteria
19*	0.016	COG0073 [R]	EMAP domain
20*	0.016	COG1472 [G]	Beta-glucosidase-related glycosidases
21	0.016	COG1043 [M]	Acyl-[acyl carrier protein]–UDP-N-acetylglucosamine O-acyltransferase
22*	0.016	COG0187 [L]	Type IIA topoisomerase (DNA gyrase/topo II, topoisomerase IV), B subunit
23	0.016	COG0773 [M]	UDP-N-acetylmuramate-alanine ligase
24*	0.016	COG0793 [M]	Periplasmic protease
25	0.016	COG0603 [R]	Predicted PP-loop superfamily ATPase
26	0.015	COG1267 [I]	Phosphatidylglycerophosphatase A and related proteins
27	0.015	COG0752 [J]	Glycyl-tRNA synthetase, alpha subunit
28	0.015	COG1077 [D]	Actin-like ATPase involved in cell morphogenesis

29*	0.015	COG5009 [M]	Membrane carboxypeptidase/penicillin-binding protein
30*	0.014	COG0050 [J]	GTPases - translation elongation factors
31	0.014	COG0602 [O]	Organic radical activating enzymes
32*	0.014	COG0450 [O]	Peroxiredoxin
33*	0.014	COG0593 [L]	ATPase involved in DNA replication initiation
34	0.013	COG3264 [M]	Small-conductance mechanosensitive channel
35	0.013	COG0797 [M]	Lipoproteins
36	0.013	COG1792 [M]	Cell shape-determining protein
37	0.013	COG0598 [P]	Mg2+ and Co2+ transporters
38*	0.012	COG0174 [E]	Glutamine synthetase
39	0.012	COG2982 [M]	Uncharacterized protein involved in outer membrane biogenesis
40*	0.012	COG0783 [P]	DNA-binding ferritin-like protein (oxidative damage protectant)
41	0.011	COG1434 [S]	Uncharacterized conserved protein
42	0.011	COG1076 [O]	DnaJ-domain-containing proteins 1
43	0.011	COG0350 [L]	Methylated DNA-protein cysteine methyltransferase
44	0.011	COG1881 [R]	Phospholipid-binding protein
45	0.010	COG0805 [U]	Sec-independent protein secretion pathway component TatC

Table S 111: Module 157 – with rank 64 according to estimated information content.

Rank	Probability	OG	Description
1*	0.087	COG0243 [C]	Anaerobic dehydrogenases, typically selenocysteine-containing
2*	0.069	COG0437 [C]	Fe-S-cluster-containing hydrogenase components 1
3*	0.069	COG0477 [G,E,P,R]	Permeases of the major facilitator superfamily
4*	0.045	COG0651 [C,P]	Formate hydrogenlyase subunit 3/Multisubunit Na+/H+ antiporter, MnhD subunit
5*	0.045	COG1053 [C]	Succinate dehydrogenase/fumarate reductase, flavoprotein subunit
6*	0.033	COG1131 [V]	ABC-type multidrug transport system, ATPase component
7	0.031	COG0582 [L]	Integrase
8*	0.027	COG3261 [C]	Ni,Fe-hydrogenase III large subunit
9*	0.026	COG3260 [C]	Ni,Fe-hydrogenase III small subunit
10*	0.022	COG0650 [C]	Formate hydrogenlyase subunit 4
11*	0.018	COG1180 [O]	Pyruvate-formate lyase-activating enzyme
12*	0.018	COG0664 [T]	cAMP-binding proteins - catabolite gene activator and regulatory subunit of cAMP-dependent protein kinases
13*	0.016	COG1410 [E]	Methionine synthase I, cobalamin-binding domain
14*	0.016	COG0348 [C]	Polyferredoxin
15*	0.016	COG1136 [V]	ABC-type antimicrobial peptide transport system, ATPase component
16*	0.016	COG0471 [P]	Di- and tricarboxylate transporters
17*	0.014	COG4237 [C]	Hydrogenase 4 membrane component (E)
18*	0.013	COG0436 [E]	Aspartate/tyrosine/aromatic aminotransferase
19*	0.013	COG1142 [C]	Fe-S-cluster-containing hydrogenase components 2
20*	0.013	COG1924 [I]	Activator of 2-hydroxyglutaryl-CoA dehydratase (HSP70-class ATPase domain)
21*	0.013	COG0440 [E]	Acetolactate synthase, small (regulatory) subunit
22*	0.012	COG0642 [T]	Signal transduction histidine kinase
23	0.012	COG1620 [C]	L-lactate permease
24*	0.011	COG3302 [R]	DMSO reductase anchor subunit
25*	0.010	COG3381 [R]	Uncharacterized component of anaerobic dehydrogenases

Table S 112: Module 71 – with rank 65 according to estimated information content.

Rank	Probability	OG	Description
1*	0.139	COG2931 [Q]	RTX toxins and related Ca2+-binding proteins
2	0.073	COG3210 [U]	Large exoproteins involved in heme utilization or adhesion
3*	0.053	COG0457 [R]	FOG: TPR repeat
4*	0.048	COG1262 [S]	Uncharacterized conserved protein
5	0.026	COG2831 [U]	Hemolysin activation/secretion protein
6*	0.026	COG2274 [V]	ABC-type bacteriocin/lantibiotic exporters, contain an N-terminal double-glycine peptidase domain
7*	0.025	COG0845 [M]	Membrane-fusion protein
8	0.023	COG0834 [E,T]	ABC-type amino acid transport/signal transduction systems, periplasmic component/domain
9*	0.017	COG0863 [L]	DNA modification methylase
10	0.017	COG0358 [L]	DNA primase (bacterial type)
11*	0.017	COG0776 [L]	Bacterial nucleoid DNA-binding protein
12*	0.016	COG0517 [R]	FOG: CBS domain
13	0.016	COG5525 [R]	Bacteriophage tail assembly protein
14*	0.014	COG0760 [O]	Parvulin-like peptidyl-prolyl isomerase
15	0.013	COG1961 [L]	Site-specific recombinases, DNA invertase Pin homologs
16	0.012	COG3344 [L]	Retron-type reverse transcriptase
17*	0.012	COG2208 [T,K]	Serine phosphatase RsbU, regulator of sigma subunit
18*	0.012	COG0790 [R]	FOG: TPR repeat, SEL1 subfamily
19	0.011	NOG69524 [S]	Annotation not available
20*	0.011	COG0639 [T]	Diadenosine tetraphosphatase and related serine/threonine protein phosphatases
21	0.011	COG0616 [O,U]	Periplasmic serine proteases (ClpP class)
22	0.011	COG5511 [R]	Bacteriophage capsid protein
23*	0.010	COG1475 [K]	Predicted transcriptional regulators

Table S 113: Module 175 – with rank 67 according to estimated information content.

Rank	Probability	OG	Description
1*	0.116	COG0477 [G,E,P,R]	Permeases of the major facilitator superfamily
2*	0.050	COG1414 [K]	Transcriptional regulator
3*	0.045	COG1012 [C]	NAD-dependent aldehyde dehydrogenases
4*	0.037	COG0583 [K]	Transcriptional regulator
5*	0.031	COG0654 [H,C]	2-polyprenyl-6-methoxyphenol hydroxylase and related FAD-dependent oxidoreductases
6*	0.024	COG1018 [C]	Flavodoxin reductases (ferredoxin-NADPH reductases) family 1
7*	0.023	COG0665 [E]	Glycine/D-amino acid oxidases (deaminating)
8*	0.023	COG0179 [Q]	2-keto-4-pentenoate hydratase/2-oxohepta-3-ene-1,7-dioic acid hydratase (catechol pathway)
9*	0.021	COG4948 [M,R]	L-alanine-DL-glutamate epimerase and related enzymes of enolase superfamily
10*	0.020	COG1802 [K]	Transcriptional regulators
11*	0.019	COG1396 [K]	Predicted transcriptional regulators
12*	0.019	COG1473 [R]	Metal-dependent amidase/aminoacylase/carboxypeptidase
13*	0.019	COG3485 [Q]	Protocatechuate 3,4-dioxygenase beta subunit
14*	0.017	COG2186 [K]	Transcriptional regulators
15*	0.016	COG1609 [K]	Transcriptional regulators
16*	0.016	COG0745 [T,K]	Response regulators consisting of a CheY-like receiver domain and a winged-helix DNA-binding domain
17*	0.015	COG1167 [K,E]	Transcriptional regulators containing a DNA-binding HTH domain and an aminotransferase domain (MocR family) and their eukaryotic orthologs
18	0.015	COG0402 [F,R]	Cytosine deaminase and related metal-dependent hydrolases
19*	0.013	COG0277 [C]	FAD/FMN-containing dehydrogenases
20*	0.013	COG0235 [G]	Ribulose-5-phosphate 4-epimerase and related epimerases and aldolases
21*	0.013	COG1853 [R]	Conserved protein/domain typically associated with flavoprotein oxygenases, DIM6/NTAB family
22*	0.012	COG1522 [K]	Transcriptional regulators
23*	0.010	COG0726 [G]	Predicted xylanase/chitin deacetylase

Table S 114: Module 2 – with rank 68 according to estimated information content.

Rank	Probability	OG	Description
1*	0.064	COG0596 [R]	Predicted hydrolases or acyltransferases (alpha/beta hydrolase superfamily)
2*	0.061	COG0500 [Q,R]	SAM-dependent methyltransferases
3*	0.051	COG0457 [R]	FOG: TPR repeat
4*	0.043	COG0438 [M]	Glycosyltransferase
5*	0.034	COG2710 [C]	Nitrogenase molybdenum-iron protein, alpha and beta chains
6*	0.027	COG1226 [P]	Kef-type K+ transport systems, predicted NAD-binding component
7*	0.024	COG0644 [C]	Dehydrogenases (flavoproteins)
8*	0.022	COG0637 [R]	Predicted phosphatase/phosphohexomutase
9*	0.020	COG0347 [E]	Nitrogen regulatory protein PII
10*	0.020	COG0443 [O]	Molecular chaperone
11	0.019	COG0226 [P]	ABC-type phosphate transport system, periplasmic component
12*	0.019	COG1208 [M,J]	Nucleoside-diphosphate-sugar pyrophosphorylase involved in lipopolysaccharide biosynthesis/translation initiation factor 2B, gamma/epsilon subunits (eIF-2Bgamma/eIF-2Bepsilon)
13*	0.018	COG0382 [H]	4-hydroxybenzoate polyprenyltransferase and related prenyltransferases
14*	0.018	COG1239 [H]	Mg-chelatase subunit ChlI
15*	0.018	COG1215 [M]	Glycosyltransferases, probably involved in cell wall biogenesis
16*	0.016	COG0607 [P]	Rhodanese-related sulfurtransferase
17*	0.016	COG1429 [H]	Cobalamin biosynthesis protein CobN and related Mg-chelatases
18*	0.014	COG1348 [P]	Nitrogenase subunit NifH (ATPase)
19*	0.014	COG0004 [P]	Ammonia permease
20*	0.014	COG1032 [C]	Fe-S oxidoreductase
21	0.013	COG1305 [E]	Transglutaminase-like enzymes, putative cysteine proteases
22*	0.013	COG0058 [G]	Glucan phosphorylase
23*	0.013	COG0616 [O,U]	Periplasmic serine proteases (ClpP class)
24*	0.012	COG0750 [M]	Predicted membrane-associated Zn-dependent proteases 1
25*	0.011	COG0535 [R]	Predicted Fe-S oxidoreductases
26*	0.011	COG1409 [R]	Predicted phosphohydrolases
27*	0.011	COG0747 [E]	ABC-type dipeptide transport system, periplasmic component
28	0.011	COG1573 [L]	Uracil-DNA glycosylase
29*	0.010	COG1104 [E]	Cysteine sulfinate desulfinase/cysteine desulfurase and related enzymes
30	0.010	COG0067 [E]	Glutamate synthase domain 1

Table S 115: Module 44 – with rank 69 according to estimated information content.

Rank	Probability	OG	Description
1*	0.136	COG1132 [V]	ABC-type multidrug transport system, ATPase and permease components
2	0.054	COG0494 [L,R]	NTP pyrophosphohydrolases including oxidative damage repair enzymes
3*	0.053	COG0577 [V]	ABC-type antimicrobial peptide transport system, permease component
4	0.030	COG1122 [P]	ABC-type cobalt transport system, ATPase component
5*	0.030	COG1309 [K]	Transcriptional regulator
6*	0.022	COG1136 [V]	ABC-type antimicrobial peptide transport system, ATPase component
7*	0.021	COG1131 [V]	ABC-type multidrug transport system, ATPase component
8	0.020	COG4166 [E]	ABC-type oligopeptide transport system, periplasmic component
9*	0.019	COG0596 [R]	Predicted hydrolases or acyltransferases (alpha/beta hydrolase superfamily)
10	0.018	COG1087 [M]	UDP-glucose 4-epimerase
11	0.015	COG1670 [J]	Acetyltransferases, including N-acetylases of ribosomal proteins
12	0.015	COG3764 [M]	Sortase (surface protein transpeptidase)
13	0.015	COG1473 [R]	Metal-dependent amidase/aminoacylase/carboxypeptidase
14	0.015	COG0456 [R]	Acetyltransferases
15	0.012	COG1168 [E]	Bifunctional PLP-dependent enzyme with beta-cystathionase and maltose regulon repressor activities
16	0.011	COG0111 [H,E]	Phosphoglycerate dehydrogenase and related dehydrogenases
17	0.011	COG2966 [S]	Uncharacterized conserved protein
18	0.011	COG1511 [S]	Predicted membrane protein
19	0.010	COG0619 [P]	ABC-type cobalt transport system, permease component CbiQ and related transporters

Table S 116: Module 77 – with rank 70 according to estimated information content.

Rank	Probability	OG	Description
1*	0.056	COG0526 [O,C]	Thiol-disulfide isomerase and thioredoxins
2*	0.032	COG1108 [P]	ABC-type Mn2+/Zn2+ transport systems, permease components
3*	0.031	COG1121 [P]	ABC-type Mn/Zn transport systems, ATPase component
4	0.028	COG2095 [U]	Multiple antibiotic transporter
5*	0.025	COG1502 [I]	Phosphatidylserine/phosphatidylglycerophosphate/cardiolipin synthases and related enzymes
6*	0.024	COG0635 [H]	Coproporphyrinogen III oxidase and related Fe-S oxidoreductases
7	0.023	COG1115 [E]	Na+/alanine symporter
8*	0.022	COG0803 [P]	ABC-type metal ion transport system, periplasmic component/surface adhesin
9*	0.022	COG0664 [T]	cAMP-binding proteins - catabolite gene activator and regulatory subunit of cAMP-dependent protein kinases
10	0.021	COG2849 [S]	Uncharacterized protein conserved in bacteria
11*	0.021	COG0616 [O,U]	Periplasmic serine proteases (ClpP class)
12*	0.020	COG0623 [I]	Enoyl-[acyl-carrier-protein] reductase (NADH)
13	0.019	COG2871 [C]	Na+-transporting NADH:ubiquinone oxidoreductase, subunit NqrF
14	0.019	COG1738 [S]	Uncharacterized conserved protein
15	0.019	COG1347 [C]	Na+-transporting NADH:ubiquinone oxidoreductase, subunit NqrD
16	0.018	COG1805 [C]	Na+-transporting NADH:ubiquinone oxidoreductase, subunit NqrB
17	0.018	COG2869 [C]	Na+-transporting NADH:ubiquinone oxidoreductase, subunit NqrC
18	0.017	COG0342 [U]	Preprotein translocase subunit SecD
19*	0.017	COG0588 [G]	Phosphoglycerate mutase 1
20	0.017	COG2239 [P]	Mg/Co/Ni transporter MgtE (contains CBS domain)
21	0.017	COG1726 [C]	Na+-transporting NADH:ubiquinone oxidoreductase, subunit NqrA
22	0.016	COG0323 [L]	DNA mismatch repair enzyme (predicted ATPase)
23	0.016	COG2209 [C]	Na+-transporting NADH:ubiquinone oxidoreductase, subunit NqrE
24*	0.016	COG0752 [J]	Glycyl-tRNA synthetase, alpha subunit
25	0.015	COG1477 [H]	Membrane-associated lipoprotein involved in thiamine biosynthesis
26*	0.015	COG0488 [R]	ATPase components of ABC transporters with duplicated ATPase domains
27	0.015	COG2885 [M]	Outer membrane protein and related peptidoglycan-associated (lipo)proteins
28	0.014	COG0775 [F]	Nucleoside phosphorylase

29*	0.014	COG0364 [G]	Glucose-6-phosphate 1-dehydrogenase
30	0.014	COG0494 [L,R]	NTP pyrophosphohydrolases including oxidative damage repair enzymes
31*	0.014	COG1539 [H]	Dihydroneopterin aldolase
32*	0.014	COG0362 [G]	6-phosphogluconate dehydrogenase
33*	0.013	COG0764 [I]	3-hydroxymyristoyl/3-hydroxydecanoyl-(acyl carrier protein) dehydratases
34	0.013	COG1295 [S]	Predicted membrane protein
35*	0.012	COG0815 [M]	Apolipoprotein N-acyltransferase
36	0.012	COG0860 [M]	N-acetylmuramoyl-L-alanine amidase
37*	0.012	COG0045 [C]	Succinyl-CoA synthetase, beta subunit
38	0.012	COG0821 [I]	Enzyme involved in the deoxyxylulose pathway of isoprenoid biosynthesis
39	0.011	COG0703 [E]	Shikimate kinase
40	0.011	COG0424 [D]	Nucleotide-binding protein implicated in inhibition of septum formation
41*	0.011	COG1154 [H,I]	Deoxyxylulose-5-phosphate synthase
42*	0.011	COG0439 [I]	Biotin carboxylase
43*	0.011	COG0486 [R]	Predicted GTPase
44	0.010	COG1734 [T]	DnaK suppressor protein
45*	0.010	COG0057 [G]	Glyceraldehyde-3-phosphate dehydrogenase/erythrose-4-phosphate dehydrogenase

Table S 117: Module 58 – with rank 72 according to estimated information content.

Rank	Probability	OG	Description
1*	0.065	COG1028 [I,Q,R]	Dehydrogenases with different specificities (related to short-chain alcohol dehydrogenases)
2*	0.064	COG1012 [C]	NAD-dependent aldehyde dehydrogenases
3*	0.044	COG0834 [E,T]	ABC-type amino acid transport/signal transduction systems, periplasmic component/domain
4*	0.031	COG0765 [E]	ABC-type amino acid transport system, permease component
5	0.029	COG2113 [E]	ABC-type proline/glycine betaine transport systems, periplasmic components
6*	0.027	COG1802 [K]	Transcriptional regulators
7*	0.027	COG0451 [M,G]	Nucleoside-diphosphate-sugar epimerases
8	0.024	COG3842 [E]	ABC-type spermidine/putrescine transport systems, ATPase components
9*	0.020	COG0665 [E]	Glycine/D-amino acid oxidases (deaminating)
10*	0.019	COG1126 [E]	ABC-type polar amino acid transport system, ATPase component
11	0.018	COG1178 [P]	ABC-type Fe3+ transport system, permease component
12*	0.018	COG0161 [H]	Adenosylmethionine-8-amino-7-oxononanoate aminotransferase
13	0.018	COG4176 [E]	ABC-type proline/glycine betaine transport system, permease component
14	0.017	COG4175 [E]	ABC-type proline/glycine betaine transport system, ATPase component
15*	0.017	COG0006 [E]	Xaa-Pro aminopeptidase
16*	0.017	COG0346 [E]	Lactoylglutathione lyase and related lyases
17*	0.016	COG1052 [C,H,R]	Lactate dehydrogenase and related dehydrogenases
18*	0.015	COG0010 [E]	Arginase/agmatinase/formimionoglutamate hydrolase, arginase family
19*	0.014	COG2188 [K]	Transcriptional regulators
20*	0.014	COG2303 [E]	Choline dehydrogenase and related flavoproteins
21*	0.014	COG2186 [K]	Transcriptional regulators
22*	0.013	COG0028 [E,H]	Thiamine pyrophosphate-requiring enzymes [acetolactate synthase, pyruvate dehydrogenase (cytochrome), glyoxylate carboligase, phosphonopyruvate decarboxylase]
23*	0.013	COG1670 [J]	Acetyltransferases, including N-acetylases of ribosomal proteins
24*	0.012	COG0365 [I]	Acyl-coenzyme A synthetases/AMP-(fatty) acid ligases
25	0.011	COG0609 [P]	ABC-type Fe3+-siderophore transport system, permease component
26*	0.011	COG2423 [E]	Predicted ornithine cyclodeaminase, mucrystallin homolog
27*	0.010	COG3938 [E]	Proline racemase

Table S 118: Module 79 – with rank 73 according to estimated information content.

Rank	Probability	OG	Description
1*	0.053	COG0451 [M,G]	Nucleoside-diphosphate-sugar epimerases
2*	0.051	COG0491 [R]	Zn-dependent hydrolases, including glyoxylases
3*	0.042	COG1884 [I]	Methylmalonyl-CoA mutase, N-terminal domain/subunit
4*	0.041	COG2185 [I]	Methylmalonyl-CoA mutase, C-terminal domain/subunit (cobalamin-binding)
5*	0.036	COG1703 [E]	Putative periplasmic protein kinase ArgK and related GTPases of G3E family
6*	0.035	COG0346 [E]	Lactoylglutathione lyase and related lyases
7*	0.031	COG0596 [R]	Predicted hydrolases or acyltransferases (alpha/beta hydrolase superfamily)
8*	0.027	COG4799 [I]	Acetyl-CoA carboxylase, carboxyltransferase component (subunits alpha and beta)
9*	0.025	COG1250 [I]	3-hydroxyacyl-CoA dehydrogenase
10*	0.022	COG0517 [R]	FOG: CBS domain
11*	0.018	COG0553 [K,L]	Superfamily II DNA/RNA helicases, SNF2 family
12*	0.017	COG0247 [C]	Fe-S oxidoreductase
13*	0.017	COG2086 [C]	Electron transfer flavoprotein, beta subunit
14*	0.017	COG0039 [C]	Malate/lactate dehydrogenases
15	0.016	COG2138 [S]	Uncharacterized conserved protein
16*	0.016	COG1063 [E,R]	Threonine dehydrogenase and related Zn-dependent dehydrogenases
17*	0.016	COG0667 [C]	Predicted oxidoreductases (related to aryl-alcohol dehydrogenases)
18*	0.015	COG0846 [K]	NAD-dependent protein deacetylases, SIR2 family
19*	0.015	COG0111 [H,E]	Phosphoglycerate dehydrogenase and related dehydrogenases
20*	0.015	COG0209 [F]	Ribonucleotide reductase, alpha subunit
21*	0.014	COG0661 [R]	Predicted unusual protein kinase
22*	0.014	COG1058 [R]	Predicted nucleotide-utilizing enzyme related to molybdopterin-biosynthesis enzyme MoeA
23*	0.013	COG0750 [M]	Predicted membrane-associated Zn-dependent proteases 1
24*	0.011	COG0043 [H]	3-polyprenyl-4-hydroxybenzoate decarboxylase and related decarboxylases
25*	0.011	COG0822 [C]	NifU homolog involved in Fe-S cluster formation
26*	0.011	COG1032 [C]	Fe-S oxidoreductase
27*	0.010	COG1226 [P]	Kef-type K+ transport systems, predicted NAD-binding component

Table S 119: Module 114 – with rank 74 according to estimated information content.

Rank	Probability	OG	Description
1*	0.070	COG0438 [M]	Glycosyltransferase
2*	0.056	COG0463 [M]	Glycosyltransferases involved in cell wall biogenesis
3*	0.052	COG0745 [T,K]	Response regulators consisting of a CheY-like receiver domain and a winged-helix DNA-binding domain
4*	0.049	COG1028 [I,Q,R]	Dehydrogenases with different specificities (related to short-chain alcohol dehydrogenases)
5*	0.047	COG0642 [T]	Signal transduction histidine kinase
6	0.045	COG0714 [R]	MoxR-like ATPases
7*	0.036	COG0304 [I,Q]	3-oxoacyl-(acyl-carrier-protein) synthase
8	0.036	COG2304 [R]	Uncharacterized protein containing a von Willebrand factor type A (vWA) domain
9	0.030	COG1721 [R]	Uncharacterized conserved protein (some members contain a von Willebrand factor type A (vWA) domain)
10*	0.018	COG0464 [O]	ATPases of the AAA+ class
11*	0.018	COG0526 [O,C]	Thiol-disulfide isomerase and thioredoxins
12*	0.017	COG0607 [P]	Rhodanese-related sulfurtransferase
13	0.015	COG0308 [E]	Aminopeptidase N
14*	0.014	COG0489 [D]	ATPases involved in chromosome partitioning
15*	0.014	COG0514 [L]	Superfamily II DNA helicase
16	0.014	COG0178 [L]	Excinuclease ATPase subunit
17*	0.013	COG2148 [M]	Sugar transferases involved in lipopolysaccharide synthesis
18	0.012	COG0668 [M]	Small-conductance mechanosensitive channel
19*	0.012	COG0204 [I]	1-acyl-sn-glycerol-3-phosphate acyltransferase

Table S 120: Module 137 – with rank 76 according to estimated information content.

Rank	Probability	OG	Description
1*	0.061	COG0247 [C]	Fe-S oxidoreductase
2*	0.042	COG0517 [R]	FOG: CBS domain
3*	0.041	COG0243 [C]	Anaerobic dehydrogenases, typically selenocysteine-containing
4*	0.041	COG0437 [C]	Fe-S-cluster-containing hydrogenase components 1
5	0.035	COG0640 [K]	Predicted transcriptional regulators
6*	0.028	COG0277 [C]	FAD/FMN-containing dehydrogenases
7*	0.022	COG0589 [T]	Universal stress protein UspA and related nucleotide-binding proteins
8*	0.020	COG1053 [C]	Succinate dehydrogenase/fumarate reductase, flavoprotein subunit
9	0.019	COG0730 [R]	Predicted permeases
10*	0.016	COG2221 [C]	Dissimilatory sulfite reductase (desulfoviridin), alpha and beta subunits
11*	0.016	COG0365 [I]	Acyl-coenzyme A synthetases/AMP-(fatty) acid ligases
12	0.015	COG0489 [D]	ATPases involved in chromosome partitioning
13*	0.014	COG0071 [O]	Molecular chaperone (small heat shock protein)
14*	0.014	COG0805 [U]	Sec-independent protein secretion pathway component TatC
15*	0.014	COG1146 [C]	Ferredoxin
16*	0.013	COG0007 [H]	Uroporphyrinogen-III methylase
17	0.013	COG1055 [P]	Na+/H+ antiporter NhaD and related arsenite permeases
18	0.013	COG0577 [V]	ABC-type antimicrobial peptide transport system, permease component
19*	0.012	COG0145 [E,Q]	N-methylhydantoinase A/acetone carboxylase, beta subunit
20*	0.012	COG0479 [C]	Succinate dehydrogenase/fumarate reductase, Fe-S protein subunit
21*	0.012	COG0123 [B,Q]	Deacetylases, including yeast histone deacetylase and acetoin utilization protein
22	0.012	COG0428 [P]	Predicted divalent heavy-metal cations transporter
23	0.012	COG1136 [V]	ABC-type antimicrobial peptide transport system, ATPase component
24*	0.012	COG2920 [P]	Dissimilatory sulfite reductase (desulfoviridin), gamma subunit
25*	0.011	COG2181 [C]	Nitrate reductase gamma subunit
26	0.011	COG0616 [O,U]	Periplasmic serine proteases (ClpP class)
27	0.011	COG0011 [S]	Uncharacterized conserved protein
28	0.010	COG0704 [P]	Phosphate uptake regulator
29	0.010	COG2210 [S]	Uncharacterized conserved protein
30*	0.010	COG0075 [E]	Serine-pyruvate aminotransferase/archaeal aspartate aminotransferase
31	0.010	COG2110 [R]	Predicted phosphatase homologous to the C-terminal domain of histone macroH2A1

Table S 121: Module 14 – with rank 77 according to estimated information content.

Rank	Probability	OG	Description
1*	0.024	COG0188 [L]	Type IIA topoisomerase (DNA gyrase/topo II, topoisomerase IV), A subunit
2*	0.024	COG0564 [J]	Pseudouridylate synthases, 23S RNA-specific
3*	0.024	COG0187 [L]	Type IIA topoisomerase (DNA gyrase/topo II, topoisomerase IV), B subunit
4*	0.017	COG0542 [O]	ATPases with chaperone activity, ATP-binding subunit
5*	0.016	COG0073 [R]	EMAP domain
6*	0.015	COG0039 [C]	Malate/lactate dehydrogenases
7*	0.015	COG0507 [L]	ATP-dependent exoDNAse (exonuclease V), alpha subunit - helicase superfamily I member
8*	0.014	COG0036 [G]	Pentose-5-phosphate-3-epimerase
9*	0.014	COG0557 [K]	Exoribonuclease R
10*	0.013	COG0180 [J]	Tryptophanyl-tRNA synthetase
11*	0.013	COG1190 [J]	Lysyl-tRNA synthetase (class II)
12*	0.013	COG0486 [R]	Predicted GTPase
13*	0.013	COG0231 [J]	Translation elongation factor P (EF-P)/translation initiation factor 5A (eIF-5A)
14*	0.013	COG0445 [D]	NAD/FAD-utilizing enzyme apparently involved in cell division
15*	0.013	COG0154 [J]	Asp-tRNAAsn/Glu-tRNAGln amidotransferase A subunit and related amidases
16	0.013	COG0533 [O]	Metal-dependent proteases with possible chaperone activity
17*	0.013	COG0148 [G]	Enolase
18	0.013	COG0782 [K]	Transcription elongation factor
19*	0.013	COG0220 [R]	Predicted S-adenosylmethionine-dependent methyltransferase
20	0.012	COG0216 [J]	Protein chain release factor A
21*	0.012	COG1080 [G]	Phosphoenolpyruvate-protein kinase (PTS system EI component in bacteria)
22	0.012	COG0858 [J]	Ribosome-binding factor A
23*	0.012	COG0219 [J]	Predicted rRNA methylase (SpoU class)
24*	0.012	COG0173 [J]	Aspartyl-tRNA synthetase
25*	0.012	COG0692 [L]	Uracil DNA glycosylase
26*	0.012	COG0594 [J]	RNase P protein component
27*	0.012	COG0166 [G]	Glucose-6-phosphate isomerase
28*	0.012	COG0080 [J]	Ribosomal protein L11

29*	0.012	COG0469 [G]	Pyruvate kinase
30*	0.012	COG0211 [J]	Ribosomal protein L27
31*	0.012	COG0353 [L]	Recombinational DNA repair protein (RecF pathway)
32*	0.012	COG0333 [J]	Ribosomal protein L32
33*	0.012	COG0742 [L]	N6-adenine-specific methylase
34*	0.012	COG0736 [I]	Phosphopantetheinyl transferase (holo-ACP synthase)
35*	0.012	COG0205 [G]	6-phosphofructokinase
36*	0.012	COG0481 [M]	Membrane GTPase LepA
37	0.011	COG0009 [J]	Putative translation factor (SUA5)
38*	0.011	COG0233 [J]	Ribosome recycling factor
39*	0.011	COG0262 [H]	Dihydrofolate reductase
40*	0.011	COG1187 [J]	16S rRNA uridine-516 pseudouridylate synthase and related pseudouridylate synthases
41*	0.011	COG1925 [G]	Phosphotransferase system, HPr-related proteins
42	0.011	COG0828 [J]	Ribosomal protein S21
43*	0.011	COG1420 [K]	Transcriptional regulator of heat shock gene
44	0.011	COG0775 [F]	Nucleoside phosphorylase
45*	0.011	COG0064 [J]	Asp-tRNAAsn/Glu-tRNAGln amidotransferase B subunit (PET112 homolog)
46*	0.011	COG0194 [F]	Guanylate kinase
47*	0.011	COG0197 [J]	Ribosomal protein L16/L10E
48*	0.011	COG0237 [H]	Dephospho-CoA kinase
49*	0.011	COG0443 [O]	Molecular chaperone
50*	0.011	COG0322 [L]	Nuclease subunit of the excinuclease complex
51*	0.011	COG0162 [J]	Tyrosyl-tRNA synthetase
52*	0.011	COG0060 [J]	Isoleucyl-tRNA synthetase
53*	0.011	COG0149 [G]	Triosephosphate isomerase
54	0.011	COG0327 [S]	Uncharacterized conserved protein
55*	0.011	COG0416 [I]	Fatty acid/phospholipid biosynthesis enzyme
56*	0.011	COG0190 [H]	5,10-methylene-tetrahydrofolate dehydrogenase/Methenyl tetrahydrofolate cyclohydrolase
57*	0.011	COG0631 [T]	Serine/threonine protein phosphatase
58*	0.011	COG0209 [F]	Ribonucleotide reductase, alpha subunit
59*	0.010	COG0470 [L]	ATPase involved in DNA replication
60*	0.010	COG0008 [J]	Glutamyl- and glutaminyl-tRNA synthetases
61*	0.010	COG0636 [C]	F0F1-type ATP synthase, subunit c/Archaeal/vacuolar-type H+-ATPase, subunit K
62*	0.010	COG0504 [F]	CTP synthase (UTP-ammonia lyase)

Table S 122: Module 27 – with rank 79 according to estimated information content.

Rank	Probability	OG	Description
1*	0.020	COG0085 [K]	DNA-directed RNA polymerase, beta subunit/140 kD subunit
2*	0.019	COG0037 [D]	Predicted ATPase of the PP-loop superfamily implicated in cell cycle control
3	0.018	COG0492 [O]	Thioredoxin reductase
4*	0.016	COG0093 [J]	Ribosomal protein L14
5*	0.015	COG0094 [J]	Ribosomal protein L5
6*	0.015	COG0048 [J]	Ribosomal protein S12
7*	0.015	COG0126 [G]	3-phosphoglycerate kinase
8*	0.014	COG0441 [J]	Threonyl-tRNA synthetase
9*	0.014	COG0096 [J]	Ribosomal protein S8
10*	0.014	COG0449 [M]	Glucosamine 6-phosphate synthetase, contains amidotransferase and phosphosugar isomerase domains
11*	0.014	COG0088 [J]	Ribosomal protein L4
12*	0.014	COG0186 [J]	Ribosomal protein S17
13*	0.014	COG0215 [J]	Cysteinyl-tRNA synthetase
14*	0.014	COG0681 [U]	Signal peptidase I
15*	0.013	COG0244 [J]	Ribosomal protein L10
16	0.013	COG0343 [J]	Queuine/archaeosine tRNA-ribosyltransferase
17*	0.013	COG0060 [J]	Isoleucyl-tRNA synthetase
18*	0.013	COG0361 [J]	Translation initiation factor 1 (IF-1)
19*	0.013	COG0018 [J]	Arginyl-tRNA synthetase
20*	0.013	COG0185 [J]	Ribosomal protein S19
21*	0.013	COG0103 [J]	Ribosomal protein S9
22*	0.013	COG0012 [J]	Predicted GTPase, probable translation factor
23*	0.013	COG0130 [J]	Pseudouridine synthase
24*	0.013	COG0024 [J]	Methionine aminopeptidase
25*	0.013	COG0097 [J]	Ribosomal protein L6P/L9E
26*	0.013	COG0250 [K]	Transcription antiterminator
27*	0.013	COG0358 [L]	DNA primase (bacterial type)
28*	0.013	COG0495 [J]	Leucyl-tRNA synthetase

29*	0.013	COG0552 [U]	Signal recognition particle GTPase
30*	0.013	COG0592 [L]	DNA polymerase sliding clamp subunit (PCNA homolog)
31*	0.012	COG0112 [E]	Glycine/serine hydroxymethyltransferase
32*	0.012	COG0172 [J]	Seryl-tRNA synthetase
33*	0.012	COG0237 [H]	Dephospho-CoA kinase
34*	0.012	COG0143 [J]	Methionyl-tRNA synthetase
35*	0.012	COG0016 [J]	Phenylalanyl-tRNA synthetase alpha subunit
36	0.012	COG0533 [O]	Metal-dependent proteases with possible chaperone activity
37*	0.012	COG0013 [J]	Alanyl-tRNA synthetase
38*	0.012	COG0092 [J]	Ribosomal protein S3
39*	0.011	COG0098 [J]	Ribosomal protein S5
40*	0.011	COG0184 [J]	Ribosomal protein S15P/S13E
41*	0.011	COG0197 [J]	Ribosomal protein L16/L10E
42*	0.011	COG0087 [J]	Ribosomal protein L3
43*	0.011	COG0089 [J]	Ribosomal protein L23
44*	0.011	COG0200 [J]	Ribosomal protein L15
45*	0.011	COG0105 [F]	Nucleoside diphosphate kinase
46*	0.011	COG0127 [F]	Xanthosine triphosphate pyrophosphatase
47*	0.011	COG0461 [F]	Orotate phosphoribosyltransferase
48*	0.011	COG0072 [J]	Phenylalanyl-tRNA synthetase beta subunit
49*	0.011	COG0284 [F]	Orotidine-5-phosphate decarboxylase
50*	0.011	COG0525 [J]	Valyl-tRNA synthetase
51*	0.011	COG0100 [J]	Ribosomal protein S11
52*	0.011	COG0180 [J]	Tryptophanyl-tRNA synthetase
53*	0.011	COG0442 [J]	Prolyl-tRNA synthetase
54*	0.011	COG0541 [U]	Signal recognition particle GTPase
55*	0.010	COG0081 [J]	Ribosomal protein L1
56*	0.010	COG0102 [J]	Ribosomal protein L13
57*	0.010	COG0162 [J]	Tyrosyl-tRNA synthetase
58*	0.010	COG0256 [J]	Ribosomal protein L18
59*	0.010	COG0202 [K]	DNA-directed RNA polymerase, alpha subunit/40 kD subunit
60*	0.010	COG0258 [L]	5-3 exonuclease (including N-terminal domain of PolI)
61*	0.010	COG0537 [F,G,R]	Diadenosine tetraphosphate (Ap4A) hydrolase and other HIT family hydrolases

Table S 123: Module 25 – with rank 80 according to estimated information content.

Rank	Probability	OG	Description
1*	0.029	COG0513 [L,K,J]	Superfamily II DNA and RNA helicases
2*	0.028	COG0516 [F]	IMP dehydrogenase/GMP reductase
3*	0.025	COG0488 [R]	ATPase components of ABC transporters with duplicated ATPase domains
4	0.022	COG0628 [R]	Predicted permease
5*	0.019	COG0517 [R]	FOG: CBS domain
6*	0.019	COG1760 [E]	L-serine deaminase
7*	0.018	COG0526 [O,C]	Thiol-disulfide isomerase and thioredoxins
8*	0.018	COG2265 [J]	SAM-dependent methyltransferases related to tRNA (uracil-5-)-methyltransferase
9*	0.017	COG0236 [I,Q]	Acyl carrier protein
10*	0.017	COG0772 [D]	Bacterial cell division membrane protein
11*	0.017	COG0225 [O]	Peptide methionine sulfoxide reductase
12*	0.016	COG1109 [G]	Phosphomannomutase
13*	0.016	COG0745 [T,K]	Response regulators consisting of a CheY-like receiver domain and a winged-helix DNA-binding domain
14	0.015	COG0681 [U]	Signal peptidase I
15*	0.014	COG0115 [E,H]	Branched-chain amino acid aminotransferase/4-amino-4-deoxychorismate lyase
16	0.014	COG0569 [P]	K+ transport systems, NAD-binding component
17*	0.013	COG0169 [E]	Shikimate 5-dehydrogenase
18*	0.013	COG1304 [C]	L-lactate dehydrogenase (FMN-dependent) and related alpha-hydroxy acid dehydrogenases
19*	0.012	COG0229 [O]	Conserved domain frequently associated with peptide methionine sulfoxide reductase
20*	0.012	COG0652 [O]	Peptidyl-prolyl cis-trans isomerase (rotamase) - cyclophilin family
21	0.012	COG0497 [L]	ATPase involved in DNA repair
22*	0.012	COG0042 [J]	tRNA-dihydrouridine synthase
23*	0.012	COG0518 [F]	GMP synthase - Glutamine amidotransferase domain
24	0.011	COG1217 [T]	Predicted membrane GTPase involved in stress response
25*	0.011	COG0796 [M]	Glutamate racemase
26*	0.011	COG1181 [M]	D-alanine-D-alanine ligase and related ATP-grasp enzymes
27	0.011	COG1488 [H]	Nicotinic acid phosphoribosyltransferase
28	0.011	COG2262 [R]	GTPases

29*	0.011	COG0285 [H]	Folylpolyglutamate synthase
30*	0.011	COG0299 [F]	Folate-dependent phosphoribosylglycinamide formyltransferase PurN
31*	0.011	COG0207 [F]	Thymidylate synthase
32	0.011	COG0340 [H]	Biotin-(acetyl-CoA carboxylase) ligase
33*	0.011	COG0519 [F]	GMP synthase, PP-ATPase domain/subunit
34	0.010	COG1066 [O]	Predicted ATP-dependent serine protease
35*	0.010	COG0711 [C]	F0F1-type ATP synthase, subunit b
36*	0.010	COG1187 [J]	16S rRNA uridine-516 pseudouridylate synthase and related pseudouridylate synthases
37*	0.010	COG1207 [M]	N-acetylglucosamine-1-phosphate uridyltransferase (contains nucleotidyltransferase and I-patch acetyltransferase domains)
38	0.010	COG1970 [M]	Large-conductance mechanosensitive channel
39*	0.010	COG0770 [M]	UDP-N-acetylmuramyl pentapeptide synthase
40*	0.010	COG1758 [K]	DNA-directed RNA polymerase, subunit K/omega

Table S 124: Module 121 – with rank 81 according to estimated information content.

Rank	Probability	OG	Description
1*	0.156	COG0477 [G,E,P,R]	Permeases of the major facilitator superfamily
2*	0.046	COG0531 [E]	Amino acid transporters
3*	0.028	COG1131 [V]	ABC-type multidrug transport system, ATPase component
4*	0.019	COG0365 [I]	Acyl-coenzyme A synthetases/AMP-(fatty) acid ligases
5*	0.017	COG1064 [R]	Zn-dependent alcohol dehydrogenases
6*	0.016	COG1028 [I,Q,R]	Dehydrogenases with different specificities (related to short-chain alcohol dehydrogenases)
7*	0.014	COG0183 [I]	Acetyl-CoA acetyltransferase
8*	0.013	COG0425 [O]	Predicted redox protein, regulator of disulfide bond formation
9*	0.013	COG1012 [C]	NAD-dependent aldehyde dehydrogenases
10*	0.013	COG4948 [M,R]	L-alanine-DL-glutamate epimerase and related enzymes of enolase superfamily
11*	0.012	COG0843 [C]	Heme/copper-type cytochrome/quinol oxidases, subunit 1
12*	0.012	COG0438 [M]	Glycosyltransferase
13*	0.012	COG0277 [C]	FAD/FMN-containing dehydrogenases
14	0.012	COG0730 [R]	Predicted permeases
15*	0.012	COG2025 [C]	Electron transfer flavoprotein, alpha subunit
16*	0.011	COG0640 [K]	Predicted transcriptional regulators
17*	0.011	COG0446 [R]	Uncharacterized NAD(FAD)-dependent dehydrogenases
18	0.011	COG1225 [O]	Peroxiredoxin
19*	0.011	COG1290 [C]	Cytochrome b subunit of the bc complex
20	0.011	COG3413 [R]	Predicted DNA binding protein
21*	0.010	COG0247 [C]	Fe-S oxidoreductase
22*	0.010	COG0723 [C]	Rieske Fe-S protein
23	0.010	COG1192 [D]	ATPases involved in chromosome partitioning

Table S 125: Module 134 – with rank 82 according to estimated information content.

Rank	Probability	OG	Description
1*	0.021	COG0142 [H]	Geranylgeranyl pyrophosphate synthase
2*	0.015	COG1186 [J]	Protein chain release factor B
3*	0.015	COG0564 [J]	Pseudouridylate synthases, 23S RNA-specific
4*	0.015	COG0188 [L]	Type IIA topoisomerase (DNA gyrase/topo II, topoisomerase IV), A subunit
5	0.014	COG0617 [J]	tRNA nucleotidyltransferase/poly(A) polymerase
6*	0.013	COG0653 [U]	Preprotein translocase subunit SecA (ATPase, RNA helicase)
7*	0.013	COG0621 [J]	2-methylthioadenine synthetase
8*	0.012	COG0539 [J]	Ribosomal protein S1
9*	0.012	COG0231 [J]	Translation elongation factor P (EF-P)/translation initiation factor 5A (eIF-5A)
10*	0.012	COG0504 [F]	CTP synthase (UTP-ammonia lyase)
11*	0.012	COG0769 [M]	UDP-N-acetylmuramyl tripeptide synthase
12*	0.011	COG0037 [D]	Predicted ATPase of the PP-loop superfamily implicated in cell cycle control
13	0.011	COG1197 [L,K]	Transcription-repair coupling factor (superfamily II helicase)
14*	0.011	COG0542 [O]	ATPases with chaperone activity, ATP-binding subunit
15*	0.011	COG0743 [I]	1-deoxy-D-xylulose 5-phosphate reductoisomerase
16*	0.011	COG0105 [F]	Nucleoside diphosphate kinase
17*	0.011	COG1104 [E]	Cysteine sulfinate desulfinase/cysteine desulfurase and related enzymes
18*	0.011	COG0707 [M]	UDP-N-acetylglucosamine:LPS N-acetylglucosamine transferase
19	0.011	COG0799 [S]	Uncharacterized homolog of plant Iojap protein
20	0.011	COG0216 [J]	Protein chain release factor A
21*	0.011	COG0342 [U]	Preprotein translocase subunit SecD
22*	0.011	COG0459 [O]	Chaperonin GroEL (HSP60 family)
23*	0.011	COG0481 [M]	Membrane GTPase LepA
24	0.011	COG0181 [H]	Porphobilinogen deaminase
25	0.011	COG0817 [L]	Holliday junction resolvasome, endonuclease subunit
26	0.011	COG0766 [M]	UDP-N-acetylglucosamine enolpyruvyl transferase
27*	0.011	COG0742 [L]	N6-adenine-specific methylase
28	0.010	COG0173 [J]	Aspartyl-tRNA synthetase

29*	0.010	COG0341 [U]	Preprotein translocase subunit SecF
30*	0.010	COG2226 [H]	Methylase involved in ubiquinone/menaquinone biosynthesis
31*	0.010	COG1825 [J]	Ribosomal protein L25 (general stress protein Ctc)
32	0.010	COG0009 [J]	Putative translation factor (SUA5)
33*	0.010	COG0211 [J]	Ribosomal protein L27
34	0.010	COG0177 [L]	Predicted EndoIII-related endonuclease
35*	0.010	COG0180 [J]	Tryptophanyl-tRNA synthetase
36	0.010	COG0801 [H]	7,8-dihydro-6-hydroxymethylpterin-pyrophosphokinase
37	0.010	COG0728 [R]	Uncharacterized membrane protein, putative virulence factor
38*	0.010	COG0080 [J]	Ribosomal protein L11
39*	0.010	COG0008 [J]	Glutamyl- and glutaminyl-tRNA synthetases
40*	0.010	COG0770 [M]	UDP-N-acetylmuramyl pentapeptide synthase
41*	0.010	COG0233 [J]	Ribosome recycling factor
42*	0.010	COG1947 [I]	4-diphosphocytidyl-2C-methyl-D-erythritol 2-phosphate synthase
43	0.010	COG1198 [L]	Primosomal protein N (replication factor Y) - superfamily II helicase
44*	0.010	COG1496 [S]	Uncharacterized conserved protein

Table S 126: Module 54 – with rank 84 according to estimated information content.

Rank	Probability	OG	Description
1*	0.028	COG0513 [L,K,J]	Superfamily II DNA and RNA helicases
2	0.027	COG0782 [K]	Transcription elongation factor
3	0.023	COG0491 [R]	Zn-dependent hydrolases, including glyoxylases
4	0.023	COG0626 [E]	Cystathionine beta-lyases/cystathionine gamma-synthases
5	0.021	COG0568 [K]	DNA-directed RNA polymerase, sigma subunit (sigma70/sigma32)
6*	0.020	COG1199 [K,L]	Rad3-related DNA helicases
7	0.019	COG0312 [R]	Predicted Zn-dependent proteases and their inactivated homologs
8*	0.019	COG1278 [K]	Cold shock proteins
9*	0.018	COG0488 [R]	ATPase components of ABC transporters with duplicated ATPase domains
10	0.017	COG1018 [C]	Flavodoxin reductases (ferredoxin-NADPH reductases) family 1
11*	0.016	COG0175 [E,H]	3-phosphoadenosine 5-phosphosulfate sulfotransferase (PAPS reductase)/FAD synthetase and related enzymes
12	0.016	COG0548 [E]	Acetylglutamate kinase
13	0.016	COG1605 [E]	Chorismate mutase
14*	0.015	COG0729 [M]	Outer membrane protein
15	0.015	COG2132 [Q]	Putative multicopper oxidases
16	0.015	COG0347 [E]	Nitrogen regulatory protein PII
17*	0.014	COG0652 [O]	Peptidyl-prolyl cis-trans isomerase (rotamase) - cyclophilin family
18	0.014	COG2003 [L]	DNA repair proteins
19	0.013	COG1734 [T]	DnaK suppressor protein
20*	0.013	COG0209 [F]	Ribonucleotide reductase, alpha subunit
21*	0.013	COG2911 [S]	Uncharacterized protein conserved in bacteria
22	0.013	COG1674 [D]	DNA segregation ATPase FtsK/SpoIIIE and related proteins
23	0.013	COG0624 [E]	Acetylornithine deacetylase/Succinyl-diaminopimelate desuccinylase and related deacylases
24	0.012	COG1845 [C]	Heme/copper-type cytochrome/quinol oxidase, subunit 3
25	0.012	COG0567 [C]	2-oxoglutarate dehydrogenase complex, dehydrogenase (E1) component, and related enzymes
26	0.012	COG0547 [E]	Anthranilate phosphoribosyltransferase
27	0.012	COG1281 [O]	Disulfide bond chaperones of the HSP33 family
28	0.012	COG1995 [H]	Pyridoxal phosphate biosynthesis protein

29	0.011	COG0259 [H]	Pyridoxamine-phosphate oxidase
30*	0.011	COG0514 [L]	Superfamily II DNA helicase
31	0.011	COG0308 [E]	Aminopeptidase N
32	0.011	COG1327 [K]	Predicted transcriptional regulator, consists of a Zn-ribbon and ATP-cone domains
33*	0.011	COG1607 [I]	Acyl-CoA hydrolase
34	0.011	COG1080 [G]	Phosphoenolpyruvate-protein kinase (PTS system EI component in bacteria)
35	0.011	COG1225 [O]	Peroxiredoxin
36	0.011	COG2844 [O]	UTP:GlnB (protein PII) uridylyltransferase
37	0.011	COG0755 [O]	ABC-type transport system involved in cytochrome c biogenesis, permease component
38	0.010	COG2062 [T]	Phosphohistidine phosphatase SixA
39	0.010	COG0109 [O]	Polyprenyltransferase (cytochrome oxidase assembly factor)
40	0.010	COG1660 [R]	Predicted P-loop-containing kinase

Table S 127: Module 122 – with rank 86 according to estimated information content.

Rank	Probability	OG	Description
1*	0.120	COG5001 [T]	Predicted signal transduction protein containing a membrane domain, an EAL and a GGDEF domain
2*	0.080	COG2202 [T]	FOG: PAS/PAC domain
3*	0.033	COG3706 [T]	Response regulator containing a CheY-like receiver domain and a GGDEF domain
4*	0.032	COG0664 [T]	cAMP-binding proteins - catabolite gene activator and regulatory subunit of cAMP-dependent protein kinases
5	0.031	COG0841 [V]	Cation/multidrug efflux pump
6*	0.022	COG1226 [P]	Kef-type K+ transport systems, predicted NAD-binding component
7*	0.021	COG0348 [C]	Polyferredoxin
8*	0.019	COG0004 [P]	Ammonia permease
9*	0.018	COG2206 [T]	HD-GYP domain
10	0.017	COG0513 [L,K,J]	Superfamily II DNA and RNA helicases
11	0.015	COG1459 [N,U]	Type II secretory pathway, component PulF
12	0.014	COG1033 [R]	Predicted exporters of the RND superfamily
13	0.012	COG0446 [R]	Uncharacterized NAD(FAD)-dependent dehydrogenases
14*	0.012	COG1639 [T]	Predicted signal transduction protein
15	0.011	COG0826 [O]	Collagenase and related proteases
16*	0.010	COG0069 [E]	Glutamate synthase domain 2
17	0.010	COG0189 [H,J]	Glutathione synthase/Ribosomal protein S6 modification enzyme (glutaminyl transferase)

Table S 128: Module 32 – with rank 88 according to estimated information content.

Rank	Probability	OG	Description
1*	0.053	COG0745 [T,K]	Response regulators consisting of a CheY-like receiver domain and a winged-helix DNA-binding domain
2*	0.050	COG0515 [R,T,K,L]	Serine/threonine protein kinase
3*	0.030	COG0265 [O]	Trypsin-like serine proteases, typically periplasmic, contain C-terminal PDZ domain
4*	0.024	COG2197 [T,K]	Response regulator containing a CheY-like receiver domain and an HTH DNA-binding domain
5*	0.023	COG0604 [C,R]	NADPH:quinone reductase and related Zn-dependent oxidoreductases
6*	0.021	COG1022 [I]	Long-chain acyl-CoA synthetases (AMP-forming)
7*	0.019	COG0210 [L]	Superfamily I DNA and RNA helicases
8*	0.019	COG1249 [C]	Pyruvate/2-oxoglutarate dehydrogenase complex, dihydrolipoamide dehydrogenase (E3) component, and related enzymes
9*	0.018	COG1716 [T]	FOG: FHA domain
10*	0.017	COG0366 [G]	Glycosidases
11*	0.017	COG0204 [I]	1-acyl-sn-glycerol-3-phosphate acyltransferase
12*	0.016	COG1651 [O]	Protein-disulfide isomerase
13	0.015	COG0719 [O]	ABC-type transport system involved in Fe-S cluster assembly, permease component
14*	0.015	COG0631 [T]	Serine/threonine protein phosphatase
15*	0.015	COG1409 [R]	Predicted phosphohydrolases
16*	0.014	COG0624 [E]	Acetylornithine deacetylase/Succinyl-diaminopimelate desuccinylase and related deacylases
17*	0.013	COG1940 [K,G]	Transcriptional regulator/sugar kinase
18*	0.013	COG0558 [I]	Phosphatidylglycerophosphate synthase
19*	0.012	COG1597 [I,R]	Sphingosine kinase and enzymes related to eukaryotic diacylglycerol kinase
20*	0.012	COG0542 [O]	ATPases with chaperone activity, ATP-binding subunit
21*	0.012	COG0652 [O]	Peptidyl-prolyl cis-trans isomerase (rotamase) - cyclophilin family
22	0.011	COG1232 [H]	Protoporphyrinogen oxidase
23*	0.011	COG0154 [J]	Asp-tRNAAsn/Glu-tRNAGln amidotransferase A subunit and related amidases
24*	0.010	COG0024 [J]	Methionine aminopeptidase
25*	0.010	COG1196 [D]	Chromosome segregation ATPases

Table S 129: Module 9 – with rank 89 according to estimated information content.

Rank	Probability	OG	Description
1*	0.038	COG0642 [T]	Signal transduction histidine kinase
2*	0.037	COG0666 [R]	FOG: Ankyrin repeat
3	0.036	COG3451 [U]	Type IV secretory pathway, VirB4 components
4*	0.031	COG0330 [O]	Membrane protease subunits, stomatin/prohibitin homologs
5	0.029	COG3505 [U]	Type IV secretory pathway, VirD4 components
6	0.024	COG3504 [U]	Type IV secretory pathway, VirB9 components
7	0.021	COG3704 [U]	Type IV secretory pathway, VirB6 components
8	0.018	COG1192 [D]	ATPases involved in chromosome partitioning
9	0.017	COG3736 [U]	Type IV secretory pathway, component VirB8
10	0.017	COG2948 [U]	Type IV secretory pathway, VirB10 components
11	0.016	COG0630 [N,U]	Type IV secretory pathway, VirB11 components, and related ATPases involved in archaeal flagella biosynthesis
12	0.016	NOG10461 [U]	Pilus assembly protein
13	0.016	COG0492 [O]	Thioredoxin reductase
14	0.016	COG0582 [L]	Integrase
15	0.015	COG0242 [J]	N-formylmethionyl-tRNA deformylase
16*	0.015	COG0688 [I]	Phosphatidylserine decarboxylase
17*	0.014	COG0596 [R]	Predicted hydrolases or acyltransferases (alpha/beta hydrolase superfamily)
18	0.014	NOG12793 [S]	Calcium ion binding protein
19	0.014	COG1200 [L,K]	RecG-like helicase
20*	0.013	COG0055 [C]	F0F1-type ATP synthase, beta subunit
21	0.013	COG1226 [P]	Kef-type K+ transport systems, predicted NAD-binding component
22*	0.012	COG0574 [G]	Phosphoenolpyruvate synthase/pyruvate phosphate dikinase
23*	0.012	COG1538 [M,U]	Outer membrane protein
24	0.012	NOG10358 [S]	Conjugative transfer protein
25	0.011	COG0621 [J]	2-methylthioadenine synthetase
26	0.011	NOG10550 [S]	Pilus assembly protein
27	0.011	NOG10907 [-]	Conjugal DNA transfer protein
28*	0.011	COG1845 [C]	Heme/copper-type cytochrome/quinol oxidase, subunit 3
29	0.011	COG0849 [D]	Actin-like ATPase involved in cell division
30	0.011	NOG10878 [-]	Pilus assembly protein
31*	0.011	COG0056 [C]	F0F1-type ATP synthase, alpha subunit
32	0.011	COG0606 [O]	Predicted ATPase with chaperone activity
33*	0.011	COG0625 [O]	Glutathione S-transferase
34	0.010	COG1301 [C]	Na+/H+-dicarboxylate symporters
35*	0.010	COG0326 [O]	Molecular chaperone, HSP90 family
36*	0.010	COG0473 [C,E]	Isocitrate/isopropylmalate dehydrogenase
37	0.010	COG0489 [D]	ATPases involved in chromosome partitioning
38*	0.010	COG0843 [C]	Heme/copper-type cytochrome/quinol oxidases, subunit 1
39	0.010	COG1127 [Q]	ABC-type transport system involved in resistance to organic solvents, ATPase component
40*	0.010	COG1520 [S]	FOG: WD40-like repeat
41	0.010	COG1040 [R]	Predicted amidophosphoribosyltransferases
42	0.010	NOG10915 [-]	Assembly protein
43	0.010	COG3702 [U]	Type IV secretory pathway, VirB3 components

Table S 130: Module 98 – with rank 90 according to estimated information content.

Rank	Probability	OG	Description
1*	0.027	COG0388 [R]	Predicted amidohydrolase
2	0.027	COG0735 [P]	Fe2+/Zn2+ uptake regulation proteins
3*	0.026	COG0719 [O]	ABC-type transport system involved in Fe-S cluster assembly, permease component
4*	0.022	COG0661 [R]	Predicted unusual protein kinase
5*	0.022	COG0500 [Q,R]	SAM-dependent methyltransferases
6*	0.019	COG0277 [C]	FAD/FMN-containing dehydrogenases
7	0.017	COG0415 [L]	Deoxyribodipyrimidine photolyase
8	0.016	COG0668 [M]	Small-conductance mechanosensitive channel
9	0.015	COG0524 [G]	Sugar kinases, ribokinase family
10*	0.015	COG0520 [E]	Selenocysteine lyase
11	0.015	COG0038 [P]	Chloride channel protein EriC
12*	0.014	COG0399 [M]	Predicted pyridoxal phosphate-dependent enzyme apparently involved in regulation of cell wall biogenesis
13*	0.014	COG0396 [O]	ABC-type transport system involved in Fe-S cluster assembly, ATPase component
14*	0.014	COG0824 [R]	Predicted thioesterase
15*	0.014	COG0566 [J]	rRNA methylases
16*	0.013	COG0507 [L]	ATP-dependent exoDNAse (exonuclease V), alpha subunit - helicase superfamily I member
17*	0.013	COG2227 [H]	2-polyprenyl-3-methyl-5-hydroxy-6-metoxy-1,4-benzoquinol methylase
18*	0.013	COG2148 [M]	Sugar transferases involved in lipopolysaccharide synthesis
19*	0.013	COG0249 [L]	Mismatch repair ATPase (MutS family)
20*	0.012	COG1249 [C]	Pyruvate/2-oxoglutarate dehydrogenase complex, dihydrolipoamide dehydrogenase (E3) component, and related enzymes
21*	0.012	COG0069 [E]	Glutamate synthase domain 2
22*	0.012	COG1195 [L]	Recombinational DNA repair ATPase (RecF pathway)
23*	0.012	COG0026 [F]	Phosphoribosylaminoimidazole carboxylase (NCAIR synthetase)
24	0.012	COG0717 [F]	Deoxycytidine deaminase
25	0.012	COG1234 [R]	Metal-dependent hydrolases of the beta-lactamase superfamily III
26	0.012	COG0529 [P]	Adenylylsulfate kinase and related kinases
27	0.012	COG1845 [C]	Heme/copper-type cytochrome/quinol oxidase, subunit 3
28*	0.012	COG0557 [K]	Exoribonuclease R

29	0.011	COG0843 [C]	Heme/copper-type cytochrome/quinol oxidases, subunit 1
30*	0.011	COG0188 [L]	Type IIA topoisomerase (DNA gyrase/topo II, topoisomerase IV), A subunit
31	0.011	COG0488 [R]	ATPase components of ABC transporters with duplicated ATPase domains
32	0.011	COG0400 [R]	Predicted esterase
33	0.011	COG0005 [F]	Purine nucleoside phosphorylase
34*	0.011	COG0491 [R]	Zn-dependent hydrolases, including glyoxylases
35*	0.010	COG2166 [R]	SufE protein probably involved in Fe-S center assembly
36	0.010	COG1586 [E]	S-adenosylmethionine decarboxylase
37*	0.010	COG1473 [R]	Metal-dependent amidase/aminoacylase/carboxypeptidase
38	0.010	COG0225 [O]	Peptide methionine sulfoxide reductase
39	0.010	COG0681 [U]	Signal peptidase I

Table S 131: Module 37 – with rank 91 according to estimated information content.

Rank	Probability	OG	Description
1*	0.097	COG0477 [G,E,P,R]	Permeases of the major facilitator superfamily
2*	0.055	COG0583 [K]	Transcriptional regulator
3*	0.029	COG0667 [C]	Predicted oxidoreductases (related to aryl-alcohol dehydrogenases)
4*	0.027	COG1289 [S]	Predicted membrane protein
5*	0.021	COG2197 [T,K]	Response regulator containing a CheY-like receiver domain and an HTH DNA-binding domain
6*	0.020	COG2814 [G]	Arabinose efflux permease
7	0.019	COG0791 [M]	Cell wall-associated hydrolases (invasion-associated proteins)
8*	0.018	COG0671 [I]	Membrane-associated phospholipid phosphatase
9*	0.015	COG0454 [K,R]	Histone acetyltransferase HPA2 and related acetyltransferases
10*	0.014	COG1566 [V]	Multidrug resistance efflux pump
11	0.013	COG1974 [K,T]	SOS-response transcriptional repressors (RecA-mediated autopeptidases)
12*	0.013	COG0778 [C]	Nitroreductase
13*	0.012	COG1502 [I]	Phosphatidylserine/phosphatidylglycerophosphate/cardiolipin synthases and related enzymes
14*	0.012	COG1414 [K]	Transcriptional regulator
15*	0.012	COG1957 [F]	Inosine-uridine nucleoside N-ribohydrolase
16	0.012	COG1925 [G]	Phosphotransferase system, HPr-related proteins

Table S 132: Module 26 – with rank 92 according to estimated information content.

Rank	Probability	OG	Description
1*	0.103	COG0583 [K]	Transcriptional regulator
2*	0.066	COG0642 [T]	Signal transduction histidine kinase
3*	0.051	COG0745 [T,K]	Response regulators consisting of a CheY-like receiver domain and a winged-helix DNA-binding domain
4*	0.032	COG0845 [M]	Membrane-fusion protein
5*	0.020	COG2204 [T]	Response regulator containing CheY-like receiver, AAA-type ATPase, and DNA-binding domains
6*	0.019	COG1280 [E]	Putative threonine efflux protein
7*	0.013	COG0784 [T]	FOG: CheY-like receiver
8*	0.013	COG0243 [C]	Anaerobic dehydrogenases, typically selenocysteine-containing
9*	0.012	COG0304 [I,Q]	3-oxoacyl-(acyl-carrier-protein) synthase
10*	0.012	COG1454 [C]	Alcohol dehydrogenase, class IV
11*	0.011	COG0183 [I]	Acetyl-CoA acetyltransferase
12*	0.011	COG2186 [K]	Transcriptional regulators
13*	0.011	COG1292 [M]	Choline-glycine betaine transporter
14	0.010	COG1278 [K]	Cold shock proteins

Table S 133: Module 96 – with rank 93 according to estimated information content.

Rank	Probability	OG	Description
1*	0.026	COG0526 [O,C]	Thiol-disulfide isomerase and thioredoxins
2*	0.026	COG0612 [R]	Predicted Zn-dependent peptidases
3*	0.025	COG1028 [I,Q,R]	Dehydrogenases with different specificities (related to short-chain alcohol dehydrogenases)
4*	0.024	COG0174 [E]	Glutamine synthetase
5*	0.018	COG0150 [F]	Phosphoribosylaminoimidazole (AIR) synthetase
6*	0.017	COG0054 [H]	Riboflavin synthase beta-chain
7*	0.017	COG0516 [F]	IMP dehydrogenase/GMP reductase
8*	0.017	COG0148 [G]	Enolase
9	0.017	COG0226 [P]	ABC-type phosphate transport system, periplasmic component
10*	0.016	COG0047 [F]	Phosphoribosylformylglycinamidine (FGAM) synthase, glutamine amidotransferase domain
11*	0.015	COG2070 [R]	Dioxygenases related to 2-nitropropane dioxygenase
12*	0.015	COG0299 [F]	Folate-dependent phosphoribosylglycinamide formyltransferase PurN
13*	0.015	COG0151 [F]	Phosphoribosylamine-glycine ligase
14*	0.014	COG0452 [H]	Phosphopantothenoylcysteine synthetase/decarboxylase
15*	0.014	COG0519 [F]	GMP synthase, PP-ATPase domain/subunit
16*	0.014	COG0138 [F]	AICAR transformylase/IMP cyclohydrolase PurH (only IMP cyclohydrolase domain in Aful)
17	0.014	COG0581 [P]	ABC-type phosphate transport system, permease component
18*	0.014	COG0057 [G]	Glyceraldehyde-3-phosphate dehydrogenase/erythrose-4-phosphate dehydrogenase
19*	0.014	COG0036 [G]	Pentose-5-phosphate-3-epimerase
20*	0.014	COG0546 [R]	Predicted phosphatases
21*	0.014	COG1211 [I]	4-diphosphocytidyl-2-methyl-D-erithritol synthase
22*	0.013	COG0518 [F]	GMP synthase - Glutamine amidotransferase domain
23	0.013	COG0312 [R]	Predicted Zn-dependent proteases and their inactivated homologs
24*	0.013	COG0034 [F]	Glutamine phosphoribosylpyrophosphate amidotransferase
25*	0.013	COG0104 [F]	Adenylosuccinate synthase
26*	0.013	COG0611 [H]	Thiamine monophosphate kinase
27*	0.013	COG2022 [H]	Uncharacterized enzyme of thiazole biosynthesis
28*	0.012	COG0149 [G]	Triosephosphate isomerase

29*	0.012	COG0167 [F]	Dihydroorotate dehydrogenase
30*	0.012	COG0542 [O]	ATPases with chaperone activity, ATP-binding subunit
31*	0.012	COG0476 [H]	Dinucleotide-utilizing enzymes involved in molybdopterin and thiamine biosynthesis family 2
32*	0.012	COG0168 [P]	Trk-type K+ transport systems, membrane components
33*	0.011	COG0352 [H]	Thiamine monophosphate synthase
34*	0.011	COG0443 [O]	Molecular chaperone
35*	0.011	COG0807 [H]	GTP cyclohydrolase II
36*	0.011	COG0245 [I]	2C-methyl-D-erythritol 2,4-cyclodiphosphate synthase
37	0.011	COG0573 [P]	ABC-type phosphate transport system, permease component
38*	0.011	COG0044 [F]	Dihydroorotase and related cyclic amidohydrolases
39	0.011	COG0679 [R]	Predicted permeases
40	0.010	COG1115 [E]	Na+/alanine symporter
41	0.010	COG1117 [P]	ABC-type phosphate transport system, ATPase component

Table S 134: Module 15 – with rank 94 according to estimated information content.

Rank	Probability	OG	Description
1*	0.050	COG1024 [I]	Enoyl-CoA hydratase/carnithine racemase
2*	0.046	COG0596 [R]	Predicted hydrolases or acyltransferases (alpha/beta hydrolase superfamily)
3*	0.042	COG1309 [K]	Transcriptional regulator
4	0.042	COG1522 [K]	Transcriptional regulators
5*	0.034	COG1960 [I]	Acyl-CoA dehydrogenases
6*	0.027	COG0789 [K]	Predicted transcriptional regulators
7*	0.026	COG0183 [I]	Acetyl-CoA acetyltransferase
8*	0.021	COG1250 [I]	3-hydroxyacyl-CoA dehydrogenase
9*	0.019	COG0604 [C,R]	NADPH:quinone reductase and related Zn-dependent oxidoreductases
10*	0.019	COG1846 [K]	Transcriptional regulators
11	0.017	COG0697 [G,E,R]	Permeases of the drug/metabolite transporter (DMT) superfamily
12*	0.016	COG2188 [K]	Transcriptional regulators
13	0.014	COG4977 [K]	Transcriptional regulator containing an amidase domain and an AraC-type DNA-binding HTH domain
14*	0.013	COG2303 [E]	Choline dehydrogenase and related flavoproteins
15	0.012	COG0546 [R]	Predicted phosphatases
16*	0.011	COG1171 [E]	Threonine dehydratase
17*	0.011	COG0534 [V]	Na+-driven multidrug efflux pump

Table S 135: Module 50 – with rank 95 according to estimated information content.

Rank	Probability	OG	Description
1*	0.059	COG3250 [G]	Beta-galactosidase/beta-glucuronidase
2*	0.058	COG0642 [T]	Signal transduction histidine kinase
3*	0.024	COG1136 [V]	ABC-type antimicrobial peptide transport system, ATPase component
4*	0.024	COG3525 [G]	N-acetyl-beta-hexosaminidase
5*	0.023	COG1472 [G]	Beta-glucosidase-related glycosidases
6*	0.021	COG3669 [G]	Alpha-L-fucosidase
7*	0.020	COG0526 [O,C]	Thiol-disulfide isomerase and thioredoxins
8*	0.019	COG2755 [E]	Lysophospholipase L1 and related esterases
9*	0.018	COG0524 [G]	Sugar kinases, ribokinase family
10*	0.018	COG1940 [K,G]	Transcriptional regulator/sugar kinase
11*	0.016	COG2197 [T,K]	Response regulator containing a CheY-like receiver domain and an HTH DNA-binding domain
12*	0.016	COG2207 [K]	AraC-type DNA-binding domain-containing proteins
13*	0.015	COG3507 [G]	Beta-xylosidase
14*	0.015	COG1501 [G]	Alpha-glucosidases, family 31 of glycosyl hydrolases
15*	0.014	COG1609 [K]	Transcriptional regulators
16*	0.014	COG0463 [M]	Glycosyltransferases involved in cell wall biogenesis
17*	0.014	COG2017 [G]	Galactose mutarotase and related enzymes
18*	0.014	COG0745 [T,K]	Response regulators consisting of a CheY-like receiver domain and a winged-helix DNA-binding domain
19*	0.013	COG0667 [C]	Predicted oxidoreductases (related to aryl-alcohol dehydrogenases)
20*	0.013	COG5434 [M]	Endopolygalacturonase
21*	0.013	COG0110 [R]	Acetyltransferase (isoleucine patch superfamily)
22*	0.012	COG0657 [I]	Esterase/lipase
23	0.012	COG3533 [S]	Uncharacterized protein conserved in bacteria
24*	0.012	COG1011 [R]	Predicted hydrolase (HAD superfamily)
25	0.012	COG3537 [G]	Putative alpha-1,2-mannosidase
26*	0.011	COG0363 [G]	6-phosphogluconolactonase/Glucosamine-6-phosphate isomerase/deaminase
27	0.011	COG0582 [L]	Integrase
28	0.010	COG0793 [M]	Periplasmic protease
29*	0.010	COG1874 [G]	Beta-galactosidase
30*	0.010	COG3534 [G]	Alpha-L-arabinofuranosidase

Table S 136: Module 130 – with rank 96 according to estimated information content.

Rank	Probability	OG	Description
1*	0.049	COG0589 [T]	Universal stress protein UspA and related nucleotide-binding proteins
2	0.030	COG0668 [M]	Small-conductance mechanosensitive channel
3	0.026	COG0471 [P]	Di- and tricarboxylate transporters
4*	0.023	COG1226 [P]	Kef-type K+ transport systems, predicted NAD-binding component
5	0.022	COG0582 [L]	Integrase
6*	0.021	COG2111 [P]	Multisubunit Na+/H+ antiporter, MnhB subunit
7*	0.021	COG1278 [K]	Cold shock proteins
8	0.020	COG1292 [M]	Choline-glycine betaine transporter
9*	0.019	COG1006 [P]	Multisubunit Na+/H+ antiporter, MnhC subunit
10*	0.018	COG0651 [C,P]	Formate hydrogenlyase subunit 3/Multisubunit Na+/H+ antiporter, MnhD subunit
11*	0.018	COG2217 [P]	Cation transport ATPase
12*	0.018	COG0591 [E,R]	Na+/proline symporter
13*	0.017	COG1009 [C,P]	NADH:ubiquinone oxidoreductase subunit 5 (chain L)/Multisubunit Na+/H+ antiporter, MnhA subunit
14*	0.017	COG1863 [P]	Multisubunit Na+/H+ antiporter, MnhE subunit
15	0.016	COG0518 [F]	GMP synthase - Glutamine amidotransferase domain
16*	0.015	COG0702 [M,G]	Predicted nucleoside-diphosphate-sugar epimerases
17	0.015	COG0534 [V]	Na+-driven multidrug efflux pump
18*	0.015	COG1320 [P]	Multisubunit Na+/H+ antiporter, MnhG subunit
19*	0.014	COG0415 [L]	Deoxyribodipyrimidine photolyase
20	0.014	COG0530 [P]	Ca2+/Na+ antiporter
21	0.014	COG0628 [R]	Predicted permease
22	0.014	COG1414 [K]	Transcriptional regulator
23	0.013	COG0398 [S]	Uncharacterized conserved protein
24*	0.013	COG2133 [G]	Glucose/sorbosone dehydrogenases
25	0.012	COG2323 [S]	Predicted membrane protein
26*	0.012	COG0168 [P]	Trk-type K+ transport systems, membrane components
27	0.012	COG1011 [R]	Predicted hydrolase (HAD superfamily)
28	0.012	COG0633 [C]	Ferredoxin
29*	0.012	COG2212 [P]	Multisubunit Na+/H+ antiporter, MnhF subunit
30	0.011	COG2128 [S]	Uncharacterized conserved protein
31*	0.011	COG0160 [E]	4-aminobutyrate aminotransferase and related aminotransferases
32	0.011	COG0435 [O]	Predicted glutathione S-transferase
33*	0.011	COG0733 [R]	Na+-dependent transporters of the SNF family
34*	0.011	COG1018 [C]	Flavodoxin reductases (ferredoxin-NADPH reductases) family 1

Table S 137: Module 153 – with rank 98 according to estimated information content.

Rank	Probability	OG	Description
1*	0.067	COG1028 [I,Q,R]	Dehydrogenases with different specificities (related to short-chain alcohol dehydrogenases)
2*	0.040	COG1404 [O]	Subtilisin-like serine proteases
3*	0.038	COG0526 [O,C]	Thiol-disulfide isomerase and thioredoxins
4*	0.032	COG2197 [T,K]	Response regulator containing a CheY-like receiver domain and an HTH DNA-binding domain
5*	0.030	COG0508 [C]	Pyruvate/2-oxoglutarate dehydrogenase complex, dihydrolipoamide acyltransferase (E2) component, and related enzymes
6*	0.022	COG2120 [S]	Uncharacterized proteins, LmbE homologs
7*	0.022	COG1233 [Q]	Phytoene dehydrogenase and related proteins
8*	0.021	COG1012 [C]	NAD-dependent aldehyde dehydrogenases
9*	0.020	COG0612 [R]	Predicted Zn-dependent peptidases
10*	0.019	COG4585 [T]	Signal transduction histidine kinase
11	0.019	COG1680 [V]	Beta-lactamase class C and other penicillin binding proteins
12*	0.018	COG0022 [C]	Pyruvate/2-oxoglutarate dehydrogenase complex, dehydrogenase (E1) component, eukaryotic type, beta subunit
13*	0.017	COG0639 [T]	Diadenosine tetraphosphatase and related serine/threonine protein phosphatases
14*	0.015	COG1011 [R]	Predicted hydrolase (HAD superfamily)
15*	0.015	COG1071 [C]	Pyruvate/2-oxoglutarate dehydrogenase complex, dehydrogenase (E1) component, eukaryotic type, alpha subunit
16*	0.015	COG0492 [O]	Thioredoxin reductase
17*	0.015	COG1506 [E]	Dipeptidyl aminopeptidases/acylaminoacyl-peptidases
18	0.014	COG0520 [E]	Selenocysteine lyase
19	0.014	COG2041 [R]	Sulfite oxidase and related enzymes
20*	0.013	COG1073 [R]	Hydrolases of the alpha/beta superfamily
21*	0.011	COG0010 [E]	Arginase/agmatinase/formimionoglutamate hydrolase, arginase family
22*	0.011	COG0664 [T]	cAMP-binding proteins - catabolite gene activator and regulatory subunit of cAMP-dependent protein kinases
23*	0.011	COG1999 [R]	Uncharacterized protein SCO1/SenC/PrrC, involved in biogenesis of respiratory and photosynthetic systems
24*	0.010	COG1562 [I]	Phytoene/squalene synthetase

Table S 138: Module 128 – with rank 100 according to estimated information content.

Rank	Probability	OG	Description
1*	0.089	COG0642 [T]	Signal transduction histidine kinase
2*	0.073	COG0784 [T]	FOG: CheY-like receiver
3*	0.036	COG0841 [V]	Cation/multidrug efflux pump
4*	0.025	COG0596 [R]	Predicted hydrolases or acyltransferases (alpha/beta hydrolase superfamily)
5*	0.017	COG4251 [T]	Bacteriophytochrome (light-regulated signal transduction histidine kinase)
6	0.013	COG1459 [N,U]	Type II secretory pathway, component PulF
7	0.013	COG1741 [R]	Pirin-related protein
8	0.012	COG2885 [M]	Outer membrane protein and related peptidoglycan-associated (lipo)proteins
9	0.012	COG1305 [E]	Transglutaminase-like enzymes, putative cysteine proteases
10*	0.011	COG1352 [N,T]	Methylase of chemotaxis methyl-accepting proteins
11	0.011	COG0564 [J]	Pseudouridylate synthases, 23S RNA-specific
12	0.010	COG0402 [F,R]	Cytosine deaminase and related metal-dependent hydrolases

Table S 139: Module 97 – with rank 101 according to estimated information content.

Rank	Probability	OG	Description
1*	0.057	COG4577 [Q,C]	Carbon dioxide concentrating mechanism/carboxysome shell protein
2*	0.033	COG0664 [T]	cAMP-binding proteins - catabolite gene activator and regulatory subunit of cAMP-dependent protein kinases
3	0.033	COG0789 [K]	Predicted transcriptional regulators
4*	0.019	COG0346 [E]	Lactoylglutathione lyase and related lyases
5*	0.019	COG4576 [Q,C]	Carbon dioxide concentrating mechanism/carboxysome shell protein
6*	0.018	COG1454 [C]	Alcohol dehydrogenase, class IV
7	0.018	COG0406 [G]	Fructose-2,6-bisphosphatase
8*	0.018	COG4886 [S]	Leucine-rich repeat (LRR) protein
9*	0.018	COG3716 [G]	Phosphotransferase system, mannose/fructose/N-acetylgalactosamine-specific component IID
10*	0.017	COG4869 [Q]	Propanediol utilization protein
11	0.017	COG1440 [G]	Phosphotransferase system cellobiose-specific component IIB
12*	0.017	COG0176 [G]	Transaldolase
13	0.016	COG0534 [V]	Na+-driven multidrug efflux pump
14*	0.015	COG3444 [G]	Phosphotransferase system, mannose/fructose/N-acetylgalactosamine-specific component IIB
15*	0.015	COG1882 [C]	Pyruvate-formate lyase
16*	0.015	COG3715 [G]	Phosphotransferase system, mannose/fructose/N-acetylgalactosamine-specific component IIC
17*	0.014	COG2188 [K]	Transcriptional regulators
18	0.014	COG1705 [N,U]	Muramidase (flagellum-specific)
19*	0.013	COG4917 [E]	Ethanolamine utilization protein
20*	0.013	COG0235 [G]	Ribulose-5-phosphate 4-epimerase and related epimerases and aldolases
21	0.013	COG3103 [T]	SH3 domain protein
22*	0.013	COG4816 [E]	Ethanolamine utilization protein
23*	0.012	COG2893 [G]	Phosphotransferase system, mannose/fructose-specific component IIA
24*	0.011	COG4810 [E]	Ethanolamine utilization protein
25*	0.011	COG1221 [K,T]	Transcriptional regulators containing an AAA-type ATPase domain and a DNA-binding domain
26	0.011	COG1802 [K]	Transcriptional regulators

Table S 140: Module 149 – with rank 104 according to estimated information content.

Rank	Probability	OG	Description
1*	0.148	COG0477 [G,E,P,R]	Permeases of the major facilitator superfamily
2*	0.025	COG1960 [I]	Acyl-CoA dehydrogenases
3*	0.023	COG0531 [E]	Amino acid transporters
4*	0.019	COG2141 [C]	Coenzyme F420-dependent N5,N10-methylene tetrahydromethanopterin reductase and related flavin-dependent oxidoreductases
5*	0.018	COG0589 [T]	Universal stress protein UspA and related nucleotide-binding proteins
6	0.016	COG2200 [T]	FOG: EAL domain
7	0.016	COG1113 [E]	Gamma-aminobutyrate permease and related permeases
8*	0.016	COG1280 [E]	Putative threonine efflux protein
9*	0.016	COG0697 [G,E,R]	Permeases of the drug/metabolite transporter (DMT) superfamily
10	0.012	COG4253 [S]	Uncharacterized protein conserved in bacteria
11	0.012	COG0028 [E,H]	Thiamine pyrophosphate-requiring enzymes [acetolactate synthase, pyruvate dehydrogenase (cytochrome), glyoxylate carboligase, phosphonopyruvate decarboxylase]
12*	0.012	COG0500 [Q,R]	SAM-dependent methyltransferases
13*	0.012	COG0494 [L,R]	NTP pyrophosphohydrolases including oxidative damage repair enzymes
14*	0.011	COG1835 [I]	Predicted acyltransferases
15	0.011	COG2050 [Q]	Uncharacterized protein, possibly involved in aromatic compounds catabolism
16*	0.011	COG3511 [M]	Phospholipase C

Table S 141: Module 169 – with rank 105 according to estimated information content.

Rank	Probability	OG	Description
1*	0.128	COG0477 [G,E,P,R]	Permeases of the major facilitator superfamily
2*	0.048	COG0531 [E]	Amino acid transporters
3	0.033	COG3104 [E]	Dipeptide/tripeptide permease
4*	0.027	COG1028 [I,Q,R]	Dehydrogenases with different specificities (related to short-chain alcohol dehydrogenases)
5*	0.017	COG0156 [H]	7-keto-8-aminopelargonate synthetase and related enzymes
6*	0.014	COG1566 [V]	Multidrug resistance efflux pump
7*	0.014	COG0814 [E]	Amino acid permeases
8*	0.014	COG0277 [C]	FAD/FMN-containing dehydrogenases
9*	0.012	COG0625 [O]	Glutathione S-transferase
10*	0.011	COG1278 [K]	Cold shock proteins
11*	0.010	COG0697 [G,E,R]	Permeases of the drug/metabolite transporter (DMT) superfamily

Table S 142: Module 162 – with rank 106 according to estimated information content.

Rank	Probability	OG	Description
1*	0.040	COG1132 [V]	ABC-type multidrug transport system, ATPase and permease components
2*	0.027	COG0454 [K,R]	Histone acetyltransferase HPA2 and related acetyltransferases
3	0.027	COG2972 [T]	Predicted signal transduction protein with a C-terminal ATPase domain
4*	0.025	COG1846 [K]	Transcriptional regulators
5	0.021	COG1653 [G]	ABC-type sugar transport system, periplasmic component
6*	0.020	COG0183 [I]	Acetyl-CoA acetyltransferase
7	0.019	COG0038 [P]	Chloride channel protein EriC
8*	0.019	COG1028 [I,Q,R]	Dehydrogenases with different specificities (related to short-chain alcohol dehydrogenases)
9*	0.018	COG0494 [L,R]	NTP pyrophosphohydrolases including oxidative damage repair enzymes
10*	0.017	COG0511 [I]	Biotin carboxyl carrier protein
11	0.016	COG0531 [E]	Amino acid transporters
12	0.016	COG0609 [P]	ABC-type Fe3+-siderophore transport system, permease component
13*	0.016	COG0726 [G]	Predicted xylanase/chitin deacetylase
14	0.013	COG1266 [R]	Predicted metal-dependent membrane protease
15*	0.013	COG5016 [C]	Pyruvate/oxaloacetate carboxyltransferase
16*	0.013	COG1883 [C]	Na+-transporting methylmalonyl-CoA/oxaloacetate decarboxylase, beta subunit
17*	0.012	COG0251 [J]	Putative translation initiation inhibitor, yjgF family
18*	0.012	COG2301 [G]	Citrate lyase beta subunit
19*	0.012	COG0657 [I]	Esterase/lipase
20*	0.012	COG1670 [J]	Acetyltransferases, including N-acetylases of ribosomal proteins
21	0.011	COG0614 [P]	ABC-type Fe3+-hydroxamate transport system, periplasmic component
22	0.011	COG1120 [P,H]	ABC-type cobalamin/Fe3+-siderophores transport systems, ATPase components
23	0.011	COG3158 [P]	K+ transporter
24*	0.011	COG1788 [I]	Acyl CoA:acetate/3-ketoacid CoA transferase, alpha subunit
25	0.010	COG1523 [G]	Type II secretory pathway, pullulanase PulA and related glycosidases

Table S 143: Module 167 – with rank 107 according to estimated information content.

Rank	Probability	OG	Description
1*	0.032	COG0765 [E]	ABC-type amino acid transport system, permease component
2*	0.029	COG0834 [E,T]	ABC-type amino acid transport/signal transduction systems, periplasmic component/domain
3*	0.027	COG0561 [R]	Predicted hydrolases of the HAD superfamily
4*	0.020	COG2188 [K]	Transcriptional regulators
5*	0.020	COG1126 [E]	ABC-type polar amino acid transport system, ATPase component
6*	0.018	COG2723 [G]	Beta-glucosidase/6-phospho-beta-glucosidase/beta-galactosidase
7*	0.017	COG3444 [G]	Phosphotransferase system, mannose/fructose/N-acetylgalactosamine-specific component IIB
8*	0.017	COG3715 [G]	Phosphotransferase system, mannose/fructose/N-acetylgalactosamine-specific component IIC
9*	0.017	COG3716 [G]	Phosphotransferase system, mannose/fructose/N-acetylgalactosamine-specific component IID
10*	0.016	COG1940 [K,G]	Transcriptional regulator/sugar kinase
11*	0.016	COG2893 [G]	Phosphotransferase system, mannose/fructose-specific component IIA
12*	0.016	COG3279 [K,T]	Response regulator of the LytR/AlgR family
13*	0.015	COG1609 [K]	Transcriptional regulators
14*	0.014	COG0789 [K]	Predicted transcriptional regulators
15*	0.013	COG1455 [G]	Phosphotransferase system cellobiose-specific component IIC
16*	0.013	COG0366 [G]	Glycosidases
17*	0.013	COG2190 [G]	Phosphotransferase system IIA components
18	0.012	COG2244 [R]	Membrane protein involved in the export of O-antigen and teichoic acid
19*	0.012	COG0542 [O]	ATPases with chaperone activity, ATP-binding subunit
20*	0.012	COG0474 [P]	Cation transport ATPase
21	0.012	COG1577 [I]	Mevalonate kinase
22*	0.011	COG2217 [P]	Cation transport ATPase
23	0.011	COG1104 [E]	Cysteine sulfinate desulfinase/cysteine desulfurase and related enzymes
24	0.011	COG4720 [S]	Predicted membrane protein
25	0.010	COG0580 [G]	Glycerol uptake facilitator and related permeases (Major Intrinsic Protein Family)
26	0.010	COG0431 [R]	Predicted flavoprotein

Table S 144: Module 60 – with rank 108 according to estimated information content.

Rank	Probability	OG	Description
1*	0.032	COG0625 [O]	Glutathione S-transferase
2	0.024	COG0494 [L,R]	NTP pyrophosphohydrolases including oxidative damage repair enzymes
3*	0.018	COG0764 [I]	3-hydroxymyristoyl/3-hydroxydecanoyl-(acyl carrier protein) dehydratases
4	0.017	COG1309 [K]	Transcriptional regulator
5	0.017	COG1643 [L]	HrpA-like helicases
6*	0.016	COG0665 [E]	Glycine/D-amino acid oxidases (deaminating)
7*	0.015	COG1092 [R]	Predicted SAM-dependent methyltransferases
8*	0.014	COG0488 [R]	ATPase components of ABC transporters with duplicated ATPase domains
9	0.014	COG0534 [V]	Na+-driven multidrug efflux pump
10*	0.013	COG2813 [J]	16S RNA G1207 methylase RsmC
11	0.013	COG0628 [R]	Predicted permease
12*	0.013	COG0824 [R]	Predicted thioesterase
13*	0.013	COG1011 [R]	Predicted hydrolase (HAD superfamily)
14*	0.012	COG0456 [R]	Acetyltransferases
15	0.011	COG1826 [U]	Sec-independent protein secretion pathway components
16	0.011	COG2194 [R]	Predicted membrane-associated, metal-dependent hydrolase
17*	0.010	COG0189 [H,J]	Glutathione synthase/Ribosomal protein S6 modification enzyme (glutaminyl transferase)
18	0.010	COG0025 [P]	NhaP-type Na+/H+ and K+/H+ antiporters

Table S 145: Module 99 – with rank 109 according to estimated information content.

Rank	Probability	OG	Description
1*	0.132	COG0583 [K]	Transcriptional regulator
2*	0.041	COG0745 [T,K]	Response regulators consisting of a CheY-like receiver domain and a winged-helix DNA-binding domain
3*	0.037	COG1538 [M,U]	Outer membrane protein
4*	0.030	COG3203 [M]	Outer membrane protein (porin)
5*	0.022	COG0477 [G,E,P,R]	Permeases of the major facilitator superfamily
6	0.020	COG1167 [K,E]	Transcriptional regulators containing a DNA-binding HTH domain and an aminotransferase domain (MocR family) and their eukaryotic orthologs
7	0.020	COG0665 [E]	Glycine/D-amino acid oxidases (deaminating)
8*	0.016	COG1846 [K]	Transcriptional regulators
9*	0.014	COG0841 [V]	Cation/multidrug efflux pump
10*	0.014	COG0526 [O,C]	Thiol-disulfide isomerase and thioredoxins
11	0.014	COG4638 [P,R]	Phenylpropionate dioxygenase and related ring-hydroxylating dioxygenases, large terminal subunit
12	0.011	COG2059 [P]	Chromate transport protein ChrA
13*	0.010	COG0625 [O]	Glutathione S-transferase

Table S 146: Module 113 – with rank 110 according to estimated information content.

Rank	Probability	OG	Description
1*	0.069	COG1960 [I]	Acyl-CoA dehydrogenases
2*	0.060	COG1028 [I,Q,R]	Dehydrogenases with different specificities (related to short-chain alcohol dehydrogenases)
3*	0.054	COG1309 [K]	Transcriptional regulator
4	0.040	COG1463 [Q]	ABC-type transport system involved in resistance to organic solvents, periplasmic component
5*	0.038	COG1024 [I]	Enoyl-CoA hydratase/carnithine racemase
6*	0.036	COG2124 [Q]	Cytochrome P450
7*	0.031	COG0318 [I,Q]	Acyl-CoA synthetases (AMP-forming)/AMP-acid ligases II
8*	0.027	COG2141 [C]	Coenzyme F420-dependent N5,N10-methylene tetrahydromethanopterin reductase and related flavin-dependent oxidoreductases
9*	0.025	COG0183 [I]	Acetyl-CoA acetyltransferase
10*	0.014	COG1804 [C]	Predicted acyl-CoA transferases/carnitine dehydratase
11*	0.013	COG0596 [R]	Predicted hydrolases or acyltransferases (alpha/beta hydrolase superfamily)
12	0.013	COG0767 [Q]	ABC-type transport system involved in resistance to organic solvents, permease component
13	0.012	COG2409 [R]	Predicted drug exporters of the RND superfamily
14*	0.012	COG3315 [Q]	O-Methyltransferase involved in polyketide biosynthesis

Table S 147: Module 90 – with rank 112 according to estimated information content.

Rank	Probability	OG	Description
1	0.029	COG1479 [S]	Uncharacterized conserved protein
2	0.028	COG0790 [R]	FOG: TPR repeat, SEL1 subfamily
3*	0.025	COG2189 [L]	Adenine specific DNA methylase Mod
4	0.022	COG1464 [P]	ABC-type metal ion transport system, periplasmic component/surface antigen
5	0.021	COG3306 [M]	Glycosyltransferase involved in LPS biosynthesis
6*	0.020	COG0270 [L]	Site-specific DNA methylase
7	0.020	COG0247 [C]	Fe-S oxidoreductase
8	0.019	COG1629 [P]	Outer membrane receptor proteins, mostly Fe transport
9	0.017	COG0277 [C]	FAD/FMN-containing dehydrogenases
10*	0.017	COG0863 [L]	DNA modification methylase
11*	0.017	COG2249 [R]	Putative NADPH-quinone reductase (modulator of drug activity B)
12	0.016	COG0730 [R]	Predicted permeases
13	0.016	COG1442 [M]	Lipopolysaccharide biosynthesis proteins, LPS:glycosyltransferases
14	0.014	COG2194 [R]	Predicted membrane-associated, metal-dependent hydrolase
15	0.014	COG0471 [P]	Di- and tricarboxylate transporters
16	0.014	COG1135 [P]	ABC-type metal ion transport system, ATPase component
17	0.013	COG2011 [P]	ABC-type metal ion transport system, permease component
18	0.013	COG0551 [L]	Zn-finger domain associated with topoisomerase type I
19	0.013	COG1966 [T]	Carbon starvation protein, predicted membrane protein
20	0.013	COG1139 [C]	Uncharacterized conserved protein containing a ferredoxin-like domain
21	0.013	COG1027 [E]	Aspartate ammonia-lyase
22	0.012	COG1115 [E]	Na+/alanine symporter
23	0.012	COG1432 [S]	Uncharacterized conserved protein
24	0.012	COG2056 [R]	Predicted permease
25	0.011	COG0494 [L,R]	NTP pyrophosphohydrolases including oxidative damage repair enzymes
26*	0.011	COG0753 [P]	Catalase
27*	0.011	COG4221 [R]	Short-chain alcohol dehydrogenase of unknown specificity
28	0.011	COG0826 [O]	Collagenase and related proteases
29*	0.011	COG0778 [C]	Nitroreductase
30	0.011	COG2252 [R]	Permeases
31	0.011	COG1757 [C]	Na+/H+ antiporter
32	0.010	COG1556 [S]	Uncharacterized conserved protein

Table S 148: Module 95 – with rank 113 according to estimated information content.

Rank	Probability	OG	Description
1*	0.032	COG1012 [C]	NAD-dependent aldehyde dehydrogenases
2*	0.018	COG0243 [C]	Anaerobic dehydrogenases, typically selenocysteine-containing
3*	0.018	COG0454 [K,R]	Histone acetyltransferase HPA2 and related acetyltransferases
4*	0.017	COG0277 [C]	FAD/FMN-containing dehydrogenases
5*	0.017	COG0296 [G]	1,4-alpha-glucan branching enzyme
6*	0.015	COG0644 [C]	Dehydrogenases (flavoproteins)
7*	0.015	COG2084 [I]	3-hydroxyisobutyrate dehydrogenase and related beta-hydroxyacid dehydrogenases
8*	0.014	COG1064 [R]	Zn-dependent alcohol dehydrogenases
9*	0.014	COG0303 [H]	Molybdopterin biosynthesis enzyme
10*	0.012	COG0175 [E,H]	3-phosphoadenosine 5-phosphosulfate sulfotransferase (PAPS reductase)/FAD synthetase and related enzymes
11	0.012	COG0599 [S]	Uncharacterized homolog of gamma-carboxymuconolactone decarboxylase subunit
12	0.012	COG2057 [I]	Acyl CoA:acetate/3-ketoacid CoA transferase, beta subunit
13	0.011	COG0079 [E]	Histidinol-phosphate/aromatic aminotransferase and cobyric acid decarboxylase
14*	0.011	COG0584 [C]	Glycerophosphoryl diester phosphodiesterase
15*	0.011	COG0122 [L]	3-methyladenine DNA glycosylase/8-oxoguanine DNA glycosylase
16*	0.011	COG2224 [C]	Isocitrate lyase
17*	0.010	COG0155 [P]	Sulfite reductase, beta subunit (hemoprotein)
18	0.010	COG1004 [M]	Predicted UDP-glucose 6-dehydrogenase
19*	0.010	COG0664 [T]	cAMP-binding proteins - catabolite gene activator and regulatory subunit of cAMP-dependent protein kinases
20*	0.010	COG0524 [G]	Sugar kinases, ribokinase family
21*	0.010	COG0604 [C,R]	NADPH:quinone reductase and related Zn-dependent oxidoreductases

Table S 149: Module 104 – with rank 114 according to estimated information content.

Rank	Probability	OG	Description
1*	0.025	COG0845 [M]	Membrane-fusion protein
2*	0.024	COG0811 [U]	Biopolymer transport proteins
3*	0.021	COG2885 [M]	Outer membrane protein and related peptidoglycan-associated (lipo)proteins
4*	0.018	COG1538 [M,U]	Outer membrane protein
5	0.016	COG0612 [R]	Predicted Zn-dependent peptidases
6	0.016	COG0760 [O]	Parvulin-like peptidyl-prolyl isomerase
7	0.016	COG0795 [R]	Predicted permeases
8*	0.016	COG0848 [U]	Biopolymer transport protein
9	0.015	COG0739 [M]	Membrane proteins related to metalloendopeptidases
10	0.015	COG0388 [R]	Predicted amidohydrolase
11	0.015	COG0488 [R]	ATPase components of ABC transporters with duplicated ATPase domains
12*	0.014	COG0735 [P]	Fe2+/Zn2+ uptake regulation proteins
13	0.013	COG0621 [J]	2-methylthioadenine synthetase
14*	0.011	COG0841 [V]	Cation/multidrug efflux pump
15	0.011	COG0501 [O]	Zn-dependent protease with chaperone function
16	0.011	COG0741 [M]	Soluble lytic murein transglycosylase and related regulatory proteins (some contain LysM/invasin domains)

Table S 150: Module 34 – with rank 115 according to estimated information content.

Rank	Probability	OG	Description
1*	0.038	COG1846 [K]	Transcriptional regulators
2*	0.031	COG0454 [K,R]	Histone acetyltransferase HPA2 and related acetyltransferases
3*	0.025	COG1131 [V]	ABC-type multidrug transport system, ATPase component
4*	0.023	COG0531 [E]	Amino acid transporters
5	0.022	COG1476 [K]	Predicted transcriptional regulators
6	0.022	COG0789 [K]	Predicted transcriptional regulators
7*	0.021	COG0778 [C]	Nitroreductase
8*	0.020	COG0491 [R]	Zn-dependent hydrolases, including glyoxylases
9	0.020	COG1695 [K]	Predicted transcriptional regulators
10*	0.018	COG0488 [R]	ATPase components of ABC transporters with duplicated ATPase domains
11	0.017	COG1115 [E]	Na+/alanine symporter
12*	0.015	COG1167 [K,E]	Transcriptional regulators containing a DNA-binding HTH domain and an aminotransferase domain (MocR family) and their eukaryotic orthologs
13*	0.015	COG0457 [R]	FOG: TPR repeat
14	0.013	COG0614 [P]	ABC-type Fe3+-hydroxamate transport system, periplasmic component
15	0.013	COG1301 [C]	Na+/H+-dicarboxylate symporters
16	0.013	COG1114 [E]	Branched-chain amino acid permeases
17*	0.012	COG0840 [N,T]	Methyl-accepting chemotaxis protein
18*	0.011	COG1725 [K]	Predicted transcriptional regulators
19	0.011	COG3708 [S]	Uncharacterized protein conserved in bacteria
20	0.011	COG0768 [M]	Cell division protein FtsI/penicillin-binding protein 2
21	0.011	COG0671 [I]	Membrane-associated phospholipid phosphatase
22*	0.011	COG1733 [K]	Predicted transcriptional regulators
23	0.011	COG3227 [E]	Zinc metalloprotease (elastase)
24*	0.010	COG0526 [O,C]	Thiol-disulfide isomerase and thioredoxins
25*	0.010	COG1277 [R]	ABC-type transport system involved in multi-copper enzyme maturation, permease component

Table S 151: Module 52 – with rank 116 according to estimated information content.

Rank	Probability	OG	Description
1*	0.039	COG1131 [V]	ABC-type multidrug transport system, ATPase component
2*	0.035	COG0477 [G,E,P,R]	Permeases of the major facilitator superfamily
3*	0.025	COG0438 [M]	Glycosyltransferase
4*	0.022	COG1014 [C]	Pyruvate:ferredoxin oxidoreductase and related 2-oxoacid:ferredoxin oxidoreductases, gamma subunit
5	0.021	COG0312 [R]	Predicted Zn-dependent proteases and their inactivated homologs
6*	0.021	COG1032 [C]	Fe-S oxidoreductase
7*	0.021	COG0842 [V]	ABC-type multidrug transport system, permease component
8*	0.017	COG0543 [H,C]	2-polyprenylphenol hydroxylase and related flavodoxin oxidoreductases
9*	0.016	COG0674 [C]	Pyruvate:ferredoxin oxidoreductase and related 2-oxoacid:ferredoxin oxidoreductases, alpha subunit
10*	0.014	COG0388 [R]	Predicted amidohydrolase
11	0.013	COG0432 [S]	Uncharacterized conserved protein
12	0.013	COG0651 [C,P]	Formate hydrogenlyase subunit 3/Multisubunit Na+/H+ antiporter, MnhD subunit
13*	0.013	COG0123 [B,Q]	Deacetylases, including yeast histone deacetylase and acetoin utilization protein
14*	0.013	COG0639 [T]	Diadenosine tetraphosphatase and related serine/threonine protein phosphatases
15*	0.012	COG0778 [C]	Nitroreductase
16*	0.012	COG1013 [C]	Pyruvate:ferredoxin oxidoreductase and related 2-oxoacid:ferredoxin oxidoreductases, beta subunit
17*	0.012	COG0493 [E,R]	NADPH-dependent glutamate synthase beta chain and related oxidoreductases
18	0.010	COG1633 [S]	Uncharacterized conserved protein

Table S 152: Module 73 – with rank 117 according to estimated information content.

Rank	Probability	OG	Description
1*	0.050	COG0454 [K,R]	Histone acetyltransferase HPA2 and related acetyltransferases
2*	0.019	COG0463 [M]	Glycosyltransferases involved in cell wall biogenesis
3*	0.017	COG0614 [P]	ABC-type Fe3+-hydroxamate transport system, periplasmic component
4*	0.015	COG2141 [C]	Coenzyme F420-dependent N5,N10-methylene tetrahydromethanopterin reductase and related flavin-dependent oxidoreductases
5*	0.014	COG0491 [R]	Zn-dependent hydrolases, including glyoxylases
6*	0.014	COG0702 [M,G]	Predicted nucleoside-diphosphate-sugar epimerases
7*	0.013	COG0607 [P]	Rhodanese-related sulfurtransferase
8*	0.013	COG0494 [L,R]	NTP pyrophosphohydrolases including oxidative damage repair enzymes
9*	0.012	COG0824 [R]	Predicted thioesterase
10	0.012	COG0737 [F]	5-nucleotidase/2,3-cyclic phosphodiesterase and related esterases
11*	0.012	COG0492 [O]	Thioredoxin reductase
12	0.011	COG0010 [E]	Arginase/agmatinase/formimionoglutamate hydrolase, arginase family
13*	0.011	COG0365 [I]	Acyl-coenzyme A synthetases/AMP-(fatty) acid ligases
14	0.010	COG1278 [K]	Cold shock proteins
15	0.010	COG0001 [H]	Glutamate-1-semialdehyde aminotransferase
16	0.010	COG1055 [P]	Na+/H+ antiporter NhaD and related arsenite permeases
17	0.010	COG1363 [G]	Cellulase M and related proteins
18	0.010	COG0394 [T]	Protein-tyrosine-phosphatase

Table S 153: Module 197 – with rank 118 according to estimated information content.

Rank	Probability	OG	Description
1*	0.073	COG2199 [T]	FOG: GGDEF domain
2*	0.042	COG2200 [T]	FOG: EAL domain
3*	0.038	COG2202 [T]	FOG: PAS/PAC domain
4	0.020	COG0526 [O,C]	Thiol-disulfide isomerase and thioredoxins
5*	0.020	COG2204 [T]	Response regulator containing CheY-like receiver, AAA-type ATPase, and DNA-binding domains
6*	0.018	COG2165 [N,U]	Type II secretory pathway, pseudopilin PulG
7*	0.017	COG2804 [N,U]	Type II secretory pathway, ATPase PulE/Tfp pilus assembly pathway, ATPase PilB
8*	0.016	COG2203 [T]	FOG: GAF domain
9*	0.013	COG1450 [N,U]	Type II secretory pathway, component PulD
10*	0.012	COG1192 [D]	ATPases involved in chromosome partitioning
11	0.012	COG2863 [C]	Cytochrome c553
12	0.012	COG0739 [M]	Membrane proteins related to metalloendopeptidases
13	0.010	COG0546 [R]	Predicted phosphatases
14*	0.010	COG1595 [K]	DNA-directed RNA polymerase specialized sigma subunit, sigma24 homolog
15*	0.010	COG0714 [R]	MoxR-like ATPases

Table S 154: Module 110 – with rank 119 according to estimated information content.

Rank	Probability	OG	Description
1*	0.077	COG0642 [T]	Signal transduction histidine kinase
2*	0.066	COG0745 [T,K]	Response regulators consisting of a CheY-like receiver domain and a winged-helix DNA-binding domain
3*	0.033	COG2207 [K]	AraC-type DNA-binding domain-containing proteins
4*	0.026	COG1396 [K]	Predicted transcriptional regulators
5*	0.025	COG1136 [V]	ABC-type antimicrobial peptide transport system, ATPase component
6*	0.024	COG0577 [V]	ABC-type antimicrobial peptide transport system, permease component
7*	0.024	COG2247 [M]	Putative cell wall-binding domain
8	0.024	COG1145 [C]	Ferredoxin
9	0.016	COG0655 [R]	Multimeric flavodoxin WrbA
10*	0.013	COG0474 [P]	Cation transport ATPase
11*	0.012	COG5492 [N]	Bacterial surface proteins containing Ig-like domains
12*	0.011	COG0210 [L]	Superfamily I DNA and RNA helicases
13*	0.011	COG5263 [R]	FOG: Glucan-binding domain (YG repeat)
14	0.011	COG0493 [E,R]	NADPH-dependent glutamate synthase beta chain and related oxidoreductases
15	0.010	COG0609 [P]	ABC-type Fe3+-siderophore transport system, permease component

Table S 155: Module 51 – with rank 121 according to estimated information content.

Rank	Probability	OG	Description
1	0.038	COG0715 [P]	ABC-type nitrate/sulfonate/bicarbonate transport systems, periplasmic components
2*	0.034	COG0607 [P]	Rhodanese-related sulfurtransferase
3	0.034	COG0845 [M]	Membrane-fusion protein
4*	0.033	COG0714 [R]	MoxR-like ATPases
5	0.028	COG0730 [R]	Predicted permeases
6*	0.025	COG2010 [C]	Cytochrome c, mono- and diheme variants
7*	0.023	COG2041 [R]	Sulfite oxidase and related enzymes
8*	0.022	COG0346 [E]	Lactoylglutathione lyase and related lyases
9*	0.017	COG4993 [G]	Glucose dehydrogenase
10	0.017	COG3284 [Q,K]	Transcriptional activator of acetoin/glycerol metabolism
11	0.016	COG0494 [L,R]	NTP pyrophosphohydrolases including oxidative damage repair enzymes
12	0.015	COG0600 [P]	ABC-type nitrate/sulfonate/bicarbonate transport system, permease component
13	0.015	COG5501 [S]	Predicted secreted protein
14	0.014	COG2391 [R]	Predicted transporter component
15	0.013	COG1116 [P]	ABC-type nitrate/sulfonate/bicarbonate transport system, ATPase component
16	0.013	COG0464 [O]	ATPases of the AAA+ class
17*	0.013	COG1894 [C]	NADH:ubiquinone oxidoreductase, NADH-binding (51 kD) subunit
18	0.012	COG0737 [F]	5-nucleotidase/2,3-cyclic phosphodiesterase and related esterases
19*	0.012	COG1052 [C,H,R]	Lactate dehydrogenase and related dehydrogenases
20	0.011	COG3391 [S]	Uncharacterized conserved protein

Table S 156: Module 86 – with rank 122 according to estimated information content.

Rank	Probability	OG	Description
1*	0.045	COG0451 [M,G]	Nucleoside-diphosphate-sugar epimerases
2*	0.040	COG1028 [I,Q,R]	Dehydrogenases with different specificities (related to short-chain alcohol dehydrogenases)
3*	0.030	COG0745 [T,K]	Response regulators consisting of a CheY-like receiver domain and a winged-helix DNA-binding domain
4*	0.027	COG0568 [K]	DNA-directed RNA polymerase, sigma subunit (sigma70/sigma32)
5*	0.020	COG0457 [R]	FOG: TPR repeat
6*	0.019	COG0652 [O]	Peptidyl-prolyl cis-trans isomerase (rotamase) - cyclophilin family
7*	0.019	COG0438 [M]	Glycosyltransferase
8*	0.019	COG2214 [O]	DnaJ-class molecular chaperone
9*	0.015	COG0845 [M]	Membrane-fusion protein
10*	0.014	COG1132 [V]	ABC-type multidrug transport system, ATPase and permease components
11*	0.013	COG0463 [M]	Glycosyltransferases involved in cell wall biogenesis
12	0.013	COG0659 [P]	Sulfate permease and related transporters (MFS superfamily)
13*	0.012	COG0626 [E]	Cystathionine beta-lyases/cystathionine gamma-synthases
14*	0.012	COG0665 [E]	Glycine/D-amino acid oxidases (deaminating)
15*	0.011	COG0465 [O]	ATP-dependent Zn proteases
16	0.011	COG0711 [C]	F0F1-type ATP synthase, subunit b
17*	0.011	COG0075 [E]	Serine-pyruvate aminotransferase/archaeal aspartate aminotransferase
18	0.011	COG0793 [M]	Periplasmic protease
19*	0.010	COG0673 [R]	Predicted dehydrogenases and related proteins

Table S 157: Module 66 – with rank 123 according to estimated information content.

Rank	Probability	OG	Description
1*	0.014	COG0115 [E,H]	Branched-chain amino acid aminotransferase/4-amino-4-deoxychorismate lyase
2	0.013	COG0772 [D]	Bacterial cell division membrane protein
3*	0.012	COG0031 [E]	Cysteine synthase
4	0.011	COG0768 [M]	Cell division protein FtsI/penicillin-binding protein 2
5*	0.011	COG0516 [F]	IMP dehydrogenase/GMP reductase
6	0.010	COG0472 [M]	UDP-N-acetylmuramyl pentapeptide phosphotransferase/UDP-N-acetylglucosamine-1-phosphate transferase

Table S 158: Module 132 – with rank 126 according to estimated information content.

Rank	Probability	OG	Description
1*	0.050	COG0642 [T]	Signal transduction histidine kinase
2*	0.036	COG1670 [J]	Acetyltransferases, including N-acetylases of ribosomal proteins
3*	0.034	COG0596 [R]	Predicted hydrolases or acyltransferases (alpha/beta hydrolase superfamily)
4*	0.033	COG0745 [T,K]	Response regulators consisting of a CheY-like receiver domain and a winged-helix DNA-binding domain
5*	0.029	COG0494 [L,R]	NTP pyrophosphohydrolases including oxidative damage repair enzymes
6*	0.028	COG0500 [Q,R]	SAM-dependent methyltransferases
7*	0.028	COG0697 [G,E,R]	Permeases of the drug/metabolite transporter (DMT) superfamily
8*	0.025	COG0577 [V]	ABC-type antimicrobial peptide transport system, permease component
9*	0.023	COG1680 [V]	Beta-lactamase class C and other penicillin binding proteins
10*	0.021	COG1136 [V]	ABC-type antimicrobial peptide transport system, ATPase component
11	0.018	COG4166 [E]	ABC-type oligopeptide transport system, periplasmic component
12	0.016	COG1266 [R]	Predicted metal-dependent membrane protease
13*	0.014	COG1132 [V]	ABC-type multidrug transport system, ATPase and permease components
14*	0.014	COG0346 [E]	Lactoylglutathione lyase and related lyases
15*	0.013	COG0604 [C,R]	NADPH:quinone reductase and related Zn-dependent oxidoreductases
16*	0.013	COG1595 [K]	DNA-directed RNA polymerase specialized sigma subunit, sigma24 homolog
17	0.012	COG3103 [T]	SH3 domain protein
18*	0.011	COG0726 [G]	Predicted xylanase/chitin deacetylase
19*	0.010	COG0451 [M,G]	Nucleoside-diphosphate-sugar epimerases
20	0.010	COG0546 [R]	Predicted phosphatases
21	0.010	COG0561 [R]	Predicted hydrolases of the HAD superfamily

Table S 159: Module 42 – with rank 127 according to estimated information content.

Rank	Probability	OG	Description
1*	0.038	COG0451 [M,G]	Nucleoside-diphosphate-sugar epimerases
2*	0.030	COG1595 [K]	DNA-directed RNA polymerase specialized sigma subunit, sigma24 homolog
3*	0.026	COG0784 [T]	FOG: CheY-like receiver
4*	0.025	COG0664 [T]	cAMP-binding proteins - catabolite gene activator and regulatory subunit of cAMP-dependent protein kinases
5*	0.018	COG0845 [M]	Membrane-fusion protein
6*	0.017	COG2197 [T,K]	Response regulator containing a CheY-like receiver domain and an HTH DNA-binding domain
7	0.014	COG2128 [S]	Uncharacterized conserved protein
8*	0.014	COG0843 [C]	Heme/copper-type cytochrome/quinol oxidases, subunit 1
9	0.014	COG3791 [S]	Uncharacterized conserved protein
10	0.013	COG2259 [S]	Predicted membrane protein
11	0.013	COG0640 [K]	Predicted transcriptional regulators
12*	0.013	COG2771 [K]	DNA-binding HTH domain-containing proteins
13*	0.013	COG3206 [M]	Uncharacterized protein involved in exopolysaccharide biosynthesis
14	0.012	COG3795 [S]	Uncharacterized protein conserved in bacteria
15*	0.012	COG1845 [C]	Heme/copper-type cytochrome/quinol oxidase, subunit 3
16*	0.012	COG4566 [T]	Response regulator
17*	0.012	COG1596 [M]	Periplasmic protein involved in polysaccharide export
18	0.011	COG1278 [K]	Cold shock proteins
19*	0.011	COG2010 [C]	Cytochrome c, mono- and diheme variants
20*	0.011	COG4962 [U]	Flp pilus assembly protein, ATPase CpaF
21*	0.011	COG4965 [U]	Flp pilus assembly protein TadB
22*	0.011	COG2064 [N,U]	Flp pilus assembly protein TadC
23*	0.010	COG4964 [U]	Flp pilus assembly protein, secretin CpaC
24	0.010	COG0431 [R]	Predicted flavoprotein
25	0.010	COG0488 [R]	ATPase components of ABC transporters with duplicated ATPase domains
26*	0.010	COG4963 [U]	Flp pilus assembly protein, ATPase CpaE

Table S 160: Module 160 – with rank 128 according to estimated information content.

Rank	Probability	OG	Description
1	0.051	COG0534 [V]	Na+-driven multidrug efflux pump
2	0.025	COG0697 [G,E,R]	Permeases of the drug/metabolite transporter (DMT) superfamily
3*	0.024	COG0438 [M]	Glycosyltransferase
4*	0.019	COG1180 [O]	Pyruvate-formate lyase-activating enzyme
5*	0.016	COG0513 [L,K,J]	Superfamily II DNA and RNA helicases
6*	0.016	COG0674 [C]	Pyruvate:ferredoxin oxidoreductase and related 2-oxoacid:ferredoxin oxidoreductases, alpha subunit
7*	0.016	COG1014 [C]	Pyruvate:ferredoxin oxidoreductase and related 2-oxoacid:ferredoxin oxidoreductases, gamma subunit
8*	0.016	COG1013 [C]	Pyruvate:ferredoxin oxidoreductase and related 2-oxoacid:ferredoxin oxidoreductases, beta subunit
9	0.016	COG1757 [C]	Na+/H+ antiporter
10*	0.015	COG0716 [C]	Flavodoxins
11	0.015	COG0037 [D]	Predicted ATPase of the PP-loop superfamily implicated in cell cycle control
12*	0.014	COG0426 [C]	Uncharacterized flavoproteins
13*	0.013	COG1086 [M,G]	Predicted nucleoside-diphosphate sugar epimerases
14*	0.013	COG0543 [H,C]	2-polyprenylphenol hydroxylase and related flavodoxin oxidoreductases
15*	0.013	COG2233 [F]	Xanthine/uracil permeases
16	0.012	COG0826 [O]	Collagenase and related proteases
17	0.012	COG4656 [C]	Predicted NADH:ubiquinone oxidoreductase, subunit RnfC
18	0.012	COG1284 [S]	Uncharacterized conserved protein
19	0.012	COG0550 [L]	Topoisomerase IA
20	0.012	COG0621 [J]	2-methylthioadenine synthetase
21*	0.011	COG1328 [F]	Oxygen-sensitive ribonucleoside-triphosphate reductase
22*	0.011	COG1853 [R]	Conserved protein/domain typically associated with flavoprotein oxygenases, DIM6/NTAB family
23*	0.011	COG0457 [R]	FOG: TPR repeat
24	0.010	COG0419 [L]	ATPase involved in DNA repair

Table S 161: Module 47 – with rank 129 according to estimated information content.

Rank	Probability	OG	Description
1*	0.059	COG0583 [K]	Transcriptional regulator
2*	0.039	COG0665 [E]	Glycine/D-amino acid oxidases (deaminating)
3	0.025	COG2114 [T]	Adenylate cyclase, family 3 (some proteins contain HAMP domain)
4*	0.025	COG0697 [G,E,R]	Permeases of the drug/metabolite transporter (DMT) superfamily
5	0.023	COG0687 [E]	Spermidine/putrescine-binding periplasmic protein
6*	0.023	COG0625 [O]	Glutathione S-transferase
7	0.022	COG3839 [G]	ABC-type sugar transport systems, ATPase components
8	0.022	COG2931 [Q]	RTX toxins and related Ca2+-binding proteins
9	0.019	COG1177 [E]	ABC-type spermidine/putrescine transport system, permease component II
10*	0.018	COG0404 [E]	Glycine cleavage system T protein (aminomethyltransferase)
11	0.016	COG1176 [E]	ABC-type spermidine/putrescine transport system, permease component I
12	0.016	COG3842 [E]	ABC-type spermidine/putrescine transport systems, ATPase components
13	0.014	COG1123 [R]	ATPase components of various ABC-type transport systems, contain duplicated ATPase
14*	0.013	COG2207 [K]	AraC-type DNA-binding domain-containing proteins
15	0.013	COG3791 [S]	Uncharacterized conserved protein
16*	0.013	COG0491 [R]	Zn-dependent hydrolases, including glyoxylases
17*	0.013	COG1280 [E]	Putative threonine efflux protein
18*	0.012	COG0667 [C]	Predicted oxidoreductases (related to aryl-alcohol dehydrogenases)
19*	0.012	COG1522 [K]	Transcriptional regulators
20	0.011	COG1376 [S]	Uncharacterized protein conserved in bacteria
21*	0.010	COG0174 [E]	Glutamine synthetase
22*	0.010	COG4977 [K]	Transcriptional regulator containing an amidase domain and an AraC-type DNA-binding HTH domain
23*	0.010	COG0446 [R]	Uncharacterized NAD(FAD)-dependent dehydrogenases

Table S 162: Module 67 – with rank 130 according to estimated information content.

Rank	Probability	OG	Description
1	0.047	COG1131 [V]	ABC-type multidrug transport system, ATPase component
2*	0.027	COG0860 [M]	N-acetylmuramoyl-L-alanine amidase
3	0.020	COG2972 [T]	Predicted signal transduction protein with a C-terminal ATPase domain
4*	0.019	COG0791 [M]	Cell wall-associated hydrolases (invasion-associated proteins)
5*	0.019	COG1077 [D]	Actin-like ATPase involved in cell morphogenesis
6	0.018	COG4753 [T]	Response regulator containing CheY-like receiver domain and AraC-type DNA-binding domain
7	0.018	COG2508 [T,Q]	Regulator of polyketide synthase expression
8	0.017	COG1387 [E,R]	Histidinol phosphatase and related hydrolases of the PHP family
9	0.017	COG1959 [K]	Predicted transcriptional regulator
10*	0.017	COG1388 [M]	FOG: LysM repeat
11*	0.017	COG0772 [D]	Bacterial cell division membrane protein
12*	0.015	COG3584 [S]	Uncharacterized protein conserved in bacteria
13	0.014	COG0845 [M]	Membrane-fusion protein
14	0.013	COG0628 [R]	Predicted permease
15	0.011	COG0553 [K,L]	Superfamily II DNA/RNA helicases, SNF2 family
16	0.011	COG1001 [F]	Adenine deaminase
17	0.010	COG1725 [K]	Predicted transcriptional regulators
18	0.010	COG2070 [R]	Dioxygenases related to 2-nitropropane dioxygenase
19	0.010	COG1982 [E]	Arginine/lysine/ornithine decarboxylases
20	0.010	COG0352 [H]	Thiamine monophosphate synthase
21	0.010	COG0681 [U]	Signal peptidase I
22	0.010	COG2244 [R]	Membrane protein involved in the export of O-antigen and teichoic acid

Table S 163: Module 168 – with rank 131 according to estimated information content.

Rank	Probability	OG	Description
1*	0.041	COG1028 [I,Q,R]	Dehydrogenases with different specificities (related to short-chain alcohol dehydrogenases)
2*	0.040	COG0625 [O]	Glutathione S-transferase
3*	0.033	COG0845 [M]	Membrane-fusion protein
4	0.021	COG2010 [C]	Cytochrome c, mono- and diheme variants
5	0.020	COG1187 [J]	16S rRNA uridine-516 pseudouridylate synthase and related pseudouridylate synthases
6	0.013	COG0643 [N,T]	Chemotaxis protein histidine kinase and related kinases
7	0.013	COG1752 [R]	Predicted esterase of the alpha-beta hydrolase superfamily
8	0.013	COG2804 [N,U]	Type II secretory pathway, ATPase PulE/Tfp pilus assembly pathway, ATPase PilB
9*	0.013	COG0824 [R]	Predicted thioesterase
10	0.011	COG1826 [U]	Sec-independent protein secretion pathway components
11	0.011	COG2223 [P]	Nitrate/nitrite transporter
12	0.011	COG1773 [C]	Rubredoxin
13	0.011	COG2334 [R]	Putative homoserine kinase type II (protein kinase fold)
14*	0.010	COG0760 [O]	Parvulin-like peptidyl-prolyl isomerase
15	0.010	COG1765 [O]	Predicted redox protein, regulator of disulfide bond formation

Table S 164: Module 199 – with rank 132 according to estimated information content.

Rank	Probability	OG	Description
1*	0.031	COG3497 [R]	Phage tail sheath protein FI
2	0.031	COG0454 [K,R]	Histone acetyltransferase HPA2 and related acetyltransferases
3*	0.026	COG3500 [R]	Phage protein D
4	0.024	COG0500 [Q,R]	SAM-dependent methyltransferases
5	0.024	COG2197 [T,K]	Response regulator containing a CheY-like receiver domain and an HTH DNA-binding domain
6	0.020	COG1846 [K]	Transcriptional regulators
7*	0.019	COG3948 [R]	Phage-related baseplate assembly protein
8*	0.019	COG5301 [R]	Phage-related tail fibre protein
9*	0.018	COG3628 [R]	Phage baseplate assembly protein W
10*	0.018	COG3498 [R]	Phage tail tube protein FII
11	0.017	COG0338 [L]	Site-specific DNA methylase
12*	0.017	COG3499 [R]	Phage protein U
13*	0.017	COG4385 [R]	Bacteriophage P2-related tail formation protein
14	0.016	COG2932 [K]	Predicted transcriptional regulator
15	0.016	COG3772 [R]	Phage-related lysozyme (muraminidase)
16*	0.016	COG4540 [R]	Phage P2 baseplate assembly protein gpV
17	0.013	COG5518 [R]	Bacteriophage capsid portal protein
18	0.013	COG1167 [K,E]	Transcriptional regulators containing a DNA-binding HTH domain and an aminotransferase domain (MocR family) and their eukaryotic orthologs
19	0.013	NOG04097 [R]	Major capsid protein
20	0.012	NOG05497 [-]	Capsid scaffolding protein
21	0.012	NOG145283 [R]	Terminase
22	0.011	COG2076 [P]	Membrane transporters of cations and cationic drugs
23	0.011	COG0810 [M]	Periplasmic protein TonB, links inner and outer membranes
24	0.011	COG5283 [S]	Phage-related tail protein
25*	0.011	COG5004 [R]	P2-like prophage tail protein X
26	0.011	NOG05927 [L]	Terminase
27	0.010	COG2274 [V]	ABC-type bacteriocin/lantibiotic exporters, contain an N-terminal double-glycine peptidase domain
28	0.010	NOG10946 [L]	Replication protein

Table S 165: Module 7 – with rank 133 according to estimated information content.

Rank	Probability	OG	Description
1	0.019	COG1316 [K]	Transcriptional regulator
2*	0.019	COG0503 [F]	Adenine/guanine phosphoribosyltransferases and related PRPP-binding proteins
3	0.015	COG1418 [R]	Predicted HD superfamily hydrolase
4	0.014	COG1122 [P]	ABC-type cobalt transport system, ATPase component
5*	0.013	COG0561 [R]	Predicted hydrolases of the HAD superfamily
6	0.012	COG0803 [P]	ABC-type metal ion transport system, periplasmic component/surface adhesin
7	0.012	COG0612 [R]	Predicted Zn-dependent peptidases
8	0.011	COG0766 [M]	UDP-N-acetylglucosamine enolpyruvyl transferase
9	0.011	COG0769 [M]	UDP-N-acetylmuramyl tripeptide synthase
10	0.011	COG0472 [M]	UDP-N-acetylmuramyl pentapeptide phosphotransferase/UDP-N-acetylglucosamine-1-phosphate transferase
11	0.011	COG0598 [P]	Mg2+ and Co2+ transporters
12*	0.010	COG0737 [F]	5-nucleotidase/2,3-cyclic phosphodiesterase and related esterases
13	0.010	COG0595 [R]	Predicted hydrolase of the metallo-beta-lactamase superfamily

Table S 166: Module 64 – with rank 134 according to estimated information content.

Rank	Probability	OG	Description
1	0.060	COG0840 [N,T]	Methyl-accepting chemotaxis protein
2	0.034	COG2885 [M]	Outer membrane protein and related peptidoglycan-associated (lipo)proteins
3*	0.034	COG1309 [K]	Transcriptional regulator
4*	0.023	COG1012 [C]	NAD-dependent aldehyde dehydrogenases
5*	0.021	COG0596 [R]	Predicted hydrolases or acyltransferases (alpha/beta hydrolase superfamily)
6	0.018	COG0730 [R]	Predicted permeases
7	0.016	COG0668 [M]	Small-conductance mechanosensitive channel
8	0.014	COG2010 [C]	Cytochrome c, mono- and diheme variants
9	0.013	COG0591 [E,R]	Na+/proline symporter
10	0.013	COG0811 [U]	Biopolymer transport proteins
11*	0.012	COG0531 [E]	Amino acid transporters
12	0.011	COG0247 [C]	Fe-S oxidoreductase
13	0.011	COG3148 [S]	Uncharacterized conserved protein
14	0.010	COG4067 [O]	Uncharacterized protein conserved in archaea

Table S 167: Module 181 – with rank 135 according to estimated information content.

Rank	Probability	OG	Description
1*	0.049	COG0454 [K,R]	Histone acetyltransferase HPA2 and related acetyltransferases
2	0.036	COG1266 [R]	Predicted metal-dependent membrane protease
3*	0.032	COG0745 [T,K]	Response regulators consisting of a CheY-like receiver domain and a winged-helix DNA-binding domain
4*	0.029	COG0463 [M]	Glycosyltransferases involved in cell wall biogenesis
5*	0.026	COG1846 [K]	Transcriptional regulators
6	0.024	COG5263 [R]	FOG: Glucan-binding domain (YG repeat)
7*	0.023	COG0534 [V]	Na+-driven multidrug efflux pump
8	0.022	COG0582 [L]	Integrase
9*	0.018	COG0500 [Q,R]	SAM-dependent methyltransferases
10*	0.016	COG0346 [E]	Lactoylglutathione lyase and related lyases
11*	0.015	COG1670 [J]	Acetyltransferases, including N-acetylases of ribosomal proteins
12	0.015	COG1695 [K]	Predicted transcriptional regulators
13*	0.015	COG1442 [M]	Lipopolysaccharide biosynthesis proteins, LPS:glycosyltransferases
14	0.013	COG0448 [G]	ADP-glucose pyrophosphorylase
15	0.010	COG1404 [O]	Subtilisin-like serine proteases
16	0.010	COG2017 [G]	Galactose mutarotase and related enzymes

Table S 168: Module 91 – with rank 136 according to estimated information content.

Rank	Probability	OG	Description
1*	0.031	COG0454 [K,R]	Histone acetyltransferase HPA2 and related acetyltransferases
2*	0.029	COG0477 [G,E,P,R]	Permeases of the major facilitator superfamily
3*	0.026	COG1396 [K]	Predicted transcriptional regulators
4*	0.025	COG1028 [I,Q,R]	Dehydrogenases with different specificities (related to short-chain alcohol dehydrogenases)
5*	0.017	COG1846 [K]	Transcriptional regulators
6	0.017	COG1455 [G]	Phosphotransferase system cellobiose-specific component IIC
7*	0.016	COG0474 [P]	Cation transport ATPase
8*	0.016	COG0604 [C,R]	NADPH:quinone reductase and related Zn-dependent oxidoreductases
9	0.015	COG1284 [S]	Uncharacterized conserved protein
10*	0.015	COG0586 [S]	Uncharacterized membrane-associated protein
11	0.014	COG1278 [K]	Cold shock proteins
12	0.014	COG1695 [K]	Predicted transcriptional regulators
13*	0.013	COG0531 [E]	Amino acid transporters
14*	0.013	COG2017 [G]	Galactose mutarotase and related enzymes
15*	0.013	COG0446 [R]	Uncharacterized NAD(FAD)-dependent dehydrogenases
16*	0.013	COG1737 [K]	Transcriptional regulators
17	0.012	COG0561 [R]	Predicted hydrolases of the HAD superfamily
18*	0.012	COG0664 [T]	cAMP-binding proteins - catabolite gene activator and regulatory subunit of cAMP-dependent protein kinases
19*	0.012	COG2755 [E]	Lysophospholipase L1 and related esterases
20	0.012	COG4166 [E]	ABC-type oligopeptide transport system, periplasmic component
21	0.011	COG4932 [M]	Predicted outer membrane protein
22	0.011	COG0791 [M]	Cell wall-associated hydrolases (invasion-associated proteins)
23*	0.010	COG0110 [R]	Acetyltransferase (isoleucine patch superfamily)
24	0.010	COG1434 [S]	Uncharacterized conserved protein

Table S 169: Module 158 – with rank 138 according to estimated information content.

Rank	Probability	OG	Description
1*	0.023	COG1145 [C]	Ferredoxin
2	0.021	COG0500 [Q,R]	SAM-dependent methyltransferases
3	0.021	COG1060 [H,R]	Thiamine biosynthesis enzyme ThiH and related uncharacterized enzymes
4*	0.019	COG0243 [C]	Anaerobic dehydrogenases, typically selenocysteine-containing
5	0.017	COG0537 [F,G,R]	Diadenosine tetraphosphate (Ap4A) hydrolase and other HIT family hydrolases
6	0.014	COG0399 [M]	Predicted pyridoxal phosphate-dependent enzyme apparently involved in regulation of cell wall biogenesis
7	0.012	COG1136 [V]	ABC-type antimicrobial peptide transport system, ATPase component
8	0.012	COG0373 [H]	Glutamyl-tRNA reductase
9*	0.012	COG0751 [J]	Glycyl-tRNA synthetase, beta subunit
10*	0.012	COG3005 [C]	Nitrate/TMAO reductases, membrane-bound tetraheme cytochrome c subunit
11	0.011	COG0482 [J]	Predicted tRNA(5-methylaminomethyl-2-thiouridylate) methyltransferase, contains the PP-loop ATPase domain
12	0.011	COG0043 [H]	3-polyprenyl-4-hydroxybenzoate decarboxylase and related decarboxylases
13*	0.011	COG0439 [I]	Biotin carboxylase
14	0.011	COG0577 [V]	ABC-type antimicrobial peptide transport system, permease component
15	0.011	COG0725 [P]	ABC-type molybdate transport system, periplasmic component
16	0.011	COG0521 [H]	Molybdopterin biosynthesis enzymes
17*	0.011	COG0348 [C]	Polyferredoxin
18	0.010	COG0019 [E]	Diaminopimelate decarboxylase

Table S 170: Module 148 – with rank 139 according to estimated information content.

Rank	Probability	OG	Description
1*	0.026	COG1148 [C]	Heterodisulfide reductase, subunit A and related polyferredoxins
2*	0.026	COG1014 [C]	Pyruvate:ferredoxin oxidoreductase and related 2-oxoacid:ferredoxin oxidoreductases, gamma subunit
3*	0.025	COG0535 [R]	Predicted Fe-S oxidoreductases
4	0.024	COG0845 [M]	Membrane-fusion protein
5*	0.021	COG1145 [C]	Ferredoxin
6*	0.019	COG1013 [C]	Pyruvate:ferredoxin oxidoreductase and related 2-oxoacid:ferredoxin oxidoreductases, beta subunit
7	0.018	COG1541 [H]	Coenzyme F390 synthetase
8*	0.018	COG0674 [C]	Pyruvate:ferredoxin oxidoreductase and related 2-oxoacid:ferredoxin oxidoreductases, alpha subunit
9	0.017	COG2206 [T]	HD-GYP domain
10*	0.017	COG0247 [C]	Fe-S oxidoreductase
11	0.015	COG2414 [C]	Aldehyde:ferredoxin oxidoreductase
12	0.014	COG3829 [K,T]	Transcriptional regulator containing PAS, AAA-type ATPase, and DNA-binding domains
13*	0.014	COG0303 [H]	Molybdopterin biosynthesis enzyme
14*	0.014	COG0778 [C]	Nitroreductase
15*	0.014	COG0574 [G]	Phosphoenolpyruvate synthase/pyruvate phosphate dikinase
16*	0.013	COG0655 [R]	Multimeric flavodoxin WrbA
17	0.012	COG2208 [T,K]	Serine phosphatase RsbU, regulator of sigma subunit
18*	0.012	COG0493 [E,R]	NADPH-dependent glutamate synthase beta chain and related oxidoreductases
19	0.012	COG1309 [K]	Transcriptional regulator
20	0.011	COG0618 [R]	Exopolyphosphatase-related proteins
21	0.011	COG1122 [P]	ABC-type cobalt transport system, ATPase component
22	0.011	COG0701 [R]	Predicted permeases
23*	0.010	COG1908 [C]	Coenzyme F420-reducing hydrogenase, delta subunit

Table S 171: Module 57 – with rank 140 according to estimated information content.

Rank	Probability	OG	Description
1*	0.063	COG0477 [G,E,P,R]	Permeases of the major facilitator superfamily
2*	0.053	COG0596 [R]	Predicted hydrolases or acyltransferases (alpha/beta hydrolase superfamily)
3*	0.037	COG0531 [E]	Amino acid transporters
4*	0.023	COG0451 [M,G]	Nucleoside-diphosphate-sugar epimerases
5*	0.020	COG0500 [Q,R]	SAM-dependent methyltransferases
6*	0.017	COG1063 [E,R]	Threonine dehydrogenase and related Zn-dependent dehydrogenases
7*	0.017	COG2197 [T,K]	Response regulator containing a CheY-like receiver domain and an HTH DNA-binding domain
8*	0.017	COG2771 [K]	DNA-binding HTH domain-containing proteins
9*	0.016	COG0640 [K]	Predicted transcriptional regulators
10	0.012	COG0791 [M]	Cell wall-associated hydrolases (invasion-associated proteins)
11	0.011	COG2188 [K]	Transcriptional regulators
12*	0.011	COG0365 [I]	Acyl-coenzyme A synthetases/AMP-(fatty) acid ligases
13*	0.011	COG0714 [R]	MoxR-like ATPases
14*	0.011	COG4977 [K]	Transcriptional regulator containing an amidase domain and an AraC-type DNA-binding HTH domain

Table S 172: Module 88 – with rank 141 according to estimated information content.

Rank	Probability	OG	Description
1*	0.017	COG1651 [O]	Protein-disulfide isomerase
2	0.015	COG0316 [S]	Uncharacterized conserved protein
3*	0.015	COG0508 [C]	Pyruvate/2-oxoglutarate dehydrogenase complex, dihydrolipoamide acyltransferase (E2) component, and related enzymes
4	0.013	COG0349 [J]	Ribonuclease D
5*	0.013	COG1249 [C]	Pyruvate/2-oxoglutarate dehydrogenase complex, dihydrolipoamide dehydrogenase (E3) component, and related enzymes
6	0.012	COG0271 [T]	Stress-induced morphogen (activity unknown)
7	0.010	COG0651 [C,P]	Formate hydrogenlyase subunit 3/Multisubunit Na+/H+ antiporter, MnhD subunit
8*	0.010	COG0708 [L]	Exonuclease III

Table S 173: Module 159 – with rank 142 according to estimated information content.

Rank	Probability	OG	Description
1*	0.052	COG0477 [G,E,P,R]	Permeases of the major facilitator superfamily
2*	0.033	COG0531 [E]	Amino acid transporters
3	0.024	COG2826 [L]	Transposase and inactivated derivatives, IS30 family
4*	0.023	COG0656 [R]	Aldo/keto reductases, related to diketogulonate reductase
5*	0.017	COG0406 [G]	Fructose-2,6-bisphosphatase
6*	0.016	COG0702 [M,G]	Predicted nucleoside-diphosphate-sugar epimerases
7	0.014	COG4690 [E]	Dipeptidase
8*	0.013	COG0589 [T]	Universal stress protein UspA and related nucleotide-binding proteins
9	0.013	COG1113 [E]	Gamma-aminobutyrate permease and related permeases
10*	0.012	COG0657 [I]	Esterase/lipase
11*	0.012	COG1957 [F]	Inosine-uridine nucleoside N-ribohydrolase
12	0.011	COG3548 [S]	Predicted integral membrane protein
13	0.011	COG4814 [R]	Uncharacterized protein with an alpha/beta hydrolase fold
14	0.011	COG2211 [G]	Na+/melibiose symporter and related transporters
15*	0.010	COG0624 [E]	Acetylornithine deacetylase/Succinyl-diaminopimelate desuccinylase and related deacylases

Table S 174: Module 3 – with rank 146 according to estimated information content.

Rank	Probability	OG	Description
1*	0.039	COG0642 [T]	Signal transduction histidine kinase
2*	0.033	COG0745 [T,K]	Response regulators consisting of a CheY-like receiver domain and a winged-helix DNA-binding domain
3*	0.023	COG1846 [K]	Transcriptional regulators
4	0.022	COG2885 [M]	Outer membrane protein and related peptidoglycan-associated (lipo)proteins
5	0.020	COG1192 [D]	ATPases involved in chromosome partitioning
6*	0.019	COG2801 [L]	Transposase and inactivated derivatives
7	0.019	COG2050 [Q]	Uncharacterized protein, possibly involved in aromatic compounds catabolism
8	0.017	COG0483 [G]	Archaeal fructose-1,6-bisphosphatase and related enzymes of inositol monophosphatase family
9*	0.016	COG0494 [L,R]	NTP pyrophosphohydrolases including oxidative damage repair enzymes
10	0.016	COG1196 [D]	Chromosome segregation ATPases
11	0.015	COG1393 [P]	Arsenate reductase and related proteins, glutaredoxin family
12	0.014	COG0626 [E]	Cystathionine beta-lyases/cystathionine gamma-synthases
13	0.013	COG0741 [M]	Soluble lytic murein transglycosylase and related regulatory proteins (some contain LysM/invasin domains)
14*	0.012	COG1670 [J]	Acetyltransferases, including N-acetylases of ribosomal proteins
15	0.011	COG2230 [M]	Cyclopropane fatty acid synthase and related methyltransferases
16	0.011	COG0776 [L]	Bacterial nucleoid DNA-binding protein
17	0.011	COG0640 [K]	Predicted transcriptional regulators
18	0.010	COG1530 [J]	Ribonucleases G and E

Table S 175: Module 152 – with rank 147 according to estimated information content.

Rank	Probability	OG	Description
1	0.042	COG1372 [L]	Intein/homing endonuclease
2	0.026	COG1672 [R]	Predicted ATPase (AAA+ superfamily)
3	0.023	COG0457 [R]	FOG: TPR repeat
4	0.016	COG0534 [V]	Na+-driven multidrug efflux pump
5	0.016	COG2244 [R]	Membrane protein involved in the export of O-antigen and teichoic acid
6	0.013	COG0037 [D]	Predicted ATPase of the PP-loop superfamily implicated in cell cycle control
7*	0.012	COG2111 [P]	Multisubunit Na+/H+ antiporter, MnhB subunit
8	0.012	COG0455 [D]	ATPases involved in chromosome partitioning
9*	0.012	COG1006 [P]	Multisubunit Na+/H+ antiporter, MnhC subunit
10*	0.012	COG1863 [P]	Multisubunit Na+/H+ antiporter, MnhE subunit
11	0.011	COG0516 [F]	IMP dehydrogenase/GMP reductase
12	0.010	COG1042 [C]	Acyl-CoA synthetase (NDP forming)
13	0.010	COG1226 [P]	Kef-type K+ transport systems, predicted NAD-binding component
14*	0.010	COG1320 [P]	Multisubunit Na+/H+ antiporter, MnhG subunit
15	0.010	COG4962 [U]	Flp pilus assembly protein, ATPase CpaF

Table S 176: Module 133 – with rank 148 according to estimated information content.

Rank	Probability	OG	Description
1	0.039	COG3539 [N,U]	P pilus assembly protein, pilin FimA
2	0.033	COG3121 [N,U]	P pilus assembly protein, chaperone PapD
3	0.027	COG1309 [K]	Transcriptional regulator
4*	0.024	COG1349 [K,G]	Transcriptional regulators of sugar metabolism
5*	0.023	COG0524 [G]	Sugar kinases, ribokinase family
6*	0.016	COG0437 [C]	Fe-S-cluster-containing hydrogenase components 1
7*	0.013	COG2186 [K]	Transcriptional regulators
8	0.012	COG0790 [R]	FOG: TPR repeat, SEL1 subfamily
9	0.011	COG1982 [E]	Arginine/lysine/ornithine decarboxylases
10*	0.011	COG1802 [K]	Transcriptional regulators
11*	0.011	COG1762 [G,T]	Phosphotransferase system mannitol/fructose-specific IIA domain (Ntr-type)
12	0.010	COG0625 [O]	Glutathione S-transferase
13	0.010	COG3772 [R]	Phage-related lysozyme (muraminidase)

Table S 177: Module 18 – with rank 150 according to estimated information content.

Rank	Probability	OG	Description
1*	0.020	COG0642 [T]	Signal transduction histidine kinase
2*	0.018	COG1131 [V]	ABC-type multidrug transport system, ATPase component
3*	0.016	COG1609 [K]	Transcriptional regulators
4	0.015	COG1307 [S]	Uncharacterized protein conserved in bacteria
5*	0.014	COG1136 [V]	ABC-type antimicrobial peptide transport system, ATPase component
6	0.013	COG1302 [S]	Uncharacterized protein conserved in bacteria
7	0.013	COG0744 [M]	Membrane carboxypeptidase (penicillin-binding protein)
8*	0.012	COG0583 [K]	Transcriptional regulator
9	0.011	COG1393 [P]	Arsenate reductase and related proteins, glutaredoxin family
10	0.010	COG0778 [C]	Nitroreductase

Table S 178: Module 78 – with rank 152 according to estimated information content.

Rank	Probability	OG	Description
1*	0.029	COG0406 [G]	Fructose-2,6-bisphosphatase
2	0.018	COG2409 [R]	Predicted drug exporters of the RND superfamily
3	0.017	COG0789 [K]	Predicted transcriptional regulators
4	0.015	COG0488 [R]	ATPase components of ABC transporters with duplicated ATPase domains
5	0.013	COG0266 [L]	Formamidopyrimidine-DNA glycosylase
6*	0.012	COG0483 [G]	Archaeal fructose-1,6-bisphosphatase and related enzymes of inositol monophosphatase family
7	0.012	COG1216 [R]	Predicted glycosyltransferases
8	0.012	COG0791 [M]	Cell wall-associated hydrolases (invasion-associated proteins)
9	0.011	COG1175 [G]	ABC-type sugar transport systems, permease components
10	0.011	COG5479 [M]	Uncharacterized protein potentially involved in peptidoglycan biosynthesis
11	0.010	COG0566 [J]	rRNA methylases

Table S 179: Module 142 – with rank 154 according to estimated information content.

Rank	Probability	OG	Description
1*	0.026	COG2204 [T]	Response regulator containing CheY-like receiver, AAA-type ATPase, and DNA-binding domains
2	0.023	COG2863 [C]	Cytochrome c553
3*	0.021	COG2202 [T]	FOG: PAS/PAC domain
4	0.017	COG2885 [M]	Outer membrane protein and related peptidoglycan-associated (lipo)proteins
5	0.013	COG1999 [R]	Uncharacterized protein SCO1/SenC/PrrC, involved in biogenesis of respiratory and photosynthetic systems
6	0.012	COG1131 [V]	ABC-type multidrug transport system, ATPase component
7	0.011	COG0247 [C]	Fe-S oxidoreductase
8	0.011	COG0543 [H,C]	2-polyprenylphenol hydroxylase and related flavodoxin oxidoreductases
9	0.011	COG1562 [I]	Phytoene/squalene synthetase
10	0.010	COG4654 [C]	Cytochrome c551/c552
11	0.010	COG0811 [U]	Biopolymer transport proteins

Table S 180: Module 31 – with rank 159 according to estimated information content.

Rank	Probability	OG	Description
1*	0.021	COG0243 [C]	Anaerobic dehydrogenases, typically selenocysteine-containing
2	0.016	COG0561 [R]	Predicted hydrolases of the HAD superfamily
3*	0.013	COG1145 [C]	Ferredoxin
4	0.012	COG1629 [P]	Outer membrane receptor proteins, mostly Fe transport
5	0.012	COG0859 [M]	ADP-heptose:LPS heptosyltransferase
6	0.011	COG1522 [K]	Transcriptional regulators
7	0.011	COG0564 [J]	Pseudouridylate synthases, 23S RNA-specific
8*	0.011	COG3381 [R]	Uncharacterized component of anaerobic dehydrogenases
9*	0.011	COG0437 [C]	Fe-S-cluster-containing hydrogenase components 1
10	0.010	COG0526 [O,C]	Thiol-disulfide isomerase and thioredoxins
11	0.010	COG2704 [R]	Anaerobic C4-dicarboxylate transporter

Table S 181: Module 12 – with rank 160 according to estimated information content.

Rank	Probability	OG	Description
1*	0.043	COG2207 [K]	AraC-type DNA-binding domain-containing proteins
2*	0.041	COG1595 [K]	DNA-directed RNA polymerase specialized sigma subunit, sigma24 homolog
3*	0.040	COG0642 [T]	Signal transduction histidine kinase
4*	0.030	COG0845 [M]	Membrane-fusion protein
5*	0.024	COG3712 [P,T]	Fe2+-dicitrate sensor, membrane component
6	0.019	COG3119 [P]	Arylsulfatase A and related enzymes
7*	0.017	COG0841 [V]	Cation/multidrug efflux pump
8*	0.015	COG3292 [T]	Predicted periplasmic ligand-binding sensor domain
9*	0.015	COG1538 [M,U]	Outer membrane protein
10*	0.015	COG3279 [K,T]	Response regulator of the LytR/AlgR family
11	0.014	COG4974 [L]	Site-specific recombinase XerD
12*	0.013	COG0784 [T]	FOG: CheY-like receiver
13*	0.013	COG2204 [T]	Response regulator containing CheY-like receiver, AAA-type ATPase, and DNA-binding domains
14*	0.012	COG0664 [T]	cAMP-binding proteins - catabolite gene activator and regulatory subunit of cAMP-dependent protein kinases
15*	0.011	COG1629 [P]	Outer membrane receptor proteins, mostly Fe transport
16	0.011	COG3537 [G]	Putative alpha-1,2-mannosidase
17*	0.011	COG3275 [T]	Putative regulator of cell autolysis

Table S 182: Module 118 – with rank 161 according to estimated information content.

Rank	Probability	OG	Description
1	0.026	COG0589 [T]	Universal stress protein UspA and related nucleotide-binding proteins
2	0.025	COG1522 [K]	Transcriptional regulators
3	0.022	COG0640 [K]	Predicted transcriptional regulators
4	0.017	COG0206 [D]	Cell division GTPase
5	0.015	COG1361 [M]	S-layer domain
6	0.015	COG0840 [N,T]	Methyl-accepting chemotaxis protein
7	0.013	COG0467 [T]	RecA-superfamily ATPases implicated in signal transduction
8	0.011	COG0392 [S]	Predicted integral membrane protein
9	0.011	COG1378 [K]	Predicted transcriptional regulators
10	0.010	COG3269 [R]	Predicted RNA-binding protein, contains TRAM domain
11	0.010	COG1681 [N]	Archaeal flagellins

Table S 183: Module 156 – with rank 162 according to estimated information content.

Rank	Probability	OG	Description
1*	0.020	COG0446 [R]	Uncharacterized NAD(FAD)-dependent dehydrogenases
2	0.020	COG4624 [R]	Iron only hydrogenase large subunit, C-terminal domain
3*	0.017	COG1924 [I]	Activator of 2-hydroxyglutaryl-CoA dehydratase (HSP70-class ATPase domain)
4	0.015	COG2025 [C]	Electron transfer flavoprotein, alpha subunit
5	0.015	COG1032 [C]	Fe-S oxidoreductase
6	0.014	COG2086 [C]	Electron transfer flavoprotein, beta subunit
7	0.013	COG2199 [T]	FOG: GGDEF domain
8	0.013	COG0619 [P]	ABC-type cobalt transport system, permease component CbiQ and related transporters
9*	0.012	COG1775 [E]	Benzoyl-CoA reductase/2-hydroxyglutaryl-CoA dehydratase subunit, BcrC/BadD/HgdB
10	0.011	COG0715 [P]	ABC-type nitrate/sulfonate/bicarbonate transport systems, periplasmic components
11	0.010	COG1116 [P]	ABC-type nitrate/sulfonate/bicarbonate transport system, ATPase component

Table S 184: Module 189 – with rank 163 according to estimated information content.

Rank	Probability	OG	Description
1	0.025	COG0526 [O,C]	Thiol-disulfide isomerase and thioredoxins
2*	0.025	COG1629 [P]	Outer membrane receptor proteins, mostly Fe transport
3*	0.024	COG0438 [M]	Glycosyltransferase
4*	0.023	COG3696 [P]	Putative silver efflux pump
5*	0.022	COG0845 [M]	Membrane-fusion protein
6	0.019	COG2165 [N,U]	Type II secretory pathway, pseudopilin PulG
7	0.019	COG0277 [C]	FAD/FMN-containing dehydrogenases
8*	0.018	COG1538 [M,U]	Outer membrane protein
9	0.017	COG3267 [U]	Type II secretory pathway, component ExeA (predicted ATPase)
10	0.014	COG1858 [P]	Cytochrome c peroxidase
11	0.013	COG1459 [N,U]	Type II secretory pathway, component PulF
12*	0.013	COG1596 [M]	Periplasmic protein involved in polysaccharide export
13*	0.011	COG0577 [V]	ABC-type antimicrobial peptide transport system, permease component
14	0.011	COG2010 [C]	Cytochrome c, mono- and diheme variants
15*	0.011	COG0841 [V]	Cation/multidrug efflux pump
16*	0.010	COG0296 [G]	1,4-alpha-glucan branching enzyme
17	0.010	COG0491 [R]	Zn-dependent hydrolases, including glyoxylases
18	0.010	NOG87598 [L]	Transposase

Table S 185: Module 147 – with rank 164 according to estimated information content.

Rank	Probability	OG	Description
1*	0.044	COG0583 [K]	Transcriptional regulator
2*	0.042	COG0784 [T]	FOG: CheY-like receiver
3*	0.031	COG0454 [K,R]	Histone acetyltransferase HPA2 and related acetyltransferases
4*	0.020	COG0346 [E]	Lactoylglutathione lyase and related lyases
5*	0.016	COG3979 [R]	Uncharacterized protein contain chitin-binding domain type 3
6	0.014	COG0110 [R]	Acetyltransferase (isoleucine patch superfamily)
7*	0.014	COG0457 [R]	FOG: TPR repeat
8*	0.012	COG0517 [R]	FOG: CBS domain
9*	0.012	COG2206 [T]	HD-GYP domain
10*	0.010	COG1280 [E]	Putative threonine efflux protein
11	0.010	COG0733 [R]	Na+-dependent transporters of the SNF family

Table S 186: Module 139 – with rank 165 according to estimated information content.

Rank	Probability	OG	Description
1	0.031	COG5295 [U,W]	Autotransporter adhesin
2	0.018	COG0583 [K]	Transcriptional regulator
3	0.017	COG1396 [K]	Predicted transcriptional regulators
4	0.017	COG3676 [L]	Transposase and inactivated derivatives
5	0.015	COG0778 [C]	Nitroreductase
6	0.015	COG2369 [S]	Uncharacterized protein, homolog of phage Mu protein gp30
7	0.015	COG3210 [U]	Large exoproteins involved in heme utilization or adhesion
8*	0.015	COG3842 [E]	ABC-type spermidine/putrescine transport systems, ATPase components
9*	0.013	COG0687 [E]	Spermidine/putrescine-binding periplasmic protein
10	0.013	COG4383 [S]	Mu-like prophage protein gp29
11	0.013	COG2932 [K]	Predicted transcriptional regulator
12	0.012	COG0791 [M]	Cell wall-associated hydrolases (invasion-associated proteins)
13	0.011	COG0733 [R]	Na+-dependent transporters of the SNF family
14*	0.010	COG1178 [P]	ABC-type Fe3+ transport system, permease component

Table S 187: Module 115 – with rank 167 according to estimated information content.

Rank	Probability	OG	Description
1*	0.041	COG0642 [T]	Signal transduction histidine kinase
2	0.039	COG3203 [M]	Outer membrane protein (porin)
3*	0.032	COG1028 [I,Q,R]	Dehydrogenases with different specificities (related to short-chain alcohol dehydrogenases)
4*	0.025	COG2207 [K]	AraC-type DNA-binding domain-containing proteins
5*	0.020	COG0834 [E,T]	ABC-type amino acid transport/signal transduction systems, periplasmic component/domain
6	0.019	COG5295 [U,W]	Autotransporter adhesin
7*	0.016	COG0457 [R]	FOG: TPR repeat
8	0.015	COG0582 [L]	Integrase
9*	0.013	COG1804 [C]	Predicted acyl-CoA transferases/carnitine dehydratase
10*	0.013	COG0318 [I,Q]	Acyl-CoA synthetases (AMP-forming)/AMP-acid ligases II
11	0.011	COG0607 [P]	Rhodanese-related sulfurtransferase
12*	0.010	COG0500 [Q,R]	SAM-dependent methyltransferases
13	0.010	COG0859 [M]	ADP-heptose:LPS heptosyltransferase

Table S 188: Module 46 – with rank 168 according to estimated information content.

Rank	Probability	OG	Description
1*	0.039	COG2197 [T,K]	Response regulator containing a CheY-like receiver domain and an HTH DNA-binding domain
2*	0.038	COG0642 [T]	Signal transduction histidine kinase
3*	0.026	COG0589 [T]	Universal stress protein UspA and related nucleotide-binding proteins
4*	0.019	COG1629 [P]	Outer membrane receptor proteins, mostly Fe transport
5*	0.017	COG0697 [G,E,R]	Permeases of the drug/metabolite transporter (DMT) superfamily
6*	0.015	COG0119 [E]	Isopropylmalate/homocitrate/citramalate synthases
7*	0.014	COG0277 [C]	FAD/FMN-containing dehydrogenases
8*	0.014	COG0631 [T]	Serine/threonine protein phosphatase
9	0.013	COG0741 [M]	Soluble lytic murein transglycosylase and related regulatory proteins (some contain LysM/invasin domains)
10	0.012	COG0501 [O]	Zn-dependent protease with chaperone function
11*	0.012	COG1595 [K]	DNA-directed RNA polymerase specialized sigma subunit, sigma24 homolog
12	0.011	COG1807 [M]	4-amino-4-deoxy-L-arabinose transferase and related glycosyltransferases of PMT family
13	0.011	COG0431 [R]	Predicted flavoprotein
14*	0.010	COG4566 [T]	Response regulator
15	0.010	COG2329 [R]	Uncharacterized enzyme involved in biosynthesis of extracellular polysaccharides

Table S 189: Module 124 – with rank 169 according to estimated information content.

Rank	Probability	OG	Description
1*	0.045	COG1020 [Q]	Non-ribosomal peptide synthetase modules and related proteins
2*	0.042	COG2202 [T]	FOG: PAS/PAC domain
3*	0.034	COG0477 [G,E,P,R]	Permeases of the major facilitator superfamily
4*	0.023	COG0665 [E]	Glycine/D-amino acid oxidases (deaminating)
5*	0.022	COG0687 [E]	Spermidine/putrescine-binding periplasmic protein
6*	0.021	COG1960 [I]	Acyl-CoA dehydrogenases
7*	0.015	COG0446 [R]	Uncharacterized NAD(FAD)-dependent dehydrogenases
8*	0.011	COG1335 [Q]	Amidases related to nicotinamidase
9*	0.011	COG0174 [E]	Glutamine synthetase
10*	0.010	COG1177 [E]	ABC-type spermidine/putrescine transport system, permease component II

Table S 190: Module 138 – with rank 172 according to estimated information content.

Rank	Probability	OG	Description
1	0.023	COG0609 [P]	ABC-type Fe3+-siderophore transport system, permease component
2*	0.017	COG1012 [C]	NAD-dependent aldehyde dehydrogenases
3*	0.016	COG0745 [T,K]	Response regulators consisting of a CheY-like receiver domain and a winged-helix DNA-binding domain
4	0.014	COG1473 [R]	Metal-dependent amidase/aminoacylase/carboxypeptidase
5*	0.014	COG2197 [T,K]	Response regulator containing a CheY-like receiver domain and an HTH DNA-binding domain
6*	0.014	COG0346 [E]	Lactoylglutathione lyase and related lyases
7	0.013	COG0584 [C]	Glycerophosphoryl diester phosphodiesterase
8*	0.012	COG0457 [R]	FOG: TPR repeat
9	0.012	COG1173 [E,P]	ABC-type dipeptide/oligopeptide/nickel transport systems, permease components
10*	0.012	COG1113 [E]	Gamma-aminobutyrate permease and related permeases

Table S 191: Module 40 – with rank 176 according to estimated information content.

Rank	Probability	OG	Description
1*	0.037	COG0834 [E,T]	ABC-type amino acid transport/signal transduction systems, periplasmic component/domain
2	0.029	COG0500 [Q,R]	SAM-dependent methyltransferases
3*	0.019	COG0437 [C]	Fe-S-cluster-containing hydrogenase components 1
4*	0.019	COG0243 [C]	Anaerobic dehydrogenases, typically selenocysteine-containing
5*	0.017	COG1301 [C]	Na+/H+-dicarboxylate symporters
6	0.016	COG0789 [K]	Predicted transcriptional regulators
7*	0.016	COG1053 [C]	Succinate dehydrogenase/fumarate reductase, flavoprotein subunit
8	0.014	COG0582 [L]	Integrase
9	0.013	COG0596 [R]	Predicted hydrolases or acyltransferases (alpha/beta hydrolase superfamily)
10	0.012	COG2801 [L]	Transposase and inactivated derivatives
11*	0.012	COG0612 [R]	Predicted Zn-dependent peptidases
12*	0.011	COG0841 [V]	Cation/multidrug efflux pump
13	0.010	COG1960 [I]	Acyl-CoA dehydrogenases

Table S 192: Module 56 – with rank 177 according to estimated information content.

Rank	Probability	OG	Description
1*	0.026	COG2114 [T]	Adenylate cyclase, family 3 (some proteins contain HAMP domain)
2*	0.024	COG2208 [T,K]	Serine phosphatase RsbU, regulator of sigma subunit
3*	0.019	COG4886 [S]	Leucine-rich repeat (LRR) protein
4*	0.018	COG1960 [I]	Acyl-CoA dehydrogenases
5*	0.018	COG0664 [T]	cAMP-binding proteins - catabolite gene activator and regulatory subunit of cAMP-dependent protein kinases
6	0.017	COG0739 [M]	Membrane proteins related to metalloendopeptidases
7*	0.016	COG1595 [K]	DNA-directed RNA polymerase specialized sigma subunit, sigma24 homolog
8*	0.015	COG0596 [R]	Predicted hydrolases or acyltransferases (alpha/beta hydrolase superfamily)
9	0.015	COG3291 [R]	FOG: PKD repeat
10*	0.013	COG1028 [I,Q,R]	Dehydrogenases with different specificities (related to short-chain alcohol dehydrogenases)
11*	0.013	COG2203 [T]	FOG: GAF domain
12*	0.012	COG0666 [R]	FOG: Ankyrin repeat
13	0.012	COG1696 [M]	Predicted membrane protein involved in D-alanine export

Table S 193: Module 155 – with rank 179 according to estimated information content.

Rank	Probability	OG	Description
1	0.028	COG2885 [M]	Outer membrane protein and related peptidoglycan-associated (lipo)proteins
2	0.027	COG0661 [R]	Predicted unusual protein kinase
3	0.023	COG1249 [C]	Pyruvate/2-oxoglutarate dehydrogenase complex, dihydrolipoamide dehydrogenase (E3) component, and related enzymes
4*	0.016	COG0655 [R]	Multimeric flavodoxin WrbA
5	0.015	COG1230 [P]	Co/Zn/Cd efflux system component
6	0.014	COG0730 [R]	Predicted permeases
7	0.014	COG1251 [C]	NAD(P)H-nitrite reductase
8*	0.013	COG0431 [R]	Predicted flavoprotein
9	0.013	COG3687 [R]	Predicted metal-dependent hydrolase

Table S 194: Module 1 – with rank 180 according to estimated information content.

Rank	Probability	OG	Description
1	0.026	COG0583 [K]	Transcriptional regulator
2	0.021	COG0607 [P]	Rhodanese-related sulfurtransferase
3	0.020	COG0463 [M]	Glycosyltransferases involved in cell wall biogenesis
4*	0.016	COG0436 [E]	Aspartate/tyrosine/aromatic aminotransferase
5	0.013	COG3201 [H]	Nicotinamide mononucleotide transporter
6	0.012	COG3049 [M]	Penicillin V acylase and related amidases
7	0.011	COG0318 [I,Q]	Acyl-CoA synthetases (AMP-forming)/AMP-acid ligases II
8	0.011	COG0760 [O]	Parvulin-like peptidyl-prolyl isomerase
9*	0.011	COG0025 [P]	NhaP-type Na+/H+ and K+/H+ antiporters
10*	0.010	COG0814 [E]	Amino acid permeases
11*	0.010	COG0076 [E]	Glutamate decarboxylase and related PLP-dependent proteins

Table S 195: Module 109 – with rank 181 according to estimated information content.

Rank	Probability	OG	Description
1*	0.035	COG0454 [K,R]	Histone acetyltransferase HPA2 and related acetyltransferases
2*	0.021	COG3321 [Q]	Polyketide synthase modules and related proteins
3	0.020	COG1506 [E]	Dipeptidyl aminopeptidases/acylaminoacyl-peptidases
4	0.019	NOG44148 [S]	Annotation not available
5*	0.018	COG1024 [I]	Enoyl-CoA hydratase/carnithine racemase
6	0.017	COG0564 [J]	Pseudouridylate synthases, 23S RNA-specific
7	0.014	COG1404 [O]	Subtilisin-like serine proteases
8*	0.012	COG0318 [I,Q]	Acyl-CoA synthetases (AMP-forming)/AMP-acid ligases II
9	0.012	COG4805 [S]	Uncharacterized protein conserved in bacteria
10	0.011	COG0526 [O,C]	Thiol-disulfide isomerase and thioredoxins
11	0.011	COG2050 [Q]	Uncharacterized protein, possibly involved in aromatic compounds catabolism

Table S 196: Module 75 – with rank 184 according to estimated information content.

Rank	Probability	OG	Description
1*	0.069	COG0477 [G,E,P,R]	Permeases of the major facilitator superfamily
2*	0.022	COG3539 [N,U]	P pilus assembly protein, pilin FimA
3*	0.016	COG3468 [M,U]	Type V secretory pathway, adhesin AidA
4	0.015	COG1113 [E]	Gamma-aminobutyrate permease and related permeases
5*	0.013	COG0841 [V]	Cation/multidrug efflux pump

Table S 197: Module 55 – with rank 190 according to estimated information content.

Rank	Probability	OG	Description
1*	0.022	COG0745 [T,K]	Response regulators consisting of a CheY-like receiver domain and a winged-helix DNA-binding domain
2*	0.020	COG1846 [K]	Transcriptional regulators
3	0.017	COG3293 [L]	Transposase and inactivated derivatives
4	0.016	COG3637 [M]	Opacity protein and related surface antigens
5*	0.015	COG2202 [T]	FOG: PAS/PAC domain
6	0.014	COG1376 [S]	Uncharacterized protein conserved in bacteria
7*	0.013	COG0494 [L,R]	NTP pyrophosphohydrolases including oxidative damage repair enzymes
8*	0.013	COG0784 [T]	FOG: CheY-like receiver
9*	0.013	COG0346 [E]	Lactoylglutathione lyase and related lyases
10*	0.012	COG0845 [M]	Membrane-fusion protein
11*	0.012	COG0483 [G]	Archaeal fructose-1,6-bisphosphatase and related enzymes of inositol monophosphatase family
12*	0.012	COG0673 [R]	Predicted dehydrogenases and related proteins

Table S 198: Module 5 – with rank 191 according to estimated information content.
