# Supplementary figures and images for: Inferring functional modules of protein families with probabilistic topic models
Source: BMC Bioinformatics. 2011 May 9;12:141. doi: 10.1186/1471-2105-12-141 (PMC3098182; doi:10.1186/1471-2105-12-141)

A

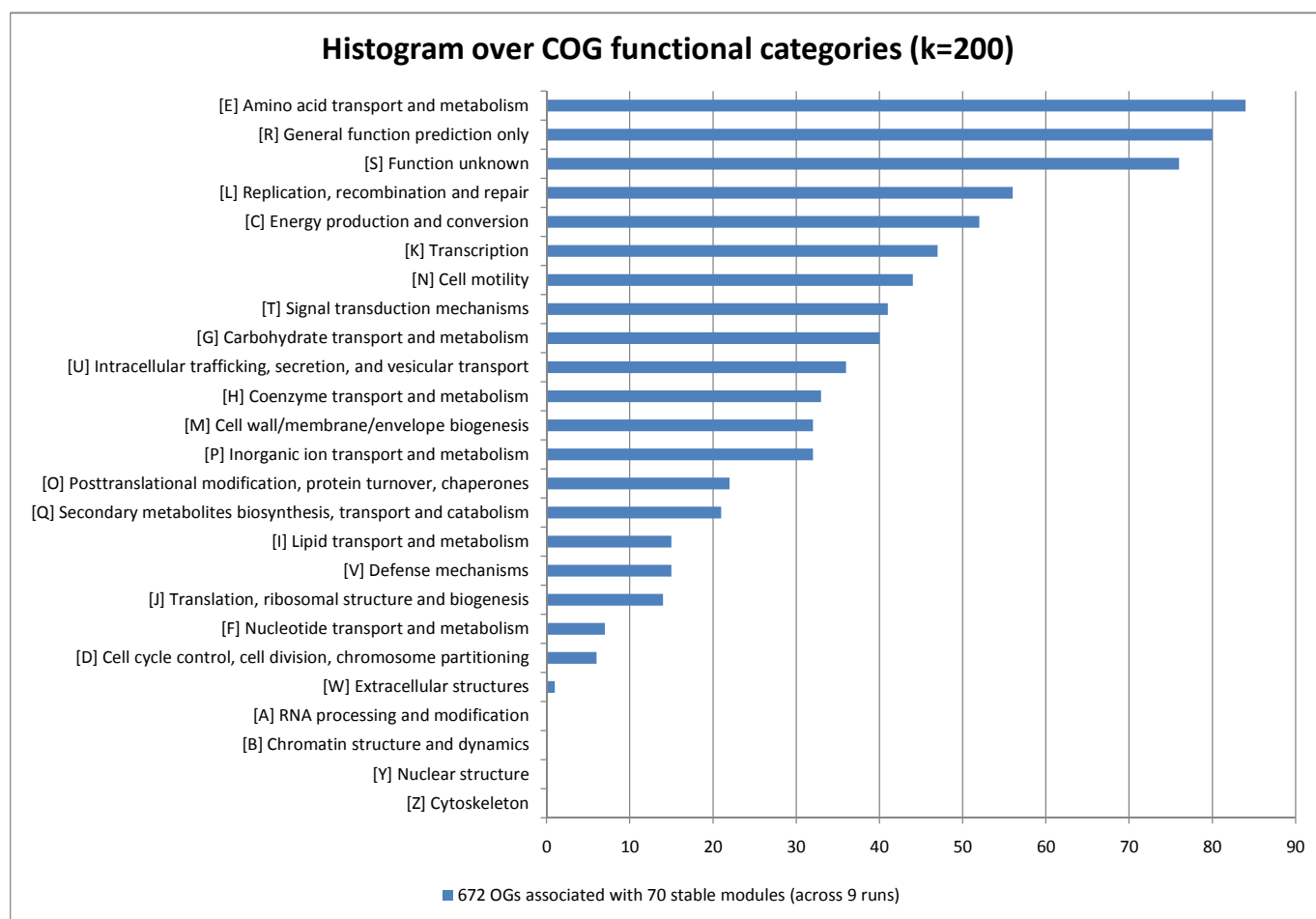

B

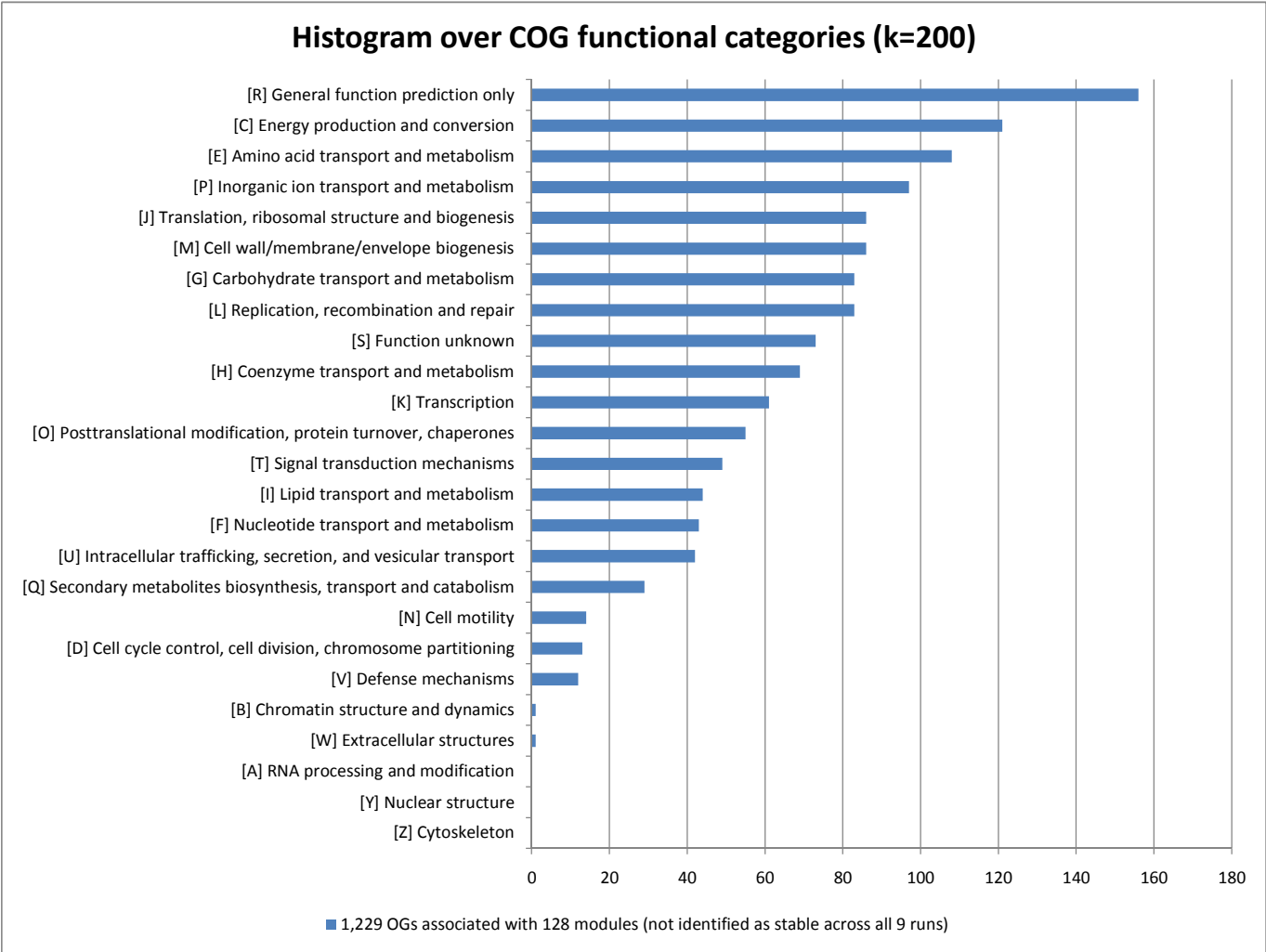

Supplement: Additional file 2 — Comparison of histograms over COG functional categories. Comparison of two histograms over COG functional categories for (A) 70 stable modules and (B) modules that could not be tracked across all nine runs. [file 1471-2105-12-141-S2.PDF]

## PORPHYRIN AND CHLOROPHYLL METABOLISM

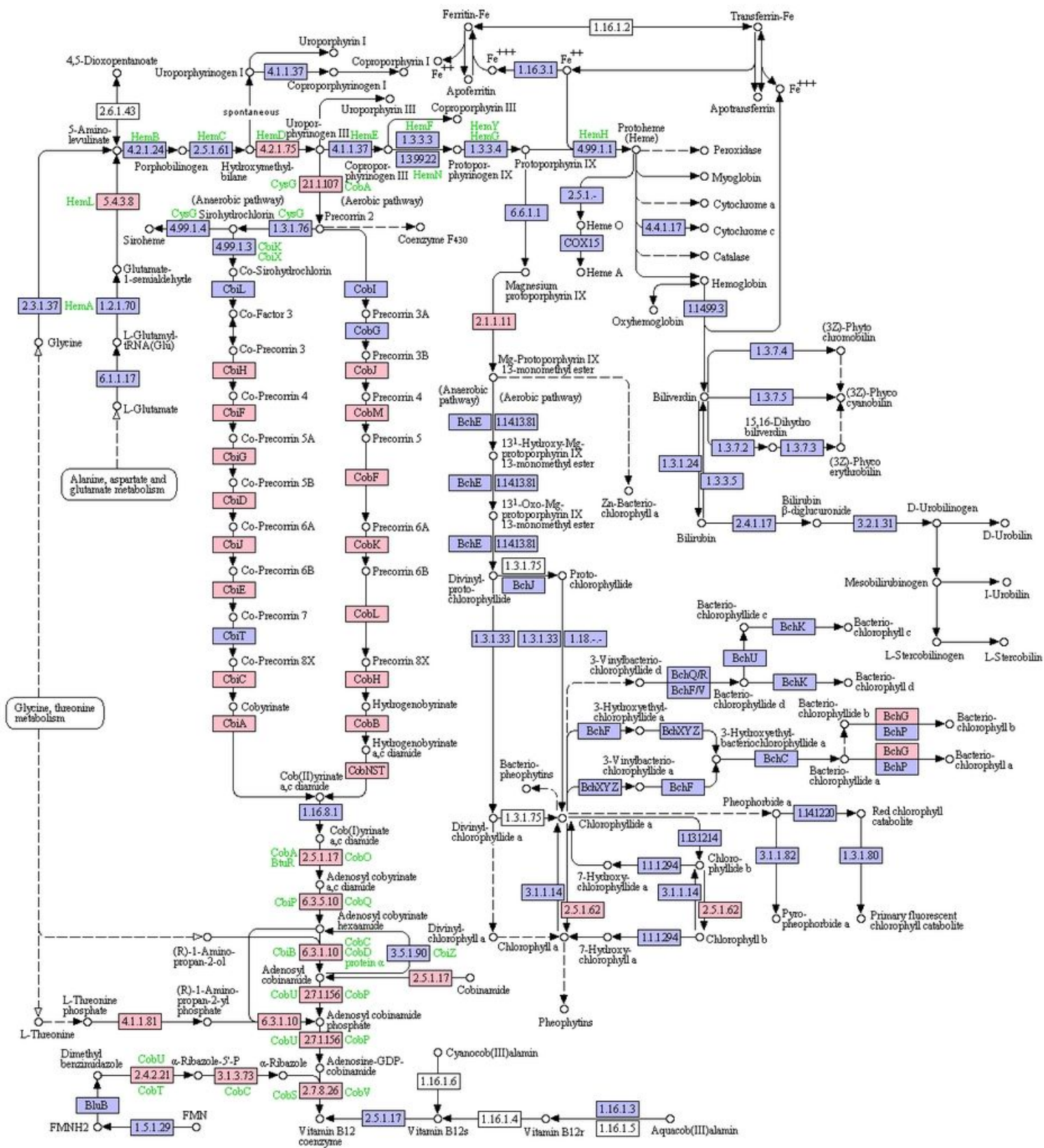

Supplement: Additional file 4 — Visualized matches to the KEGG pathway 'Porphyrin and chlorophyll metabolism'. KO terms that are matched by the OGs of the respective potential functional module are highlighted in pink. [file 1471-2105-12-141-S4.PDF]

## Ribosomal RNAs

|                                  |     |    |      |     |
|----------------------------------|-----|----|------|-----|
| Bacteria / Archaea<br>Eukaryotes | 23S | 5S |      | 16S |
|                                  | 25S | 5S | 5.8S | 18S |

## Ribosomal proteins

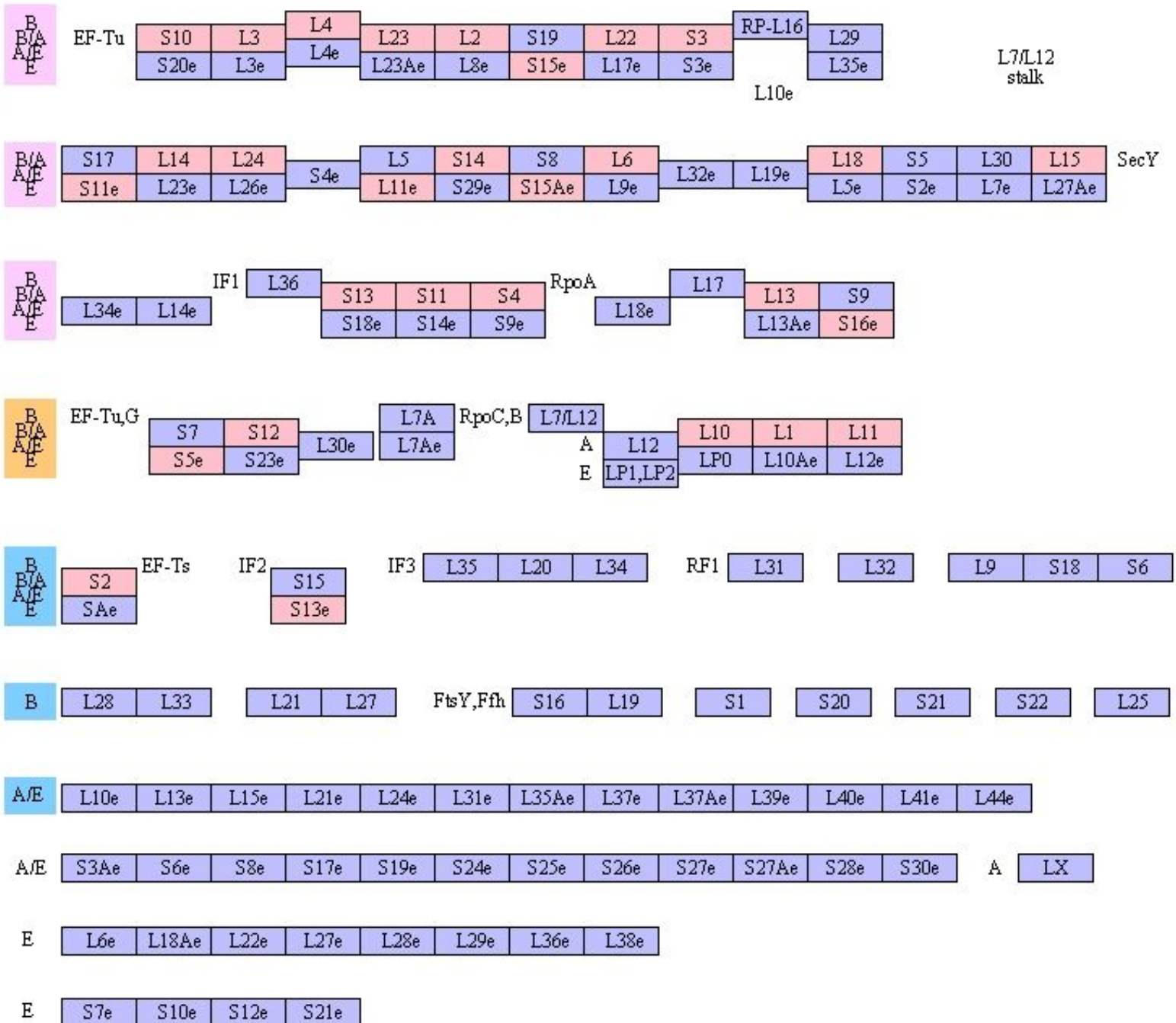

Supplement: Additional file 6 — Visualized matches to the KEGG pathway 'Ribosome'. KO terms that are matched by the OGs of the respective potential functional module are highlighted in pink. [file 1471-2105-12-141-S6.PDF]

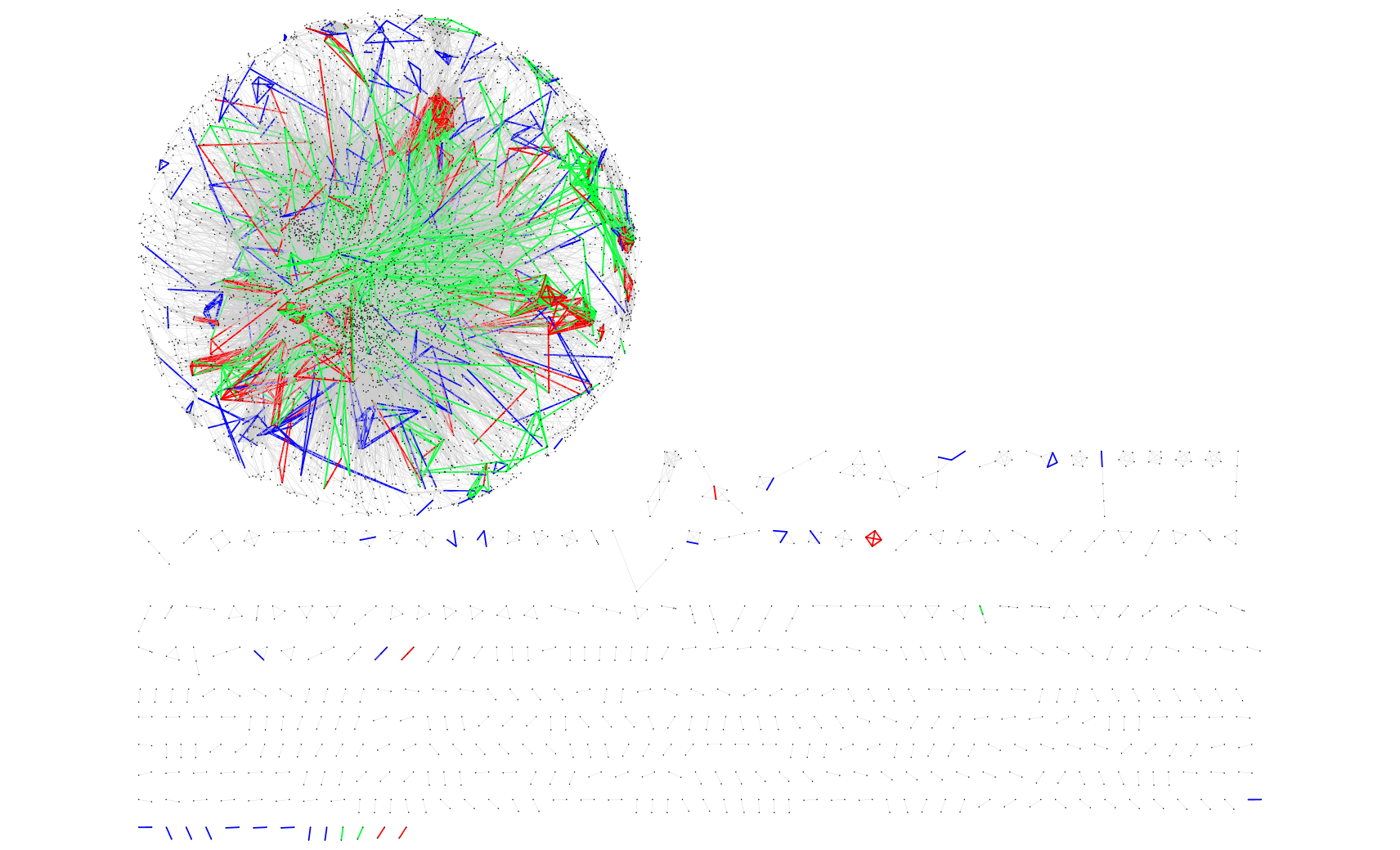

Supplement: Additional file 8 — Visualization of the functional network spanned by the OG pairs of the reference set. Pairwise functional interactions are defined by the reference set as edges between OGs in a network graph. The subset of verified pairwise predictions from the modules is shown in green, whereas the subset of verified predictions by pairwise co-occurrence profiling is shown in blue. Functional interactions that are predicted by both methods are colored red, and those not detected by any of the methods are shown in gray. [file 1471-2105-12-141-S8.PNG]
