# Supplementary material for: Inferring functional modules of protein families with probabilistic topic models
Source: BMC Bioinformatics. 2011 May 9;12:141. doi: 10.1186/1471-2105-12-141 (PMC3098182; doi:10.1186/1471-2105-12-141)
Supplement: Additional file 3 — Profile of KEGG pathways with at least six matches to one of the 198 modules. Supplementary Table S1: List of KEGG pathways with at least six matches of their KO terms to one of the 198 potential functional modules inferred in the exemplary run with k = 200. [file 1471-2105-12-141-S3.PDF]

Table S1

KEGG-Name	Modules
ABC transporters	12
Two-component system	10
Porphyrin and chlorophyll metabolism	3
Oxidative phosphorylation	2
Flagellar assembly	1
Bacterial secretion system	3
Purine metabolism	3
Amino sugar and nucleotide sugar metabolism	3
Ribosome	1
Phosphotransferase system (PTS)	2
Phenylalanine, tyrosine and tryptophan biosynthesis	1
Aminoacyl-tRNA biosynthesis	1
Arginine and proline metabolism	1
Phenylalanine metabolism	1
Fructose and mannose metabolism	1
Riboflavin metabolism	1
Propanoate metabolism	1
Peptidoglycan biosynthesis	1
Benzoate degradation via CoA ligation	1
Starch and sucrose metabolism	1
Galactose metabolism	1
Pyruvate metabolism	1
Butanoate metabolism	1
