# Supplementary material for: Inferring functional modules of protein families with probabilistic topic models
Source: BMC Bioinformatics. 2011 May 9;12:141. doi: 10.1186/1471-2105-12-141 (PMC3098182; doi:10.1186/1471-2105-12-141)
Supplement: Additional file 5 — 'Ribosome'-related functional module. Supplementary Table S1: OGs of the 'Ribosome'-related functional module that was identified in nine runs with k = 400. [file 1471-2105-12-141-S5.PDF]

'Ribosome' related module

Sebastian Gil Anthony Konietzny

April 28, 2011

Table S 1: Module 'Ribosome' (stable across many runs with k=200; stable across all nine runs with k=400. Displayed table is taken from runs with k=400.).

Rank	Probability	OG	Description
1*	0.018	COG0051 [J]	Ribosomal protein S10
2*	0.018	COG0100 [J]	Ribosomal protein S11
3*	0.017	COG0013 [J]	Alanyl-tRNA synthetase
4*	0.017	COG0124 [J]	Histidyl-tRNA synthetase
5*	0.017	COG0103 [J]	Ribosomal protein S9
6*	0.017	COG0541 [U]	Signal recognition particle GTPase
7*	0.017	COG0244 [J]	Ribosomal protein L10
8*	0.017	COG0250 [K]	Transcription antiterminator
9*	0.017	COG0522 [J]	Ribosomal protein S4 and related proteins
10*	0.016	COG0048 [J]	Ribosomal protein S12
11*	0.016	COG0162 [J]	Tyrosyl-tRNA synthetase
12*	0.016	COG0087 [J]	Ribosomal protein L3
13*	0.016	COG0199 [J]	Ribosomal protein S14
14*	0.016	COG0088 [J]	Ribosomal protein L4
15*	0.015	COG0143 [J]	Methionyl-tRNA synthetase
16*	0.015	COG0089 [J]	Ribosomal protein L23
17*	0.015	COG0256 [J]	Ribosomal protein L18
18*	0.015	COG0528 [F]	Uridylate kinase
19*	0.015	COG0060 [J]	Isoleucyl-tRNA synthetase
20*	0.015	COG0092 [J]	Ribosomal protein S3
21*	0.015	COG0201 [U]	Preprotein translocase subunit SecY
22*	0.015	COG0231 [J]	Translation elongation factor P (EF-P)/translation initiation factor 5A (eIF-5A)
23*	0.014	COG0049 [J]	Ribosomal protein S7
24*	0.014	COG0202 [K]	DNA-directed RNA polymerase, alpha subunit/40 kD subunit
25*	0.014	COG0358 [L]	DNA primase (bacterial type)
26*	0.014	COG0441 [J]	Threonyl-tRNA synthetase
27*	0.014	COG0495 [J]	Leucyl-tRNA synthetase
28*	0.014	COG0099 [J]	Ribosomal protein S13
29*	0.014	COG0016 [J]	Phenylalanyl-tRNA synthetase alpha subunit
30*	0.014	COG0185 [J]	Ribosomal protein S19
31*	0.014	COG0084 [L]	Mg-dependent DNase
32*	0.014	COG0080 [J]	Ribosomal protein L11
33*	0.014	COG0126 [G]	3-phosphoglycerate kinase
34*	0.014	COG0361 [J]	Translation initiation factor 1 (IF-1)
35*	0.014	COG0090 [J]	Ribosomal protein L2

Rank	Probability	OG	Description
36*	0.013	COG0091 [J]	Ribosomal protein L22
37*	0.013	COG0180 [J]	Tryptophanyl-tRNA synthetase
38*	0.013	COG0552 [U]	Signal recognition particle GTPase
39	0.013	COG0221 [C]	Inorganic pyrophosphatase
40*	0.013	COG0081 [J]	Ribosomal protein L1
41*	0.013	COG0258 [L]	5-3 exonuclease (including N-terminal domain of PolI)
42*	0.013	COG0468 [L]	RecA/RadA recombinase
43	0.013	COG0533 [O]	Metal-dependent proteases with possible chaperone activity
44*	0.013	COG0575 [I]	CDP-diglyceride synthetase
45*	0.013	COG0030 [J]	Dimethyladenosine transferase (rRNA methylation)
46*	0.012	COG0525 [J]	Valyl-tRNA synthetase
47*	0.012	COG0096 [J]	Ribosomal protein S8
48*	0.012	COG0072 [J]	Phenylalanyl-tRNA synthetase beta subunit
49*	0.012	COG0172 [J]	Seryl-tRNA synthetase
50*	0.012	COG0052 [J]	Ribosomal protein S2
51*	0.012	COG0024 [J]	Methionine aminopeptidase
52*	0.012	COG0442 [J]	Prolyl-tRNA synthetase
53*	0.012	COG0198 [J]	Ribosomal protein L24
54*	0.011	COG0186 [J]	Ribosomal protein S17
55*	0.011	COG0550 [L]	Topoisomerase IA
56*	0.011	COG0149 [G]	Triosephosphate isomerase
57*	0.011	COG0592 [L]	DNA polymerase sliding clamp subunit (PCNA homolog)
58*	0.011	COG0094 [J]	Ribosomal protein L5
59*	0.011	COG0184 [J]	Ribosomal protein S15P/S13E
60*	0.011	COG0112 [E]	Glycine/serine hydroxymethyltransferase
61*	0.011	COG0097 [J]	Ribosomal protein L6P/L9E
62*	0.011	COG0102 [J]	Ribosomal protein L13
63*	0.011	COG0480 [J]	Translation elongation factors (GTPases)
64*	0.010	COG0200 [J]	Ribosomal protein L15
65*	0.010	COG0093 [J]	Ribosomal protein L14
66*	0.010	COG0532 [J]	Translation initiation factor 2 (IF-2; GTPase)
