# Supplementary material for: Inferring functional modules of protein families with probabilistic topic models
Source: BMC Bioinformatics. 2011 May 9;12:141. doi: 10.1186/1471-2105-12-141 (PMC3098182; doi:10.1186/1471-2105-12-141)
Supplement: Additional file 7 — Supplementary note. This document includes additional details of the comparison of results for different settings of k, and a discussion on the distribution of the probability weights of the modules across the analyzed genomes. [file 1471-2105-12-141-S7.PDF]

– SUPPLEMENTARY NOTE –

Inferring functional modules of protein families with probabilistic topic models

Sebastian GA Konietzny, Laura Dietz and Alice C McHardy

Analysis of results with alternative settings for the number of topics inferred with our method

We matched the stable modules from each experiment against the KEGG reference pathway database to compute profiles of pathways that posses at least 6 hits to any one of the modules. For *k*=200 and *k*=400 this yielded most diverse profiles with 20 different KEGG pathways. However, a single KEGG pathway may be mapped by several of the identified stable modules, and we observed higher numbers of occurrences of the matched KEGG pathways for *k*=400 than for *k*=200. Moreover, the KEGG profile over the complete set of 400 inferred modules is more diverse than for *k*=200.

The stable modules which are discussed in more detail, i.e. 'ChemoTax', 'Flagell' and 'VitB12', could be identified in all runs for *k* = 200, 300, 400 and 500. For *k* = 100, especially the 'VitB12' module was unstable and could not be identified in two of three runs. The 'Ribosome' related module wasn't found for *k* = 100 at all.

In total, 256 OGs belong to the sets of identified stable topics across all tested settings of *k*. The most abundant COG functional categories are general function prediction only (R), amino acid transport and metabolism (E) as well as signal transduction mechanisms (T) and carbohydrate transport and metabolism (G). This set of 256 re-occurring OGs makes up 72.8% (k=100), 26.9% (k=200), 19.8% (k=300), 15.2% (k=400) und 15.1% (k=500) of the OGs associated with the corresponding sets of stable modules.

We observed a few modules to be more stable across runs for higher values of *k*. For example, the presented 'Ribosome' related module could be tracked across nine runs for *k*=400, but it was less stable across runs with k=*200*. In general, the set of stable modules for *k*=400 and *k*=500 is larger than for runs with smaller *k*. Moreover, the averaged coverage values are surprisingly high for larger *k,* though more and larger modules are evaluated, which also comprise a larger set of distinct OGs. Thus, the modules capture a larger interaction network for higher numbers of inferred topics.

In summary, we suggest to use a large number for *k,* i.e. $k \geq 200$. However, the more modules are being inferred, the more difficult it becomes to extract the relevant modules from the set, especially if one is interested in finding new groupings of OGs that do not map to known pathways, such as KEGG pathways. In these cases one can use the coverage values as guidance to finding interesting, functionally coherent groupings, as shown for the 'ChemoTax' module in the presented study. Also, enrichments of COG functional categories are useful. An alternative is to query the set of modules for a list of OGs of interest.

Distribution of the probability weights of the modules across the analyzed genomes

Every genome defines a probability distribution, *P(PF-Module|Genome)*, that describes for each of the *k* inferred modules the probability to be encoded in the respective genome (Methods). The figure in Additional file **9** visualizes the distributions of probability weights for every genome. The majority of genomes features only a small subset of modules with a high probability, instead of giving equal probability to all modules. We also observed that some PF-modules receive high weights for almost all of the genomes. Analysis of the corresponding topics revealed that these correspond to empty modules, meaning that OGs with highest probability in the topic distribution do not exceed the threshold criterion of *C=0.01*. This means that none of the OGs of the vocabulary are specifically associated with these modules, and thus it is likely that they serve somehow as ‘default topics’ which associate with OGs without significant co-occurrence signals.

For the ‘Flagell’ module we used the module’s probability weights to select a subset of genomes where this module seems to be encoded. As cutoff threshold for selecting potentially ‘Flagell’ encoding genomes we chose the mean of the weights of the ‘Flagell’ module across all genomes. Thus, the set of genomes was divided into 228 potentially ‘motile’ and 347 ‘nonmotile’ genomes. We then acquired phenotype annotations for the genomes from the GOLD-Genomes Online Database [2]. In total, 302 genomes were thus labeled as ‘truly motile’, whereas 215 genomes were flagged as ‘truly nonmotile’. With this setting, our method achieved a recall of 65.2% and a very high precision of 94.3%. As most of the genomes with a predicted ‘motility’ are indeed motile, this provides further evidence for the relation of the inferred module to ‘motility’ of the organism. The weaker value of recall could be explained by the fact that within some of the genomes only a smaller subset of the OGs related to the module were annotated, resulting in a decreased probability weight of the module for these genomes.

Choosing hyperparameters α and β

We tested performance of the Latent Dirichlet Allocation model for different settings of the hyperparameters α and β. In many applications of LDA, settings

$$\alpha = 1/k \qquad \text{and} \qquad \beta = \frac{1}{size(vocabulary)}$$

were used. However, we obtained significantly worse results with this setting. Parameter values used in our study, which yielded best results, are the default settings of the ‘topic modeling toolbox’ [1], which is an alternative implementation of the LDA model.

References:

1. Steyvers M, Griffiths T (Authors): **Topic Modeling Toolbox 1.3.2** [http://psiexp.ss.uci.edu/research/programs_data/toolbox.htm]

2. Liolios K, Mavromatis K, Tavernarakis N, Kyrpides NC: **The Genomes On Line Database (GOLD) in 2007: status of genomic and metagenomic projects and their associated metadata**. *Nucleic acids research (*2008) 36, no. Database issue (January): D475-9.
