# Supplementary material for: Inferring functional modules of protein families with probabilistic topic models
Source: BMC Bioinformatics. 2011 May 9;12:141. doi: 10.1186/1471-2105-12-141 (PMC3098182; doi:10.1186/1471-2105-12-141)
Supplement: Additional file 9 — Visualization of the distribution of probability weights of the modules across the analyzed genomes. The unclustered heatmap indicates the strengths of probability weights of the 198 modules across the genomes. Rows represent the genomes, whereas columns represent the weights of the modules. The brighter the color of a cell, the larger is the probability weight for the respective PF-module. We re-scaled the values of each row, using minimum and maximum values, to fit values to the interval [0,1]. A discussion of this heatmap is part of the Supplementary note in Additional file 7. [file 1471-2105-12-141-S9.PDF]

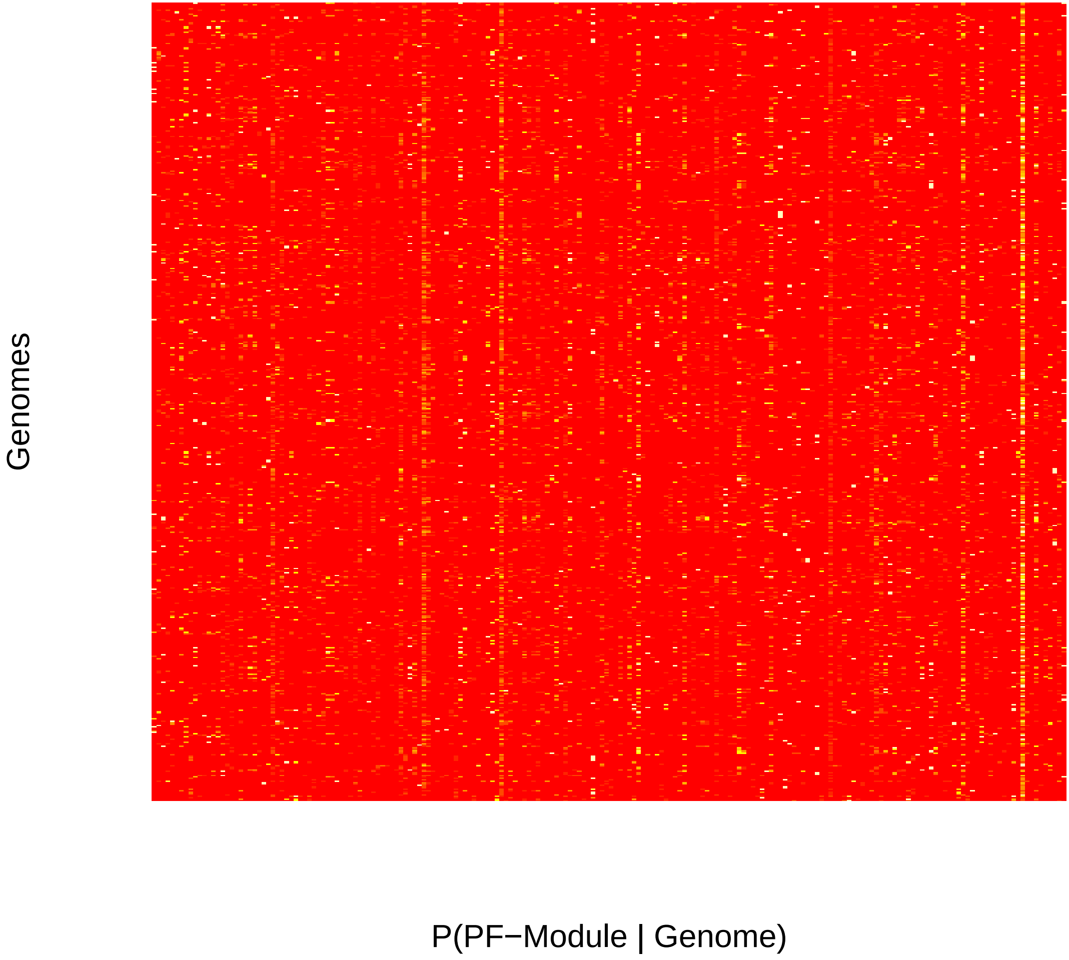
Genomes
P(PF−Module | Genome)
